# Two genes involved in clindamycin resistance of *Bacillus licheniformis* and *Bacillus paralicheniformis* identified by comparative genomic analysis

**DOI:** 10.1371/journal.pone.0231274

**Published:** 2020-04-09

**Authors:** Do-Won Jeong, Byunghoon Lee, Sojeong Heo, Yeongmin Oh, Ganghun Heo, Jong-Hoon Lee

**Affiliations:** 1 Department of Food and Nutrition, Dongduk Women’s University, Seoul, Republic of Korea; 2 Department of Food Science and Biotechnology, Kyonggi University, Suwon, Republic of Korea; Kyungpook National University, REPUBLIC OF KOREA

## Abstract

We evaluated the minimum inhibitory concentrations of clindamycin and erythromycin toward 98 *Bacillus licheniformis* strains isolated from several types of fermented soybean foods manufactured in several districts of Korea. First, based on recent taxonomic standards for bacteria, the 98 strains were separated into 74 *B*. *licheniformis* strains and 24 *B*. *paralicheniformis* strains. Both species exhibited profiles of erythromycin resistance as an acquired characteristic. *B*. *licheniformis* strains exhibited acquired clindamycin resistance, while *B*. *paralicheniformis* strains showed unimodal clindamycin resistance, indicating an intrinsic characteristic. Comparative genomic analysis of five strains showing three different patterns of clindamycin and erythromycin resistance identified 23S rRNA (adenine 2058-N6)-dimethyltransferase gene *ermC* and spermidine acetyltransferase gene *speG* as candidates potentially involved in clindamycin resistance. Functional analysis of these genes using *B*. *subtilis* as a host showed that *ermC* contributes to cross-resistance to clindamycin and erythromycin, and *speG* confers resistance to clindamycin. *ermC* is located in the chromosomes of strains showing clindamycin and erythromycin resistance and no transposable element was identified in its flanking regions. The acquisition of *ermC* might be attributable to a homologous recombination. *speG* was identified in not only the five genome-analyzed strains but also eight strains randomly selected from the 98 test strains, and deletions in the structural gene or putative promoter region caused clindamycin sensitivity, which supports the finding that the clindamycin resistance of *Bacillus* species is an intrinsic property.

## Introduction

Recently, the gastrointestinal (GI) tract has been speculated to be a potent reservoir of antibiotic-resistance genes, and the food chain is considered a possible transfer route for antibiotic resistance from animal- and environment-associated antibiotic-resistant bacteria into the human GI tract where antibiotic-resistance genes may be transferred to pathogenic and opportunistic bacteria [1]. Thus, food fermentation starters with antibiotic resistance genes can act as possible reservoirs and vehicles for transfer of antibiotic resistance determinants to microbiota in fermented foods, and these determinants can then spread to humans through food consumption. In this context, the European Food Safety Authority requires the absence of any acquired genes for antimicrobial resistance that could cause potential risks to humans when implementing a strain for human use. Although the spread of antibiotic-resistant bacteria in food ecosystems is becoming a global concern, only pathogenic bacteria and lactic acid bacteria have received particular attention, and limited information is available about the antibiotic resistance of *Bacillus* spp.

*Bacillus* spp. have been reported as populous bacteria present in alkaline-fermented foods from Asia and Africa, especially in Asian fermented soybean foods [2]. In this context, they have been considered to be potential candidate starter cultures for Asian and African fermented food production. *Bacillus* spp. are also used for biotechnological applications, including as probiotic dietary supplements for humans and animal feed inoculants, based on their ability to stimulate the immune system and produce antimicrobial compounds inhibiting pathogenic microorganisms [3].

*B*. *licheniformis* is an extensively used species in many bioindustries. *B*. *licheniformis* can be intentionally added to foods or feeds in the European Union based on the European Food Safety Authority’s qualification of the species as a safe biological agent [4], and the US Food and Drug Administration allows genetically modified strains of this species to be used for enzyme production.

*B*. *licheniformis* has been isolated as a predominant species in fermented soybean foods from Korea and exhibits the highest salt tolerance among isolated *Bacillus* spp. [5]. Based on the salt tolerance and high protease activity, we considered that *B*. *licheniformis* could be a potential starter culture candidate for high-salt soybean fermentations. We assessed the antibiotic susceptibilities and technological properties of 94 *B*. *licheniformis* isolates from our stock cultures to select a safe and functional candidate starter strain [6]. The minimum inhibitory concentrations (MICs) of eight tested antibiotics (chloramphenicol, clindamycin, erythromycin, gentamicin, kanamycin, streptomycin, tetracycline, and vancomycin) were determined for the 94 isolates; clindamycin and erythromycin susceptibility profiles exhibited acquired resistance properties. However, *B*. *licheniformis* strains from African traditional bread showed intrinsic resistance to clindamycin [7]. Acquired antibiotic resistance occurs as a result of either genetic mutation of pre-existing genes or horizontal transfer of new genes. Intrinsic antibiotic resistance is a natural insensitivity in bacteria that have never been susceptible to a particular antibiotic. Possession of an intrinsic antibiotic resistance gene is not a safety concern in the selection of bacterial strains for human use.

In staphylococci, resistance to clindamycin and erythromycin was reported to occur through methylation of their ribosomal target sites [8]. The ribosomal target site modification mechanism, so-called macrolide-lincosamide-streptogramin B (MLS_B_) resistance, results in cross-resistance to erythromycin, clindamycin, and streptogramin B. Such resistance is typically mediated by erythromycin ribosome methylase (Erm) genes and is the most widespread resistance mechanism to macrolides and lincosamides [9].

To clarify the genomic background explaining clindamycin resistance of *B*. *licheniformis*, we evaluated the MICs of clindamycin and erythromycin toward an additional 98 *B*. *licheniformis* strains isolated in Korea and undertook comparative genomic analysis of five strains exhibiting different patterns of clindamycin and erythromycin resistance.

## Materials and methods

### *Bacillus* strains and cultures

To evaluate the MICs of clindamycin and erythromycin for *B*. *licheniformis* with diverse strains, we collected 42 and 56 *B*. *licheniformis* strains from the Korea Food Research Institute (http://www.kfri.re.kr) and the Microbial Institute for Fermentation Industry (http://mifi.kr), respectively. The strains were isolated from several types of fermented soybean foods manufactured in several districts of Korea.

Genome sequence-published strains *B*. *licheniformis* DSM 13^T^ (KCTC 1918^T^) and *B*. *paralicheniformis* KJ-16^T^ (KACC 18426^T^) were purchased from the Korean Collection for Type Cultures (KCTC; http://kctc.kribb.re.kr/) and the Korean Agricultural Culture Collection (KACC; http://genebank.rda.go.kr/), respectively. Genome sequence-published strains *B*. *licheniformis* 14ADL4 (KCTC 33983), *B*. *licheniformis* 0DA23-1 (KCTC 43013), and *B*. *paralicheniformis* 14DA11 (KCTC 33996) were isolated from traditional Korean fermented soybean foods and are deposited at KCTC [10–12]. All *Bacillus* strains were cultured in tryptic soy agar (TSA; BD Diagnostic Systems, Sparks, MD, USA) and tryptic soy broth (TSB; BD Diagnostic Systems) at 30°C for 24 h.

### Taxonomic identity confirmation of *B*. *licheniformis* strains

The identity of 98 strains was confirmed by *spo0A* (stage 0 sporulation protein A) gene sequence analysis [13].

### Determination of MICs

MICs of clindamycin and erythromycin were determined by the broth microdilution method according to the guidelines of the Clinical and Laboratory Standards Institute [14]. A twofold serial dilution was prepared for each antibiotic in deionized water, and final concentrations in each well of the microplate ranged between 0.5 and 4096 mg/L. Tested strains were cultured twice in TSB and matched to a 0.5 McFarland turbidity standard (bioMérieux, Marcy l’Étoile, France). The cultured strains were further diluted (1:100) in cation-adjusted Mueller-Hinton broth (Oxoid, Basingstoke, Hants, UK) to achieve the desired inoculum concentration. The final inoculum density was 5×10^5^ colony-forming units/mL in each well in 96-microwell plates. Microwell plates were incubated at 35°C for 18 h to determine the MICs of clindamycin and erythromycin. The MIC of each antibiotic was recorded as the lowest concentration where no growth was observed in the wells after incubation for 18 h. MIC results were confirmed by at least three independently performed tests.

### Comparative genomic analysis

For comparative genomic analysis of the genomes of *Bacillus* strains showing different clindamycin and erythromycin resistance patterns, complete genome sequence data for *B*. *paralicheniformis* 14DA11 (C^R^E^R^, clindamycin and erythromycin resistance; GenBank accession, NZ_CP023168), *B*. *paralicheniformis* KJ-16^T^ (C^R^E^R^; LBMN02000039), *B*. *licheniformis* DSM 13^T^ (C^R^E^S^, clindamycin resistance and erythromycin sensitivity; NC_006270), *B*. *licheniformis* 14ADL4 (C^R^E^S^; CP026673), and *B*. *licheniformis* 0DA23-1 (C^S^E^S^, clindamycin and erythromycin sensitivity; CP031126) were obtained from NCBI (http://ncbi.nlm.nih.gov/genomes). The Efficient Database framework for comparative Genome Analyses using BLAST score Ratios (EDGAR) was used for core genome, pan-genome, and singleton analyses [15]; the genome of strain 0DA23-1 was used as a reference genome for Venn diagram construction in analysis of the five genomes. Comparative analyses at the protein level were performed by an all-against-all comparison of the annotated genomes. The algorithm used was BLASTP and data were normalized according to the best score [16]. The score ratio value, which shows the quality of the hit, was calculated by dividing the scores of further hits by that for the best hit [17]. Two genes were considered orthologous when a bidirectional best BLAST hit with a single score ratio value threshold of ≥32% was obtained in orthology estimation.

### Identification of potential clindamycin resistance genes

Genomic DNA of *Bacillus* strains was extracted using a DNeasy Tissue Kit (Qiagen, Hilden, Germany). Identification of potential clindamycin resistance genes was performed by PCR amplification with specific primer sets ermC-106/ermC-535, ereAB-229/ereAB-806, and speG-23/speG-243 (Table 1) using a T-3000 Thermocycler (Biometra, Gottingen, Germany). PCR mixtures consisted of template DNA, 0.5 μM of each primer, 1.25 units of *Taq* polymerase (Inclone Biotech, Daejeon, Korea), 10 mM dNTPs, and 2 mM MgCl_2_. Samples were preheated for 5 min at 95°C and then amplified using 30 cycles of 1 min at 95°C, 30 s at 55°C, and 1 min at 72°C. Amplified PCR products were sequenced using a custom service provided by Bionics (Seoul, Korea). The web-hosted BLAST program was used to find sequence homologies of the amplified fragments with known gene sequences in the NCBI database. For the analysis of putative promoter sequences of *speG*, the primer set PspeG-Up/PspeG-Down was designed based on the genome sequence of strain 14DA11 (Table 1).

**Table 1:** Oligonucleotides used in this study.

Primer	Sequence (5ʹ–3ʹ)	Use
ermC-106	ATTGTGGATCGGGCAAATATT	*ermC* identification
ermC-535	TGGAGGGGGAGAAAAATG	*ermC* identification
ereAB-229	CTGCATCAGGAATTAGGATTTC	*ereAB* identification
ereAB-806	TTGATGTGAAGGTTGTGAGCC	*ereAB* identification
speG-23	GTCCGCTTGAAAGAGAAGACC	*speG* identification
speG-243	TTATGATTTGAAACTCCGCC	*speG* identification
ermC-Up	TCCCCCGGGGATGAGAGGAAGAGGAAACATG	*ermC* cloning
ermC-Down	GAAGATCTTATTTCTCCGGGTTTTCGCTTATTTGC	*ermC* cloning
ereAB-Up	CCGATATCGGAGAGATTCAAGAGATGGGCAACC	*ereAB* cloning
ereAB-Down	GAAGATCTCCTTAGAGGTTATGCTTAACCCGTC	*ereAB* cloning
speG-Up	CCGATATCGATCGGCCTGCGCCTTACATCAT	*speG* cloning
speG-Down	GAAGATCTGGGAAGAGGTTGACAAAGACG	*speG* cloning
pCL55-itet-F	GGCCCTTTCGTCTTCAAGAAT	Cloned DNA sequence confirmation
pCL55-itet-R	ATTTTACATCCCTCCGGATCC	Cloned DNA sequence confirmation
PspeG-Up	GTTCGCTTAGTGCTCTGGTGATC	Putative *speG* promoter identification
PspeG-Down	GCGGACGCAATTTAAGCTGATT	Putative *speG* promoter identification

Restriction sites are underlined. The boxed sequence is the deleted region of *speG* absent from the gene in strain 0DA23-1.

### Cloning of potential clindamycin resistance genes

Total DNAs of *B*. *paralicheniformis* strains 14DA11 and KJ-16^T^ were used as the templates for PCR amplification of potential clindamycin resistance genes. The candidates, 23S rRNA (adenine 2058-N6)-dimethyltransferase gene (*ermC*), erythromycin esterase gene (*ereAB*), and spermidine acetyltransferase gene (*speG*), were amplified with primer sets ermC-Up/ermC-Down, ereAB-Up/ereAB-Down, and speG-Up/speG-Down, respectively (Table 1). Each primer contained a restriction enzyme site: *Sma*I in ermC-Up; *Eco*RV in ereAB-Up and speG-Up; and *Bgl*II in ermC-Down, ereAB-Down, and speG-Down. The amplified PCR products were double-digested with *Sma*I/*Bgl*II or *Eco*RV/*Bgl*II and then inserted into *Eco*RV and *Bgl*II digested pCL55-itet under the control of a tetracycline inducible promoter [18]. The successful integration of the target fragments was confirmed by sequence analysis using primer set pCL55-itet-F/pCL55-itet-R. Plasmid DNA was introduced into *B*. *subtilis* ISW1214 by electroporation [19] with a gene pulser (Bio-Rad, Hercules, CA, USA).

### Cell growth monitoring in the presence of clindamycin and erythromycin

Transformants cultured in TSB were normalized to 0.5 turbidity at OD_600_ and then diluted 1:100 in TSB supplemented with clindamycin or erythromycin to check the function of candidate genes. Clindamycin and erythromycin were employed at 32 μg/mL. Cell growth was monitored by measuring OD_600_ using a Varioskan Flash (Thermo Scientific, Waltham, MA, USA). All analyses were performed in triplicate on independent samples prepared in the same way.

## Results and discussion

### Taxonomic status of 98 *B*. *licheniformis* strains

Recently, *B*. *paralicheniformis* was separated from *B*. *licheniformis* on the basis of phylogenomic and phylogenetic studies [20]. *spo0A* sequence analysis separated 24 strains as *B*. *paralicheniformis* from the 98 strains previously identified as *B*. *licheniformis*.

### Clindamycin and erythromycin resistance of *B*. *licheniformis* and *B*. *paralicheniformis*

The MICs of clindamycin and erythromycin toward the 74 *B*. *licheniformis* strains and 24 *B*. *paralicheniformis* strains are summarized in Table 2. Four *B*. *licheniformis* strains and 21 *B*. *paralicheniformis* strains were resistant to erythromycin, and their resistance profiles exhibited an acquired characteristic. More than fourfold higher resistance to clindamycin than the breakpoint was identified in 70.2% of the 74 *B*. *licheniformis* strains and the population distribution of the 74 strains was discontinuous. *B*. *paralicheniformis* strains exhibited a unimodal clindamycin resistance profile, which supports that this resistance is an intrinsic characteristic.

**Table 2:** Distribution of 74 *Bacillus licheniformis* and 24 *Bacillus paralicheniformis* strains isolated from traditional Korean fermented soybean foods over a range of minimum inhibitory concentrations (MICs) for clindamycin and erythromycin.

Species	Antibiotic	MIC (mg/L)														Breakpoint (mg/L)^a^
		0.5	1	2	4	8	16	32	64	128	256	512	1024	2048	4096	
*B*. *licheniformis*	Clindamycin	15	3	3	1		1	20	19	12						4
	Erythromycin	25	23	12	10									1	3	4
*B*. *paralicheniformis*	Clindamycin						2	5	6	11						4
	Erythromycin	0	1	2										1	20	4

^a^Breakpoint values for *Bacillus* spp. taken from EFSA [21].

If independent determinants of clindamycin and erythromycin resistance contribute to the phenotype of *Bacillus* strains, four combinations of phenotypes can be exhibited. Three such patterns of resistance (C^R^E^R^; C^R^E^S^; and C^S^E^S^) were identified among the *B*. *licheniformis* and *B*. *paralicheniformis* strains we tested (Table 3). These results stimulated further studies to illuminate the determinants and characteristics of clindamycin resistance in *B*. *licheniformis* and *B*. *paralicheniformis*.

**Table 3:** MICs of clindamycin and erythromycin, and identification of *ermC*, *ereAB*, *speG*, and putative *speG* promoter in selected *B*. *licheniformis* and *B*. *paralicheniformis* strains.

Phenotype	Species	Strain	GenBank accession no.	MIC (mg/L)		Gene identification			
				Clindamycin	Erythromycin	*ermC*	*ereAB*	*speG*	Promoter^a^
C^R^E^R^	*B*. *paralicheniformis*	14DA11	CP023168	32	2048	+	–	+	–
	*B*. *paralicheniformis*	KJ-16^T^	LBMN00000000	16	4096	+	+	+	–
	*B*. *paralicheniformis*	SRCM100038	–	128	4096	+	+	+	–
	*B*. *licheniformis*	SRCM100160	–	128	4096	+	–	+	+
	*B*. *licheniformis*	SRCM100163	–	32	2048	+	+	+	+
C^R^E^S^	*B*. *licheniformis*	14ADL4	CP026673	64	0.5	–	–	+	+
	*B*. *licheniformis*	DSM 13^T^	CP000002.3	128	0.5	–	–	+	+
	*B*. *paralicheniformis*	CHKJ1206-1	–	16	2	–	–	+	+
	*B*. *paralicheniformis*	CHKJ1310	–	16	1	–	–	+	+
	*B*. *licheniformis*	TPP0006	–	64	2	–	–	+	+
C^S^E^S^	*B*. *licheniformis*	0DA23-1	CP031126	0.5	0.5	–	–	ND	+
	*B*. *licheniformis*	F1082	–	0.5	2	–	–	ND	+
	*B*. *licheniformis*	SRCM100107	–	0.5	0.5	–	–	ND	+

Strains with names beginning “SCRM” were selected from 56 strains kindly provided by the Microbial Institute for Fermentation Industry. Strains CHKJ1206-1, CHKJ1310, TPP0006, and F1082 were selected from 42 strains kindly provided by the Korea Food Research Institute.

ND means nucleotide deletions occurred in the open reading frame. ^a^Identification of a putative promoter sequence upstream of *speG*.

### Genomic insight into clindamycin resistance

To shed light on the genetic background behind clindamycin resistance of *B*. *licheniformis* and *B*. *paralicheniformis*, we attempted comparative genome analysis of five strains showing different clindamycin and erythromycin resistance patterns (S1 Table).

**Fig 1:**
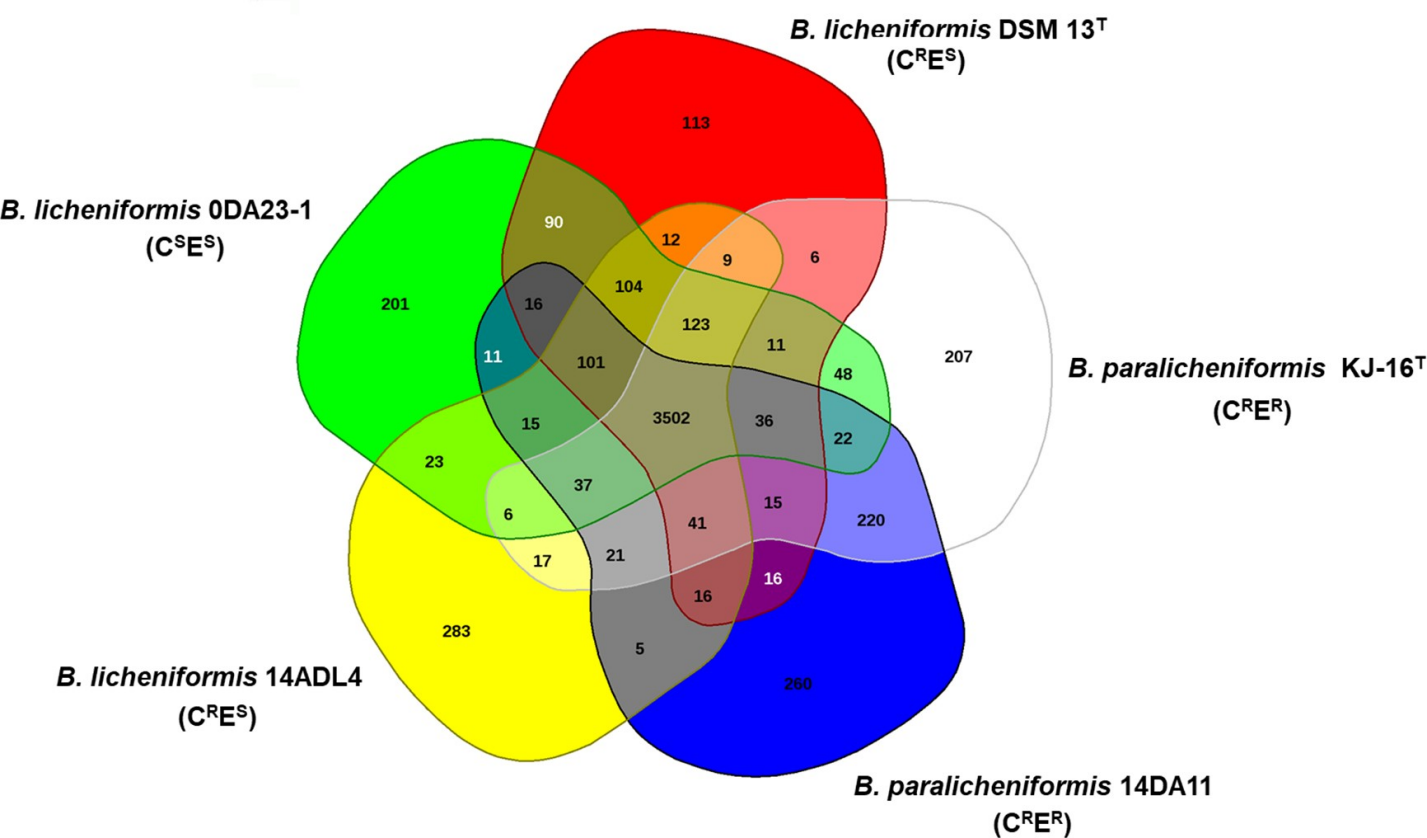
Venn diagram of five *Bacillus* genomes (*B*. *licheniformis* and *B*. *paralicheniformis*). The Venn diagram shows the pan-genome of strains 0DA23-1, DSM 13^T^, KJ-16 ^T^, 14DA11, and 14ADL4 generated using EDGAR. Overlapping regions represent common coding sequences (CDSs) shared between the genomes. The numbers outside the overlapping regions indicate the numbers of CDSs in each genome without homologs in the other genomes.

The gene pools shared by the genomes of the five strains—*B*. *paralicheniformis* 14DA11 (C^R^E^R^), *B*. *paralicheniformis* KJ-16^T^ (C^R^E^R^), *B*. *licheniformis* DSM 13^T^ (C^R^E^S^), *B*. *licheniformis* 14ADL4 (C^R^E^S^), and *B*. *licheniformis* 0DA23-1 (C^S^E^S^)—are depicted in a Venn diagram (Fig 1). The five strains share 3,502 protein-coding sequences (CDSs) in their core genome, corresponding to 73.5%–81.7% of their own open reading frames. Many of the CDSs in the core genome were assigned via Clusters of Orthologous Groups annotation to functions relating to metabolism and the transport of amino acids and carbohydrates.

Comparative genomic analysis revealed that two C^R^E^R^ strains, KJ-16^T^ and 14DA11, shared 220 CDSs that are absent from the other three analyzed *Bacillus* genomes (Fig 1). The shared CDSs correspond to 4.6% and 4.8% of the total CDSs, respectively, and include an annotated *ermC* (S2 Table). A variety of *erm* genes have been reported in a large number of microorganisms and *ermC* is a typical staphylococcal gene class that can endow MLS_B_ resistance [22]. PCR amplification with the *ermC*-specific primer set ermC-106/ermC-535 confirmed the presence of *ermC* in strains exhibiting the C^R^E^R^ phenotype (Tables 1 and 3). *ermC* potentially contributes to the clindamycin resistance of *B*. *licheniformis* and *B*. *paralicheniformis*.

An annotated *ereAB* was identified only in the genome of strain KJ-16^T^ (C^R^E^R^) and this gene was amplified from some of the C^R^E^R^ strains with an *ereAB*-specific primer set (Tables 1 and 3). Erythromycin esterase was reported to confer erythromycin resistance through erythromycin esterification [23].

Comparative genomic analysis revealed that two C^R^E^S^ strains, DSM13^T^ and 14ADL4, have 12 CDSs that are shared only between them (Fig 1 and S3 Table). Four of these CDSs were predicted hypothetical protein-encoding genes. Six CDSs were homologs of multicopy genes and homologs were identified in the genomes of strains 0DA23-1, 14DA11, and KJ-16^T^. The other two genes were a putative lysine transporter gene and a DNA (cytosine-5-)-methyltransferase gene. However, we could not find any earlier report to correlate those genes with clindamycin resistance.

Erm proteins were reported to dimethylate a single adenine in nascent 23S rRNA, which is part of the large 50S ribosomal subunit [8]. Schlunzen et al. [24] reported that the nucleotide residues G2057, A2058, A2059, A2503, A2505, and C2611 of the *Escherichia coli* 23S rRNA sequence are interaction sites with the lincosamide clindamycin and nucleotide residues G2057, A2058, A2059, A2062, A2505, and C2609 interact with the macrolide erythromycin. We could not find any difference in the corresponding base residues of the 23S rRNA sequences of the five genome-analyzed *B*. *licheniformis* and *B*. *paralicheniformis* strains (S4 Table). This finding supports that 23S rRNA gene mutation was not the cause of clindamycin or erythromycin resistance in these strains.

**Fig 2:**
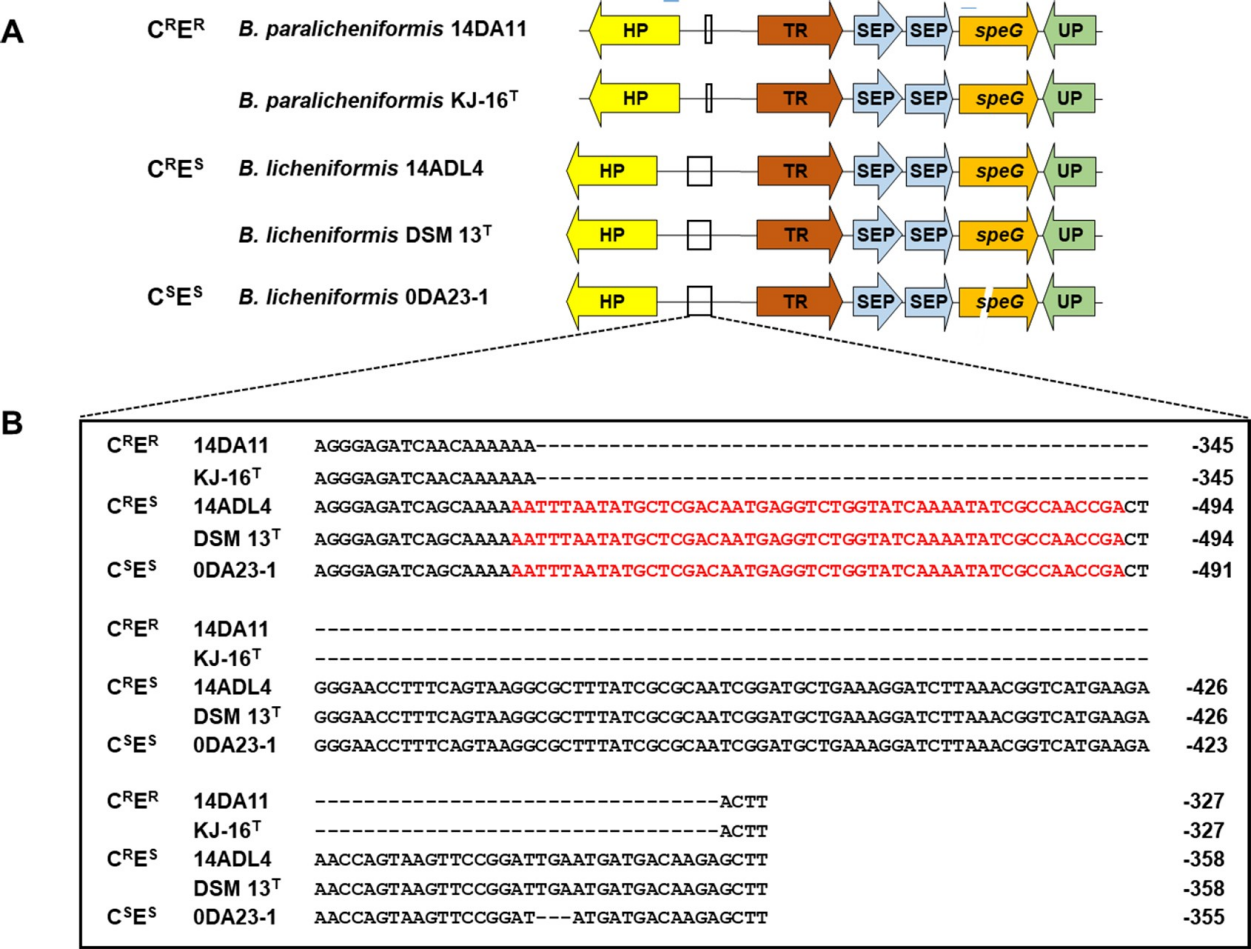
**Genetic structures surrounding the *speG* genes (A) and the putative *speG* promoter sequences (B) in *Bacillus* strains.** Abbreviations: HP, hypothetical protein gene; TR, TetR/AcrR family transcriptional regulator gene; SEP, spermidine export protein gene; *speG*, spermidine acetyltransferase gene; UP, uncharacterized protein gene. Red letters indicate the putative promoter sequences detected using PromoterHunter software. Negative numbers show the upstream locations from TetR/AcrR family transcriptional regulator genes.

We found a report of synergistic effects between biogenic polyamines (e.g., spermidine and spermine) and antibiotics on the antibiotic susceptibility of clinically-relevant bacteria [25]. The synergistic effect of spermidine with clindamycin, gentamicin, and mupirocin, all of which are important antibiotics for treatment of staphylococcal infection, was disrupted by the presence of *speG* [26]. A putative *speG* was identified in all five *Bacillus* genomes. Strain 0DA23-1 (C^S^E^S^) possessed a frame-shifted *speG* with a deletion of 11 nucleotides, while the other four strains (C^R^E^R^ and C^R^E^S^) possessed *speG* with the same DNA sequence as each other (Fig 2). We identified *speG* in eight strains randomly selected from the 98 *Bacillus* strains by PCR amplification with primer set speG-23/speG-243, which was designed to encompass the deleted region of *speG* as observed in strain 0DA23-1 (Table 1). The gene was identified in all C^R^E^R^ and C^R^E^S^ strains, while an amplicon was not produced from strains F1082 and SRCM100107, which have a C^S^E^S^ phenotype (Table 3).

To find clues about how *speG* may have been acquired, we analyzed DNA sequences of the flanking regions of *speG* in five genome-analyzed strains (Fig 2). The five strains had the same genetic organization around *speG* in their chromosome and mobile elements were not found around the gene. However, DNA sequence differences were identified in their putative promoter sequences, detected using PromoterHunter software [27]. Two C^R^E^R^ strains, 14DA11 and KJ-16^T^, lacked the putative promoter region, while three strains sensitive to erythromycin (C^R^E^S^ and C^S^E^S^) possessed this region. The clindamycin resistance of the two C^R^E^R^ strains might be attributable to the expression of *ermC*. To determine whether promoter deletion is a common event in *B*. *licheniformis* and *B*. *paralicheniformis* C^R^E^R^ strains, we analyzed the promoter region of eight more strains showing phenotypes C^R^E^R^, C^R^E^S^, and C^S^E^S^ randomly selected from the 98 MIC-tested strains (Table 3). Two C^R^E^R^ *B*. *licheniformis* strains possessing the promoter were identified. These results indicated that the expression of *speG* can contribute to the C^R^E^R^ phenotype in *Bacillus* species, but the phenotype mainly depends on possession of *ermC*. The C^R^E^S^ phenotype may be attributed to the expression of *speG* in the absence of *ermC*.

### Functional analysis of potential clindamycin resistance genes

**Fig 3:**
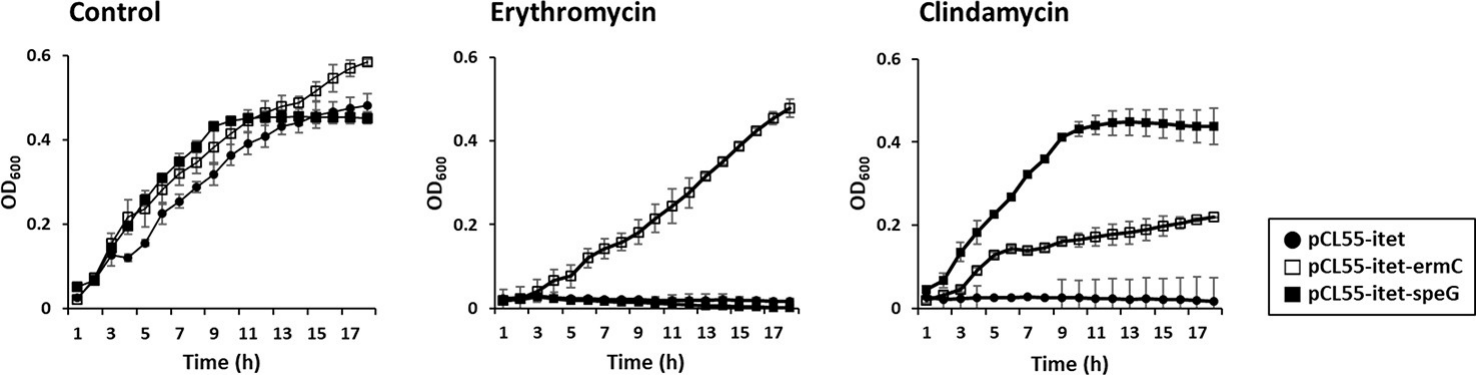
Effects of *ermC* and *speG* on the growth of *B*. *subtilis* ISW1214 transformants under clindamycin and erythromycin stress.

To confirm the function of *ermC*, the locus CK945_RS15790 of strain 14DA11 was amplified and inserted into pCL55-itet under the control of a tetracycline inducible promoter (Table 4). The resulting plasmid was designated pCL55-itet-ermC. *B*. *subtilis* ISW1214 harboring pCL55-itet-ermC grew in the presence of 32 μg/mL erythromycin, while the host containing pCL55-itet did not grow under erythromycin pressure (Fig 3). The *ermC*-expressing transformant also grew in the presence of 32 μg/mL clindamycin. The MICs of clindamycin and erythromycin for *B*. *subtilis* ISW1214 containing pCL55-itet-ermC were 512 mg/L and 4096 mg/L, respectively. These results confirmed that *ermC* was responsible for the cross-resistance of *B*. *licheniformis* and *B*. *paralicheniformis* to erythromycin and clindamycin.

**Table 4:** Potential clindamycin resistance determinants identified in five *Bacillus* genomes.

Product	Gene	Gene locus				
		DSM 13^T^	14ADL4	0DA23-1	14DA11	KJ-16^T^
23S rRNA (adenine 2058-N6)-dimethyltransferase	*ermC*				CK945_RS15790	ACH97_219465
Erythromycin esterase	*ereAB*					ACH97_221905
Spermidine acetyltransferase	*speG*	TRNA_RS31465	BL14DL4_00234	BLDA23_RS10890	CK945_RS11785	ACH97_208005

The locus CK945_RS11785 of strain 14DA11 was amplified and used to construct a recombinant plasmid containing *speG* in the same way as pCL55-itet-ermC was constructed. The resulting plasmid was named pCL55-itet-speG. The transformant harboring pCL55-itet- speG grew in the presence of 32 μg/mL clindamycin, but did not grow under 32 μg/mL erythromycin pressure (Fig 3). The MIC of clindamycin for *B*. *subtilis* ISW1214 containing pCL55-itet-speG was >512 mg/L. Thus, *speG* was proved to endow clindamycin resistance to *B*. *licheniformis* and *B*. *paralicheniformis*.

The locus ACH97_221905 of strain KJ-16^T^ was amplified and used to construct recombinant plasmid pCL55-itet-ereAB containing *ereAB*. The transformant harboring pCL55-itet-ereAB did not exhibit clindamycin or erythromycin resistance.

### The intrinsic gene *speG* endows clindamycin resistance

Several studies have reported that *erm* genes located in mobile elements such as plasmid pE194 contribute to erythromycin resistance of bacteria [7, 28–30] and these genes can also endow clindamycin resistance [9]. In this study, we also found that *ermC* causes erythromycin and clindamycin resistance in *B*. *licheniformis* and *B*. *paralicheniformis*, while the gene is located not in mobile elements but in the chromosomes of C^R^E^R^ strains (S1 Fig). No transposable element was identified in the flanking regions of *ermC*, which suggests that insertion events by mobile elements are not the cause of *ermC* acquisition. The acquisition of *ermC* might be attributable to a homologous recombination.

Although the possible working mechanism of *speG* in clindamycin resistance of *B*. *licheniformis* and *B*. *paralicheniformis* was not postulated owing to clindamycin and spermidine being chemically distinct, this study is the first to illuminate that *speG* contributes to the clindamycin resistance of *B*. *licheniformis* and *B*. *paralicheniformis* and the gene was detected in not only the five genome-analyzed strains but also in eight strains randomly selected from the 98 *Bacillus* test strains (Table 3). We found that nucleotide deletions in this gene caused clindamycin sensitivity. We also found strains with a deletion in the putative promoter region of *speG*; such deletion caused clindamycin sensitivity. This study proved that clindamycin resistance of *Bacillus* species is an intrinsic property which can be lost by deletion events in the structural gene or promoter of *speG*. Therefore, clindamycin resistance of *B*. *licheniformis* and *B*. *paralicheniformis* is not a safety concern in the selection of bacterial strains for human use. However, their erythromycin susceptibility needs to be evaluated to clarify the determinants acquisition.

## Supporting information

S1 Table Partial list of CDSs in the pan-genome of five *Bacillus* strains. (DOCX)

S2 Table List of CDSs identified only in the genomes of C^R^E^R^ strains 14DA11 and KJ-16^T^. (DOCX)

S3 Table List of CDSs identified only in the genomes of C^R^E^S^ strains DSM 13^T^ and 14ADL4. (DOCX)

S4 Table Predicted interaction sites of clindamycin and erythromycin in the 23S rRNA of *B*. *licheniformis* and *B*. *paralicheniformis*. (DOCX)

S1 Fig Structures of the genes surrounding erythromycin resistance gene *ermC* in five *Bacillus* genomes. (DOCX)
