# Supplementary material for: Two genes involved in clindamycin resistance of Bacillus licheniformis and Bacillus paralicheniformis identified by comparative genomic analysis
Source: PLoS One. 2020 Apr 9;15(4):e0231274. doi: 10.1371/journal.pone.0231274 (PMC7144989; doi:10.1371/journal.pone.0231274)
Supplement: S1 Table — (DOCX) [file pone.0231274.s001.docx]

**S1 Table. Partial list of CDSs in the pan-genome of five *Bacillus* strains.**

|  | DSM 13^T^ | 14ADL4 | 0DA23-1 | 14DA11 | KJ-16^T^ |
| --- | --- | --- | --- | --- | --- |
| chromosomal replication initiator protein DnaA | TRNA_RS21520 | BL14DL4_02596 | BLDA23_00005 | CK945_RS00005 | ACH97_211950 |
| DNA polymerase III subunit beta | TRNA_RS21525 | BL14DL4_02597 | BLDA23_00010 | CK945_RS00010 | ACH97_211945 |
| S4 domain-containing protein YaaA | TRNA_RS21530 | BL14DL4_02598 | BLDA23_00015 | CK945_RS00015 | ACH97_211940 |
| DNA replication/repair protein RecF | TRNA_RS21535 | BL14DL4_02599 | BLDA23_00020 | CK945_RS00020 | ACH97_211935 |
| DNA topoisomerase (ATP-hydrolyzing) subunit B | TRNA_RS21545 | BL14DL4_02600 | BLDA23_00030 | CK945_RS00030 | ACH97_211925 |
| DNA gyrase subunit A | TRNA_RS21550 | BL14DL4_02601 | BLDA23_00035 | CK945_RS00035 | ACH97_211920 |
| hypothetical protein | TRNA_RS21580 | BL14DL4_02607 | BLDA23_00065 | CK945_RS00065 | ACH97_222465 |
| IMP dehydrogenase | TRNA_RS21585 | BL14DL4_02608 | BLDA23_00070 | CK945_RS00070 | ACH97_222460 |
| D-alanyl-D-alanine carboxypeptidase | TRNA_RS21590 | BL14DL4_02609 | BLDA23_00075 | CK945_RS00075 | ACH97_222455 |
| pyridoxal 5-phosphate synthase lyase subunit PdxS | TRNA_RS21595 | BL14DL4_02610 | BLDA23_00080 | CK945_RS00080 | ACH97_222450 |
| pyridoxal 5-phosphate synthase glutaminase subunit PdxT | TRNA_RS21600 | BL14DL4_02611 | BLDA23_00085 | CK945_RS00085 | ACH97_222445 |
| serine--tRNA ligase | TRNA_RS21605 | BL14DL4_02612 | BLDA23_00090 | CK945_RS00090 | ACH97_222440 |
| glycerate kinase | TRNA_RS21610 | BL14DL4_02613 | BLDA23_00095 | CK945_RS00095 | ACH97_222435 |
| YsfB protein | TRNA_RS21620 | BL14DL4_02615 | BLDA23_00105 | CK945_RS00105 | ACH97_222425 |
| deoxynucleoside kinase | TRNA_RS21635 | BL14DL4_02618 | BLDA23_00120 | CK945_RS00120 | ACH97_222410 |
| deoxyguanosine kinase | TRNA_RS21640 | BL14DL4_02619 | BLDA23_00125 | CK945_RS00125 | ACH97_222405 |
| LysM peptidoglycan-binding domain-containing protein | TRNA_RS21645 | BL14DL4_02620 | BLDA23_00130 | CK945_RS00130 | ACH97_222400 |
| cysteine hydrolase | TRNA_RS21650 | BL14DL4_02621 | BLDA23_00135 | CK945_RS00135 | ACH97_222395 |
| nucleoside deaminase | TRNA_RS21655 | BL14DL4_02622 | BLDA23_00140 | CK945_RS00140 | ACH97_222390 |
| DNA polymerase III subunit gamma/tau | TRNA_RS21660 | BL14DL4_02623 | BLDA23_00150 | CK945_RS00150 | ACH97_222385 |
| YbaB/EbfC family nucleoid-associated protein | TRNA_RS21665 | BL14DL4_02624 | BLDA23_00155 | CK945_RS00155 | ACH97_222380 |
| recombination protein RecR | TRNA_RS21670 | BL14DL4_02625 | BLDA23_00160 | CK945_RS00160 | ACH97_222375 |
| DUF2508 family protein | TRNA_RS21675 | BL14DL4_02626 | BLDA23_00165 | CK945_RS00165 | ACH97_222370 |
| pro-sigmaK processing inhibitor BofA | TRNA_RS21680 | BL14DL4_02627 | BLDA23_00170 | CK945_RS00170 | ACH97_222365 |
| protein xpaC | TRNA_RS21710 | BL14DL4_02634 | BLDA23_00205 | CK945_RS00205 | ACH97_205760 |
| toxic anion resistance protein | TRNA_RS21715 | BL14DL4_02635 | BLDA23_00210 | CK945_RS00210 | ACH97_205765 |
| aminotransferase class I/II-fold pyridoxal phosphate-dependent enzyme | TRNA_RS21720 | BL14DL4_02636 | BLDA23_00215 | CK945_RS00215 | ACH97_205770 |
| dTMP kinase | TRNA_RS21725 | BL14DL4_02637 | BLDA23_00220 | CK945_RS00220 | ACH97_205775 |
| hypothetical protein | TRNA_RS21730 | BL14DL4_02638 | BLDA23_00225 | CK945_RS00225 | ACH97_205780 |
| DUF327 family protein | TRNA_RS21735 | BL14DL4_02639 | BLDA23_00230 | CK945_RS00230 | ACH97_205785 |
| DNA polymerase III subunit delta | TRNA_RS21740 | BL14DL4_02640 | BLDA23_00235 | CK945_RS00235 | ACH97_205790 |
| stage 0 sporulation protein | TRNA_RS21745 | BL14DL4_02641 | BLDA23_00240 | CK945_RS00240 | ACH97_205795 |
| DNA replication initiation control protein YabA | TRNA_RS21750 | BL14DL4_02642 | BLDA23_00245 | CK945_RS00245 | ACH97_205800 |
| tRNA1(Val) (adenine(37)-N6)-methyltransferase | TRNA_RS21755 | BL14DL4_02643 | BLDA23_00250 | CK945_RS00250 | ACH97_205805 |
| GIY-YIG nuclease family protein | TRNA_RS21760 | BL14DL4_02644 | BLDA23_00255 | CK945_RS00255 | ACH97_205810 |
| AbrB/MazE/SpoVT family DNA-binding domain-containing protein | TRNA_RS21770 | BL14DL4_02646 | BLDA23_00265 | CK945_RS00265 | ACH97_205820 |
| methionine--tRNA ligase | TRNA_RS21775 | BL14DL4_02647 | BLDA23_00270 | CK945_RS00270 | ACH97_205825 |
| YchF/TatD family DNA exonuclease | TRNA_RS21780 | BL14DL4_02648 | BLDA23_00275 | CK945_RS00275 | ACH97_205830 |
| DUF348 domain-containing protein | TRNA_RS21785 | BL14DL4_02649 | BLDA23_00280 | CK945_RS00280 | ACH97_205835 |
| ribonuclease M5 | TRNA_RS21790 | BL14DL4_02650 | BLDA23_00285 | CK945_RS00285 | ACH97_205840 |
| ribosomal RNA small subunit methyltransferase A | TRNA_RS21795 | BL14DL4_02651 | BLDA23_00290 | CK945_RS00290 | ACH97_205845 |
| sporulation peptidase YabG | TRNA_RS21800 | BL14DL4_02652 | BLDA23_00295 | CK945_RS00295 | ACH97_205850 |
| hypothetical protein | TRNA_RS21805 | BL14DL4_02653 | BLDA23_00300 | CK945_RS00300 | ACH97_205855 |
| small, acid-soluble spore protein, alpha/beta type | TRNA_RS21810 | BL14DL4_02654 | BLDA23_00305 | CK945_RS00305 | ACH97_205860 |
| 4-(cytidine 5-diphospho)-2-C-methyl-D-erythritol kinase | TRNA_RS21815 | BL14DL4_02656 | BLDA23_00310 | CK945_RS00310 | ACH97_205865 |
| pur operon repressor | TRNA_RS21820 | BL14DL4_02657 | BLDA23_00315 | CK945_RS00315 | ACH97_205870 |
| RidA family protein | TRNA_RS21825 | BL14DL4_02658 | BLDA23_00320 | CK945_RS00320 | ACH97_205875 |
| septation protein SpoVG | TRNA_RS21830 | BL14DL4_02659 | BLDA23_00325 | CK945_RS00325 | ACH97_205880 |
| bifunctional UDP-N-acetylglucosamine diphosphorylase/glucosamine-1-phosphate N-acetyltransferase GlmU | TRNA_RS21835 | BL14DL4_02660 | BLDA23_00330 | CK945_RS00330 | ACH97_205885 |
| ribose-phosphate diphosphokinase | TRNA_RS21840 | BL14DL4_02661 | BLDA23_00335 | CK945_RS00335 | ACH97_205890 |
| 50S ribosomal protein L25 | TRNA_RS21845 | BL14DL4_02662 | BLDA23_00340 | CK945_RS00340 | ACH97_205895 |
| peptidyl-tRNA hydrolase | TRNA_RS21850 | BL14DL4_02663 | BLDA23_00345 | CK945_RS00345 | ACH97_205900 |
| DUF2757 family protein | TRNA_RS21855 | BL14DL4_02664 | BLDA23_00350 | CK945_RS00350 | ACH97_205905 |
| stage V sporulation protein T | TRNA_RS21865 | BL14DL4_02666 | BLDA23_00360 | CK945_RS00360 | ACH97_205915 |
| polysaccharide biosynthesis protein | TRNA_RS21870 | BL14DL4_02667 | BLDA23_00365 | CK945_RS00365 | ACH97_205920 |
| nucleoside triphosphate pyrophosphohydrolase | TRNA_RS21875 | BL14DL4_02668 | BLDA23_00370 | CK945_RS00370 | ACH97_205925 |
| RNA-binding S4 domain-containing protein | TRNA_RS21880 | BL14DL4_02669 | BLDA23_00375 | CK945_RS00375 | ACH97_205930 |
| sporulation protein YabP | TRNA_RS21885 | BL14DL4_02670 | BLDA23_00380 | CK945_RS00380 | ACH97_205935 |
| spore cortex biosynthesis protein YabQ | TRNA_RS21890 | BL14DL4_02671 | BLDA23_00385 | CK945_RS00385 | ACH97_205940 |
| septum formation initiator family protein | TRNA_RS21895 | BL14DL4_02672 | BLDA23_00390 | CK945_RS00390 | ACH97_205945 |
| RNA-binding protein S1 | TRNA_RS21900 | BL14DL4_02673 | BLDA23_00395 | CK945_RS00395 | ACH97_205950 |
| stage II sporulation protein E | TRNA_RS21915 | BL14DL4_02676 | BLDA23_00410 | CK945_RS00410 | ACH97_205965 |
| VWA domain-containing protein | TRNA_RS21920 | BL14DL4_02677 | BLDA23_00415 | CK945_RS00415 | ACH97_205970 |
| serine/threonine protein kinase | TRNA_RS21925 | BL14DL4_02678 | BLDA23_00420 | CK945_RS00420 | ACH97_205975 |
| tRNA(Ile)-lysidine synthetase | TRNA_RS21930 | BL14DL4_02679 | BLDA23_00425 | CK945_RS00425 | ACH97_205980 |
| hypoxanthine phosphoribosyltransferase | TRNA_RS21935 | BL14DL4_02680 | BLDA23_00430 | CK945_RS00430 | ACH97_205985 |
| ATP-dependent metallopeptidase FtsH/Yme1/Tma family protein | TRNA_RS21940 | BL14DL4_02681 | BLDA23_00435 | CK945_RS00435 | ACH97_205990 |
| type III pantothenate kinase | TRNA_RS21945 | BL14DL4_02682 | BLDA23_00440 | CK945_RS00440 | ACH97_205995 |
| Hsp33 family molecular chaperone HslO | TRNA_RS21950 | BL14DL4_02683 | BLDA23_00445 | CK945_RS00445 | ACH97_206000 |
| peptidyl-prolyl cis-trans isomerase | TRNA_RS21955 | BL14DL4_02684 | BLDA23_00450 | CK945_RS00450 | ACH97_206005 |
| cysteine synthase A | TRNA_RS21960 | BL14DL4_02685 | BLDA23_00455 | CK945_RS00455 | ACH97_206010 |
| anthranilate synthase component I family protein | TRNA_RS21970 | BL14DL4_02686 | BLDA23_00465 | CK945_RS00465 | ACH97_206015 |
| aminodeoxychorismate/anthranilate synthase component II | TRNA_RS21975 | BL14DL4_02687 | BLDA23_00470 | CK945_RS00470 | ACH97_206020 |
| 4-amino-4-deoxychorismate lyase | TRNA_RS21980 | BL14DL4_02688 | BLDA23_00475 | CK945_RS00475 | ACH97_206025 |
| dihydropteroate synthase | TRNA_RS21985 | BL14DL4_02689 | BLDA23_00480 | CK945_RS00480 | ACH97_206030 |
| dihydroneopterin aldolase | TRNA_RS21990 | BL14DL4_02690 | BLDA23_00485 | CK945_RS00485 | ACH97_206035 |
| 2-amino-4-hydroxy-6- hydroxymethyldihydropteridine diphosphokinase | TRNA_RS21995 | BL14DL4_02691 | BLDA23_00490 | CK945_RS00490 | ACH97_206040 |
| tRNA dihydrouridine synthase DusB | TRNA_RS22005 | BL14DL4_02693 | BLDA23_00500 | CK945_RS00500 | ACH97_206045 |
| lysine--tRNA ligase | TRNA_RS22010 | BL14DL4_02694 | BLDA23_00505 | CK945_RS00505 | ACH97_206050 |
| CtsR family transcriptional regulator | TRNA_RS22030 | BL14DL4_02710 | BLDA23_00585 | CK945_RS00585 | ACH97_220650 |
| protein arginine kinase | TRNA_RS22040 | BL14DL4_02712 | BLDA23_00595 | CK945_RS00595 | ACH97_220640 |
| ATP-dependent Clp protease ATP-binding subunit | TRNA_RS22045 | BL14DL4_02713 | BLDA23_00600 | CK945_RS00600 | ACH97_220635 |
| DNA repair protein RadA | TRNA_RS22050 | BL14DL4_02714 | BLDA23_00605 | CK945_RS00605 | ACH97_220630 |
| dna integrity scanning protein disa | TRNA_RS22055 | BL14DL4_02715 | BLDA23_00610 | CK945_RS00610 | ACH97_220625 |
| PIN/TRAM domain-containing protein | TRNA_RS22060 | BL14DL4_02716 | BLDA23_00615 | CK945_RS00615 | ACH97_220620 |
| 2-C-methyl-D-erythritol 4-phosphate cytidylyltransferase | TRNA_RS22065 | BL14DL4_02717 | BLDA23_00620 | CK945_RS00620 | ACH97_220615 |
| 2-C-methyl-D-erythritol 2,4-cyclodiphosphate synthase | TRNA_RS22070 | BL14DL4_02718 | BLDA23_00625 | CK945_RS00625 | ACH97_220610 |
| glutamate--tRNA ligase | TRNA_RS22075 | BL14DL4_02719 | BLDA23_00630 | CK945_RS00630 | ACH97_220605 |
| serine acetyltransferase | TRNA_RS22080 | BL14DL4_02720 | BLDA23_00635 | CK945_RS00635 | ACH97_220600 |
| cysteine--tRNA ligase | TRNA_RS22085 | BL14DL4_02721 | BLDA23_00640 | CK945_RS00640 | ACH97_220595 |
| ribonuclease III | TRNA_RS22090 | BL14DL4_02722 | BLDA23_00645 | CK945_RS00645 | ACH97_220590 |
| 23S rRNA (guanosine(2251)-2-O)-methyltransferase RlmB | TRNA_RS22095 | BL14DL4_02723 | BLDA23_00650 | CK945_RS00650 | ACH97_220585 |
| ribosome-dependent mRNA decay endonuclease Rae1/YacP | TRNA_RS22100 | BL14DL4_02724 | BLDA23_00655 | CK945_RS00655 | ACH97_220580 |
| RNA polymerase sporulation sigma factor SigH | TRNA_RS22105 | BL14DL4_02725 | BLDA23_00660 | CK945_RS00660 | ACH97_220575 |
| protein translocase subunit SecE | TRNA_RS22110 | BL14DL4_02726 | BLDA23_00670 | CK945_RS00670 | ACH97_220570 |
| transcription termination/antitermination protein NusG | TRNA_RS22115 | BL14DL4_02727 | BLDA23_00675 | CK945_RS00675 | ACH97_220565 |
| 50S ribosomal protein L11 | TRNA_RS22120 | BL14DL4_02728 | BLDA23_00680 | CK945_RS00680 | ACH97_220560 |
| 50S ribosomal protein L1 | TRNA_RS22125 | BL14DL4_02729 | BLDA23_00685 | CK945_RS00685 | ACH97_220555 |
| 50S ribosomal protein L10 | TRNA_RS22130 | BL14DL4_02730 | BLDA23_00690 | CK945_RS00690 | ACH97_220550 |
| 50S ribosomal protein L7/L12 | TRNA_RS22135 | BL14DL4_02731 | BLDA23_00695 | CK945_RS00695 | ACH97_220545 |
| DNA-directed RNA polymerase subunit beta | TRNA_RS22145 | BL14DL4_02733 | BLDA23_00705 | CK945_RS00705 | ACH97_220535 |
| DNA-directed RNA polymerase subunit beta | TRNA_RS22150 | BL14DL4_02734 | BLDA23_00710 | CK945_RS00710 | ACH97_220530 |
| 50S ribosomal protein L7ae-like protein | TRNA_RS22155 | BL14DL4_02735 | BLDA23_00715 | CK945_RS00715 | ACH97_220525 |
| 30S ribosomal protein S12 | TRNA_RS22160 | BL14DL4_02736 | BLDA23_00720 | CK945_RS00720 | ACH97_220520 |
| 30S ribosomal protein S7 | TRNA_RS22165 | BL14DL4_02737 | BLDA23_00725 | CK945_RS00725 | ACH97_220515 |
| elongation factor G | TRNA_RS22170 | BL14DL4_02738 | BLDA23_00730 | CK945_RS00730 | ACH97_220510 |
| elongation factor Tu | TRNA_RS22175 | BL14DL4_02739 | BLDA23_00735 | CK945_RS00735 | ACH97_220505 |
| 30S ribosomal protein S10 | TRNA_RS22180 | BL14DL4_02740 | BLDA23_00740 | CK945_RS00740 | ACH97_220500 |
| 50S ribosomal protein L3 | TRNA_RS22185 | BL14DL4_02741 | BLDA23_00745 | CK945_RS00745 | ACH97_220495 |
| 50S ribosomal protein L4 | TRNA_RS22190 | BL14DL4_02742 | BLDA23_00750 | CK945_RS00750 | ACH97_220490 |
| 50S ribosomal protein L23 | TRNA_RS22195 | BL14DL4_02743 | BLDA23_00755 | CK945_RS00755 | ACH97_220485 |
| 50S ribosomal protein L2 | TRNA_RS22200 | BL14DL4_02744 | BLDA23_00760 | CK945_RS00760 | ACH97_220480 |
| 30S ribosomal protein S19 | TRNA_RS22205 | BL14DL4_02745 | BLDA23_00765 | CK945_RS00765 | ACH97_220475 |
| 50S ribosomal protein L22 | TRNA_RS22210 | BL14DL4_02746 | BLDA23_00770 | CK945_RS00770 | ACH97_220470 |
| 30S ribosomal protein S3 | TRNA_RS22215 | BL14DL4_02747 | BLDA23_00775 | CK945_RS00775 | ACH97_220465 |
| 50S ribosomal protein L16 | TRNA_RS22220 | BL14DL4_02748 | BLDA23_00780 | CK945_RS00780 | ACH97_220460 |
| 50S ribosomal protein L29 | TRNA_RS22225 | BL14DL4_02749 | BLDA23_00785 | CK945_RS00785 | ACH97_220455 |
| 30S ribosomal protein S17 | TRNA_RS22230 | BL14DL4_02750 | BLDA23_00790 | CK945_RS00790 | ACH97_220450 |
| 50S ribosomal protein L14 | TRNA_RS22235 | BL14DL4_02751 | BLDA23_00795 | CK945_RS00795 | ACH97_220445 |
| 50S ribosomal protein L24 | TRNA_RS22240 | BL14DL4_02752 | BLDA23_00800 | CK945_RS00800 | ACH97_220440 |
| 50S ribosomal protein L5 | TRNA_RS22245 | BL14DL4_02753 | BLDA23_00805 | CK945_RS00805 | ACH97_220435 |
| 30S ribosomal protein S8 | TRNA_RS22250 | BL14DL4_02755 | BLDA23_00815 | CK945_RS00815 | ACH97_220430 |
| 50S ribosomal protein L6 | TRNA_RS22255 | BL14DL4_02756 | BLDA23_00820 | CK945_RS00820 | ACH97_220425 |
| 50S ribosomal protein L18 | TRNA_RS22260 | BL14DL4_02757 | BLDA23_00825 | CK945_RS00825 | ACH97_220420 |
| 30S ribosomal protein S5 | TRNA_RS22265 | BL14DL4_02758 | BLDA23_00830 | CK945_RS00830 | ACH97_220415 |
| 50S ribosomal protein L30 | TRNA_RS22270 | BL14DL4_02759 | BLDA23_00835 | CK945_RS00835 | ACH97_220410 |
| 50S ribosomal protein L15 | TRNA_RS22275 | BL14DL4_02760 | BLDA23_00840 | CK945_RS00840 | ACH97_220405 |
| protein translocase subunit SecY | TRNA_RS22280 | BL14DL4_02761 | BLDA23_00845 | CK945_RS00845 | ACH97_220400 |
| adenylate kinase | TRNA_RS22285 | BL14DL4_02762 | BLDA23_00850 | CK945_RS00850 | ACH97_220395 |
| type I methionyl aminopeptidase | TRNA_RS22290 | BL14DL4_02763 | BLDA23_00855 | CK945_RS00855 | ACH97_220390 |
| RNA-binding protein | TRNA_RS22295 | BL14DL4_02764 | BLDA23_00860 | CK945_RS00860 | ACH97_220385 |
| translation initiation factor IF-1 | TRNA_RS22300 | BL14DL4_02765 | BLDA23_00865 | CK945_RS00865 | ACH97_220380 |
| 50S ribosomal protein L36 | TRNA_RS22305 | BL14DL4_02766 | BLDA23_00870 | CK945_RS00870 | ACH97_220375 |
| 30S ribosomal protein S13 | TRNA_RS22310 | BL14DL4_02767 | BLDA23_00875 | CK945_RS00875 | ACH97_220370 |
| 30S ribosomal protein S11 | TRNA_RS22315 | BL14DL4_02768 | BLDA23_00880 | CK945_RS00880 | ACH97_220365 |
| DNA-directed RNA polymerase subunit alpha | TRNA_RS22320 | BL14DL4_02769 | BLDA23_00885 | CK945_RS00885 | ACH97_220360 |
| 50S ribosomal protein L17 | TRNA_RS22325 | BL14DL4_02770 | BLDA23_00890 | CK945_RS00890 | ACH97_220355 |
| energy-coupling factor transporter ATPase | TRNA_RS22330 | BL14DL4_02771 | BLDA23_00895 | CK945_RS00895 | ACH97_220350 |
| energy-coupling factor transporter ATPase | TRNA_RS22335 | BL14DL4_02772 | BLDA23_00900 | CK945_RS00900 | ACH97_220345 |
| energy-coupling factor transporter transmembrane protein EcfT | TRNA_RS22340 | BL14DL4_02773 | BLDA23_00905 | CK945_RS00905 | ACH97_220340 |
| tRNA pseudouridine(38-40) synthase TruA | TRNA_RS22345 | BL14DL4_02774 | BLDA23_00910 | CK945_RS00910 | ACH97_220335 |
| 50S ribosomal protein L13 | TRNA_RS22350 | BL14DL4_02775 | BLDA23_00915 | CK945_RS00915 | ACH97_220330 |
| 30S ribosomal protein S9 | TRNA_RS22355 | BL14DL4_02776 | BLDA23_00920 | CK945_RS00920 | ACH97_220325 |
| DNA damage-inducible protein DinB | TRNA_RS22360 | BL14DL4_02777 | BLDA23_00925 | CK945_RS00945 | ACH97_220305 |
| DUF2521 family protein | TRNA_RS22365 | BL14DL4_02778 | BLDA23_00930 | CK945_RS01045 | ACH97_220215 |
| N-acetylmuramoyl-L-alanine amidase CwlD | TRNA_RS22370 | BL14DL4_02779 | BLDA23_00935 | CK945_RS01050 | ACH97_220210 |
| chromosome partitioning protein ParA | TRNA_RS22375 | BL14DL4_02780 | BLDA23_00940 | CK945_RS01055 | ACH97_220205 |
| hypothetical protein | TRNA_RS22380 | BL14DL4_02781 | BLDA23_00945 | CK945_RS01060 | ACH97_220200 |
| kinB signaling pathway activation protein KbaA | TRNA_RS22385 | BL14DL4_02782 | BLDA23_00950 | CK945_RS01065 | ACH97_220195 |
| polysaccharide deacetylase family sporulation protein PdaB | TRNA_RS22390 | BL14DL4_02783 | BLDA23_00955 | CK945_RS01070 | ACH97_220190 |
| penicillin-binding protein X | TRNA_RS22420 | BL14DL4_02790 | BLDA23_00985 | CK945_RS01100 | ACH97_221725 |
| hypothetical protein | TRNA_RS22425 | BL14DL4_02791 | BLDA23_00990 | CK945_RS01105 | ACH97_221730 |
| bile acid:sodium symporter family protein | TRNA_RS22430 | BL14DL4_02792 | BLDA23_00995 | CK945_RS01110 | ACH97_221735 |
| hypothetical protein | TRNA_RS22435 | BL14DL4_02793 | BLDA23_01000 | CK945_RS01115 | ACH97_221740 |
| DUF1343 domain-containing protein | TRNA_RS22445 | BL14DL4_02795 | BLDA23_01010 | CK945_RS01125 | ACH97_221750 |
| hypothetical protein | TRNA_RS22450 | BL14DL4_02796 | BLDA23_01015 | CK945_RS01130 | ACH97_221755 |
| penicillin binding protein PBP4B | TRNA_RS22455 | BL14DL4_02797 | BLDA23_01020 | CK945_RS01135 | ACH97_221760 |
| permease | TRNA_RS22460 | BL14DL4_02798 | BLDA23_01025 | CK945_RS01140 | ACH97_221765 |
| MurR/RpiR family transcriptional regulator | TRNA_RS22465 | BL14DL4_02799 | BLDA23_01030 | CK945_RS01145 | ACH97_221770 |
| N-acetylmuramic acid 6-phosphate etherase | TRNA_RS22470 | BL14DL4_02800 | BLDA23_01035 | CK945_RS01150 | ACH97_221775 |
| DUF523 domain-containing protein | TRNA_RS22475 | BL14DL4_02801 | BLDA23_01040 | CK945_RS01155 | ACH97_221780 |
| arginase | TRNA_RS22505 | BL14DL4_02807 | BLDA23_01070 | CK945_RS01185 | ACH97_221810 |
| RNA polymerase sigma factor SigW | TRNA_RS22510 | BL14DL4_02808 | BLDA23_01075 | CK945_RS01190 | ACH97_221815 |
| anti-sigma factor | TRNA_RS22515 | BL14DL4_02809 | BLDA23_01080 | CK945_RS01195 | ACH97_221820 |
| TIGR00159 family protein | TRNA_RS22520 | BL14DL4_02810 | BLDA23_01085 | CK945_RS01200 | ACH97_221825 |
| YbbR-like domain-containing protein | TRNA_RS22525 | BL14DL4_02811 | BLDA23_01090 | CK945_RS01205 | ACH97_221830 |
| phosphoglucosamine mutase | TRNA_RS22530 | BL14DL4_02812 | BLDA23_01095 | CK945_RS01210 | ACH97_221835 |
| glutamine--fructose-6-phosphate aminotransferase | TRNA_RS22535 | BL14DL4_02813 | BLDA23_01100 | CK945_RS01215 | ACH97_221840 |
| hypothetical protein | TRNA_RS22540 | BL14DL4_02814 | BLDA23_01105 | CK945_RS01220 | ACH97_221845 |
| ABC-2 transporter permease | TRNA_RS22545 | BL14DL4_02815 | BLDA23_01110 | CK945_RS01225 | ACH97_221850 |
| ABC transporter ATP-binding protein | TRNA_RS22550 | BL14DL4_02816 | BLDA23_01115 | CK945_RS01230 | ACH97_221855 |
| GntR family transcriptional regulator | TRNA_RS22555 | BL14DL4_02817 | BLDA23_01120 | CK945_RS01235 | ACH97_221860 |
| ArsR family transcriptional regulator | TRNA_RS22560 | BL14DL4_02818 | BLDA23_01125 | CK945_RS01255 | ACH97_221865 |
| MFS transporter | TRNA_RS22565 | BL14DL4_02819 | BLDA23_01130 | CK945_RS01260 | ACH97_221870 |
| DinB family protein | TRNA_RS22570 | BL14DL4_02820 | BLDA23_01135 | CK945_RS01265 | ACH97_221885 |
| ABC transporter ATP-binding protein | TRNA_RS22575 | BL14DL4_02821 | BLDA23_01140 | CK945_RS01305 | ACH97_221890 |
| DsbA family oxidoreductase | TRNA_RS22580 | BL14DL4_02822 | BLDA23_01145 | CK945_RS01310 | ACH97_221895 |
| hypothetical protein | TRNA_RS22585 | BL14DL4_02823 | BLDA23_01150 | CK945_RS01315 | ACH97_221900 |
| aminoglycoside 6-adenylyltransferase | TRNA_RS22595 | BL14DL4_02825 | BLDA23_01160 | CK945_RS01335 | ACH97_221915 |
| aminoglycoside phosphotransferase family protein | TRNA_RS22600 | BL14DL4_02826 | BLDA23_01165 | CK945_RS01340 | ACH97_221920 |
| , YjjG familynoncanonical pyrimidine nucleotidase | TRNA_RS22605 | BL14DL4_02827 | BLDA23_01170 | CK945_RS01345 | ACH97_221925 |
| hypothetical protein | TRNA_RS22650 | BL14DL4_02836 | BLDA23_01215 | CK945_RS01385 | ACH97_221950 |
| hypothetical protein | TRNA_RS22655 | BL14DL4_02837 | BLDA23_01220 | CK945_RS01390 | ACH97_221955 |
| hypothetical protein | TRNA_RS22660 | BL14DL4_02838 | BLDA23_01225 | CK945_RS01395 | ACH97_221960 |
| thioredoxin domain-containing protein | TRNA_RS22665 | BL14DL4_02839 | BLDA23_01230 | CK945_RS01400 | ACH97_221965 |
| MFS transporter | TRNA_RS22705 | BL14DL4_02845 | BLDA23_01250 | CK945_RS03200 | ACH97_216430 |
| class I SAM-dependent methyltransferase | TRNA_RS22710 | BL14DL4_02846 | BLDA23_01255 | CK945_RS01475 | ACH97_215680 |
| SDR family oxidoreductase | TRNA_RS22715 | BL14DL4_02847 | BLDA23_01260 | CK945_RS01500 | ACH97_215655 |
| DUF1648 domain-containing protein | TRNA_RS22720 | BL14DL4_02848 | BLDA23_01265 | CK945_RS01510 | ACH97_215645 |
| membrane protein | TRNA_RS22730 | BL14DL4_02850 | BLDA23_01275 | CK945_RS01520 | ACH97_215635 |
| hypothetical protein | TRNA_RS22740 | BL14DL4_02852 | BLDA23_01285 | CK945_RS01530 | ACH97_215625 |
| hypothetical protein | TRNA_RS22745 | BL14DL4_02853 | BLDA23_01290 | CK945_RS01535 | ACH97_215620 |
| DUF2000 domain-containing protein | TRNA_RS22750 | BL14DL4_02854 | BLDA23_01295 | CK945_RS01540 | ACH97_215615 |
| DMT family transporter | TRNA_RS22755 | BL14DL4_02855 | BLDA23_01300 | CK945_RS01545 | ACH97_215610 |
| helix-turn-helix domain-containing protein | TRNA_RS22770 | BL14DL4_02858 | BLDA23_01315 | CK945_RS01555 | ACH97_215600 |
| LuxR family transcriptional regulator | TRNA_RS22775 | BL14DL4_02859 | BLDA23_01320 | CK945_RS01560 | ACH97_215595 |
| iron-containing alcohol dehydrogenase family protein | TRNA_RS22780 | BL14DL4_02860 | BLDA23_01325 | CK945_RS01575 | ACH97_215580 |
| hypothetical protein | TRNA_RS22795 | BL14DL4_02863 | BLDA23_01340 | CK945_RS01580 | ACH97_215575 |
| hypothetical protein | TRNA_RS22800 | BL14DL4_02864 | BLDA23_01345 | CK945_RS01585 | ACH97_215570 |
| virginiamycin B lyase | TRNA_RS22805 | BL14DL4_02865 | BLDA23_01350 | CK945_RS01590 | ACH97_215565 |
| DUF2512 family protein | TRNA_RS22810 | BL14DL4_02866 | BLDA23_01355 | CK945_RS01595 | ACH97_215560 |
| S-methylmethionine permease | TRNA_RS22815 | BL14DL4_02867 | BLDA23_01360 | CK945_RS01600 | ACH97_215555 |
| homocysteine S-methyltransferase | TRNA_RS22820 | BL14DL4_02868 | BLDA23_01365 | CK945_RS01605 | ACH97_215550 |
| chitin disaccharide deacetylase | TRNA_RS22825 | BL14DL4_02869 | BLDA23_01370 | CK945_RS01610 | ACH97_215545 |
| PTS acetylglucosamine transporter subunit IIB | TRNA_RS22830 | BL14DL4_02870 | BLDA23_01375 | CK945_RS01615 | ACH97_215540 |
| MerR family transcriptional regulator | TRNA_RS22835 | BL14DL4_02871 | BLDA23_01380 | CK945_RS01620 | ACH97_215535 |
| DUF421 domain-containing protein | TRNA_RS22840 | BL14DL4_02872 | BLDA23_01385 | CK945_RS01625 | ACH97_215530 |
| carbonic anhydrase | TRNA_RS22845 | BL14DL4_02873 | BLDA23_01390 | CK945_RS01630 | ACH97_215525 |
| DUF2809 domain-containing protein | TRNA_RS22855 | BL14DL4_02874 | BLDA23_01400 | CK945_RS01635 | ACH97_215515 |
| alanine:cation symporter family protein | TRNA_RS22860 | BL14DL4_02875 | BLDA23_01405 | CK945_RS01640 | ACH97_215510 |
| glutaminase | TRNA_RS22865 | BL14DL4_02876 | BLDA23_01410 | CK945_RS01645 | ACH97_215505 |
| sensor histidine kinase | TRNA_RS22870 | BL14DL4_02877 | BLDA23_01415 | CK945_RS01650 | ACH97_215500 |
| response regulator | TRNA_RS22875 | BL14DL4_02878 | BLDA23_01420 | CK945_RS01655 | ACH97_215495 |
| N-acetyltransferase | TRNA_RS22880 | BL14DL4_02879 | BLDA23_01425 | CK945_RS01660 | ACH97_215490 |
| BlaR1 family beta-lactam sensor/signal transducer | TRNA_RS22885 | BL14DL4_02880 | BLDA23_01430 | CK945_RS01665 | ACH97_215485 |
| BlaI/MecI/CopY family transcriptional regulator | TRNA_RS22890 | BL14DL4_02881 | BLDA23_01435 | CK945_RS01670 | ACH97_215480 |
| alkaline phosphatase | TRNA_RS22900 | BL14DL4_02883 | BLDA23_01445 | CK945_RS01680 | ACH97_215470 |
| twin-arginine translocase TatA/TatE family subunit | TRNA_RS22905 | BL14DL4_02884 | BLDA23_01450 | CK945_RS01685 | ACH97_215465 |
| twin-arginine translocase subunit TatC | TRNA_RS22910 | BL14DL4_02885 | BLDA23_01455 | CK945_RS01690 | ACH97_215460 |
| 5-dehydro-4-deoxyglucarate dehydratase | TRNA_RS22915 | BL14DL4_02886 | BLDA23_01460 | CK945_RS01695 | ACH97_215455 |
| aldehyde dehydrogenase family protein | TRNA_RS22920 | BL14DL4_02887 | BLDA23_01465 | CK945_RS01700 | ACH97_215450 |
| MFS transporter | TRNA_RS22925 | BL14DL4_02888 | BLDA23_01470 | CK945_RS01705 | ACH97_215445 |
| glucarate dehydratase | TRNA_RS22930 | BL14DL4_02889 | BLDA23_01475 | CK945_RS01710 | ACH97_215440 |
| FadR family transcriptional regulator | TRNA_RS22935 | BL14DL4_02890 | BLDA23_01480 | CK945_RS01715 | ACH97_215435 |
| galactarate dehydratase | TRNA_RS22940 | BL14DL4_02891 | BLDA23_01485 | CK945_RS01720 | ACH97_215430 |
| sensor histidine kinase | TRNA_RS22950 | BL14DL4_02894 | BLDA23_01495 | CK945_RS01745 | ACH97_215405 |
| ABC transporter ATP-binding protein | TRNA_RS22955 | BL14DL4_02895 | BLDA23_01500 | CK945_RS01750 | ACH97_215400 |
| hypothetical protein | TRNA_RS22960 | BL14DL4_02896 | BLDA23_01505 | CK945_RS01755 | ACH97_215395 |
| DUF3900 domain-containing protein | TRNA_RS22970 | BL14DL4_02898 | BLDA23_01515 | CK945_RS01765 | ACH97_215385 |
| DUF4885 domain-containing protein | TRNA_RS22975 | BL14DL4_02899 | BLDA23_01520 | CK945_RS01770 | ACH97_215380 |
| hypothetical protein | TRNA_RS22980 | BL14DL4_02900 | BLDA23_01525 | CK945_RS01775 | ACH97_215375 |
| MarR family transcriptional regulator | TRNA_RS22985 | BL14DL4_02901 | BLDA23_01530 | CK945_RS01780 | ACH97_215370 |
| acyl--CoA ligase | TRNA_RS22990 | BL14DL4_02902 | BLDA23_01535 | CK945_RS01785 | ACH97_215365 |
| proteinase inhibitor | TRNA_RS22995 | BL14DL4_02903 | BLDA23_01540 | CK945_RS01790 | ACH97_215360 |
| anti protein | TRNA_RS23000 | BL14DL4_02904 | BLDA23_01545 | CK945_RS01795 | ACH97_215355 |
| DUF4885 domain-containing protein | TRNA_RS23005 | BL14DL4_02905 | BLDA23_01550 | CK945_RS01800 | ACH97_215350 |
| MerR family transcriptional regulator | TRNA_RS23010 | BL14DL4_02906 | BLDA23_01555 | CK945_RS01805 | ACH97_215345 |
| PadR family transcriptional regulator | TRNA_RS23020 | BL14DL4_02908 | BLDA23_01565 | CK945_RS01810 | ACH97_215340 |
| DUF2812 domain-containing protein | TRNA_RS23025 | BL14DL4_02909 | BLDA23_01570 | CK945_RS01815 | ACH97_215335 |
| tryptophan RNA-binding attenuator protein inhibitory protein | TRNA_RS23030 | BL14DL4_02910 | BLDA23_01575 | CK945_RS01820 | ACH97_215330 |
| DMT family transporter | TRNA_RS23035 | BL14DL4_02911 | BLDA23_01580 | CK945_RS01825 | ACH97_215325 |
| hypothetical protein | TRNA_RS23045 | BL14DL4_02913 | BLDA23_01590 | CK945_RS01835 | ACH97_215320 |
| lantibiotic protection ABC transporter ATP-binding subunit | TRNA_RS23050 | BL14DL4_02914 | BLDA23_01595 | CK945_RS01840 | ACH97_215315 |
| lantibiotic immunity ABC transporter MutE/EpiE family permease subunit | TRNA_RS23055 | BL14DL4_02915 | BLDA23_01600 | CK945_RS01845 | ACH97_215310 |
| lantibiotic immunity ABC transporter MutG family permease subunit | TRNA_RS23060 | BL14DL4_02916 | BLDA23_01605 | CK945_RS01850 | ACH97_215305 |
| DNA-binding response regulator | TRNA_RS23065 | BL14DL4_02917 | BLDA23_01610 | CK945_RS01855 | ACH97_215300 |
| sensor histidine kinase | TRNA_RS23070 | BL14DL4_02918 | BLDA23_01615 | CK945_RS01860 | ACH97_215295 |
| LysR family transcriptional regulator | TRNA_RS23075 | BL14DL4_02919 | BLDA23_01620 | CK945_RS01865 | ACH97_215290 |
| tautomerase family protein | TRNA_RS23080 | BL14DL4_02920 | BLDA23_01625 | CK945_RS01870 | ACH97_215285 |
| class I SAM-dependent methyltransferase | TRNA_RS23090 | BL14DL4_02922 | BLDA23_01635 | CK945_RS01880 | ACH97_215275 |
| DUF4352 domain-containing protein | TRNA_RS23095 | BL14DL4_02923 | BLDA23_01640 | CK945_RS01885 | ACH97_215270 |
| putative basic amino acid antiporter YfcC | TRNA_RS23100 | BL14DL4_02924 | BLDA23_01645 | CK945_RS01890 | ACH97_215265 |
| M20 family peptidase | TRNA_RS23105 | BL14DL4_02925 | BLDA23_01650 | CK945_RS01895 | ACH97_215260 |
| GNAT family N-acetyltransferase | TRNA_RS23110 | BL14DL4_02926 | BLDA23_01655 | CK945_RS01905 | ACH97_215250 |
| amidase | TRNA_RS23115 | BL14DL4_02927 | BLDA23_01660 | CK945_RS01910 | ACH97_215245 |
| GlsB/YeaQ/YmgE family stress response membrane protein | TRNA_RS23125 | BL14DL4_02929 | BLDA23_01670 | CK945_RS01920 | ACH97_215235 |
| DUF3471 domain-containing protein | TRNA_RS23130 | BL14DL4_02930 | BLDA23_01675 | CK945_RS01925 | ACH97_215230 |
| (Fe-S)-binding protein | TRNA_RS23135 | BL14DL4_02931 | BLDA23_01680 | CK945_RS01930 | ACH97_215225 |
| glycolate oxidase subunit GlcD | TRNA_RS23140 | BL14DL4_02932 | BLDA23_01685 | CK945_RS01935 | ACH97_215220 |
| PTS sugar transporter subunit IIB | TRNA_RS23145 | BL14DL4_02933 | BLDA23_01690 | CK945_RS01940 | ACH97_215215 |
| PTS lactose/cellobiose transporter subunit IIA | TRNA_RS23150 | BL14DL4_02934 | BLDA23_01695 | CK945_RS01945 | ACH97_215210 |
| PTS cellobiose transporter subunit IIC | TRNA_RS23155 | BL14DL4_02935 | BLDA23_01700 | CK945_RS01950 | ACH97_215205 |
| 6-phospho-beta-glucosidase | TRNA_RS23160 | BL14DL4_02936 | BLDA23_01705 | CK945_RS01955 | ACH97_215200 |
| GntR family transcriptional regulator | TRNA_RS23165 | BL14DL4_02937 | BLDA23_01710 | CK945_RS01960 | ACH97_215195 |
| chitin disaccharide deacetylase | TRNA_RS23170 | BL14DL4_02938 | BLDA23_01715 | CK945_RS01965 | ACH97_215190 |
| chitinase | TRNA_RS23175 | BL14DL4_02939 | BLDA23_01720 | CK945_RS01970 | ACH97_215185 |
| serine protease | TRNA_RS23185 | BL14DL4_02941 | BLDA23_01730 | CK945_RS01980 | ACH97_215175 |
| SDR family oxidoreductase | TRNA_RS23190 | BL14DL4_02942 | BLDA23_01735 | CK945_RS01985 | ACH97_215170 |
| DUF1835 domain-containing protein | TRNA_RS23195 | BL14DL4_02943 | BLDA23_01740 | CK945_RS01990 | ACH97_215165 |
| DUF4901 domain-containing protein | TRNA_RS23200 | BL14DL4_02944 | BLDA23_01745 | CK945_RS01995 | ACH97_215160 |
| cell wall hydrolase | TRNA_RS23210 | BL14DL4_02946 | BLDA23_01755 | CK945_RS02005 | ACH97_215150 |
| LLM class flavin-dependent oxidoreductase | TRNA_RS23215 | BL14DL4_02947 | BLDA23_01760 | CK945_RS02010 | ACH97_215145 |
| peptidoglycan DL-endopeptidase CwlO | TRNA_RS23220 | BL14DL4_02948 | BLDA23_01765 | CK945_RS02015 | ACH97_215140 |
| permease | TRNA_RS23225 | BL14DL4_02949 | BLDA23_01770 | CK945_RS02020 | ACH97_215135 |
| TetR/AcrR family transcriptional regulator | TRNA_RS23235 | BL14DL4_02951 | BLDA23_01780 | CK945_RS02030 | ACH97_215125 |
| DUF2207 domain-containing protein | TRNA_RS23240 | BL14DL4_02952 | BLDA23_01785 | CK945_RS02035 | ACH97_215120 |
| TerD family protein | TRNA_RS23255 | BL14DL4_02955 | BLDA23_01800 | CK945_RS02050 | ACH97_215115 |
| TerD family protein | TRNA_RS23260 | BL14DL4_02956 | BLDA23_01805 | CK945_RS02055 | ACH97_215110 |
| TerD family protein | TRNA_RS23265 | BL14DL4_02957 | BLDA23_01810 | CK945_RS02060 | ACH97_215105 |
| DUF475 domain-containing protein | TRNA_RS23270 | BL14DL4_02958 | BLDA23_01815 | CK945_RS02065 | ACH97_215100 |
| ATP/GTP-binding protein | TRNA_RS23275 | BL14DL4_02959 | BLDA23_01820 | CK945_RS02070 | ACH97_215095 |
| hypothetical protein | TRNA_RS23280 | BL14DL4_02960 | BLDA23_01825 | CK945_RS02075 | ACH97_215090 |
| hypothetical protein | TRNA_RS23285 | BL14DL4_02961 | BLDA23_01830 | CK945_RS02080 | ACH97_215085 |
| toxic anion resistance protein | TRNA_RS23290 | BL14DL4_02962 | BLDA23_01835 | CK945_RS02085 | ACH97_215080 |
| cytochrome C biogenesis protein CcdA | TRNA_RS23295 | BL14DL4_02963 | BLDA23_01840 | CK945_RS02090 | ACH97_215075 |
| L-lactate dehydrogenase | TRNA_RS23315 | BL14DL4_02967 | BLDA23_01860 | CK945_RS02105 | ACH97_215055 |
| L-lactate permease | TRNA_RS23320 | BL14DL4_02968 | BLDA23_01865 | CK945_RS02110 | ACH97_215050 |
| amino acid transporter | TRNA_RS23325 | BL14DL4_02969 | BLDA23_01870 | CK945_RS02115 | ACH97_215045 |
| NAD(+) synthetase | TRNA_RS23335 | BL14DL4_02971 | BLDA23_01880 | CK945_RS02125 | ACH97_215035 |
| shikimate kinase | TRNA_RS23340 | BL14DL4_02972 | BLDA23_01885 | CK945_RS02130 | ACH97_215030 |
| nucleotidyltransferase domain-containing protein | TRNA_RS23345 | BL14DL4_02973 | BLDA23_01890 | CK945_RS02135 | ACH97_215025 |
| proline dehydrogenase | TRNA_RS23350 | BL14DL4_02974 | BLDA23_01895 | CK945_RS02145 | ACH97_215015 |
| L-glutamate gamma-semialdehyde dehydrogenase | TRNA_RS23355 | BL14DL4_02975 | BLDA23_01900 | CK945_RS02150 | ACH97_215010 |
| sodium/proline symporter PutP | TRNA_RS23360 | BL14DL4_02976 | BLDA23_01905 | CK945_RS02155 | ACH97_215005 |
| PucR family transcriptional regulator | TRNA_RS23365 | BL14DL4_02977 | BLDA23_01910 | CK945_RS02160 | ACH97_215000 |
| TIGR03943 family protein | TRNA_RS23370 | BL14DL4_02978 | BLDA23_01915 | CK945_RS02165 | ACH97_214995 |
| permease | TRNA_RS23375 | BL14DL4_02979 | BLDA23_01920 | CK945_RS02170 | ACH97_214990 |
| cephalosporin deacetylase | TRNA_RS23380 | BL14DL4_02980 | BLDA23_01925 | CK945_RS02175 | ACH97_214985 |
| MarR family transcriptional regulator | TRNA_RS23385 | BL14DL4_02981 | BLDA23_01930 | CK945_RS02180 | ACH97_214980 |
| DUF3147 family protein | TRNA_RS23390 | BL14DL4_02982 | BLDA23_01935 | CK945_RS02185 | ACH97_214975 |
| hypothetical protein | TRNA_RS23395 | BL14DL4_02983 | BLDA23_01940 | CK945_RS02190 | ACH97_214970 |
| amino acid ABC transporter permease | TRNA_RS23400 | BL14DL4_02984 | BLDA23_01945 | CK945_RS02195 | ACH97_214965 |
| ABC transporter substrate-binding protein | TRNA_RS23405 | BL14DL4_02985 | BLDA23_01950 | CK945_RS02200 | ACH97_214960 |
| flavodoxin family protein | TRNA_RS23410 | BL14DL4_02986 | BLDA23_01955 | CK945_RS02205 | ACH97_214955 |
| ATP-binding cassette domain-containing protein | TRNA_RS23415 | BL14DL4_02987 | BLDA23_01960 | CK945_RS02210 | ACH97_214950 |
| aryl-phospho-beta-D-glucosidase | TRNA_RS23420 | BL14DL4_02988 | BLDA23_01965 | CK945_RS02215 | ACH97_214945 |
| peptidase S24 | TRNA_RS23425 | BL14DL4_02989 | BLDA23_01970 | CK945_RS02220 | ACH97_214940 |
| protein kinase | TRNA_RS23435 | BL14DL4_02991 | BLDA23_01980 | CK945_RS02230 | ACH97_214930 |
| transposase | TRNA_RS23445 | BL14DL4_02994 | BLDA23_01990 | CK945_RS02235 | ACH97_214925 |
| hydantoinase/oxoprolinase family protein | TRNA_RS23450 | BL14DL4_02995 | BLDA23_01995 | CK945_RS02240 | ACH97_214920 |
| hydantoinase B/oxoprolinase family protein | TRNA_RS23455 | BL14DL4_02996 | BLDA23_02000 | CK945_RS02245 | ACH97_214915 |
| ABC transporter substrate-binding protein | TRNA_RS23460 | BL14DL4_02997 | BLDA23_02005 | CK945_RS02250 | ACH97_214910 |
| ABC transporter ATP-binding protein | TRNA_RS23465 | BL14DL4_02998 | BLDA23_02010 | CK945_RS02255 | ACH97_214905 |
| ABC transporter permease | TRNA_RS23470 | BL14DL4_02999 | BLDA23_02015 | CK945_RS02260 | ACH97_214900 |
| ABC transporter permease | TRNA_RS23475 | BL14DL4_03000 | BLDA23_02020 | CK945_RS02265 | ACH97_214895 |
| lichenysin non-ribosomal peptide synthetase LicA | TRNA_RS23485 | BL14DL4_03001 | BLDA23_02025 | CK945_RS02270 | ACH97_214885 |
| lichenysin non-ribosomal peptide synthetase LicB | TRNA_RS23490 | BL14DL4_03002 | BLDA23_02030 | CK945_RS02275 | ACH97_214880 |
| lichenysin non-ribosomal peptide synthetase LicC | TRNA_RS23495 | BL14DL4_03003 | BLDA23_02035 | CK945_RS02280 | ACH97_214875 |
| lichenysin biosynthesis thioesterase LicTE | TRNA_RS23500 | BL14DL4_03004 | BLDA23_02040 | CK945_RS02285 | ACH97_214870 |
| PLP-dependent aminotransferase family protein | TRNA_RS23510 | BL14DL4_03006 | BLDA23_02050 | CK945_RS02295 | ACH97_214860 |
| 4-phosphopantetheinyl transferase | TRNA_RS23515 | BL14DL4_03007 | BLDA23_02055 | CK945_RS02300 | ACH97_214855 |
| alpha/beta hydrolase | TRNA_RS23520 | BL14DL4_03008 | BLDA23_02060 | CK945_RS02305 | ACH97_214850 |
| YitT family protein | TRNA_RS23530 | BL14DL4_03010 | BLDA23_02070 | CK945_RS02315 | ACH97_214840 |
| aspartate aminotransferase family protein | TRNA_RS23555 | BL14DL4_03015 | BLDA23_02095 | CK945_RS02335 | ACH97_214825 |
| methyl-accepting chemotaxis protein | TRNA_RS23560 | BL14DL4_03016 | BLDA23_02100 | CK945_RS02340 | ACH97_214820 |
| amino acid ABC transporter ATP-binding protein | TRNA_RS23565 | BL14DL4_03017 | BLDA23_02105 | CK945_RS02355 | ACH97_214815 |
| amino acid ABC transporter permease | TRNA_RS23570 | BL14DL4_03018 | BLDA23_02110 | CK945_RS02360 | ACH97_214810 |
| amino acid ABC transporter substrate-binding protein | TRNA_RS23575 | BL14DL4_03019 | BLDA23_02115 | CK945_RS02365 | ACH97_214805 |
| sigma-54-dependent Fis family transcriptional regulator | TRNA_RS23580 | BL14DL4_03020 | BLDA23_02120 | CK945_RS02370 | ACH97_214800 |
| ornithine--oxo-acid transaminase | TRNA_RS23585 | BL14DL4_03021 | BLDA23_02125 | CK945_RS02375 | ACH97_214795 |
| amino acid permease | TRNA_RS23590 | BL14DL4_03022 | BLDA23_02130 | CK945_RS02380 | ACH97_214790 |
| arginase | TRNA_RS23595 | BL14DL4_03023 | BLDA23_02135 | CK945_RS02385 | ACH97_214785 |
| UbiX family flavin prenyltransferase | TRNA_RS23605 | BL14DL4_03025 | BLDA23_02145 | CK945_RS02395 | ACH97_214775 |
| UbiD family decarboxylase | TRNA_RS23610 | BL14DL4_03026 | BLDA23_02150 | CK945_RS02400 | ACH97_214770 |
| hypothetical protein | TRNA_RS23615 | BL14DL4_03027 | BLDA23_02155 | CK945_RS02405 | ACH97_214765 |
| hypothetical protein | TRNA_RS23620 | BL14DL4_03028 | BLDA23_02160 | CK945_RS02410 | ACH97_214760 |
| hypothetical protein | TRNA_RS23625 | BL14DL4_03029 | BLDA23_02165 | CK945_RS02415 | ACH97_214755 |
| hypothetical protein | TRNA_RS23630 | BL14DL4_03030 | BLDA23_02170 | CK945_RS02420 | ACH97_214750 |
| hypothetical protein | TRNA_RS23635 | BL14DL4_03031 | BLDA23_02175 | CK945_RS02425 | ACH97_214745 |
| hypothetical protein | TRNA_RS23640 | BL14DL4_03032 | BLDA23_02180 | CK945_RS02430 | ACH97_214740 |
| hypothetical protein | TRNA_RS23645 | BL14DL4_03033 | BLDA23_02185 | CK945_RS02435 | ACH97_214735 |
| hypothetical protein | TRNA_RS23665 | BL14DL4_03037 | BLDA23_02205 | CK945_RS02455 | ACH97_214695 |
| DUF5082 domain-containing protein | TRNA_RS23670 | BL14DL4_03038 | BLDA23_02210 | CK945_RS02460 | ACH97_214690 |
| sensor histidine kinase | TRNA_RS23680 | BL14DL4_03040 | BLDA23_02220 | CK945_RS02470 | ACH97_214680 |
| sugar ABC transporter substrate-binding protein | TRNA_RS23690 | BL14DL4_03042 | BLDA23_02230 | CK945_RS02480 | ACH97_214670 |
| ATP-binding cassette domain-containing protein | TRNA_RS23695 | BL14DL4_03043 | BLDA23_02235 | CK945_RS02485 | ACH97_214665 |
| sugar ABC transporter permease | TRNA_RS23700 | BL14DL4_03044 | BLDA23_02240 | CK945_RS02490 | ACH97_214660 |
| MFS transporter | TRNA_RS23705 | BL14DL4_03045 | BLDA23_02245 | CK945_RS02495 | ACH97_214655 |
| MarR family transcriptional regulator | TRNA_RS23710 | BL14DL4_03046 | BLDA23_02250 | CK945_RS02500 | ACH97_214650 |
| beta-galactosidase | TRNA_RS23715 | BL14DL4_03047 | BLDA23_02255 | CK945_RS02505 | ACH97_214645 |
| 3-phytase | TRNA_RS23720 | BL14DL4_03048 | BLDA23_02260 | CK945_RS02510 | ACH97_214640 |
| peptide MFS transporter | TRNA_RS23725 | BL14DL4_03049 | BLDA23_02265 | CK945_RS02520 | ACH97_214630 |
| hypothetical protein | TRNA_RS23730 | BL14DL4_03050 | BLDA23_02270 | CK945_RS02525 | ACH97_214625 |
| hypothetical protein | TRNA_RS23735 | BL14DL4_03051 | BLDA23_02275 | CK945_RS02530 | ACH97_217080 |
| spore germination protein | TRNA_RS23740 | BL14DL4_03052 | BLDA23_02280 | CK945_RS02535 | ACH97_217075 |
| Ger(x)C family spore germination protein | TRNA_RS23745 | BL14DL4_03053 | BLDA23_02285 | CK945_RS02540 | ACH97_217070 |
| MerR family transcriptional regulator | TRNA_RS23755 | BL14DL4_03055 | BLDA23_02295 | CK945_RS02550 | ACH97_217060 |
| ABC transporter ATP-binding protein | TRNA_RS23760 | BL14DL4_03056 | BLDA23_02300 | CK945_RS02555 | ACH97_217055 |
| ABC transporter permease | TRNA_RS23765 | BL14DL4_03057 | BLDA23_02305 | CK945_RS02560 | ACH97_217050 |
| DNA-binding response regulator | TRNA_RS23770 | BL14DL4_03058 | BLDA23_02310 | CK945_RS02565 | ACH97_217045 |
| sensor histidine kinase | TRNA_RS23775 | BL14DL4_03059 | BLDA23_02315 | CK945_RS02570 | ACH97_217040 |
| aspartate kinase | TRNA_RS23785 | BL14DL4_03061 | BLDA23_02325 | CK945_RS02580 | ACH97_217030 |
| ABC transporter permease | TRNA_RS23790 | BL14DL4_03062 | BLDA23_02330 | CK945_RS02585 | ACH97_217025 |
| iron ABC transporter permease | TRNA_RS23795 | BL14DL4_03063 | BLDA23_02335 | CK945_RS02590 | ACH97_217020 |
| ATP-binding cassette domain-containing protein | TRNA_RS23800 | BL14DL4_03064 | BLDA23_02340 | CK945_RS02595 | ACH97_217015 |
| siderophore ABC transporter substrate-binding protein | TRNA_RS23805 | BL14DL4_03065 | BLDA23_02345 | CK945_RS02600 | ACH97_217010 |
| DHA2 family efflux MFS transporter permease subunit | TRNA_RS23810 | BL14DL4_03066 | BLDA23_02350 | CK945_RS02605 | ACH97_217005 |
| TetR/AcrR family transcriptional regulator | TRNA_RS23815 | BL14DL4_03067 | BLDA23_02355 | CK945_RS02610 | ACH97_217000 |
| isocitrate lyase/phosphoenolpyruvate mutase family protein | TRNA_RS23820 | BL14DL4_03068 | BLDA23_02360 | CK945_RS02615 | ACH97_216995 |
| NADPH-dependent oxidoreductase | TRNA_RS23825 | BL14DL4_03069 | BLDA23_02365 | CK945_RS02620 | ACH97_216990 |
| antibiotic biosynthesis monooxygenase | TRNA_RS23830 | BL14DL4_03070 | BLDA23_02370 | CK945_RS02625 | ACH97_216985 |
| ArsR family transcriptional regulator | TRNA_RS23835 | BL14DL4_03071 | BLDA23_02375 | CK945_RS02630 | ACH97_216980 |
| PLP-dependent aminotransferase family protein | TRNA_RS23840 | BL14DL4_03072 | BLDA23_02380 | CK945_RS02635 | ACH97_216975 |
| 4-aminobutyrate--2-oxoglutarate transaminase | TRNA_RS23845 | BL14DL4_03073 | BLDA23_02385 | CK945_RS02640 | ACH97_216970 |
| APC family permease | TRNA_RS23850 | BL14DL4_03074 | BLDA23_02390 | CK945_RS02645 | ACH97_216965 |
| NAD-dependent succinate-semialdehyde dehydrogenase | TRNA_RS23855 | BL14DL4_03075 | BLDA23_02395 | CK945_RS02650 | ACH97_216960 |
| EamA family transporter | TRNA_RS23860 | BL14DL4_03076 | BLDA23_02400 | CK945_RS02655 | ACH97_216955 |
| hypothetical protein | TRNA_RS23865 | BL14DL4_03077 | BLDA23_02405 | CK945_RS02660 | ACH97_216950 |
| DUF1775 domain-containing protein | TRNA_RS23870 | BL14DL4_03078 | BLDA23_02410 | CK945_RS02665 | ACH97_216945 |
| copper transporter | TRNA_RS23875 | BL14DL4_03079 | BLDA23_02415 | CK945_RS02670 | ACH97_216940 |
| DeoR family transcriptional regulator | TRNA_RS23880 | BL14DL4_03080 | BLDA23_02420 | CK945_RS02675 | ACH97_216935 |
| NAD(P)/FAD-dependent oxidoreductase | TRNA_RS23885 | BL14DL4_03081 | BLDA23_02425 | CK945_RS02680 | ACH97_216930 |
| nitrite reductase | TRNA_RS23890 | BL14DL4_03082 | BLDA23_02430 | CK945_RS02685 | ACH97_216925 |
| NAD(P)/FAD-dependent oxidoreductase | TRNA_RS23895 | BL14DL4_03083 | BLDA23_02435 | CK945_RS02690 | ACH97_216920 |
| nitrite reductase (NAD(P)H) small subunit | TRNA_RS23900 | BL14DL4_03084 | BLDA23_02440 | CK945_RS02695 | ACH97_216915 |
| uroporphyrinogen-III C-methyltransferase | TRNA_RS23905 | BL14DL4_03085 | BLDA23_02445 | CK945_RS02700 | ACH97_216910 |
| GntR family transcriptional regulator | TRNA_RS23910 | BL14DL4_03086 | BLDA23_02450 | CK945_RS02705 | ACH97_216905 |
| DASS family sodium-coupled anion symporter | TRNA_RS23915 | BL14DL4_03087 | BLDA23_02455 | CK945_RS02710 | ACH97_216900 |
| tRNA (N6-threonylcarbamoyladenosine(37)-N6)-methyltransferase TrmO | TRNA_RS23930 | BL14DL4_03089 | BLDA23_02470 | CK945_RS02720 | ACH97_216890 |
| PTS sugar transporter subunit IIB | TRNA_RS23945 | BL14DL4_03091 | BLDA23_02485 | CK945_RS02730 | ACH97_216880 |
| transketolase | TRNA_RS23955 | BL14DL4_03093 | BLDA23_02495 | CK945_RS02740 | ACH97_216870 |
| transketolase | TRNA_RS23960 | BL14DL4_03094 | BLDA23_02500 | CK945_RS02745 | ACH97_216865 |
| HAD family phosphatase | TRNA_RS23965 | BL14DL4_03095 | BLDA23_02505 | CK945_RS02750 | ACH97_216860 |
| 5-oxoprolinase subunit PxpA | TRNA_RS23970 | BL14DL4_03096 | BLDA23_02510 | CK945_RS02755 | ACH97_216855 |
| divalent metal cation transporter | TRNA_RS23975 | BL14DL4_03097 | BLDA23_02515 | CK945_RS02760 | ACH97_216850 |
| putative hydro-lyase | TRNA_RS23980 | BL14DL4_03098 | BLDA23_02520 | CK945_RS02765 | ACH97_216845 |
| 5-oxoprolinase subunit PxpB | TRNA_RS23985 | BL14DL4_03099 | BLDA23_02525 | CK945_RS02770 | ACH97_216840 |
| biotin-dependent carboxyltransferase family protein | TRNA_RS23990 | BL14DL4_03100 | BLDA23_02530 | CK945_RS02775 | ACH97_216835 |
| IclR family transcriptional regulator | TRNA_RS23995 | BL14DL4_03101 | BLDA23_02535 | CK945_RS02780 | ACH97_216830 |
| germination protein | TRNA_RS24000 | BL14DL4_03102 | BLDA23_02540 | CK945_RS02785 | ACH97_216825 |
| PTS mannitol transporter subunit IICBA | TRNA_RS24005 | BL14DL4_03103 | BLDA23_02545 | CK945_RS02790 | ACH97_216820 |
| PTS mannitol transporter subunit IIA | TRNA_RS24010 | BL14DL4_03104 | BLDA23_02550 | CK945_RS02795 | ACH97_216815 |
| mannitol-1-phosphate 5-dehydrogenase | TRNA_RS24015 | BL14DL4_03105 | BLDA23_02555 | CK945_RS02800 | ACH97_216810 |
| PRD domain-containing protein | TRNA_RS24020 | BL14DL4_03106 | BLDA23_02560 | CK945_RS02805 | ACH97_216805 |
| D-lyxose/D-mannose family sugar isomerase | TRNA_RS24030 | BL14DL4_03108 | BLDA23_02570 | CK945_RS02815 | ACH97_216795 |
| DUF2283 domain-containing protein | TRNA_RS24035 | BL14DL4_03109 | BLDA23_02575 | CK945_RS02820 | ACH97_216790 |
| general stress protein | TRNA_RS24040 | BL14DL4_03110 | BLDA23_02580 | CK945_RS02825 | ACH97_216785 |
| DUF2837 family protein | TRNA_RS24045 | BL14DL4_03111 | BLDA23_02585 | CK945_RS02830 | ACH97_216780 |
| LytR family transcriptional regulator | TRNA_RS24050 | BL14DL4_03112 | BLDA23_02590 | CK945_RS02835 | ACH97_216775 |
| DUF3817 domain-containing protein | TRNA_RS24055 | BL14DL4_03113 | BLDA23_02595 | CK945_RS02840 | ACH97_216770 |
| carboxymuconolactone decarboxylase family protein | TRNA_RS24060 | BL14DL4_03114 | BLDA23_02600 | CK945_RS02845 | ACH97_216765 |
| Lrp/AsnC family transcriptional regulator | TRNA_RS24065 | BL14DL4_03115 | BLDA23_02605 | CK945_RS02850 | ACH97_216760 |
| DNA topoisomerase III | TRNA_RS24070 | BL14DL4_03116 | BLDA23_02610 | CK945_RS02855 | ACH97_216755 |
| pyruvate oxidase | TRNA_RS24080 | BL14DL4_03118 | BLDA23_02620 | CK945_RS02875 | ACH97_216735 |
| chitin-binding protein | TRNA_RS24085 | BL14DL4_03119 | BLDA23_02625 | CK945_RS02880 | ACH97_216730 |
| hypothetical protein | TRNA_RS24100 | BL14DL4_03120 | BLDA23_02635 | CK945_RS02885 | ACH97_216725 |
| N-acetyltransferase | TRNA_RS24105 | BL14DL4_03121 | BLDA23_02640 | CK945_RS02890 | ACH97_216720 |
| divalent metal cation transporter | TRNA_RS24110 | BL14DL4_03122 | BLDA23_02645 | CK945_RS02895 | ACH97_216715 |
| GlsB/YeaQ/YmgE family stress response membrane protein | TRNA_RS24115 | BL14DL4_03123 | BLDA23_02650 | CK945_RS02900 | ACH97_216710 |
| aspartate ammonia-lyase | TRNA_RS24120 | BL14DL4_03124 | BLDA23_02655 | CK945_RS02905 | ACH97_216705 |
| glycosyl transferase family 1 | TRNA_RS24125 | BL14DL4_03125 | BLDA23_02660 | CK945_RS02910 | ACH97_216700 |
| DUF2188 domain-containing protein | TRNA_RS24130 | BL14DL4_03126 | BLDA23_02665 | CK945_RS02915 | ACH97_216695 |
| EcsC family protein | TRNA_RS24135 | BL14DL4_03127 | BLDA23_02670 | CK945_RS02920 | ACH97_216690 |
| Na+/H+ antiporter NhaC family protein | TRNA_RS24140 | BL14DL4_03128 | BLDA23_02675 | CK945_RS02925 | ACH97_216685 |
| DUF4937 domain-containing protein | TRNA_RS24145 | BL14DL4_03129 | BLDA23_02680 | CK945_RS02930 | ACH97_216680 |
| glucose starvation-inducible protein B | TRNA_RS24150 | BL14DL4_03130 | BLDA23_02685 | CK945_RS02935 | ACH97_216675 |
| AI-2E family transporter | TRNA_RS24155 | BL14DL4_03131 | BLDA23_02690 | CK945_RS02940 | ACH97_216665 |
| dicarboxylate/amino acid:cation symporter | TRNA_RS24160 | BL14DL4_03132 | BLDA23_02695 | CK945_RS02945 | ACH97_216660 |
| ABC transporter ATP-binding protein | TRNA_RS24165 | BL14DL4_03133 | BLDA23_02700 | CK945_RS02950 | ACH97_216655 |
| ABC transporter permease | TRNA_RS24170 | BL14DL4_03134 | BLDA23_02705 | CK945_RS02955 | ACH97_216650 |
| hypothetical protein | TRNA_RS24175 | BL14DL4_03135 | BLDA23_02710 | CK945_RS02960 | ACH97_216645 |
| Fur-regulated basic protein FbpB | TRNA_RS24185 | BL14DL4_03137 | BLDA23_02720 | CK945_RS02970 | ACH97_216635 |
| Fur-regulated basic protein FbpA | TRNA_RS24190 | BL14DL4_03138 | BLDA23_02725 | CK945_RS02975 | ACH97_216630 |
| thioredoxin | TRNA_RS24195 | BL14DL4_03139 | BLDA23_02730 | CK945_RS02980 | ACH97_216625 |
| D-alanine--D-alanine ligase | TRNA_RS24200 | BL14DL4_03140 | BLDA23_02735 | CK945_RS02985 | ACH97_216620 |
| UDP-N-acetylmuramoyl-tripeptide--D-alanyl-D- alanine ligase | TRNA_RS24205 | BL14DL4_03141 | BLDA23_02740 | CK945_RS02990 | ACH97_216615 |
| alpha/beta fold hydrolase | TRNA_RS24210 | BL14DL4_03142 | BLDA23_02745 | CK945_RS02995 | ACH97_216610 |
| DEAD/DEAH box family ATP-dependent RNA helicase | TRNA_RS24215 | BL14DL4_03143 | BLDA23_02750 | CK945_RS03000 | ACH97_216605 |
| membrane protein | TRNA_RS24220 | BL14DL4_03144 | BLDA23_02755 | CK945_RS03005 | ACH97_216600 |
| membrane protein | TRNA_RS24225 | BL14DL4_03145 | BLDA23_02760 | CK945_RS03010 | ACH97_216595 |
| rhomboid family intramembrane serine protease | TRNA_RS24230 | BL14DL4_03146 | BLDA23_02765 | CK945_RS03015 | ACH97_216590 |
| outer membrane lipoprotein carrier protein LolA | TRNA_RS24235 | BL14DL4_03147 | BLDA23_02770 | CK945_RS03020 | ACH97_216585 |
| alanine racemase | TRNA_RS24240 | BL14DL4_03148 | BLDA23_02775 | CK945_RS03025 | ACH97_216580 |
| hypothetical protein | TRNA_RS24245 | BL14DL4_03149 | BLDA23_02780 | CK945_RS03030 | ACH97_216575 |
| mRNA interferase EndoA | TRNA_RS24250 | BL14DL4_03150 | BLDA23_02785 | CK945_RS03035 | ACH97_216570 |
| STAS domain-containing protein | TRNA_RS24255 | BL14DL4_03151 | BLDA23_02790 | CK945_RS03040 | ACH97_216565 |
| STAS domain-containing protein | TRNA_RS24260 | BL14DL4_03152 | BLDA23_02795 | CK945_RS03045 | ACH97_216560 |
| anti-sigma regulatory factor | TRNA_RS24265 | BL14DL4_03153 | BLDA23_02800 | CK945_RS03050 | ACH97_216555 |
| phosphoserine phosphatase | TRNA_RS24270 | BL14DL4_03154 | BLDA23_02805 | CK945_RS03055 | ACH97_216550 |
| STAS domain-containing protein | TRNA_RS24275 | BL14DL4_03155 | BLDA23_02810 | CK945_RS03060 | ACH97_216545 |
| anti-sigma B factor RsbW | TRNA_RS24280 | BL14DL4_03156 | BLDA23_02815 | CK945_RS03065 | ACH97_216540 |
| RNA polymerase sigma factor SigB | TRNA_RS24285 | BL14DL4_03157 | BLDA23_02820 | CK945_RS03070 | ACH97_216535 |
| phosphoserine phosphatase | TRNA_RS24290 | BL14DL4_03158 | BLDA23_02825 | CK945_RS03075 | ACH97_216530 |
| RNA-binding transcriptional accessory protein | TRNA_RS24295 | BL14DL4_03159 | BLDA23_02830 | CK945_RS03080 | ACH97_216525 |
| TetR/AcrR family transcriptional regulator | TRNA_RS24300 | BL14DL4_03160 | BLDA23_02835 | CK945_RS03085 | ACH97_216520 |
| SprT family protein | TRNA_RS24310 | BL14DL4_03162 | BLDA23_02850 | CK945_RS03105 | ACH97_216505 |
| cold-shock protein | TRNA_RS24375 | BL14DL4_03173 | BLDA23_02905 | CK945_RS03185 | ACH97_216445 |
| hypothetical protein | TRNA_RS24380 | BL14DL4_03174 | BLDA23_02910 | CK945_RS03190 | ACH97_216440 |
| MarR family transcriptional regulator | TRNA_RS24395 | BL14DL4_03177 | BLDA23_02925 | CK945_RS03215 | ACH97_216415 |
| spermidine acetyltransferase | TRNA_RS24400 | BL14DL4_03178 | BLDA23_02930 | CK945_RS03220 | ACH97_216410 |
| FMN-binding negative transcriptional regulator | TRNA_RS24405 | BL14DL4_03179 | BLDA23_02935 | CK945_RS03225 | ACH97_216405 |
| arginase family protein | TRNA_RS24410 | BL14DL4_03180 | BLDA23_02940 | CK945_RS03230 | ACH97_216400 |
| transcriptional regulator | TRNA_RS24415 | BL14DL4_03181 | BLDA23_02945 | CK945_RS03235 | ACH97_216395 |
| YitT family protein | TRNA_RS24420 | BL14DL4_03182 | BLDA23_02950 | CK945_RS03240 | ACH97_216390 |
| hypothetical protein | TRNA_RS24435 | BL14DL4_03185 | BLDA23_02965 | CK945_RS03265 | ACH97_216355 |
| cyclase family protein | TRNA_RS24445 | BL14DL4_03187 | BLDA23_02975 | CK945_RS03275 | ACH97_216345 |
| amino acid permease | TRNA_RS24450 | BL14DL4_03188 | BLDA23_02980 | CK945_RS23010 | ACH97_216340 |
| DUF2306 domain-containing protein | TRNA_RS24455 | BL14DL4_03189 | BLDA23_02985 | CK945_RS03285 | ACH97_216335 |
| sigma-70 family RNA polymerase sigma factor | TRNA_RS24465 | BL14DL4_03191 | BLDA23_02995 | CK945_RS03305 | ACH97_216315 |
| DUF3298 domain-containing protein | TRNA_RS24470 | BL14DL4_03192 | BLDA23_03000 | CK945_RS03310 | ACH97_216310 |
| acetyltransferase | TRNA_RS24475 | BL14DL4_03193 | BLDA23_03005 | CK945_RS03315 | ACH97_216305 |
| MFS transporter | TRNA_RS24480 | BL14DL4_03194 | BLDA23_03010 | CK945_RS03325 | ACH97_216295 |
| DNA-binding response regulator | TRNA_RS24490 | BL14DL4_03196 | BLDA23_03020 | CK945_RS03340 | ACH97_216280 |
| LPXTG cell wall anchor domain-containing protein | TRNA_RS24495 | BL14DL4_03197 | BLDA23_03025 | CK945_RS03345 | ACH97_216275 |
| class A sortase | TRNA_RS24500 | BL14DL4_03198 | BLDA23_03030 | CK945_RS03350 | ACH97_216270 |
| sugar porter family MFS transporter | TRNA_RS24505 | BL14DL4_03199 | BLDA23_03035 | CK945_RS20720 | ACH97_216260 |
| thiamine-phosphate kinase | TRNA_RS24545 | BL14DL4_03207 | BLDA23_03075 | CK945_RS03400 | ACH97_218770 |
| tRNA (adenosine(37)-N6)-threonylcarbamoyltransferase complex ATPase subunit type 1 TsaE | TRNA_RS24550 | BL14DL4_03208 | BLDA23_03080 | CK945_RS03405 | ACH97_218775 |
| tRNA (adenosine(37)-N6)-threonylcarbamoyltransferase complex dimerization subunit type 1 TsaB | TRNA_RS24555 | BL14DL4_03209 | BLDA23_03085 | CK945_RS03410 | ACH97_218780 |
| ribosomal-protein-alanine N-acetyltransferase | TRNA_RS24560 | BL14DL4_03210 | BLDA23_03090 | CK945_RS03415 | ACH97_218785 |
| tRNA (adenosine(37)-N6)-threonylcarbamoyltransferase complex transferase subunit TsaD | TRNA_RS24565 | BL14DL4_03211 | BLDA23_03095 | CK945_RS03420 | ACH97_218790 |
| ABC transporter ATP-binding protein | TRNA_RS24570 | BL14DL4_03212 | BLDA23_03100 | CK945_RS03425 | ACH97_218795 |
| cyclic pyranopterin monophosphate synthase MoaC | TRNA_RS24575 | BL14DL4_03213 | BLDA23_03105 | CK945_RS03430 | ACH97_218800 |
| redox-sensing transcriptional repressor Rex | TRNA_RS24580 | BL14DL4_03214 | BLDA23_03110 | CK945_RS03435 | ACH97_218805 |
| twin-arginine translocase TatA/TatE family subunit | TRNA_RS24585 | BL14DL4_03215 | BLDA23_03115 | CK945_RS03440 | ACH97_218810 |
| twin-arginine translocase subunit TatC | TRNA_RS24590 | BL14DL4_03216 | BLDA23_03120 | CK945_RS03445 | ACH97_218815 |
| DUF4305 domain-containing protein | TRNA_RS24595 | BL14DL4_03217 | BLDA23_03125 | CK945_RS03450 | ACH97_218820 |
| CPBP family intramembrane metalloprotease | TRNA_RS24600 | BL14DL4_03218 | BLDA23_03130 | CK945_RS03455 | ACH97_218825 |
| co-chaperone GroES | TRNA_RS24605 | BL14DL4_03219 | BLDA23_03135 | CK945_RS03460 | ACH97_218830 |
| chaperonin GroEL | TRNA_RS24610 | BL14DL4_03220 | BLDA23_03140 | CK945_RS03465 | ACH97_218835 |
| hypothetical protein | TRNA_RS24640 | BL14DL4_03225 | BLDA23_03435 | CK945_RS03870 | ACH97_204840 |
| hypothetical protein | TRNA_RS24645 | BL14DL4_03226 | BLDA23_03440 | CK945_RS03875 | ACH97_204845 |
| hypothetical protein | TRNA_RS24650 | BL14DL4_03227 | BLDA23_03445 | CK945_RS03880 | ACH97_204850 |
| GNAT family N-acetyltransferase | TRNA_RS24655 | BL14DL4_03228 | BLDA23_03450 | CK945_RS03890 | ACH97_204860 |
| SOS response-associated peptidase | TRNA_RS24660 | BL14DL4_03229 | BLDA23_03455 | CK945_RS03895 | ACH97_204865 |
| hypothetical protein | TRNA_RS24680 | BL14DL4_03232 | BLDA23_03475 | CK945_RS03915 | ACH97_204885 |
| DUF488 domain-containing protein | TRNA_RS24685 | BL14DL4_03233 | BLDA23_03480 | CK945_RS03920 | ACH97_204890 |
| SDR family NAD(P)-dependent oxidoreductase | TRNA_RS24690 | BL14DL4_03234 | BLDA23_03485 | CK945_RS03925 | ACH97_204895 |
| carboxylesterase/lipase family protein | TRNA_RS24695 | BL14DL4_03235 | BLDA23_03490 | CK945_RS03930 | ACH97_204900 |
| MFS transporter | TRNA_RS24700 | BL14DL4_03236 | BLDA23_03495 | CK945_RS03935 | ACH97_204905 |
| PspA/IM30 family protein | TRNA_RS24705 | BL14DL4_03237 | BLDA23_03500 | CK945_RS03940 | ACH97_204910 |
| TFIIB-type zinc ribbon-containing protein | TRNA_RS24710 | BL14DL4_03238 | BLDA23_03505 | CK945_RS03945 | ACH97_204915 |
| hypothetical protein | TRNA_RS24715 | BL14DL4_03239 | BLDA23_03510 | CK945_RS03950 | ACH97_204920 |
| hypothetical protein | TRNA_RS24720 | BL14DL4_03240 | BLDA23_03515 | CK945_RS03955 | ACH97_204925 |
| LLM class flavin-dependent oxidoreductase | TRNA_RS24725 | BL14DL4_03241 | BLDA23_03520 | CK945_RS03960 | ACH97_204930 |
| TrmB family transcriptional regulator | TRNA_RS24730 | BL14DL4_03242 | BLDA23_03525 | CK945_RS03965 | ACH97_204935 |
| helix-turn-helix domain-containing protein | TRNA_RS24740 | BL14DL4_03244 | BLDA23_03535 | CK945_RS03975 | ACH97_204945 |
| rod shape-determining protein RodA | TRNA_RS24745 | BL14DL4_03245 | BLDA23_03540 | CK945_RS03980 | ACH97_204950 |
| putative lipid II flippase FtsW | TRNA_RS24750 | BL14DL4_03246 | BLDA23_03545 | CK945_RS03985 | ACH97_204955 |
| VOC family protein | TRNA_RS24760 | BL14DL4_03247 | BLDA23_03555 | CK945_RS03990 | ACH97_204960 |
| alpha-amylase | TRNA_RS24780 | BL14DL4_03251 | BLDA23_03575 | CK945_RS04010 | ACH97_204980 |
| LacI family transcriptional regulator | TRNA_RS24785 | BL14DL4_03252 | BLDA23_03580 | CK945_RS04015 | ACH97_204985 |
| alpha-glycosidase | TRNA_RS24790 | BL14DL4_03253 | BLDA23_03585 | CK945_RS04020 | ACH97_204990 |
| extracellular solute-binding protein | TRNA_RS24795 | BL14DL4_03254 | BLDA23_03590 | CK945_RS04025 | ACH97_204995 |
| sugar ABC transporter permease | TRNA_RS24800 | BL14DL4_03255 | BLDA23_03595 | CK945_RS04030 | ACH97_205000 |
| sugar ABC transporter permease | TRNA_RS24805 | BL14DL4_03256 | BLDA23_03600 | CK945_RS04035 | ACH97_205005 |
| DUF1189 domain-containing protein | TRNA_RS24810 | BL14DL4_03257 | BLDA23_03605 | CK945_RS04040 | ACH97_205010 |
| glycoside hydrolase family 65 protein | TRNA_RS24815 | BL14DL4_03258 | BLDA23_03610 | CK945_RS04045 | ACH97_205015 |
| alpha-glucosidase | TRNA_RS24820 | BL14DL4_03259 | BLDA23_03615 | CK945_RS04050 | ACH97_205020 |
| beta-phosphoglucomutase | TRNA_RS24825 | BL14DL4_03260 | BLDA23_03620 | CK945_RS04055 | ACH97_205025 |
| tyrosine--tRNA ligase | TRNA_RS24830 | BL14DL4_03261 | BLDA23_03625 | CK945_RS04060 | ACH97_205030 |
| MarR family transcriptional regulator | TRNA_RS24835 | BL14DL4_03262 | BLDA23_03630 | CK945_RS04065 | ACH97_205035 |
| hypothetical protein | TRNA_RS24840 | BL14DL4_03263 | BLDA23_03635 | CK945_RS04070 | ACH97_205040 |
| hypothetical protein | TRNA_RS24845 | BL14DL4_03264 | BLDA23_03640 | CK945_RS04075 | ACH97_205045 |
| hypothetical protein | TRNA_RS24850 | BL14DL4_03265 | BLDA23_03645 | CK945_RS04080 | ACH97_205050 |
| hypothetical protein | TRNA_RS24855 | BL14DL4_03266 | BLDA23_03650 | CK945_RS04085 | ACH97_205055 |
| DUF4003 domain-containing protein | TRNA_RS24860 | BL14DL4_03267 | BLDA23_03655 | CK945_RS04090 | ACH97_205060 |
| hypothetical protein | TRNA_RS24865 | BL14DL4_03268 | BLDA23_03660 | CK945_RS04095 | ACH97_205065 |
| hypothetical protein | TRNA_RS24870 | BL14DL4_03269 | BLDA23_03665 | CK945_RS04100 | ACH97_205070 |
| signal peptidase I | TRNA_RS24875 | BL14DL4_03270 | BLDA23_03670 | CK945_RS04105 | ACH97_205075 |
| hypothetical protein | TRNA_RS24880 | BL14DL4_03271 | BLDA23_03675 | CK945_RS15055 | ACH97_205080 |
| SDR family oxidoreductase | TRNA_RS24885 | BL14DL4_03272 | BLDA23_03680 | CK945_RS04110 | ACH97_205085 |
| helix-turn-helix domain-containing protein | TRNA_RS24890 | BL14DL4_03273 | BLDA23_03685 | CK945_RS04115 | ACH97_205090 |
| copper oxidase | TRNA_RS24895 | BL14DL4_03274 | BLDA23_03690 | CK945_RS04120 | ACH97_205095 |
| acyltransferase | TRNA_RS24900 | BL14DL4_03275 | BLDA23_03695 | CK945_RS04125 | ACH97_205100 |
| DUF4352 domain-containing protein | TRNA_RS24905 | BL14DL4_03276 | BLDA23_03700 | CK945_RS04130 | ACH97_205105 |
| cation transporter | TRNA_RS24910 | BL14DL4_03277 | BLDA23_03705 | CK945_RS04140 | ACH97_205110 |
| MoxR family ATPase | TRNA_RS24915 | BL14DL4_03278 | BLDA23_03710 | CK945_RS04145 | ACH97_205115 |
| DUF58 domain-containing protein | TRNA_RS24920 | BL14DL4_03279 | BLDA23_03715 | CK945_RS04150 | ACH97_205120 |
| DUF4129 domain-containing protein | TRNA_RS24925 | BL14DL4_03280 | BLDA23_03720 | CK945_RS04155 | ACH97_205125 |
| GMP synthase (glutamine-hydrolyzing) | TRNA_RS24930 | BL14DL4_03281 | BLDA23_03725 | CK945_RS04160 | ACH97_205130 |
| NCS2 family permease | TRNA_RS24940 | BL14DL4_03282 | BLDA23_03735 | CK945_RS04170 | ACH97_205140 |
| hypothetical protein | TRNA_RS24945 | BL14DL4_03283 | BLDA23_03740 | CK945_RS04175 | ACH97_205145 |
| hypothetical protein | TRNA_RS24950 | BL14DL4_03284 | BLDA23_03745 | CK945_RS04180 | ACH97_205150 |
| DUF2179 domain-containing protein | TRNA_RS24955 | BL14DL4_03285 | BLDA23_03750 | CK945_RS04185 | ACH97_205155 |
| NETI motif-containing protein | TRNA_RS24960 | BL14DL4_03286 | BLDA23_03755 | CK945_RS04190 | ACH97_205160 |
| 5-(carboxyamino)imidazole ribonucleotide mutase | TRNA_RS24965 | BL14DL4_03287 | BLDA23_03760 | CK945_RS04195 | ACH97_205165 |
| 5-(carboxyamino)imidazole ribonucleotide synthase | TRNA_RS24970 | BL14DL4_03288 | BLDA23_03765 | CK945_RS04200 | ACH97_205170 |
| adenylosuccinate lyase | TRNA_RS24975 | BL14DL4_03289 | BLDA23_03770 | CK945_RS04205 | ACH97_205175 |
| phosphoribosylaminoimidazolesuccinocarboxamide synthase | TRNA_RS24980 | BL14DL4_03290 | BLDA23_03775 | CK945_RS04210 | ACH97_205180 |
| phosphoribosylformylglycinamidine synthase, purS protein | TRNA_RS24985 | BL14DL4_03291 | BLDA23_03780 | CK945_RS04215 | ACH97_205185 |
| phosphoribosylformylglycinamidine synthase subunit PurQ | TRNA_RS24990 | BL14DL4_03292 | BLDA23_03785 | CK945_RS04220 | ACH97_205190 |
| phosphoribosylformylglycinamidine synthase subunit PurL | TRNA_RS24995 | BL14DL4_03293 | BLDA23_03790 | CK945_RS04225 | ACH97_205195 |
| amidophosphoribosyltransferase | TRNA_RS25000 | BL14DL4_03294 | BLDA23_03795 | CK945_RS04230 | ACH97_205200 |
| phosphoribosylformylglycinamidine cyclo-ligase | TRNA_RS25005 | BL14DL4_03295 | BLDA23_03800 | CK945_RS04235 | ACH97_205205 |
| phosphoribosylglycinamide formyltransferase | TRNA_RS25010 | BL14DL4_03296 | BLDA23_03805 | CK945_RS04240 | ACH97_205210 |
| bifunctional phosphoribosylaminoimidazolecarboxamide formyltransferase/IMP cyclohydrolase | TRNA_RS25015 | BL14DL4_03297 | BLDA23_03810 | CK945_RS04245 | ACH97_205215 |
| phosphoribosylamine--glycine ligase | TRNA_RS25020 | BL14DL4_03298 | BLDA23_03815 | CK945_RS04250 | ACH97_205220 |
| TetR/AcrR family transcriptional regulator | TRNA_RS25025 | BL14DL4_03299 | BLDA23_03825 | CK945_RS04255 | ACH97_205225 |
| cytochrome P450 | TRNA_RS25030 | BL14DL4_03300 | BLDA23_03830 | CK945_RS04260 | ACH97_205230 |
| hypothetical protein | TRNA_RS25035 | BL14DL4_03302 | BLDA23_03835 | CK945_RS04265 | ACH97_205235 |
| DUF2892 domain-containing protein | TRNA_RS25045 | BL14DL4_03304 | BLDA23_03845 | CK945_RS04275 | ACH97_205245 |
| adenine deaminase | TRNA_RS25050 | BL14DL4_03305 | BLDA23_03850 | CK945_RS04280 | ACH97_205250 |
| DUF3048 domain-containing protein | TRNA_RS25055 | BL14DL4_03306 | BLDA23_03855 | CK945_RS04285 | ACH97_205255 |
| hypothetical protein | TRNA_RS25060 | BL14DL4_03307 | BLDA23_03860 | CK945_RS04290 | ACH97_205260 |
| geranylgeranylglyceryl/heptaprenylglyceryl phosphate synthase | TRNA_RS25065 | BL14DL4_03308 | BLDA23_03865 | CK945_RS04295 | ACH97_205265 |
| DNA helicase PcrA | TRNA_RS25070 | BL14DL4_03309 | BLDA23_03870 | CK945_RS04300 | ACH97_205270 |
| DNA ligase (NAD(+)) LigA | TRNA_RS25075 | BL14DL4_03310 | BLDA23_03875 | CK945_RS04305 | ACH97_205275 |
| hypothetical protein | TRNA_RS25080 | BL14DL4_03311 | BLDA23_03880 | CK945_RS04310 | ACH97_205280 |
| transcriptional regulator | TRNA_RS25085 | BL14DL4_03312 | BLDA23_03885 | CK945_RS04315 | ACH97_205285 |
| PRD domain-containing protein | TRNA_RS25090 | BL14DL4_03313 | BLDA23_03890 | CK945_RS04320 | ACH97_205290 |
| DUF2620 domain-containing protein | TRNA_RS25095 | BL14DL4_03314 | BLDA23_03895 | CK945_RS04325 | ACH97_205295 |
| membrane protein | TRNA_RS25100 | BL14DL4_03315 | BLDA23_03900 | CK945_RS04330 | ACH97_205300 |
| hydrolase | TRNA_RS25105 | BL14DL4_03316 | BLDA23_03905 | CK945_RS04335 | ACH97_205305 |
| aminotransferase class V-fold PLP-dependent enzyme | TRNA_RS25110 | BL14DL4_03317 | BLDA23_03910 | CK945_RS04340 | ACH97_205310 |
| phosphopentomutase | TRNA_RS25115 | BL14DL4_03318 | BLDA23_03915 | CK945_RS04345 | ACH97_205315 |
| YhfX family PLP-dependent enzyme | TRNA_RS25120 | BL14DL4_03319 | BLDA23_03920 | CK945_RS04350 | ACH97_205320 |
| glucosamine-6-phosphate deaminase | TRNA_RS25125 | BL14DL4_03320 | BLDA23_03925 | CK945_RS04355 | ACH97_205325 |
| MgtC/SapB family protein | TRNA_RS25130 | BL14DL4_03321 | BLDA23_03930 | CK945_RS04360 | ACH97_205330 |
| sodium/proline symporter PutP | TRNA_RS25135 | BL14DL4_03322 | BLDA23_03935 | CK945_RS04365 | ACH97_205335 |
| Asp-tRNA(Asn)/Glu-tRNA(Gln) amidotransferase GatCAB subunit C | TRNA_RS25140 | BL14DL4_03324 | BLDA23_03940 | CK945_RS04370 | ACH97_205340 |
| Asp-tRNA(Asn)/Glu-tRNA(Gln) amidotransferase subunit GatA | TRNA_RS25145 | BL14DL4_03325 | BLDA23_03945 | CK945_RS04375 | ACH97_205345 |
| Asp-tRNA(Asn)/Glu-tRNA(Gln) amidotransferase GatCAB subunit B | TRNA_RS25150 | BL14DL4_03326 | BLDA23_03950 | CK945_RS04380 | ACH97_205350 |
| DUF2089 family protein | TRNA_RS25155 | BL14DL4_03327 | BLDA23_03955 | CK945_RS04385 | ACH97_205355 |
| hypothetical protein | TRNA_RS25160 | BL14DL4_03328 | BLDA23_03960 | CK945_RS04390 | ACH97_205360 |
| mannan endo-1,4-beta-mannosidase | TRNA_RS25165 | BL14DL4_03329 | BLDA23_03965 | CK945_RS04395 | ACH97_205365 |
| TetR/AcrR family transcriptional regulator | TRNA_RS25170 | BL14DL4_03330 | BLDA23_03970 | CK945_RS04400 | ACH97_205370 |
| AcrB/AcrD/AcrF family protein | TRNA_RS25175 | BL14DL4_03331 | BLDA23_03975 | CK945_RS04405 | ACH97_205375 |
| hypothetical protein | TRNA_RS25180 | BL14DL4_03332 | BLDA23_03980 | CK945_RS04410 | ACH97_205380 |
| N-acetyltransferase | TRNA_RS25185 | BL14DL4_03333 | BLDA23_03985 | CK945_RS04415 | ACH97_205385 |
| inosine-uridine preferring nucleoside hydrolase | TRNA_RS25190 | BL14DL4_03334 | BLDA23_03990 | CK945_RS04420 | ACH97_205390 |
| diacylglycerol kinase | TRNA_RS25195 | BL14DL4_03335 | BLDA23_03995 | CK945_RS04425 | ACH97_205395 |
| 23S rRNA (uracil(1939)-C(5))-methyltransferase RlmD | TRNA_RS25200 | BL14DL4_03336 | BLDA23_04000 | CK945_RS04430 | ACH97_205400 |
| tetratricopeptide repeat protein | TRNA_RS25245 | BL14DL4_03343 | BLDA23_04080 | CK945_RS04465 | ACH97_205440 |
| hypothetical protein | TRNA_RS25250 | BL14DL4_03344 | BLDA23_04085 | CK945_RS04470 | ACH97_205445 |
| class I SAM-dependent methyltransferase | TRNA_RS25255 | BL14DL4_03345 | BLDA23_04090 | CK945_RS04475 | ACH97_205450 |
| YebC/PmpR family DNA-binding transcriptional regulator | TRNA_RS25260 | BL14DL4_03346 | BLDA23_04100 | CK945_RS04505 | ACH97_205480 |
| transcriptional regulator | TRNA_RS25265 | BL14DL4_03347 | BLDA23_04105 | CK945_RS04510 | ACH97_205485 |
| DUF2178 domain-containing protein | TRNA_RS25270 | BL14DL4_03348 | BLDA23_04110 | CK945_RS04515 | ACH97_205490 |
| aldehyde dehydrogenase family protein | TRNA_RS25275 | BL14DL4_03349 | BLDA23_04115 | CK945_RS04520 | ACH97_205510 |
| methyl-accepting chemotaxis protein | TRNA_RS25280 | BL14DL4_03350 | BLDA23_04120 | CK945_RS04525 | ACH97_205515 |
| ABC transporter ATP-binding protein | TRNA_RS25290 | BL14DL4_03352 | BLDA23_04130 | CK945_RS04545 | ACH97_205535 |
| GTP cyclohydrolase I FolE2 | TRNA_RS25300 | BL14DL4_03353 | BLDA23_04135 | CK945_RS04570 | ACH97_205550 |
| GTP-binding protein | TRNA_RS25320 | BL14DL4_03356 | BLDA23_04150 | CK945_RS04585 | ACH97_205565 |
| adenosylmethionine--8-amino-7-oxononanoate transaminase | TRNA_RS25330 | BL14DL4_03358 | BLDA23_04160 | CK945_RS04595 | ACH97_205575 |
| 8-amino-7-oxononanoate synthase | TRNA_RS25335 | BL14DL4_03359 | BLDA23_04165 | CK945_RS04600 | ACH97_205580 |
| ATP-dependent dethiobiotin synthetase BioD | TRNA_RS25340 | BL14DL4_03360 | BLDA23_04170 | CK945_RS04605 | ACH97_205585 |
| biotin synthase | TRNA_RS25345 | BL14DL4_03361 | BLDA23_04175 | CK945_RS04610 | ACH97_205590 |
| cytochrome P450 | TRNA_RS25350 | BL14DL4_03362 | BLDA23_04180 | CK945_RS04615 | ACH97_205595 |
| cation transporter | TRNA_RS25355 | BL14DL4_03363 | BLDA23_04185 | CK945_RS04620 | ACH97_205600 |
| MerR family transcriptional regulator | TRNA_RS25360 | BL14DL4_03364 | BLDA23_04190 | CK945_RS04625 | ACH97_205605 |
| MFS transporter | TRNA_RS25365 | BL14DL4_03365 | BLDA23_04195 | CK945_RS04630 | ACH97_205610 |
| ABC transporter ATP-binding protein | TRNA_RS25370 | BL14DL4_03366 | BLDA23_04200 | CK945_RS04635 | ACH97_205620 |
| DEAD/DEAH box helicase | TRNA_RS25375 | BL14DL4_03367 | BLDA23_04205 | CK945_RS04640 | ACH97_205625 |
| NADP-dependent oxidoreductase | TRNA_RS25380 | BL14DL4_03368 | BLDA23_04210 | CK945_RS04645 | ACH97_205630 |
| DUF3212 domain-containing protein | TRNA_RS25385 | BL14DL4_03369 | BLDA23_04215 | CK945_RS04650 | ACH97_205635 |
| general stress protein | TRNA_RS25390 | BL14DL4_03370 | BLDA23_04220 | CK945_RS04655 | ACH97_205640 |
| anion permease | TRNA_RS25395 | BL14DL4_03371 | BLDA23_04225 | CK945_RS04660 | ACH97_205645 |
| MBL fold metallo-hydrolase | TRNA_RS25405 | BL14DL4_03373 | BLDA23_04235 | CK945_RS04685 | ACH97_205670 |
| MBL fold metallo-hydrolase | TRNA_RS25410 | BL14DL4_03374 | BLDA23_04240 | CK945_RS04690 | ACH97_205675 |
| peptidase M14 | TRNA_RS25415 | BL14DL4_03375 | BLDA23_04245 | CK945_RS04695 | ACH97_205680 |
| nitric oxide synthase oxygenase | TRNA_RS25420 | BL14DL4_03376 | BLDA23_04250 | CK945_RS04700 | ACH97_205685 |
| DUF1648 domain-containing protein | TRNA_RS25425 | BL14DL4_03377 | BLDA23_04255 | CK945_RS04710 | ACH97_205695 |
| ArsR family transcriptional regulator | TRNA_RS25430 | BL14DL4_03378 | BLDA23_04260 | CK945_RS04715 | ACH97_205700 |
| acylphosphatase | TRNA_RS25435 | BL14DL4_03379 | BLDA23_04265 | CK945_RS04720 | ACH97_205705 |
| MOSC domain-containing protein | TRNA_RS25440 | BL14DL4_03380 | BLDA23_04270 | CK945_RS04725 | ACH97_205710 |
| type I methionyl aminopeptidase | TRNA_RS25445 | BL14DL4_03383 | BLDA23_04280 | CK945_RS04740 | ACH97_205715 |
| LTA synthase family protein | TRNA_RS25450 | BL14DL4_03384 | BLDA23_04285 | CK945_RS04745 | ACH97_205720 |
| DUF1992 domain-containing protein | TRNA_RS25455 | BL14DL4_03385 | BLDA23_04290 | CK945_RS04750 | ACH97_205725 |
| PTS trehalose transporter subunit IIBC | TRNA_RS25465 | BL14DL4_03387 | BLDA23_04300 | CK945_RS04760 | ACH97_205735 |
| alpha,alpha-phosphotrehalase | TRNA_RS25470 | BL14DL4_03388 | BLDA23_04305 | CK945_RS04765 | ACH97_205740 |
| trehalose operon repressor | TRNA_RS25475 | BL14DL4_03389 | BLDA23_04310 | CK945_RS04770 | ACH97_205745 |
| UDP-3-O-(3-hydroxymyristoyl)glucosamine N-acyltransferase | TRNA_RS25480 | BL14DL4_03390 | BLDA23_04315 | CK945_RS04775 | ACH97_205750 |
| DegT/DnrJ/EryC1/StrS family aminotransferase | TRNA_RS25500 | BL14DL4_03394 | BLDA23_04340 | CK945_RS04795 | ACH97_211260 |
| transferase | TRNA_RS25505 | BL14DL4_03395 | BLDA23_04345 | CK945_RS04800 | ACH97_211265 |
| glycosyltransferase family 1 protein | TRNA_RS25515 | BL14DL4_03397 | BLDA23_04355 | CK945_RS04810 | ACH97_211275 |
| glycosyltransferase | TRNA_RS25520 | BL14DL4_03398 | BLDA23_04360 | CK945_RS04815 | ACH97_211280 |
| glycosyltransferase | TRNA_RS25525 | BL14DL4_03399 | BLDA23_04365 | CK945_RS04820 | ACH97_211285 |
| DegT/DnrJ/EryC1/StrS family aminotransferase | TRNA_RS25530 | BL14DL4_03400 | BLDA23_04370 | CK945_RS04825 | ACH97_211290 |
| gfo/Idh/MocA family oxidoreductase | TRNA_RS25535 | BL14DL4_03401 | BLDA23_04375 | CK945_RS04830 | ACH97_211295 |
| nucleotide sugar dehydrogenase | TRNA_RS25540 | BL14DL4_03402 | BLDA23_04380 | CK945_RS04835 | ACH97_211300 |
| alpha/beta hydrolase | TRNA_RS25545 | BL14DL4_03403 | BLDA23_04385 | CK945_RS04840 | ACH97_211305 |
| transcriptional regulator | TRNA_RS25550 | BL14DL4_03404 | BLDA23_04390 | CK945_RS04845 | ACH97_211310 |
| NAD(P)H-dependent oxidoreductase | TRNA_RS25555 | BL14DL4_03405 | BLDA23_04395 | CK945_RS04850 | ACH97_211315 |
| multifunctional 2,3-cyclic-nucleotide 2-phosphodiesterase/3-nucleotidase/5-nucleotidase | TRNA_RS25560 | BL14DL4_03406 | BLDA23_04400 | CK945_RS04855 | ACH97_211320 |
| type 1 glutamine amidotransferase | TRNA_RS25565 | BL14DL4_03407 | BLDA23_04405 | CK945_RS04860 | ACH97_211325 |
| DUF1128 domain-containing protein | TRNA_RS25570 | BL14DL4_03408 | BLDA23_04410 | CK945_RS04865 | ACH97_211330 |
| sodium:alanine symporter family protein | TRNA_RS25575 | BL14DL4_03409 | BLDA23_04415 | CK945_RS04870 | ACH97_211335 |
| low molecular weight phosphotyrosine protein phosphatase | TRNA_RS25580 | BL14DL4_03410 | BLDA23_04420 | CK945_RS04875 | ACH97_211340 |
| hypothetical protein | TRNA_RS25585 | BL14DL4_03411 | BLDA23_04425 | CK945_RS04880 | ACH97_211345 |
| YihY family inner membrane protein | TRNA_RS25590 | BL14DL4_03412 | BLDA23_04430 | CK945_RS04885 | ACH97_211350 |
| MFS transporter | TRNA_RS25595 | BL14DL4_03414 | BLDA23_04435 | CK945_RS04890 | ACH97_211355 |
| OsmC family peroxiredoxin | TRNA_RS25600 | BL14DL4_03415 | BLDA23_04440 | CK945_RS04895 | ACH97_211360 |
| calcium/proton exchanger | TRNA_RS25605 | BL14DL4_03416 | BLDA23_04445 | CK945_RS04900 | ACH97_211365 |
| hypothetical protein | TRNA_RS25610 | BL14DL4_03417 | BLDA23_04450 | CK945_RS04905 | ACH97_211370 |
| radical SAM/CxCxxxxC motif protein YfkAB | TRNA_RS25615 | BL14DL4_03418 | BLDA23_04455 | CK945_RS04910 | ACH97_211375 |
| hypothetical protein | TRNA_RS25620 | BL14DL4_03419 | BLDA23_04460 | CK945_RS04915 | ACH97_211380 |
| glycerol dehydrogenase | TRNA_RS25630 | BL14DL4_03421 | BLDA23_04470 | CK945_RS04925 | ACH97_211390 |
| magnesium and cobalt transport protein CorA | TRNA_RS25640 | BL14DL4_03423 | BLDA23_04480 | CK945_RS04935 | ACH97_211400 |
| DNA-3-methyladenine glycosylase 2 family protein | TRNA_RS25645 | BL14DL4_03424 | BLDA23_04485 | CK945_RS04940 | ACH97_211405 |
| 23S rRNA (uracil(1939)-C(5))-methyltransferase RlmD | TRNA_RS25650 | BL14DL4_03425 | BLDA23_04490 | CK945_RS04945 | ACH97_211410 |
| hypothetical protein | TRNA_RS25655 | BL14DL4_03426 | BLDA23_04495 | CK945_RS04950 | ACH97_211415 |
| hypothetical protein | TRNA_RS25665 | BL14DL4_03428 | BLDA23_04510 | CK945_RS04965 | ACH97_211430 |
| TetR/AcrR family transcriptional regulator | TRNA_RS25670 | BL14DL4_03429 | BLDA23_04515 | CK945_RS04970 | ACH97_211435 |
| UDP-glucosyltransferase | TRNA_RS25675 | BL14DL4_03430 | BLDA23_04520 | CK945_RS04975 | ACH97_211440 |
| DUF2680 domain-containing protein | TRNA_RS25680 | BL14DL4_03431 | BLDA23_04525 | CK945_RS04980 | ACH97_211445 |
| MFS transporter | TRNA_RS25685 | BL14DL4_03432 | BLDA23_04530 | CK945_RS04985 | ACH97_211450 |
| PTS sugar transporter | TRNA_RS25690 | BL14DL4_03433 | BLDA23_04535 | CK945_RS04995 | ACH97_211460 |
| PRD domain-containing protein | TRNA_RS25695 | BL14DL4_03434 | BLDA23_04540 | CK945_RS05000 | ACH97_211465 |
| carbonic anhydrase | TRNA_RS25700 | BL14DL4_03435 | BLDA23_04545 | CK945_RS05005 | ACH97_211470 |
| DUF2309 family protein | TRNA_RS25705 | BL14DL4_03436 | BLDA23_04550 | CK945_RS05010 | ACH97_211475 |
| NADH dehydrogenase subunit 5 | TRNA_RS25710 | BL14DL4_03437 | BLDA23_04555 | CK945_RS05015 | ACH97_211480 |
| DUF2294 domain-containing protein | TRNA_RS25715 | BL14DL4_03438 | BLDA23_04560 | CK945_RS05020 | ACH97_211485 |
| DJ-1 family protein | TRNA_RS25720 | BL14DL4_03439 | BLDA23_04565 | CK945_RS05025 | ACH97_211490 |
| thiamine pyrophosphate-dependent dehydrogenase E1 component subunit alpha | TRNA_RS25725 | BL14DL4_03440 | BLDA23_04570 | CK945_RS05030 | ACH97_211500 |
| alpha-ketoacid dehydrogenase subunit beta | TRNA_RS25730 | BL14DL4_03441 | BLDA23_04575 | CK945_RS05035 | ACH97_211505 |
| 2-oxo acid dehydrogenase subunit E2 | TRNA_RS25735 | BL14DL4_03442 | BLDA23_04580 | CK945_RS05040 | ACH97_211510 |
| dihydrolipoyl dehydrogenase | TRNA_RS25740 | BL14DL4_03443 | BLDA23_04585 | CK945_RS05045 | ACH97_211515 |
| YnfA family protein | TRNA_RS25750 | BL14DL4_03445 | BLDA23_04595 | CK945_RS05055 | ACH97_211525 |
| 6-phospho-alpha-glucosidase | TRNA_RS25755 | BL14DL4_03446 | BLDA23_04600 | CK945_RS05060 | ACH97_211530 |
| MurR/RpiR family transcriptional regulator | TRNA_RS25760 | BL14DL4_03447 | BLDA23_04605 | CK945_RS05065 | ACH97_211535 |
| PTS alpha-glucoside transporter subunit IIBC | TRNA_RS25765 | BL14DL4_03448 | BLDA23_04610 | CK945_RS05070 | ACH97_211540 |
| class I SAM-dependent methyltransferase | TRNA_RS25770 | BL14DL4_03449 | BLDA23_04615 | CK945_RS05075 | ACH97_211545 |
| glycosyl transferase family 1 | TRNA_RS25775 | BL14DL4_03450 | BLDA23_04620 | CK945_RS05080 | ACH97_211550 |
| HxlR family transcriptional regulator | TRNA_RS25780 | BL14DL4_03451 | BLDA23_04625 | CK945_RS05085 | ACH97_211555 |
| DoxX family protein | TRNA_RS25785 | BL14DL4_03452 | BLDA23_04630 | CK945_RS05090 | ACH97_211560 |
| VOC family protein | TRNA_RS25790 | BL14DL4_03453 | BLDA23_04635 | CK945_RS05095 | ACH97_211565 |
| glycoside hydrolase | TRNA_RS25795 | BL14DL4_03454 | BLDA23_04640 | CK945_RS05100 | ACH97_211570 |
| glycoside hydrolase family 43 protein | TRNA_RS25800 | BL14DL4_03455 | BLDA23_04645 | CK945_RS05105 | ACH97_211575 |
| AraC family transcriptional regulator | TRNA_RS25805 | BL14DL4_03456 | BLDA23_04650 | CK945_RS05110 | ACH97_211580 |
| NUDIX domain-containing protein | TRNA_RS25820 | BL14DL4_03459 | BLDA23_04665 | CK945_RS05125 | ACH97_211595 |
| putative metal-dependent hydrolase | TRNA_RS25825 | BL14DL4_03460 | BLDA23_04670 | CK945_RS05130 | ACH97_211600 |
| bifunctional lysylphosphatidylglycerol flippase/synthetase MprF | TRNA_RS25830 | BL14DL4_03461 | BLDA23_04675 | CK945_RS05135 | ACH97_211605 |
| PhzF family phenazine biosynthesis isomerase | TRNA_RS25835 | BL14DL4_03463 | BLDA23_04680 | CK945_RS05140 | ACH97_211610 |
| nitroreductase | TRNA_RS25840 | BL14DL4_03464 | BLDA23_04685 | CK945_RS05145 | ACH97_211615 |
| YfhD family protein | TRNA_RS25845 | BL14DL4_03465 | BLDA23_04690 | CK945_RS05150 | ACH97_211620 |
| N-acetyltransferase | TRNA_RS25850 | BL14DL4_03467 | BLDA23_04700 | CK945_RS05160 | ACH97_211625 |
| TIGR01777 family protein | TRNA_RS25855 | BL14DL4_03468 | BLDA23_04705 | CK945_RS05165 | ACH97_211630 |
| recombination regulator RecX | TRNA_RS25860 | BL14DL4_03469 | BLDA23_04710 | CK945_RS05170 | ACH97_211635 |
| DUF1811 family protein | TRNA_RS25865 | BL14DL4_03470 | BLDA23_04715 | CK945_RS05175 | ACH97_211640 |
| small, acid-soluble spore protein K | TRNA_RS25870 | BL14DL4_03472 | BLDA23_04725 | CK945_RS05185 | ACH97_211645 |
| hypothetical protein | TRNA_RS25875 | BL14DL4_03473 | BLDA23_04730 | CK945_RS05190 | ACH97_211650 |
| glycosyltransferase | TRNA_RS25885 | BL14DL4_03474 | BLDA23_04740 | CK945_RS05195 | ACH97_211655 |
| GtrA family protein | TRNA_RS25890 | BL14DL4_03475 | BLDA23_04745 | CK945_RS05200 | ACH97_211660 |
| hypothetical protein | TRNA_RS25895 | BL14DL4_03476 | BLDA23_04750 | CK945_RS05205 | ACH97_211665 |
| metal-dependent hydrolase | TRNA_RS25900 | BL14DL4_03477 | BLDA23_04755 | CK945_RS05210 | ACH97_211670 |
| A/G-specific adenine glycosylase | TRNA_RS25905 | BL14DL4_03478 | BLDA23_04760 | CK945_RS05215 | ACH97_211675 |
| hypothetical protein | TRNA_RS25910 | BL14DL4_03479 | BLDA23_04765 | CK945_RS05220 | ACH97_211680 |
| enoyl-[acyl-carrier-protein] reductase FabL | TRNA_RS25915 | BL14DL4_03480 | BLDA23_04770 | CK945_RS05225 | ACH97_211685 |
| gamma-type small acid-soluble spore protein | TRNA_RS25920 | BL14DL4_03481 | BLDA23_04775 | CK945_RS05230 | ACH97_211690 |
| hypothetical protein | TRNA_RS25925 | BL14DL4_03482 | BLDA23_04780 | CK945_RS05235 | ACH97_211695 |
| DUF402 domain-containing protein | TRNA_RS25930 | BL14DL4_03483 | BLDA23_04785 | CK945_RS05240 | ACH97_211700 |
| ABC transporter ATP-binding protein | TRNA_RS25935 | BL14DL4_03484 | BLDA23_04790 | CK945_RS05245 | ACH97_211705 |
| ABC transporter ATP-binding protein | TRNA_RS25940 | BL14DL4_03485 | BLDA23_04795 | CK945_RS05250 | ACH97_211710 |
| ABC transporter ATP-binding protein | TRNA_RS25945 | BL14DL4_03486 | BLDA23_04800 | CK945_RS05255 | ACH97_211715 |
| ABC transporter substrate-binding protein | TRNA_RS25950 | BL14DL4_03487 | BLDA23_04805 | CK945_RS05260 | ACH97_211720 |
| ABC transporter permease | TRNA_RS25955 | BL14DL4_03488 | BLDA23_04810 | CK945_RS05265 | ACH97_211725 |
| ABC transporter permease | TRNA_RS25960 | BL14DL4_03489 | BLDA23_04815 | CK945_RS05270 | ACH97_211730 |
| aromatic acid exporter family protein | TRNA_RS25965 | BL14DL4_03490 | BLDA23_04820 | CK945_RS05275 | ACH97_211735 |
| glutamate-1-semialdehyde 2,1-aminomutase | TRNA_RS25970 | BL14DL4_03491 | BLDA23_04825 | CK945_RS05280 | ACH97_211740 |
| thioredoxin-dependent thiol peroxidase | TRNA_RS25975 | BL14DL4_03492 | BLDA23_04830 | CK945_RS05285 | ACH97_211750 |
| transcriptional repressor | TRNA_RS25980 | BL14DL4_03493 | BLDA23_04835 | CK945_RS05290 | ACH97_211755 |
| hypothetical protein | TRNA_RS25985 | BL14DL4_03494 | BLDA23_04840 | CK945_RS05295 | ACH97_211760 |
| hypothetical protein | TRNA_RS25990 | BL14DL4_03495 | BLDA23_04845 | CK945_RS05300 | ACH97_211765 |
| TGS domain-containing protein | TRNA_RS26145 | BL14DL4_03522 | BLDA23_05460 | CK945_RS05690 | ACH97_204625 |
| type I 3-dehydroquinate dehydratase | TRNA_RS26155 | BL14DL4_03523 | BLDA23_05470 | CK945_RS05710 | ACH97_204605 |
| phosphomethylpyrimidine synthase ThiC | TRNA_RS26160 | BL14DL4_03524 | BLDA23_05475 | CK945_RS05715 | ACH97_204600 |
| ABC transporter ATP-binding protein | TRNA_RS26165 | BL14DL4_03525 | BLDA23_05480 | CK945_RS05720 | ACH97_204595 |
| aliphatic sulfonate ABC transporter substrate-binding protein | TRNA_RS26170 | BL14DL4_03526 | BLDA23_05485 | CK945_RS05725 | ACH97_204590 |
| ABC transporter permease | TRNA_RS26175 | BL14DL4_03527 | BLDA23_05490 | CK945_RS05730 | ACH97_204585 |
| alkanesulfonate monooxygenase, FMNH(2)-dependent | TRNA_RS26180 | BL14DL4_03528 | BLDA23_05495 | CK945_RS05735 | ACH97_204580 |
| hypothetical protein | TRNA_RS26185 | BL14DL4_03529 | BLDA23_05500 | CK945_RS05740 | ACH97_204575 |
| transcriptional regulator | TRNA_RS26190 | BL14DL4_03530 | BLDA23_05505 | CK945_RS05745 | ACH97_204570 |
| hypothetical protein | TRNA_RS26195 | BL14DL4_03531 | BLDA23_05510 | CK945_RS05750 | ACH97_204565 |
| ABC transporter ATP-binding protein | TRNA_RS26200 | BL14DL4_03532 | BLDA23_05515 | CK945_RS05755 | ACH97_204560 |
| hypothetical protein | TRNA_RS26205 | BL14DL4_03533 | BLDA23_05520 | CK945_RS05760 | ACH97_204555 |
| PqqD family protein | TRNA_RS26210 | BL14DL4_03534 | BLDA23_05525 | CK945_RS05765 | ACH97_204550 |
| hypothetical protein | TRNA_RS26215 | BL14DL4_03535 | BLDA23_05530 | CK945_RS05770 | ACH97_204545 |
| 30S ribosomal protein S14 | TRNA_RS26220 | BL14DL4_03536 | BLDA23_05535 | CK945_RS05775 | ACH97_204540 |
| hypothetical protein | TRNA_RS26230 | BL14DL4_03538 | BLDA23_05545 | CK945_RS05785 | ACH97_204530 |
| tRNA epoxyqueuosine(34) reductase QueG | TRNA_RS26235 | BL14DL4_03539 | BLDA23_05550 | CK945_RS05790 | ACH97_204525 |
| hypothetical protein | TRNA_RS26240 | BL14DL4_03540 | BLDA23_05555 | CK945_RS05795 | ACH97_204520 |
| tRNA (uridine(34)/cytosine(34)/5- carboxymethylaminomethyluridine(34)-2-O)- methyltransferase TrmL | TRNA_RS26245 | BL14DL4_03541 | BLDA23_05560 | CK945_RS05800 | ACH97_204515 |
| DUF4004 family protein | TRNA_RS26250 | BL14DL4_03542 | BLDA23_05565 | CK945_RS05805 | ACH97_204510 |
| hypothetical protein | TRNA_RS26255 | BL14DL4_03543 | BLDA23_05570 | CK945_RS05810 | ACH97_204505 |
| hypothetical protein | TRNA_RS26260 | BL14DL4_03544 | BLDA23_05575 | CK945_RS05815 | ACH97_204500 |
| protein PrkA | TRNA_RS26265 | BL14DL4_03545 | BLDA23_05580 | CK945_RS05820 | ACH97_204490 |
| HlyD family secretion protein | TRNA_RS26275 | BL14DL4_03547 | BLDA23_06105 | CK945_RS05830 | ACH97_204480 |
| DHA2 family efflux MFS transporter permease subunit | TRNA_RS26280 | BL14DL4_03548 | BLDA23_06110 | CK945_RS05835 | ACH97_204475 |
| hypothetical protein | TRNA_RS26285 | BL14DL4_03549 | BLDA23_06115 | CK945_RS05840 | ACH97_204470 |
| hypothetical protein | TRNA_RS26290 | BL14DL4_03550 | BLDA23_06120 | CK945_RS05845 | ACH97_204465 |
| ABC transporter ATP-binding protein | TRNA_RS26310 | BL14DL4_03552 | BLDA23_06140 | CK945_RS05855 | ACH97_204455 |
| ABC transporter permease | TRNA_RS26315 | BL14DL4_03553 | BLDA23_06145 | CK945_RS05860 | ACH97_204450 |
| cold-shock protein | TRNA_RS26320 | BL14DL4_03554 | BLDA23_06155 | CK945_RS05865 | ACH97_204445 |
| methionine import ATP-binding protein MetN 1 | TRNA_RS26325 | BL14DL4_03555 | BLDA23_06160 | CK945_RS05870 | ACH97_204440 |
| ABC transporter permease | TRNA_RS26330 | BL14DL4_03556 | BLDA23_06165 | CK945_RS05875 | ACH97_204435 |
| methionine ABC transporter substrate-binding protein | TRNA_RS26335 | BL14DL4_03557 | BLDA23_06170 | CK945_RS05880 | ACH97_204430 |
| L-cystine transporter | TRNA_RS26340 | BL14DL4_03558 | BLDA23_06175 | CK945_RS05885 | ACH97_204425 |
| hypothetical protein | TRNA_RS26345 | BL14DL4_03559 | BLDA23_06180 | CK945_RS05890 | ACH97_204420 |
| YhcN/YlaJ family sporulation lipoprotein | TRNA_RS26355 | BL14DL4_03561 | BLDA23_06190 | CK945_RS05900 | ACH97_204410 |
| spore coat protein | TRNA_RS26365 | BL14DL4_03563 | BLDA23_06200 | CK945_RS05910 | ACH97_204400 |
| PAS domain-containing sensor histidine kinase | TRNA_RS26370 | BL14DL4_03564 | BLDA23_06205 | CK945_RS05915 | ACH97_204390 |
| endonuclease | TRNA_RS26375 | BL14DL4_03565 | BLDA23_06210 | CK945_RS05920 | ACH97_204385 |
| class D sortase | TRNA_RS26380 | BL14DL4_03566 | BLDA23_06215 | CK945_RS05925 | ACH97_204380 |
| RluA family pseudouridine synthase | TRNA_RS26385 | BL14DL4_03567 | BLDA23_06220 | CK945_RS05930 | ACH97_204375 |
| hypothetical protein | TRNA_RS26390 | BL14DL4_03568 | BLDA23_06225 | CK945_RS05935 | ACH97_204370 |
| CBS domain-containing protein | TRNA_RS26395 | BL14DL4_03569 | BLDA23_06230 | CK945_RS05940 | ACH97_204365 |
| HAD family hydrolase | TRNA_RS26400 | BL14DL4_03570 | BLDA23_06235 | CK945_RS05945 | ACH97_204360 |
| ABC transporter ATP-binding protein | TRNA_RS26410 | BL14DL4_03573 | BLDA23_06245 | CK945_RS05955 | ACH97_204350 |
| formate/nitrite transporter family protein | TRNA_RS26415 | BL14DL4_03574 | BLDA23_06255 | CK945_RS05960 | ACH97_204345 |
| AEC family transporter | TRNA_RS26420 | BL14DL4_03575 | BLDA23_06260 | CK945_RS05970 | ACH97_204335 |
| alcohol dehydrogenase | TRNA_RS26425 | BL14DL4_03576 | BLDA23_06265 | CK945_RS05975 | ACH97_204330 |
| glycerol-3-phosphate responsive antiterminator | TRNA_RS26430 | BL14DL4_03577 | BLDA23_06270 | CK945_RS05980 | ACH97_204325 |
| aquaporin family protein | TRNA_RS26435 | BL14DL4_03578 | BLDA23_06275 | CK945_RS05985 | ACH97_204320 |
| glycerol kinase | TRNA_RS26440 | BL14DL4_03579 | BLDA23_06280 | CK945_RS05990 | ACH97_204315 |
| glycerol-3-phosphate dehydrogenase/oxidase | TRNA_RS26445 | BL14DL4_03581 | BLDA23_06285 | CK945_RS05995 | ACH97_204310 |
| phospho-sugar mutase | TRNA_RS26450 | BL14DL4_03582 | BLDA23_06290 | CK945_RS06000 | ACH97_204305 |
| hypothetical protein | TRNA_RS26455 | BL14DL4_03583 | BLDA23_06295 | CK945_RS06005 | ACH97_204300 |
| transcriptional regulator | TRNA_RS26460 | BL14DL4_03584 | BLDA23_06300 | CK945_RS06010 | ACH97_204295 |
| GAF domain-containing protein | TRNA_RS26465 | BL14DL4_03585 | BLDA23_06305 | CK945_RS06015 | ACH97_204290 |
| DNA-binding response regulator | TRNA_RS26470 | BL14DL4_03586 | BLDA23_06310 | CK945_RS06020 | ACH97_204285 |
| NAD(P)H-dependent oxidoreductase | TRNA_RS26475 | BL14DL4_03587 | BLDA23_06315 | CK945_RS06025 | ACH97_204280 |
| hypothetical protein | TRNA_RS26480 | BL14DL4_03588 | BLDA23_06320 | CK945_RS06030 | ACH97_204275 |
| DUF3889 domain-containing protein | TRNA_RS26485 | BL14DL4_03589 | BLDA23_06325 | CK945_RS06035 | ACH97_204270 |
| transcriptional regulator | TRNA_RS26490 | BL14DL4_03590 | BLDA23_06330 | CK945_RS06040 | ACH97_204265 |
| NO-inducible flavohemoprotein | TRNA_RS26495 | BL14DL4_03591 | BLDA23_06335 | CK945_RS06045 | ACH97_219370 |
| SpoVR family protein | TRNA_RS26500 | BL14DL4_03592 | BLDA23_06340 | CK945_RS06050 | ACH97_204255 |
| peptidoglycan endopeptidase | TRNA_RS26505 | BL14DL4_03593 | BLDA23_06345 | CK945_RS06055 | ACH97_204250 |
| LysR family transcriptional regulator | TRNA_RS26510 | BL14DL4_03594 | BLDA23_06350 | CK945_RS06060 | ACH97_204245 |
| citrate synthase/methylcitrate synthase | TRNA_RS26515 | BL14DL4_03595 | BLDA23_06355 | CK945_RS06065 | ACH97_204240 |
| NAD(P)-dependent oxidoreductase | TRNA_RS26525 | BL14DL4_03597 | BLDA23_06365 | CK945_RS06075 | ACH97_204230 |
| sodium-dependent transporter | TRNA_RS26530 | BL14DL4_03598 | BLDA23_06370 | CK945_RS06080 | ACH97_204225 |
| aspartate aminotransferase family protein | TRNA_RS26535 | BL14DL4_03599 | BLDA23_06375 | CK945_RS06085 | ACH97_204220 |
| AraC family transcriptional regulator | TRNA_RS26540 | BL14DL4_03600 | BLDA23_06380 | CK945_RS06110 | ACH97_204195 |
| GNAT family N-acetyltransferase | TRNA_RS26550 | BL14DL4_03602 | BLDA23_06390 | CK945_RS06120 | ACH97_204185 |
| hypothetical protein | TRNA_RS26555 | BL14DL4_03603 | BLDA23_06395 | CK945_RS06125 | ACH97_204180 |
| anti-sigma-M factor | TRNA_RS26560 | BL14DL4_03604 | BLDA23_06400 | CK945_RS06130 | ACH97_204175 |
| sigma-70 family RNA polymerase sigma factor | TRNA_RS26565 | BL14DL4_03605 | BLDA23_06405 | CK945_RS06135 | ACH97_204170 |
| cupin domain-containing protein | TRNA_RS26570 | BL14DL4_03606 | BLDA23_06410 | CK945_RS06140 | ACH97_204165 |
| 1-acyl-sn-glycerol-3-phosphate acyltransferase | TRNA_RS26580 | BL14DL4_03608 | BLDA23_06420 | CK945_RS06150 | ACH97_204155 |
| DUF805 domain-containing protein | TRNA_RS26585 | BL14DL4_03610 | BLDA23_06425 | CK945_RS06155 | ACH97_204150 |
| acyl-CoA thioesterase | TRNA_RS26590 | BL14DL4_03611 | BLDA23_06430 | CK945_RS06160 | ACH97_204145 |
| TetR/AcrR family transcriptional regulator | TRNA_RS26595 | BL14DL4_03612 | BLDA23_06435 | CK945_RS06165 | ACH97_204140 |
| HlyC/CorC family transporter | TRNA_RS26600 | BL14DL4_03613 | BLDA23_06440 | CK945_RS06170 | ACH97_204135 |
| MerR family transcriptional regulator | TRNA_RS26605 | BL14DL4_03614 | BLDA23_06445 | CK945_RS06175 | ACH97_204130 |
| HlyC/CorC family transporter | TRNA_RS26610 | BL14DL4_03616 | BLDA23_06450 | CK945_RS06180 | ACH97_204125 |
| hypothetical protein | TRNA_RS26615 | BL14DL4_03617 | BLDA23_06455 | CK945_RS06185 | ACH97_204120 |
| hypothetical protein | TRNA_RS26620 | BL14DL4_03618 | BLDA23_06460 | CK945_RS06190 | ACH97_204115 |
| N-acetylmuramoyl-L-alanine amidase | TRNA_RS26625 | BL14DL4_03619 | BLDA23_06465 | CK945_RS06195 | ACH97_204110 |
| fluoride efflux transporter CrcB | TRNA_RS26630 | BL14DL4_03620 | BLDA23_06470 | CK945_RS06200 | ACH97_204105 |
| chromosome condensation protein CrcB | TRNA_RS26635 | BL14DL4_03621 | BLDA23_06475 | CK945_RS06205 | ACH97_204100 |
| glycerophosphoryl diester phosphodiesterase | TRNA_RS26640 | BL14DL4_03622 | BLDA23_06480 | CK945_RS06210 | ACH97_204095 |
| mechanosensitive ion channel protein | TRNA_RS26645 | BL14DL4_03624 | BLDA23_06490 | CK945_RS06220 | ACH97_204090 |
| NAD-dependent protein deacylase | TRNA_RS26650 | BL14DL4_03625 | BLDA23_06495 | CK945_RS06225 | ACH97_204085 |
| GNAT family N-acetyltransferase | TRNA_RS26655 | BL14DL4_03626 | BLDA23_06505 | CK945_RS06230 | ACH97_204080 |
| polysaccharide deacetylase | TRNA_RS26660 | BL14DL4_03627 | BLDA23_06510 | CK945_RS06235 | ACH97_204075 |
| D-amino-acid transaminase | TRNA_RS26665 | BL14DL4_03628 | BLDA23_06515 | CK945_RS06240 | ACH97_204070 |
| Na+/H+ antiporter NhaC | TRNA_RS26670 | BL14DL4_03629 | BLDA23_06520 | CK945_RS06245 | ACH97_204065 |
| aldose 1-epimerase | TRNA_RS26675 | BL14DL4_03630 | BLDA23_06525 | CK945_RS06250 | ACH97_204060 |
| DUF4944 domain-containing protein | TRNA_RS26680 | BL14DL4_03631 | BLDA23_06530 | CK945_RS06255 | ACH97_204055 |
| hypothetical protein | TRNA_RS26685 | BL14DL4_03632 | BLDA23_06535 | CK945_RS06260 | ACH97_204050 |
| putative thiazole-containing bacteriocin maturation protein | TRNA_RS26690 | BL14DL4_03633 | BLDA23_06540 | CK945_RS06265 | ACH97_204045 |
| universal stress protein | TRNA_RS26695 | BL14DL4_03634 | BLDA23_06550 | CK945_RS06275 | ACH97_204035 |
| multidrug ABC transporter permease/ATP-binding protein | TRNA_RS26700 | BL14DL4_03635 | BLDA23_06555 | CK945_RS06280 | ACH97_204030 |
| ABC transporter ATP-binding protein | TRNA_RS26705 | BL14DL4_03636 | BLDA23_06560 | CK945_RS06285 | ACH97_204025 |
| hypothetical protein | TRNA_RS26710 | BL14DL4_03637 | BLDA23_06565 | CK945_RS06290 | ACH97_204020 |
| small acid-soluble spore protein | TRNA_RS26715 | BL14DL4_03639 | BLDA23_06570 | CK945_RS06295 | ACH97_204015 |
| hypothetical protein | TRNA_RS26720 | BL14DL4_03640 | BLDA23_06575 | CK945_RS06300 | ACH97_204010 |
| sn-glycerol-3-phosphate ABC transporter ATP-binding protein UgpC | TRNA_RS26725 | BL14DL4_03641 | BLDA23_06580 | CK945_RS06305 | ACH97_204005 |
| PucR family transcriptional regulator | TRNA_RS26730 | BL14DL4_03642 | BLDA23_06585 | CK945_RS06310 | ACH97_204000 |
| hypothetical protein | TRNA_RS26735 | BL14DL4_03643 | BLDA23_06590 | CK945_RS06315 | ACH97_203995 |
| YheC/YheD family protein | TRNA_RS26740 | BL14DL4_03644 | BLDA23_06595 | CK945_RS06320 | ACH97_203990 |
| YheC/YheD family protein | TRNA_RS26745 | BL14DL4_03645 | BLDA23_06600 | CK945_RS06325 | ACH97_203985 |
| DUF445 family protein | TRNA_RS26750 | BL14DL4_03646 | BLDA23_06605 | CK945_RS06330 | ACH97_203980 |
| YlbF family regulator | TRNA_RS26755 | BL14DL4_03647 | BLDA23_06610 | CK945_RS06335 | ACH97_203975 |
| coproporphyrinogen III oxidase | TRNA_RS26770 | BL14DL4_03649 | BLDA23_06625 | CK945_RS06350 | ACH97_203960 |
| enoyl-CoA hydratase | TRNA_RS26775 | BL14DL4_03653 | BLDA23_06645 | CK945_RS06370 | ACH97_203940 |
| hypothetical protein | TRNA_RS26785 | BL14DL4_03656 | BLDA23_06655 | CK945_RS06380 | ACH97_203930 |
| hypothetical protein | TRNA_RS26790 | BL14DL4_03657 | BLDA23_06660 | CK945_RS06385 | ACH97_203925 |
| ATP-binding cassette domain-containing protein | TRNA_RS26795 | BL14DL4_03658 | BLDA23_06665 | CK945_RS06390 | ACH97_203920 |
| ABC transporter permease | TRNA_RS26800 | BL14DL4_03659 | BLDA23_06670 | CK945_RS06395 | ACH97_203915 |
| DNA repair exonuclease | TRNA_RS26805 | BL14DL4_03660 | BLDA23_06675 | CK945_RS06400 | ACH97_203910 |
| hypothetical protein | TRNA_RS26810 | BL14DL4_03661 | BLDA23_06680 | CK945_RS06405 | ACH97_203905 |
| 3-5 exoribonuclease yhaM | TRNA_RS26815 | BL14DL4_03662 | BLDA23_06685 | CK945_RS06410 | ACH97_203900 |
| sporulation protein | TRNA_RS26820 | BL14DL4_03663 | BLDA23_06690 | CK945_RS06415 | ACH97_203895 |
| foldase | TRNA_RS26825 | BL14DL4_03664 | BLDA23_06695 | CK945_RS06420 | ACH97_203890 |
| DUF3267 domain-containing protein | TRNA_RS26840 | BL14DL4_03666 | BLDA23_06715 | CK945_RS06440 | ACH97_203880 |
| DUF1878 family protein | TRNA_RS26845 | BL14DL4_03667 | BLDA23_06720 | CK945_RS06445 | ACH97_203875 |
| HTH-type transcriptional regulator Hpr | TRNA_RS26850 | BL14DL4_03668 | BLDA23_06725 | CK945_RS06450 | ACH97_203870 |
| YtxH domain-containing protein | TRNA_RS26855 | BL14DL4_03669 | BLDA23_06730 | CK945_RS06455 | ACH97_203865 |
| hypothetical protein | TRNA_RS26860 | BL14DL4_03670 | BLDA23_06735 | CK945_RS06460 | ACH97_203860 |
| tryptophan transporter | TRNA_RS26865 | BL14DL4_03671 | BLDA23_06740 | CK945_RS06465 | ACH97_203855 |
| 3-phosphoserine/phosphohydroxythreonine transaminase | TRNA_RS26870 | BL14DL4_03672 | BLDA23_06745 | CK945_RS06470 | ACH97_203850 |
| HIT family protein | TRNA_RS26875 | BL14DL4_03673 | BLDA23_06750 | CK945_RS06475 | ACH97_203845 |
| ABC transporter ATP-binding protein | TRNA_RS26885 | BL14DL4_03674 | BLDA23_06760 | CK945_RS06485 | ACH97_203840 |
| ABC transporter permease EscB | TRNA_RS26890 | BL14DL4_03675 | BLDA23_06765 | CK945_RS06490 | ACH97_203835 |
| EcsC family protein | TRNA_RS26895 | BL14DL4_03676 | BLDA23_06770 | CK945_RS06495 | ACH97_203830 |
| amidohydrolase | TRNA_RS26900 | BL14DL4_03677 | BLDA23_06775 | CK945_RS06500 | ACH97_203825 |
| hypothetical protein | TRNA_RS26905 | BL14DL4_03678 | BLDA23_06780 | CK945_RS06505 | ACH97_203820 |
| antibiotic biosynthesis monooxygenase | TRNA_RS26910 | BL14DL4_03679 | BLDA23_06785 | CK945_RS06510 | ACH97_203815 |
| penicillin-binding protein | TRNA_RS26915 | BL14DL4_03680 | BLDA23_06790 | CK945_RS06515 | ACH97_203810 |
| uroporphyrinogen decarboxylase | TRNA_RS26920 | BL14DL4_03681 | BLDA23_06795 | CK945_RS06520 | ACH97_203805 |
| ferrochelatase | TRNA_RS26925 | BL14DL4_03682 | BLDA23_06800 | CK945_RS06525 | ACH97_203800 |
| protoporphyrinogen oxidase | TRNA_RS26930 | BL14DL4_03683 | BLDA23_06805 | CK945_RS06530 | ACH97_203795 |
| TetR/AcrR family transcriptional regulator | TRNA_RS26935 | BL14DL4_03684 | BLDA23_06810 | CK945_RS06535 | ACH97_203790 |
| YhgE/Pip domain-containing protein | TRNA_RS26940 | BL14DL4_03685 | BLDA23_06815 | CK945_RS06540 | ACH97_203785 |
| ketoacyl-ACP synthase III | TRNA_RS26945 | BL14DL4_03686 | BLDA23_06820 | CK945_RS06545 | ACH97_203780 |
| M20/M25/M40 family metallo-hydrolase | TRNA_RS26950 | BL14DL4_03687 | BLDA23_06825 | CK945_RS06550 | ACH97_203775 |
| hypothetical protein | TRNA_RS26955 | BL14DL4_03688 | BLDA23_06830 | CK945_RS06555 | ACH97_203770 |
| proton glutamate symport protein | TRNA_RS26960 | BL14DL4_03689 | BLDA23_06835 | CK945_RS06560 | ACH97_203765 |
| MBL fold metallo-hydrolase | TRNA_RS26965 | BL14DL4_03691 | BLDA23_06845 | CK945_RS06570 | ACH97_203760 |
| lipoate--protein ligase | TRNA_RS26970 | BL14DL4_03692 | BLDA23_06850 | CK945_RS06575 | ACH97_203755 |
| fatty acid--CoA ligase family protein | TRNA_RS26975 | BL14DL4_03693 | BLDA23_06855 | CK945_RS06580 | ACH97_203750 |
| hypothetical protein | TRNA_RS26980 | BL14DL4_03694 | BLDA23_06860 | CK945_RS06585 | ACH97_203745 |
| XRE family transcriptional regulator | TRNA_RS26985 | BL14DL4_03695 | BLDA23_06865 | CK945_RS06590 | ACH97_203740 |
| branched-chain amino acid ABC transporter permease | TRNA_RS26990 | BL14DL4_03696 | BLDA23_06870 | CK945_RS06595 | ACH97_203735 |
| AzlD domain-containing protein | TRNA_RS26995 | BL14DL4_03697 | BLDA23_06875 | CK945_RS06600 | ACH97_203730 |
| M48 family peptidase | TRNA_RS27000 | BL14DL4_03698 | BLDA23_06880 | CK945_RS06605 | ACH97_203725 |
| peptidase S8 | TRNA_RS27005 | BL14DL4_03699 | BLDA23_06885 | CK945_RS06610 | ACH97_203720 |
| EamA family transporter | TRNA_RS27010 | BL14DL4_03700 | BLDA23_06890 | CK945_RS06615 | ACH97_203715 |
| iron-dicitrate ABC transporter substrate-binding protein | TRNA_RS27015 | BL14DL4_03701 | BLDA23_06895 | CK945_RS06620 | ACH97_203710 |
| iron ABC transporter permease | TRNA_RS27020 | BL14DL4_03702 | BLDA23_06900 | CK945_RS06630 | ACH97_203705 |
| iron ABC transporter permease | TRNA_RS27025 | BL14DL4_03703 | BLDA23_06905 | CK945_RS06635 | ACH97_203700 |
| histidine phosphatase family protein | TRNA_RS27030 | BL14DL4_03704 | BLDA23_06910 | CK945_RS06640 | ACH97_203695 |
| globin-coupled sensor protein | TRNA_RS27035 | BL14DL4_03705 | BLDA23_06915 | CK945_RS06645 | ACH97_203690 |
| FAD-dependent oxidoreductase | TRNA_RS27040 | BL14DL4_03706 | BLDA23_06920 | CK945_RS06650 | ACH97_203685 |
| NAD(P)-dependent oxidoreductase | TRNA_RS27045 | BL14DL4_03707 | BLDA23_06925 | CK945_RS06655 | ACH97_203680 |
| IDEAL domain-containing protein | TRNA_RS27050 | BL14DL4_03708 | BLDA23_06930 | CK945_RS06660 | ACH97_203675 |
| competence protein | TRNA_RS27055 | BL14DL4_03709 | BLDA23_06935 | CK945_RS06665 | ACH97_203670 |
| hypothetical protein | TRNA_RS27060 | BL14DL4_03710 | BLDA23_06940 | CK945_RS06670 | ACH97_203665 |
| TVP38/TMEM64 family protein | TRNA_RS27065 | BL14DL4_03711 | BLDA23_06945 | CK945_RS06675 | ACH97_203660 |
| signal peptidase I | TRNA_RS27070 | BL14DL4_03712 | BLDA23_06950 | CK945_RS06680 | ACH97_203655 |
| peptidase S8 | TRNA_RS27075 | BL14DL4_03713 | BLDA23_06955 | CK945_RS06690 | ACH97_203650 |
| MFS transporter | TRNA_RS27080 | BL14DL4_03714 | BLDA23_06960 | CK945_RS06695 | ACH97_203645 |
| allantoinase | TRNA_RS27095 | BL14DL4_03717 | BLDA23_06975 | CK945_RS06705 | ACH97_203635 |
| (S)-ureidoglycine aminohydrolase | TRNA_RS27105 | BL14DL4_03719 | BLDA23_06985 | CK945_RS06715 | ACH97_203625 |
| ureidoglycolate dehydrogenase | TRNA_RS27110 | BL14DL4_03720 | BLDA23_06990 | CK945_RS06720 | ACH97_203620 |
| acyl-CoA synthetase FdrA | TRNA_RS27120 | BL14DL4_03722 | BLDA23_07000 | CK945_RS06730 | ACH97_203610 |
| DUF1116 domain-containing protein | TRNA_RS27125 | BL14DL4_03723 | BLDA23_07005 | CK945_RS06735 | ACH97_203605 |
| DUF2877 domain-containing protein | TRNA_RS27130 | BL14DL4_03724 | BLDA23_07010 | CK945_RS06740 | ACH97_203600 |
| MFS transporter | TRNA_RS27140 | BL14DL4_03726 | BLDA23_07020 | CK945_RS06750 | ACH97_203590 |
| tetratricopeptide repeat protein | TRNA_RS27150 | BL14DL4_03727 | BLDA23_07030 | CK945_RS06760 | ACH97_203580 |
| hypothetical protein | TRNA_RS27155 | BL14DL4_03728 | BLDA23_07035 | CK945_RS06765 | ACH97_203575 |
| AbrB family transcriptional regulator | TRNA_RS27195 | BL14DL4_03736 | BLDA23_07075 | CK945_RS06780 | ACH97_203545 |
| spore coat associated protein CotJA | TRNA_RS27200 | BL14DL4_03737 | BLDA23_07080 | CK945_RS06785 | ACH97_203540 |
| spore coat protein CotJB | TRNA_RS27205 | BL14DL4_03738 | BLDA23_07085 | CK945_RS06790 | ACH97_203535 |
| manganese catalase family protein | TRNA_RS27210 | BL14DL4_03739 | BLDA23_07090 | CK945_RS06795 | ACH97_203530 |
| AMP-binding protein | TRNA_RS27215 | BL14DL4_03740 | BLDA23_07095 | CK945_RS06800 | ACH97_203525 |
| MFS transporter | TRNA_RS27220 | BL14DL4_03741 | BLDA23_07100 | CK945_RS06805 | ACH97_203520 |
| cell wall-binding repeat 2 family protein | TRNA_RS27225 | BL14DL4_03742 | BLDA23_07105 | CK945_RS06815 | ACH97_203510 |
| sensor histidine kinase | TRNA_RS27230 | BL14DL4_03743 | BLDA23_07110 | CK945_RS06820 | ACH97_203505 |
| DNA-binding response regulator | TRNA_RS27235 | BL14DL4_03744 | BLDA23_07115 | CK945_RS06825 | ACH97_203500 |
| molybdate ABC transporter substrate-binding protein | TRNA_RS27245 | BL14DL4_03746 | BLDA23_07125 | CK945_RS06835 | ACH97_203490 |
| molybdate ABC transporter permease subunit | TRNA_RS27250 | BL14DL4_03747 | BLDA23_07130 | CK945_RS06840 | ACH97_203485 |
| helicase-exonuclease AddAB subunit AddA | TRNA_RS27260 | BL14DL4_03749 | BLDA23_07140 | CK945_RS06850 | ACH97_203475 |
| exonuclease SbcCD subunit D | TRNA_RS27265 | BL14DL4_03750 | BLDA23_07145 | CK945_RS06855 | ACH97_203470 |
| SMC family ATPase | TRNA_RS27270 | BL14DL4_03751 | BLDA23_07150 | CK945_RS06860 | ACH97_203465 |
| spore germination protein | TRNA_RS27275 | BL14DL4_03752 | BLDA23_07155 | CK945_RS06865 | ACH97_203460 |
| spore germination protein GerPE | TRNA_RS27280 | BL14DL4_03753 | BLDA23_07160 | CK945_RS06870 | ACH97_203455 |
| germination protein GerPD | TRNA_RS27285 | BL14DL4_03754 | BLDA23_07165 | CK945_RS06875 | ACH97_203450 |
| germination protein GerPC | TRNA_RS27290 | BL14DL4_03755 | BLDA23_07170 | CK945_RS06880 | ACH97_203445 |
| germination protein GerPB | TRNA_RS27295 | BL14DL4_03756 | BLDA23_07175 | CK945_RS06885 | ACH97_203440 |
| spore germination protein | TRNA_RS27300 | BL14DL4_03757 | BLDA23_07180 | CK945_RS06890 | ACH97_203435 |
| DUF3784 domain-containing protein | TRNA_RS27305 | BL14DL4_03758 | BLDA23_07185 | CK945_RS06895 | ACH97_203430 |
| aspartyl-phosphate phosphatase Spo0E family protein | TRNA_RS27310 | BL14DL4_03759 | BLDA23_07190 | CK945_RS06900 | ACH97_203425 |
| spore coat protein | TRNA_RS27315 | BL14DL4_03760 | BLDA23_07195 | CK945_RS06905 | ACH97_203420 |
| hypothetical protein | TRNA_RS27320 | BL14DL4_03761 | BLDA23_07200 | CK945_RS06910 | ACH97_203415 |
| FAA hydrolase family protein | TRNA_RS27325 | BL14DL4_03762 | BLDA23_07205 | CK945_RS06915 | ACH97_203410 |
| ribosome small subunit-dependent GTPase A | TRNA_RS27330 | BL14DL4_03764 | BLDA23_07210 | CK945_RS06925 | ACH97_203400 |
| DUF1516 family protein | TRNA_RS27335 | BL14DL4_03765 | BLDA23_07215 | CK945_RS06930 | ACH97_203395 |
| DUF2777 domain-containing protein | TRNA_RS27340 | BL14DL4_03766 | BLDA23_07220 | CK945_RS06935 | ACH97_203390 |
| asparagine synthase (glutamine-hydrolyzing) | TRNA_RS27345 | BL14DL4_03767 | BLDA23_07225 | CK945_RS06940 | ACH97_203385 |
| P-II family nitrogen regulator | TRNA_RS27355 | BL14DL4_03769 | BLDA23_07235 | CK945_RS06950 | ACH97_203375 |
| MATE family efflux transporter | TRNA_RS27360 | BL14DL4_03770 | BLDA23_07240 | CK945_RS06955 | ACH97_203370 |
| AraC family transcriptional regulator | TRNA_RS27365 | BL14DL4_03771 | BLDA23_07245 | CK945_RS06960 | ACH97_203365 |
| N-acetyltransferase | TRNA_RS27370 | BL14DL4_03772 | BLDA23_07250 | CK945_RS06965 | ACH97_203360 |
| LacI family transcriptional regulator | TRNA_RS27375 | BL14DL4_03773 | BLDA23_07255 | CK945_RS06970 | ACH97_203355 |
| PLP-dependent aminotransferase family protein | TRNA_RS27385 | BL14DL4_03779 | BLDA23_07265 | CK945_RS07000 | ACH97_203325 |
| diaminobutyrate--2-oxoglutarate transaminase | TRNA_RS27390 | BL14DL4_03780 | BLDA23_07270 | CK945_RS07005 | ACH97_203320 |
| aspartate aminotransferase family protein | TRNA_RS27395 | BL14DL4_03781 | BLDA23_07275 | CK945_RS07010 | ACH97_203315 |
| N-acetyltransferase | TRNA_RS27405 | BL14DL4_03783 | BLDA23_07285 | CK945_RS07020 | ACH97_203305 |
| lysine 6-monooxygenase | TRNA_RS27410 | BL14DL4_03784 | BLDA23_07290 | CK945_RS07025 | ACH97_203300 |
| IucA/IucC family siderophore biosynthesis protein | TRNA_RS27415 | BL14DL4_03785 | BLDA23_07295 | CK945_RS07030 | ACH97_203295 |
| GNAT family N-acetyltransferase | TRNA_RS27420 | BL14DL4_03786 | BLDA23_07300 | CK945_RS07035 | ACH97_203290 |
| SDR family NAD(P)-dependent oxidoreductase | TRNA_RS27425 | BL14DL4_03787 | BLDA23_07305 | CK945_RS07040 | ACH97_203285 |
| YafY family transcriptional regulator | TRNA_RS27430 | BL14DL4_03788 | BLDA23_07310 | CK945_RS07045 | ACH97_203280 |
| methionine synthase | TRNA_RS27435 | BL14DL4_03789 | BLDA23_07315 | CK945_RS07050 | ACH97_203275 |
| bifunctional homocysteine S-methyltransferase/methylenetetrahydrofolate reductase | TRNA_RS27440 | BL14DL4_03790 | BLDA23_07320 | CK945_RS07055 | ACH97_203270 |
| YajQ family cyclic di-GMP-binding protein | TRNA_RS27445 | BL14DL4_03791 | BLDA23_07325 | CK945_RS07060 | ACH97_203265 |
| hypothetical protein | TRNA_RS27450 | BL14DL4_03792 | BLDA23_07330 | CK945_RS07065 | ACH97_203260 |
| hypothetical protein | TRNA_RS27455 | BL14DL4_03794 | BLDA23_07340 | CK945_RS07075 | ACH97_203255 |
| DegV family protein | TRNA_RS27460 | BL14DL4_03795 | BLDA23_07345 | CK945_RS07080 | ACH97_203250 |
| YitT family protein | TRNA_RS27465 | BL14DL4_03796 | BLDA23_07350 | CK945_RS07085 | ACH97_203245 |
| proteinase inhibitor | TRNA_RS27470 | BL14DL4_03797 | BLDA23_07355 | CK945_RS07090 | ACH97_203240 |
| GMP reductase | TRNA_RS27475 | BL14DL4_03798 | BLDA23_07360 | CK945_RS07095 | ACH97_203235 |
| DUF3813 domain-containing protein | TRNA_RS27480 | BL14DL4_03799 | BLDA23_07365 | CK945_RS07100 | ACH97_203230 |
| Cof-type HAD-IIB family hydrolase | TRNA_RS27485 | BL14DL4_03800 | BLDA23_07370 | CK945_RS07105 | ACH97_203225 |
| esterase | TRNA_RS27490 | BL14DL4_03801 | BLDA23_07375 | CK945_RS07110 | ACH97_203220 |
| metal-sulfur cluster assembly factor | TRNA_RS27495 | BL14DL4_03802 | BLDA23_07380 | CK945_RS07115 | ACH97_203215 |
| N-acetyl-gamma-glutamyl-phosphate reductase | TRNA_RS27505 | BL14DL4_03803 | BLDA23_07390 | CK945_RS07125 | ACH97_203205 |
| bifunctional glutamate N-acetyltransferase/amino-acid N-acetyltransferase | TRNA_RS27510 | BL14DL4_03804 | BLDA23_07395 | CK945_RS07130 | ACH97_203200 |
| acetylglutamate kinase | TRNA_RS27515 | BL14DL4_03805 | BLDA23_07400 | CK945_RS07135 | ACH97_203195 |
| acetylornithine transaminase | TRNA_RS27520 | BL14DL4_03806 | BLDA23_07405 | CK945_RS07140 | ACH97_203190 |
| carbamoyl phosphate synthase small subunit | TRNA_RS27525 | BL14DL4_03807 | BLDA23_07410 | CK945_RS07145 | ACH97_203185 |
| carbamoyl phosphate synthase large subunit | TRNA_RS27530 | BL14DL4_03808 | BLDA23_07415 | CK945_RS07150 | ACH97_203180 |
| ornithine carbamoyltransferase | TRNA_RS27535 | BL14DL4_03809 | BLDA23_07420 | CK945_RS07155 | ACH97_203175 |
| undecaprenyl-diphosphate phosphatase | TRNA_RS27540 | BL14DL4_03810 | BLDA23_07425 | CK945_RS07160 | ACH97_203170 |
| YjzC family protein | TRNA_RS27545 | BL14DL4_03811 | BLDA23_07430 | CK945_RS07165 | ACH97_203165 |
| DUF2929 family protein | TRNA_RS27550 | BL14DL4_03812 | BLDA23_07435 | CK945_RS07170 | ACH97_203160 |
| hypothetical protein | TRNA_RS27555 | BL14DL4_03813 | BLDA23_07440 | CK945_RS07175 | ACH97_203155 |
| hypothetical protein | TRNA_RS27560 | BL14DL4_03814 | BLDA23_07445 | CK945_RS07180 | ACH97_203150 |
| BMP family ABC transporter substrate-binding protein | TRNA_RS27565 | BL14DL4_03815 | BLDA23_07450 | CK945_RS07185 | ACH97_203145 |
| competence protein ComG | TRNA_RS27570 | BL14DL4_03816 | BLDA23_07455 | CK945_RS07190 | ACH97_203140 |
| hypothetical protein | TRNA_RS27575 | BL14DL4_03817 | BLDA23_07460 | CK945_RS07195 | ACH97_203135 |
| ketoacyl-ACP synthase III | TRNA_RS27580 | BL14DL4_03818 | BLDA23_07465 | CK945_RS07200 | ACH97_203130 |
| beta-ketoacyl-[acyl-carrier-protein] synthase II | TRNA_RS27585 | BL14DL4_03819 | BLDA23_07470 | CK945_RS07205 | ACH97_203125 |
| hypothetical protein | TRNA_RS27590 | BL14DL4_03820 | BLDA23_07475 | CK945_RS07210 | ACH97_203120 |
| ABC transporter ATP-binding protein | TRNA_RS27595 | BL14DL4_03821 | BLDA23_07480 | CK945_RS07215 | ACH97_203115 |
| dipeptide ABC transporter ATP-binding protein | TRNA_RS27600 | BL14DL4_03822 | BLDA23_07485 | CK945_RS07220 | ACH97_203110 |
| peptide-binding protein | TRNA_RS27605 | BL14DL4_03823 | BLDA23_07490 | CK945_RS07225 | ACH97_203105 |
| ABC transporter permease | TRNA_RS27610 | BL14DL4_03824 | BLDA23_07495 | CK945_RS07230 | ACH97_203100 |
| ABC transporter permease | TRNA_RS27615 | BL14DL4_03825 | BLDA23_07500 | CK945_RS07235 | ACH97_203095 |
| MFS transporter | TRNA_RS27620 | BL14DL4_03828 | BLDA23_07515 | CK945_RS07240 | ACH97_203090 |
| DUF3603 family protein | TRNA_RS27625 | BL14DL4_03829 | BLDA23_07520 | CK945_RS07245 | ACH97_203085 |
| tryptophan--tRNA ligase | TRNA_RS27630 | BL14DL4_03830 | BLDA23_07525 | CK945_RS07250 | ACH97_203080 |
| peptide ABC transporter substrate-binding protein | TRNA_RS27640 | BL14DL4_03831 | BLDA23_07535 | CK945_RS07260 | ACH97_203070 |
| ABC transporter permease | TRNA_RS27645 | BL14DL4_03832 | BLDA23_07540 | CK945_RS07265 | ACH97_203065 |
| ABC transporter permease | TRNA_RS27650 | BL14DL4_03833 | BLDA23_07545 | CK945_RS07270 | ACH97_203060 |
| ABC transporter ATP-binding protein | TRNA_RS27655 | BL14DL4_03834 | BLDA23_07550 | CK945_RS07275 | ACH97_203055 |
| ABC transporter ATP-binding protein | TRNA_RS27660 | BL14DL4_03835 | BLDA23_07555 | CK945_RS07280 | ACH97_203050 |
| N-acetyltransferase | TRNA_RS27665 | BL14DL4_03836 | BLDA23_07560 | CK945_RS07285 | ACH97_203045 |
| transcriptional regulator Spx | TRNA_RS27670 | BL14DL4_03837 | BLDA23_07565 | CK945_RS07290 | ACH97_203040 |
| TerC family protein | TRNA_RS27675 | BL14DL4_03838 | BLDA23_07570 | CK945_RS07295 | ACH97_203035 |
| adaptor protein MecA | TRNA_RS27680 | BL14DL4_03839 | BLDA23_07575 | CK945_RS07300 | ACH97_203030 |
| tripartite tricarboxylate transporter substrate binding protein | TRNA_RS27685 | BL14DL4_03840 | BLDA23_07580 | CK945_RS07310 | ACH97_203020 |
| tripartite tricarboxylate transporter TctB family protein | TRNA_RS27690 | BL14DL4_03841 | BLDA23_07585 | CK945_RS07315 | ACH97_203015 |
| tripartite tricarboxylate transporter TctA | TRNA_RS27695 | BL14DL4_03842 | BLDA23_07590 | CK945_RS07320 | ACH97_203010 |
| response regulator | TRNA_RS27700 | BL14DL4_03843 | BLDA23_07595 | CK945_RS07325 | ACH97_203005 |
| sensor histidine kinase | TRNA_RS27705 | BL14DL4_03844 | BLDA23_07600 | CK945_RS07330 | ACH97_203000 |
| hypothetical protein | TRNA_RS27710 | BL14DL4_03845 | BLDA23_07605 | CK945_RS07335 | ACH97_202995 |
| oligoendopeptidase F | TRNA_RS27715 | BL14DL4_03846 | BLDA23_07610 | CK945_RS07340 | ACH97_202990 |
| DsbA family protein | TRNA_RS27725 | BL14DL4_03848 | BLDA23_07615 | CK945_RS07355 | ACH97_202985 |
| thiol management oxidoreductase | TRNA_RS27730 | BL14DL4_03849 | BLDA23_07620 | CK945_RS07360 | ACH97_202980 |
| lytic transglycosylase domain-containing protein | TRNA_RS27735 | BL14DL4_03850 | BLDA23_07625 | CK945_RS07370 | ACH97_202975 |
| CYTH domain-containing protein | TRNA_RS27740 | BL14DL4_03851 | BLDA23_07630 | CK945_RS07375 | ACH97_202970 |
| hypothetical protein | TRNA_RS27745 | BL14DL4_03852 | BLDA23_07635 | CK945_RS07380 | ACH97_202965 |
| GTP pyrophosphokinase family protein | TRNA_RS27750 | BL14DL4_03853 | BLDA23_07640 | CK945_RS07385 | ACH97_202960 |
| NAD kinase | TRNA_RS27755 | BL14DL4_03854 | BLDA23_07645 | CK945_RS07390 | ACH97_202955 |
| RluA family pseudouridine synthase | TRNA_RS27760 | BL14DL4_03855 | BLDA23_07650 | CK945_RS07395 | ACH97_202950 |
| bis(5-nucleosyl)-tetraphosphatase PrpE | TRNA_RS27765 | BL14DL4_03856 | BLDA23_07655 | CK945_RS07400 | ACH97_202945 |
| sodium:proton antiporter | TRNA_RS27775 | BL14DL4_03857 | BLDA23_07665 | CK945_RS07405 | ACH97_202940 |
| thiaminase II | TRNA_RS27780 | BL14DL4_03858 | BLDA23_07670 | CK945_RS07410 | ACH97_202935 |
| thiazole tautomerase TenI | TRNA_RS27785 | BL14DL4_03859 | BLDA23_07675 | CK945_RS07415 | ACH97_202930 |
| glycine oxidase ThiO | TRNA_RS27790 | BL14DL4_03860 | BLDA23_07680 | CK945_RS07420 | ACH97_202925 |
| sulfur carrier protein ThiS | TRNA_RS27795 | BL14DL4_03861 | BLDA23_07685 | CK945_RS07425 | ACH97_202920 |
| thiazole synthase | TRNA_RS27800 | BL14DL4_03862 | BLDA23_07690 | CK945_RS07430 | ACH97_202915 |
| thiazole biosynthesis adenylyltransferase ThiF | TRNA_RS27805 | BL14DL4_03863 | BLDA23_07695 | CK945_RS07435 | ACH97_202910 |
| bifunctional hydroxymethylpyrimidine kinase/phosphomethylpyrimidine kinase | TRNA_RS27810 | BL14DL4_03864 | BLDA23_07700 | CK945_RS07440 | ACH97_202905 |
| enoyl-[acyl-carrier-protein] reductase FabI | TRNA_RS27815 | BL14DL4_03865 | BLDA23_07705 | CK945_RS07445 | ACH97_202900 |
| hypothetical protein | TRNA_RS27820 | BL14DL4_03866 | BLDA23_07710 | CK945_RS07450 | ACH97_202895 |
| spore coat protein | TRNA_RS27825 | BL14DL4_03867 | BLDA23_07715 | CK945_RS07455 | ACH97_202890 |
| spore coat protein | TRNA_RS27830 | BL14DL4_03868 | BLDA23_07720 | CK945_RS07460 | ACH97_202885 |
| spore coat protein | TRNA_RS27835 | BL14DL4_03869 | BLDA23_07725 | CK945_RS07465 | ACH97_202880 |
| hypothetical protein | TRNA_RS27840 | BL14DL4_03870 | BLDA23_07730 | CK945_RS07470 | ACH97_202875 |
| spore coat protein | TRNA_RS27845 | BL14DL4_03871 | BLDA23_07735 | CK945_RS07475 | ACH97_202870 |
| DUF1360 domain-containing protein | TRNA_RS27850 | BL14DL4_03872 | BLDA23_07740 | CK945_RS07480 | ACH97_202865 |
| hypothetical protein | TRNA_RS27855 | BL14DL4_03873 | BLDA23_07745 | CK945_RS07485 | ACH97_202860 |
| sporulation protein | TRNA_RS27865 | BL14DL4_03875 | BLDA23_07755 | CK945_RS07495 | ACH97_202855 |
| ATP-dependent helicase | TRNA_RS27870 | BL14DL4_03876 | BLDA23_07760 | CK945_RS07500 | ACH97_202850 |
| hypothetical protein | TRNA_RS27875 | BL14DL4_03877 | BLDA23_07765 | CK945_RS07505 | ACH97_202845 |
| DedA family protein | TRNA_RS27880 | BL14DL4_03878 | BLDA23_07770 | CK945_RS07510 | ACH97_202840 |
| UTP--glucose-1-phosphate uridylyltransferase | TRNA_RS27890 | BL14DL4_03880 | BLDA23_07780 | CK945_RS07520 | ACH97_202830 |
| GtrA family protein | TRNA_RS27895 | BL14DL4_03881 | BLDA23_07785 | CK945_RS07525 | ACH97_202825 |
| GNAT family N-acetyltransferase | TRNA_RS27900 | BL14DL4_03882 | BLDA23_07790 | CK945_RS07530 | ACH97_202820 |
| hypothetical protein | TRNA_RS27905 | BL14DL4_03883 | BLDA23_07795 | CK945_RS07535 | ACH97_202815 |
| esterase family protein | TRNA_RS27910 | BL14DL4_03884 | BLDA23_07800 | CK945_RS07540 | ACH97_202810 |
| DNA damage-inducible protein DinB | TRNA_RS27920 | BL14DL4_03885 | BLDA23_07810 | CK945_RS07550 | ACH97_202800 |
| YafY family transcriptional regulator | TRNA_RS27925 | BL14DL4_03886 | BLDA23_07815 | CK945_RS07555 | ACH97_202795 |
| methionine biosynthesis PLP-dependent protein | TRNA_RS27930 | BL14DL4_03887 | BLDA23_07820 | CK945_RS07560 | ACH97_202790 |
| cystathionine beta-lyase MetC | TRNA_RS27935 | BL14DL4_03888 | BLDA23_07825 | CK945_RS07565 | ACH97_202785 |
| DUF819 domain-containing protein | TRNA_RS27940 | BL14DL4_03889 | BLDA23_07830 | CK945_RS07570 | ACH97_202780 |
| MarR family transcriptional regulator | TRNA_RS27945 | BL14DL4_03890 | BLDA23_07835 | CK945_RS07575 | ACH97_202775 |
| hypothetical protein | TRNA_RS27950 | BL14DL4_03891 | BLDA23_07840 | CK945_RS07580 | ACH97_202770 |
| arabinan endo-1,5-alpha-L-arabinosidase | TRNA_RS27960 | BL14DL4_03893 | BLDA23_07850 | CK945_RS07595 | ACH97_202755 |
| hypothetical protein | TRNA_RS27975 | BL14DL4_03895 | BLDA23_07865 | CK945_RS07665 | ACH97_202690 |
| maltose O-acetyltransferase | TRNA_RS27980 | BL14DL4_03896 | BLDA23_07870 | CK945_RS07670 | ACH97_202685 |
| hypothetical protein | TRNA_RS27985 | BL14DL4_03897 | BLDA23_07875 | CK945_RS07675 | ACH97_202680 |
| LysR family transcriptional regulator | TRNA_RS27990 | BL14DL4_03898 | BLDA23_07880 | CK945_RS07680 | ACH97_202675 |
| assimilatory sulfite reductase (NADPH) flavoprotein subunit | TRNA_RS27995 | BL14DL4_03899 | BLDA23_07890 | CK945_RS07685 | ACH97_202670 |
| assimilatory sulfite reductase (NADPH) hemoprotein subunit | TRNA_RS28000 | BL14DL4_03900 | BLDA23_07895 | CK945_RS07690 | ACH97_202665 |
| DMT family transporter | TRNA_RS28010 | BL14DL4_03902 | BLDA23_07905 | CK945_RS07700 | ACH97_202655 |
| glyoxalase | TRNA_RS28015 | BL14DL4_03903 | BLDA23_07910 | CK945_RS07705 | ACH97_202650 |
| hypothetical protein | TRNA_RS28020 | BL14DL4_03904 | BLDA23_07915 | CK945_RS07710 | ACH97_202645 |
| LysM peptidoglycan-binding domain-containing protein | TRNA_RS28025 | BL14DL4_03905 | BLDA23_07920 | CK945_RS07715 | ACH97_202640 |
| DHA2 family efflux MFS transporter permease subunit | TRNA_RS28030 | BL14DL4_03906 | BLDA23_07925 | CK945_RS07720 | ACH97_202635 |
| TetR/AcrR family transcriptional regulator | TRNA_RS28035 | BL14DL4_03907 | BLDA23_07930 | CK945_RS07725 | ACH97_202630 |
| hypothetical protein | TRNA_RS28045 | BL14DL4_03908 | BLDA23_07940 | CK945_RS07735 | ACH97_202620 |
| hypothetical protein | TRNA_RS28060 | BL14DL4_03912 | BLDA23_07955 | CK945_RS07750 | ACH97_202615 |
| ImmA/IrrE family metallo-endopeptidase | TRNA_RS28065 | BL14DL4_03913 | BLDA23_07960 | CK945_RS07755 | ACH97_202610 |
| XRE family transcriptional regulator | TRNA_RS28070 | BL14DL4_03914 | BLDA23_07965 | CK945_RS07760 | ACH97_202605 |
| hypothetical protein | TRNA_RS28080 | BL14DL4_03916 | BLDA23_07975 | CK945_RS07770 | ACH97_202600 |
| hypothetical protein | TRNA_RS28085 | BL14DL4_03917 | BLDA23_07980 | CK945_RS07775 | ACH97_202595 |
| ATP-binding protein | TRNA_RS28090 | BL14DL4_03918 | BLDA23_07985 | CK945_RS07780 | ACH97_202590 |
| hypothetical protein | TRNA_RS28095 | BL14DL4_03920 | BLDA23_07990 | CK945_RS07785 | ACH97_202585 |
| hypothetical protein | TRNA_RS28100 | BL14DL4_03921 | BLDA23_07995 | CK945_RS07790 | ACH97_202580 |
| sigma-70 family RNA polymerase sigma factor | TRNA_RS28105 | BL14DL4_03922 | BLDA23_08000 | CK945_RS07795 | ACH97_202575 |
| terminase | TRNA_RS28110 | BL14DL4_03923 | BLDA23_08005 | CK945_RS07800 | ACH97_202570 |
| PBSX family phage terminase large subunit | TRNA_RS28115 | BL14DL4_03924 | BLDA23_08010 | CK945_RS07805 | ACH97_202565 |
| phage portal protein | TRNA_RS28120 | BL14DL4_03925 | BLDA23_08015 | CK945_RS07810 | ACH97_202560 |
| phage portal protein | TRNA_RS28125 | BL14DL4_03926 | BLDA23_08020 | CK945_RS07815 | ACH97_202555 |
| phage portal protein | TRNA_RS28130 | BL14DL4_03927 | BLDA23_08025 | CK945_RS07820 | ACH97_202550 |
| DUF3199 family protein | TRNA_RS28135 | BL14DL4_03928 | BLDA23_08030 | CK945_RS07825 | ACH97_202545 |
| DUF3599 family protein | TRNA_RS28140 | BL14DL4_03929 | BLDA23_08035 | CK945_RS07830 | ACH97_202540 |
| HK97 gp10 family phage protein | TRNA_RS28145 | BL14DL4_03930 | BLDA23_08040 | CK945_RS07835 | ACH97_202535 |
| hypothetical protein | TRNA_RS28150 | BL14DL4_03931 | BLDA23_08045 | CK945_RS07840 | ACH97_202530 |
| hypothetical protein | TRNA_RS28155 | BL14DL4_03932 | BLDA23_08050 | CK945_RS07845 | ACH97_202525 |
| phage portal protein | TRNA_RS28160 | BL14DL4_03933 | BLDA23_08055 | CK945_RS07850 | ACH97_202520 |
| phage portal protein | TRNA_RS28165 | BL14DL4_03934 | BLDA23_08060 | CK945_RS07855 | ACH97_202515 |
| phage portal protein | TRNA_RS28170 | BL14DL4_03935 | BLDA23_08065 | CK945_RS07860 | ACH97_202510 |
| LysM peptidoglycan-binding domain-containing protein | TRNA_RS28180 | BL14DL4_03937 | BLDA23_08080 | CK945_RS07875 | ACH97_202500 |
| hypothetical protein | TRNA_RS28185 | BL14DL4_03938 | BLDA23_08085 | CK945_RS07880 | ACH97_202495 |
| DUF2577 domain-containing protein | TRNA_RS28190 | BL14DL4_03939 | BLDA23_08090 | CK945_RS07885 | ACH97_202490 |
| DUF2634 domain-containing protein | TRNA_RS28195 | BL14DL4_03940 | BLDA23_08095 | CK945_RS07890 | ACH97_202485 |
| baseplate J/gp47 family protein | TRNA_RS28200 | BL14DL4_03941 | BLDA23_08100 | CK945_RS07895 | ACH97_202480 |
| hypothetical protein | TRNA_RS28210 | BL14DL4_03943 | BLDA23_08110 | CK945_RS07905 | ACH97_202470 |
| protein xhlA | TRNA_RS28225 | BL14DL4_03946 | BLDA23_08125 | CK945_RS07920 | ACH97_202725 |
| phage holin | TRNA_RS28230 | BL14DL4_03947 | BLDA23_08130 | CK945_RS07925 | ACH97_202455 |
| N-acetylmuramoyl-L-alanine amidase | TRNA_RS28235 | BL14DL4_03948 | BLDA23_08135 | CK945_RS07930 | ACH97_202445 |
| GntR family transcriptional regulator | TRNA_RS28240 | BL14DL4_03949 | BLDA23_08145 | CK945_RS07935 | ACH97_202440 |
| alcohol dehydrogenase | TRNA_RS28245 | BL14DL4_03950 | BLDA23_08150 | CK945_RS07940 | ACH97_202435 |
| Mannonate dehydratase 1 | TRNA_RS28250 | BL14DL4_03951 | BLDA23_08155 | CK945_RS07945 | ACH97_202430 |
| SDR family oxidoreductase | TRNA_RS28255 | BL14DL4_03952 | BLDA23_08160 | CK945_RS07950 | ACH97_202425 |
| alcohol dehydrogenase | TRNA_RS28260 | BL14DL4_03953 | BLDA23_08165 | CK945_RS07955 | ACH97_202420 |
| MFS transporter | TRNA_RS28265 | BL14DL4_03954 | BLDA23_08170 | CK945_RS07960 | ACH97_202415 |
| stage II sporulation protein SB | TRNA_RS28270 | BL14DL4_03955 | BLDA23_08175 | CK945_RS07965 | ACH97_202410 |
| inorganic phosphate transporter | TRNA_RS28280 | BL14DL4_03957 | BLDA23_08185 | CK945_RS07975 | ACH97_202400 |
| DUF47 domain-containing protein | TRNA_RS28285 | BL14DL4_03958 | BLDA23_08190 | CK945_RS07980 | ACH97_202395 |
| gamma-glutamyltransferase | TRNA_RS28290 | BL14DL4_03959 | BLDA23_08195 | CK945_RS07985 | ACH97_202390 |
| sensor histidine kinase | TRNA_RS28300 | BL14DL4_03961 | BLDA23_08205 | CK945_RS07995 | ACH97_202380 |
| DNA-binding response regulator | TRNA_RS28305 | BL14DL4_03962 | BLDA23_08210 | CK945_RS08000 | ACH97_202375 |
| carbohydrate ABC transporter substrate-binding protein | TRNA_RS28310 | BL14DL4_03963 | BLDA23_08215 | CK945_RS08005 | ACH97_202370 |
| sugar ABC transporter permease | TRNA_RS28315 | BL14DL4_03964 | BLDA23_08220 | CK945_RS08010 | ACH97_202365 |
| carbohydrate ABC transporter permease | TRNA_RS28320 | BL14DL4_03965 | BLDA23_08225 | CK945_RS08015 | ACH97_202360 |
| glycoside hydrolase family 105 protein | TRNA_RS28325 | BL14DL4_03966 | BLDA23_08230 | CK945_RS08020 | ACH97_202355 |
| AraC family transcriptional regulator | TRNA_RS28330 | BL14DL4_03967 | BLDA23_08235 | CK945_RS08025 | ACH97_202350 |
| rhamnogalacturonan acetylesterase | TRNA_RS28335 | BL14DL4_03968 | BLDA23_08240 | CK945_RS08030 | ACH97_202345 |
| DUF1961 family protein | TRNA_RS28340 | BL14DL4_03969 | BLDA23_08245 | CK945_RS08035 | ACH97_202340 |
| DUF624 domain-containing protein | TRNA_RS28345 | BL14DL4_03970 | BLDA23_08250 | CK945_RS08040 | ACH97_202335 |
| rhamnogalacturonan lyase | TRNA_RS28350 | BL14DL4_03971 | BLDA23_08255 | CK945_RS08045 | ACH97_202330 |
| carbohydrate esterase | TRNA_RS43195 | BL14DL4_03973 | BLDA23_08265 | CK945_RS08055 | ACH97_202320 |
| rhamnogalacturonan lyase | TRNA_RS28365 | BL14DL4_03974 | BLDA23_08270 | CK945_RS08060 | ACH97_202315 |
| beta-galactosidase | TRNA_RS28380 | BL14DL4_03977 | BLDA23_08285 | CK945_RS08075 | ACH97_202300 |
| hypothetical protein | TRNA_RS28385 | BL14DL4_03978 | BLDA23_08290 | CK945_RS08080 | ACH97_202295 |
| extracellular solute-binding protein | TRNA_RS28390 | BL14DL4_03979 | BLDA23_08295 | CK945_RS08085 | ACH97_202290 |
| sugar ABC transporter permease | TRNA_RS28395 | BL14DL4_03980 | BLDA23_08300 | CK945_RS08090 | ACH97_202285 |
| carbohydrate ABC transporter permease | TRNA_RS28400 | BL14DL4_03981 | BLDA23_08305 | CK945_RS08095 | ACH97_202280 |
| amino acid permease | TRNA_RS28405 | BL14DL4_03982 | BLDA23_08310 | CK945_RS08100 | ACH97_202275 |
| ring-cleaving dioxygenase | TRNA_RS28410 | BL14DL4_03983 | BLDA23_08315 | CK945_RS08105 | ACH97_202270 |
| hypothetical protein | TRNA_RS28415 | BL14DL4_03984 | BLDA23_08320 | CK945_RS13050 | ACH97_208865 |
| PDZ domain-containing protein | TRNA_RS28420 | BL14DL4_03985 | BLDA23_08325 | CK945_RS08110 | ACH97_202265 |
| pyrroline-5-carboxylate reductase | TRNA_RS28425 | BL14DL4_03986 | BLDA23_08330 | CK945_RS08115 | ACH97_202260 |
| D-aminopeptidase | TRNA_RS28430 | BL14DL4_03987 | BLDA23_08335 | CK945_RS08120 | ACH97_221705 |
| ABC transporter permease | TRNA_RS28435 | BL14DL4_03988 | BLDA23_08340 | CK945_RS08125 | ACH97_221700 |
| ABC transporter permease | TRNA_RS28440 | BL14DL4_03989 | BLDA23_08345 | CK945_RS08130 | ACH97_221695 |
| ABC transporter ATP-binding protein | TRNA_RS28445 | BL14DL4_03990 | BLDA23_08350 | CK945_RS08135 | ACH97_221690 |
| peptide ABC transporter substrate-binding protein | TRNA_RS28450 | BL14DL4_03991 | BLDA23_08355 | CK945_RS08140 | ACH97_221685 |
| LD-carboxypeptidase | TRNA_RS28455 | BL14DL4_03992 | BLDA23_08360 | CK945_RS08145 | ACH97_221680 |
| dipeptide epimerase | TRNA_RS28460 | BL14DL4_03993 | BLDA23_08365 | CK945_RS08150 | ACH97_221675 |
| NlpC/P60 family protein | TRNA_RS28465 | BL14DL4_03994 | BLDA23_08370 | CK945_RS08155 | ACH97_221670 |
| ABC transporter ATP-binding protein | TRNA_RS28470 | BL14DL4_03995 | BLDA23_08375 | CK945_RS08160 | ACH97_221665 |
| hypothetical protein | TRNA_RS28475 | BL14DL4_03996 | BLDA23_08380 | CK945_RS08165 | ACH97_221650 |
| PadR family transcriptional regulator | TRNA_RS28495 | BL14DL4_04000 | BLDA23_08400 | CK945_RS08185 | ACH97_221630 |
| QacE family quaternary ammonium compound efflux SMR transporter | TRNA_RS28515 | BL14DL4_04004 | BLDA23_08420 | CK945_RS08205 | ACH97_221610 |
| QacE family quaternary ammonium compound efflux SMR transporter | TRNA_RS28520 | BL14DL4_04005 | BLDA23_08425 | CK945_RS08210 | ACH97_221605 |
| formyltetrahydrofolate deformylase | TRNA_RS28525 | BL14DL4_04006 | BLDA23_08430 | CK945_RS08215 | ACH97_221600 |
| glutamate 5-kinase | TRNA_RS28530 | BL14DL4_04007 | BLDA23_08435 | CK945_RS08225 | ACH97_221590 |
| glutamate-5-semialdehyde dehydrogenase | TRNA_RS28535 | BL14DL4_04008 | BLDA23_08440 | CK945_RS08230 | ACH97_221585 |
| organic hydroperoxide resistance protein | TRNA_RS28540 | BL14DL4_04009 | BLDA23_08445 | CK945_RS08235 | ACH97_221580 |
| MarR family transcriptional regulator | TRNA_RS28545 | BL14DL4_04010 | BLDA23_08455 | CK945_RS08240 | ACH97_221575 |
| organic hydroperoxide resistance protein | TRNA_RS28550 | BL14DL4_04011 | BLDA23_08460 | CK945_RS08245 | ACH97_221570 |
| hypothetical protein | TRNA_RS28555 | BL14DL4_04012 | BLDA23_08465 | CK945_RS08250 | ACH97_221565 |
| nucleoside deaminase | TRNA_RS28560 | BL14DL4_04013 | BLDA23_08470 | CK945_RS08255 | ACH97_221560 |
| histidine phosphatase family protein | TRNA_RS28565 | BL14DL4_04014 | BLDA23_08475 | CK945_RS08260 | ACH97_221555 |
| hypothetical protein | TRNA_RS28570 | BL14DL4_04015 | BLDA23_08480 | CK945_RS08265 | ACH97_221550 |
| DUF5082 domain-containing protein | TRNA_RS28575 | BL14DL4_04016 | BLDA23_08485 | CK945_RS08270 | ACH97_221545 |
| 5-methyltetrahydropteroyltriglutamate-- homocysteine methyltransferase | TRNA_RS28580 | BL14DL4_04017 | BLDA23_08490 | CK945_RS08275 | ACH97_221540 |
| serine protease | TRNA_RS28585 | BL14DL4_04018 | BLDA23_08495 | CK945_RS08280 | ACH97_221535 |
| magnesium transporter | TRNA_RS28590 | BL14DL4_04020 | BLDA23_08505 | CK945_RS08285 | ACH97_221530 |
| hypothetical protein | TRNA_RS28595 | BL14DL4_04021 | BLDA23_08510 | CK945_RS08290 | ACH97_221525 |
| phage holin | TRNA_RS28885 | BL14DL4_01838 | BLDA23_15725 | CK945_RS05645 | ACH97_210740 |
| MerR family transcriptional regulator | TRNA_RS28910 | BL14DL4_04022 | BLDA23_08515 | CK945_RS08295 | ACH97_221520 |
| hypothetical protein | TRNA_RS28915 | BL14DL4_04024 | BLDA23_08525 | CK945_RS08305 | ACH97_221515 |
| MarR family transcriptional regulator | TRNA_RS28920 | BL14DL4_04026 | BLDA23_08530 | CK945_RS08310 | ACH97_221510 |
| DNA ligase D | TRNA_RS28925 | BL14DL4_04027 | BLDA23_08535 | CK945_RS08315 | ACH97_221505 |
| Ku protein | TRNA_RS28930 | BL14DL4_04028 | BLDA23_08540 | CK945_RS08320 | ACH97_221500 |
| diguanylate cyclase | TRNA_RS28935 | BL14DL4_04029 | BLDA23_08545 | CK945_RS08325 | ACH97_221495 |
| DedA family protein | TRNA_RS28940 | BL14DL4_04030 | BLDA23_08550 | CK945_RS08330 | ACH97_221490 |
| membrane protein | TRNA_RS28945 | BL14DL4_04031 | BLDA23_08555 | CK945_RS08335 | ACH97_221485 |
| RNA polymerase sigma factor SigI | TRNA_RS28950 | BL14DL4_04032 | BLDA23_08560 | CK945_RS08340 | ACH97_221480 |
| hypothetical protein | TRNA_RS28955 | BL14DL4_04033 | BLDA23_08565 | CK945_RS08345 | ACH97_221475 |
| small acid-soluble spore protein | TRNA_RS28960 | BL14DL4_04034 | BLDA23_08570 | CK945_RS08350 | ACH97_221470 |
| DUF1836 domain-containing protein | TRNA_RS28965 | BL14DL4_04035 | BLDA23_08575 | CK945_RS08355 | ACH97_221465 |
| protease HtpX | TRNA_RS28970 | BL14DL4_04036 | BLDA23_08580 | CK945_RS08360 | ACH97_221460 |
| TrkH family potassium uptake protein | TRNA_RS28975 | BL14DL4_04037 | BLDA23_08585 | CK945_RS08365 | ACH97_221455 |
| hypothetical protein | TRNA_RS28985 | BL14DL4_04039 | BLDA23_08595 | CK945_RS08380 | ACH97_221445 |
| hypothetical protein | TRNA_RS28990 | BL14DL4_04040 | BLDA23_08600 | CK945_RS08385 | ACH97_221440 |
| acyltransferase | TRNA_RS28995 | BL14DL4_04041 | BLDA23_08605 | CK945_RS08390 | ACH97_222220 |
| PAS domain-containing sensor histidine kinase | TRNA_RS29000 | BL14DL4_04042 | BLDA23_08610 | CK945_RS08395 | ACH97_222225 |
| S-methyl-5-thioribose-1-phosphate isomerase | TRNA_RS29010 | BL14DL4_04045 | BLDA23_08620 | CK945_RS08405 | ACH97_222235 |
| S-methyl-5-thioribose kinase | TRNA_RS29015 | BL14DL4_04046 | BLDA23_08625 | CK945_RS08410 | ACH97_222240 |
| carbon-nitrogen family hydrolase | TRNA_RS29020 | BL14DL4_04047 | BLDA23_08630 | CK945_RS08415 | ACH97_222245 |
| pyridoxal phosphate-dependent aminotransferase | TRNA_RS29025 | BL14DL4_04048 | BLDA23_08635 | CK945_RS08420 | ACH97_222250 |
| 2,3-diketo-5-methylthiopentyl-1-phosphate enolase | TRNA_RS29030 | BL14DL4_04049 | BLDA23_08640 | CK945_RS08425 | ACH97_222255 |
| methylthioribulose 1-phosphate dehydratase | TRNA_RS29035 | BL14DL4_04050 | BLDA23_08645 | CK945_RS08430 | ACH97_222260 |
| cupin domain-containing protein | TRNA_RS29040 | BL14DL4_04051 | BLDA23_08650 | CK945_RS08440 | ACH97_222270 |
| metallothiol transferase FosB | TRNA_RS29045 | BL14DL4_04052 | BLDA23_08655 | CK945_RS08445 | ACH97_222275 |
| DUF1232 domain-containing protein | TRNA_RS29050 | BL14DL4_04053 | BLDA23_08660 | CK945_RS08450 | ACH97_222280 |
| aspartyl-phosphate phosphatase Spo0E family protein | TRNA_RS29055 | BL14DL4_04054 | BLDA23_08665 | CK945_RS08455 | ACH97_222285 |
| two-component sensor histidine kinase | TRNA_RS29060 | BL14DL4_04055 | BLDA23_08670 | CK945_RS08460 | ACH97_222290 |
| MarR family transcriptional regulator | TRNA_RS29065 | BL14DL4_04056 | BLDA23_08675 | CK945_RS08465 | ACH97_222295 |
| flagellar motor protein MotB | TRNA_RS29070 | BL14DL4_04057 | BLDA23_08680 | CK945_RS08470 | ACH97_222300 |
| flagellar motor protein MotA | TRNA_RS29075 | BL14DL4_04058 | BLDA23_08685 | CK945_RS08475 | ACH97_222305 |
| ATP-dependent Clp protease ATP-binding subunit | TRNA_RS29080 | BL14DL4_04059 | BLDA23_08690 | CK945_RS08480 | ACH97_222310 |
| hypothetical protein | TRNA_RS29085 | BL14DL4_04060 | BLDA23_08695 | CK945_RS08485 | ACH97_222315 |
| 7-cyano-7-deazaguanine synthase QueC | TRNA_RS29090 | BL14DL4_04061 | BLDA23_08700 | CK945_RS08490 | ACH97_222320 |
| 6-carboxytetrahydropterin synthase QueD | TRNA_RS29095 | BL14DL4_04062 | BLDA23_08705 | CK945_RS08495 | ACH97_222325 |
| 7-carboxy-7-deazaguanine synthase QueE | TRNA_RS29100 | BL14DL4_04063 | BLDA23_08710 | CK945_RS08500 | ACH97_222330 |
| NADPH-dependent 7-cyano-7-deazaguanine reductase QueF | TRNA_RS29105 | BL14DL4_04064 | BLDA23_08715 | CK945_RS08505 | ACH97_222335 |
| DUF817 domain-containing protein | TRNA_RS29360 | BL14DL4_04069 | BLDA23_08725 | CK945_RS08665 | ACH97_218550 |
| transcriptional regulator | TRNA_RS29365 | BL14DL4_04070 | BLDA23_08730 | CK945_RS08670 | ACH97_218545 |
| DUF2975 domain-containing protein | TRNA_RS29370 | BL14DL4_04071 | BLDA23_08735 | CK945_RS08675 | ACH97_218540 |
| DUF1433 domain-containing protein | TRNA_RS29385 | BL14DL4_04074 | BLDA23_08755 | CK945_RS08690 | ACH97_218525 |
| DUF1672 family protein | TRNA_RS29390 | BL14DL4_04075 | BLDA23_08760 | CK945_RS08700 | ACH97_218515 |
| DUF2187 domain-containing protein | TRNA_RS29395 | BL14DL4_04076 | BLDA23_08765 | CK945_RS08710 | ACH97_218510 |
| hypothetical protein | TRNA_RS29400 | BL14DL4_04077 | BLDA23_08770 | CK945_RS08715 | ACH97_218505 |
| cell wall hydrolase | TRNA_RS29405 | BL14DL4_04078 | BLDA23_08775 | CK945_RS08720 | ACH97_218500 |
| polysaccharide biosynthesis protein | TRNA_RS29410 | BL14DL4_04079 | BLDA23_08780 | CK945_RS08725 | ACH97_218495 |
| TlpA family protein disulfide reductase | TRNA_RS29415 | BL14DL4_04080 | BLDA23_08785 | CK945_RS08730 | ACH97_218490 |
| heavy metal translocating P-type ATPase | TRNA_RS29420 | BL14DL4_04081 | BLDA23_08790 | CK945_RS08735 | ACH97_218485 |
| aminopeptidase P family protein | TRNA_RS29425 | BL14DL4_04082 | BLDA23_08795 | CK945_RS08740 | ACH97_218480 |
| hypothetical protein | TRNA_RS29430 | BL14DL4_04084 | BLDA23_08800 | CK945_RS08745 | ACH97_218475 |
| LacI family DNA-binding transcriptional regulator | TRNA_RS29435 | BL14DL4_04085 | BLDA23_08805 | CK945_RS08750 | ACH97_218470 |
| transcription antiterminator | TRNA_RS29440 | BL14DL4_04086 | BLDA23_08810 | CK945_RS08755 | ACH97_218465 |
| phosphocarrier protein HPr | TRNA_RS29450 | BL14DL4_04088 | BLDA23_08820 | CK945_RS08765 | ACH97_218455 |
| phosphoenolpyruvate--protein phosphotransferase | TRNA_RS29455 | BL14DL4_04089 | BLDA23_08825 | CK945_RS08770 | ACH97_218450 |
| transcriptional regulator | TRNA_RS29460 | BL14DL4_04090 | BLDA23_08830 | CK945_RS08775 | ACH97_218445 |
| spore photoproduct lyase | TRNA_RS29465 | BL14DL4_04091 | BLDA23_08835 | CK945_RS08780 | ACH97_218440 |
| GNAT family N-acetyltransferase | TRNA_RS29470 | BL14DL4_04092 | BLDA23_08840 | CK945_RS08785 | ACH97_218435 |
| methyl-accepting chemotaxis protein | TRNA_RS29475 | BL14DL4_04093 | BLDA23_08845 | CK945_RS08790 | ACH97_218430 |
| SDR family oxidoreductase | TRNA_RS29480 | BL14DL4_04094 | BLDA23_08850 | CK945_RS08795 | ACH97_218425 |
| NAD(P)-dependent oxidoreductase | TRNA_RS29485 | BL14DL4_04095 | BLDA23_08855 | CK945_RS08800 | ACH97_218420 |
| hypothetical protein | TRNA_RS29490 | BL14DL4_04096 | BLDA23_08860 | CK945_RS08805 | ACH97_218415 |
| penicillin-binding protein 2 | TRNA_RS29495 | BL14DL4_04097 | BLDA23_08865 | CK945_RS08810 | ACH97_218410 |
| PAS domain-containing sensor histidine kinase | TRNA_RS29500 | BL14DL4_04098 | BLDA23_08870 | CK945_RS08815 | ACH97_218405 |
| aminotransferase A | TRNA_RS29505 | BL14DL4_04099 | BLDA23_08875 | CK945_RS08820 | ACH97_218400 |
| RDD family protein | TRNA_RS29510 | BL14DL4_04101 | BLDA23_08880 | CK945_RS08825 | ACH97_218395 |
| MFS transporter | TRNA_RS29515 | BL14DL4_04102 | BLDA23_08885 | CK945_RS08830 | ACH97_218390 |
| chemotaxis protein CheV | TRNA_RS29520 | BL14DL4_04103 | BLDA23_08890 | CK945_RS08835 | ACH97_218385 |
| hypothetical protein | TRNA_RS29525 | BL14DL4_04104 | BLDA23_08895 | CK945_RS08840 | ACH97_218380 |
| MFS transporter | TRNA_RS29530 | BL14DL4_04105 | BLDA23_08900 | CK945_RS08845 | ACH97_218375 |
| L,D-transpeptidase | TRNA_RS29535 | BL14DL4_04106 | BLDA23_08905 | CK945_RS08850 | ACH97_218370 |
| metallophosphoesterase | TRNA_RS29540 | BL14DL4_04107 | BLDA23_08915 | CK945_RS08855 | ACH97_218365 |
| 2,4-dienoyl-CoA reductase | TRNA_RS29545 | BL14DL4_04108 | BLDA23_08920 | CK945_RS08860 | ACH97_218360 |
| EAL domain-containing protein | TRNA_RS29550 | BL14DL4_04109 | BLDA23_08925 | CK945_RS08865 | ACH97_218355 |
| DUF3993 domain-containing protein | TRNA_RS29555 | BL14DL4_04110 | BLDA23_08930 | CK945_RS08870 | ACH97_218350 |
| DUF1797 family protein | TRNA_RS29560 | BL14DL4_04111 | BLDA23_08935 | CK945_RS08880 | ACH97_218345 |
| hypothetical protein | TRNA_RS29565 | BL14DL4_04113 | BLDA23_08940 | CK945_RS08890 | ACH97_218340 |
| antirepressor AbbA | TRNA_RS29570 | BL14DL4_04114 | BLDA23_08945 | CK945_RS08895 | ACH97_218335 |
| CBS domain-containing protein | TRNA_RS29575 | BL14DL4_04115 | BLDA23_08950 | CK945_RS08900 | ACH97_218330 |
| LysR family transcriptional regulator | TRNA_RS29580 | BL14DL4_04116 | BLDA23_08955 | CK945_RS08905 | ACH97_218325 |
| flavodoxin | TRNA_RS29585 | BL14DL4_04117 | BLDA23_08960 | CK945_RS08910 | ACH97_218320 |
| hypothetical protein | TRNA_RS29590 | BL14DL4_04118 | BLDA23_08965 | CK945_RS08915 | ACH97_218315 |
| flavodoxin | TRNA_RS29595 | BL14DL4_04119 | BLDA23_08970 | CK945_RS08920 | ACH97_218310 |
| 2,3,4,5-tetrahydropyridine-2,6-dicarboxylate N-acetyltransferase | TRNA_RS29600 | BL14DL4_04120 | BLDA23_08975 | CK945_RS08925 | ACH97_218305 |
| N-acetyldiaminopimelate deacetylase | TRNA_RS29605 | BL14DL4_04121 | BLDA23_08980 | CK945_RS08930 | ACH97_218300 |
| YkuS family protein | TRNA_RS29610 | BL14DL4_04122 | BLDA23_08985 | CK945_RS08935 | ACH97_218295 |
| peroxiredoxin | TRNA_RS29615 | BL14DL4_04123 | BLDA23_08990 | CK945_RS08940 | ACH97_218290 |
| thiol-disulfide oxidoreductase | TRNA_RS29620 | BL14DL4_04124 | BLDA23_08995 | CK945_RS08945 | ACH97_218285 |
| hypothetical protein | TRNA_RS29625 | BL14DL4_04125 | BLDA23_09000 | CK945_RS08950 | ACH97_218280 |
| hypothetical protein | TRNA_RS29630 | BL14DL4_04126 | BLDA23_09005 | CK945_RS08955 | ACH97_218275 |
| molybdenum cofactor guanylyltransferase | TRNA_RS29635 | BL14DL4_04127 | BLDA23_09010 | CK945_RS08960 | ACH97_218270 |
| molybdopterin-synthase adenylyltransferase MoeB | TRNA_RS29640 | BL14DL4_04128 | BLDA23_09015 | CK945_RS08965 | ACH97_218265 |
| molybdopterin molybdenumtransferase MoeA | TRNA_RS29645 | BL14DL4_04129 | BLDA23_09020 | CK945_RS08970 | ACH97_218260 |
| molybdopterin-guanine dinucleotide biosynthesis protein B | TRNA_RS29650 | BL14DL4_04130 | BLDA23_09025 | CK945_RS08975 | ACH97_218255 |
| molybdenum cofactor biosynthesis protein MoaE | TRNA_RS29655 | BL14DL4_04131 | BLDA23_09030 | CK945_RS08980 | ACH97_218250 |
| molybdopterin converting factor subunit 1 | TRNA_RS29660 | BL14DL4_04132 | BLDA23_09035 | CK945_RS08985 | ACH97_218245 |
| hypothetical protein | TRNA_RS29665 | BL14DL4_04133 | BLDA23_09040 | CK945_RS08990 | ACH97_218240 |
| ABC transporter ATP-binding protein | TRNA_RS29670 | BL14DL4_04134 | BLDA23_09045 | CK945_RS08995 | ACH97_218235 |
| ABC transporter ATP-binding protein | TRNA_RS29675 | BL14DL4_04135 | BLDA23_09050 | CK945_RS09000 | ACH97_218230 |
| YIP1 family protein | TRNA_RS29680 | BL14DL4_04136 | BLDA23_09055 | CK945_RS09005 | ACH97_218225 |
| efflux RND transporter periplasmic adaptor subunit | TRNA_RS29685 | BL14DL4_04137 | BLDA23_09060 | CK945_RS09010 | ACH97_218220 |
| macrolide ABC transporter permease | TRNA_RS29695 | BL14DL4_04139 | BLDA23_09070 | CK945_RS09020 | ACH97_218210 |
| DeoR/GlpR transcriptional regulator | TRNA_RS29700 | BL14DL4_04140 | BLDA23_09075 | CK945_RS09025 | ACH97_218200 |
| 1-phosphofructokinase | TRNA_RS29705 | BL14DL4_04141 | BLDA23_09080 | CK945_RS09030 | ACH97_218195 |
| PTS fructose transporter subunit IIC | TRNA_RS29710 | BL14DL4_04142 | BLDA23_09085 | CK945_RS09035 | ACH97_218190 |
| signal peptidase I | TRNA_RS29715 | BL14DL4_04143 | BLDA23_09090 | CK945_RS09040 | ACH97_218185 |
| hypothetical protein | TRNA_RS29720 | BL14DL4_04144 | BLDA23_09095 | CK945_RS09045 | ACH97_218180 |
| hypothetical protein | TRNA_RS29725 | BL14DL4_04145 | BLDA23_09100 | CK945_RS09050 | ACH97_218175 |
| ATP-binding cassette domain-containing protein | TRNA_RS29730 | BL14DL4_04146 | BLDA23_09105 | CK945_RS09055 | ACH97_218170 |
| DUF4440 domain-containing protein | TRNA_RS29735 | BL14DL4_04147 | BLDA23_09110 | CK945_RS09060 | ACH97_218165 |
| aminopeptidase | TRNA_RS29740 | BL14DL4_04148 | BLDA23_09115 | CK945_RS09065 | ACH97_218160 |
| rod shape-determining protein | TRNA_RS29745 | BL14DL4_04150 | BLDA23_09125 | CK945_RS09075 | ACH97_218155 |
| AbrB/MazE/SpoVT family DNA-binding domain-containing protein | TRNA_RS29750 | BL14DL4_04151 | BLDA23_09130 | CK945_RS09080 | ACH97_218150 |
| PAS domain-containing sensor histidine kinase | TRNA_RS29755 | BL14DL4_04152 | BLDA23_09135 | CK945_RS09085 | ACH97_218145 |
| TrkA family potassium uptake protein | TRNA_RS29760 | BL14DL4_04153 | BLDA23_09140 | CK945_RS09090 | ACH97_218140 |
| ribonuclease J | TRNA_RS29770 | BL14DL4_04155 | BLDA23_09150 | CK945_RS09100 | ACH97_218130 |
| DUF1447 family protein | TRNA_RS29775 | BL14DL4_04156 | BLDA23_09155 | CK945_RS09105 | ACH97_218125 |
| Cof-type HAD-IIB family hydrolase | TRNA_RS29780 | BL14DL4_04157 | BLDA23_09160 | CK945_RS09115 | ACH97_218115 |
| peptide deformylase | TRNA_RS29785 | BL14DL4_04159 | BLDA23_09170 | CK945_RS09120 | ACH97_218110 |
| hypothetical protein | TRNA_RS29795 | BL14DL4_04161 | BLDA23_09180 | CK945_RS09130 | ACH97_218105 |
| pyruvate dehydrogenase (acetyl-transferring) E1 component subunit alpha | TRNA_RS29800 | BL14DL4_04162 | BLDA23_09185 | CK945_RS09135 | ACH97_218100 |
| alpha-ketoacid dehydrogenase subunit beta | TRNA_RS29805 | BL14DL4_04163 | BLDA23_09190 | CK945_RS09140 | ACH97_218095 |
| 2-oxo acid dehydrogenase subunit E2 | TRNA_RS29810 | BL14DL4_04164 | BLDA23_09195 | CK945_RS09145 | ACH97_218090 |
| dihydrolipoyl dehydrogenase | TRNA_RS29815 | BL14DL4_04165 | BLDA23_09200 | CK945_RS09150 | ACH97_218085 |
| hypothetical protein | TRNA_RS29830 | BL14DL4_04167 | BLDA23_09205 | CK945_RS09155 | ACH97_218080 |
| aminotransferase class I/II-fold pyridoxal phosphate-dependent enzyme | TRNA_RS29835 | BL14DL4_04169 | BLDA23_09215 | CK945_RS09165 | ACH97_218075 |
| hypothetical protein | TRNA_RS29840 | BL14DL4_04170 | BLDA23_09220 | CK945_RS09175 | ACH97_218065 |
| DUF1054 domain-containing protein | TRNA_RS29845 | BL14DL4_04171 | BLDA23_09225 | CK945_RS09180 | ACH97_218060 |
| hypothetical protein | TRNA_RS29850 | BL14DL4_04172 | BLDA23_09230 | CK945_RS09185 | ACH97_218055 |
| inositol monophosphatase family protein | TRNA_RS29855 | BL14DL4_04173 | BLDA23_09235 | CK945_RS09190 | ACH97_218050 |
| GNAT family N-acetyltransferase | TRNA_RS29860 | BL14DL4_04174 | BLDA23_09240 | CK945_RS09195 | ACH97_218045 |
| hypothetical protein | TRNA_RS29885 | BL14DL4_04179 | BLDA23_09265 | CK945_RS09200 | ACH97_218020 |
| translational GTPase TypA | TRNA_RS29890 | BL14DL4_04180 | BLDA23_09270 | CK945_RS09205 | ACH97_218015 |
| membrane protein | TRNA_RS29895 | BL14DL4_04181 | BLDA23_09275 | CK945_RS09210 | ACH97_218010 |
| MarR family transcriptional regulator | TRNA_RS29900 | BL14DL4_04182 | BLDA23_09280 | CK945_RS09215 | ACH97_218005 |
| monooxygenase | TRNA_RS29905 | BL14DL4_04183 | BLDA23_09285 | CK945_RS09220 | ACH97_218000 |
| DUF2197 domain-containing protein | TRNA_RS29910 | BL14DL4_04184 | BLDA23_09290 | CK945_RS09225 | ACH97_217995 |
| YhcN/YlaJ family sporulation lipoprotein | TRNA_RS29915 | BL14DL4_04185 | BLDA23_09295 | CK945_RS09230 | ACH97_217990 |
| PhoH family protein | TRNA_RS29920 | BL14DL4_04186 | BLDA23_09300 | CK945_RS09235 | ACH97_217985 |
| hypothetical protein | TRNA_RS29925 | BL14DL4_04187 | BLDA23_09305 | CK945_RS09240 | ACH97_217980 |
| glutaminase A | TRNA_RS29930 | BL14DL4_04188 | BLDA23_09310 | CK945_RS09245 | ACH97_217975 |
| DUF1507 family protein | TRNA_RS29935 | BL14DL4_04189 | BLDA23_09315 | CK945_RS09250 | ACH97_217970 |
| FtsW/RodA/SpoVE family cell cycle protein | TRNA_RS29940 | BL14DL4_04190 | BLDA23_09320 | CK945_RS09255 | ACH97_217965 |
| pyruvate carboxylase | TRNA_RS29945 | BL14DL4_04191 | BLDA23_09325 | CK945_RS09260 | ACH97_217960 |
| heme A synthase | TRNA_RS29950 | BL14DL4_04192 | BLDA23_09330 | CK945_RS09265 | ACH97_217955 |
| protoheme IX farnesyltransferase | TRNA_RS29955 | BL14DL4_04193 | BLDA23_09335 | CK945_RS09270 | ACH97_217950 |
| cytochrome c oxidase subunit II | TRNA_RS29960 | BL14DL4_04194 | BLDA23_09340 | CK945_RS09275 | ACH97_217945 |
| cytochrome c oxidase subunit I | TRNA_RS29965 | BL14DL4_04195 | BLDA23_09345 | CK945_RS09280 | ACH97_217940 |
| cytochrome (ubi)quinol oxidase subunit III | TRNA_RS29970 | BL14DL4_04196 | BLDA23_09350 | CK945_RS09285 | ACH97_217935 |
| cytochrome c oxidase subunit IVB | TRNA_RS29975 | BL14DL4_04197 | BLDA23_09355 | CK945_RS09290 | ACH97_217930 |
| cytochrome c oxidase assembly factor CtaG | TRNA_RS29980 | BL14DL4_04198 | BLDA23_09360 | CK945_RS09295 | ACH97_217925 |
| hypothetical protein | TRNA_RS29985 | BL14DL4_04199 | BLDA23_09365 | CK945_RS09300 | ACH97_217920 |
| CBS domain-containing protein | TRNA_RS29990 | BL14DL4_04200 | BLDA23_09370 | CK945_RS09305 | ACH97_217915 |
| membrane protein | TRNA_RS29995 | BL14DL4_04201 | BLDA23_09375 | CK945_RS09310 | ACH97_217910 |
| hypothetical protein | TRNA_RS30000 | BL14DL4_04202 | BLDA23_09380 | CK945_RS09315 | ACH97_217905 |
| hypothetical protein | TRNA_RS30005 | BL14DL4_04203 | BLDA23_09385 | CK945_RS09320 | ACH97_217900 |
| YlbF family regulator | TRNA_RS30010 | BL14DL4_04204 | BLDA23_09390 | CK945_RS09325 | ACH97_217895 |
| DUF2129 domain-containing protein | TRNA_RS30015 | BL14DL4_04205 | BLDA23_09395 | CK945_RS09330 | ACH97_217890 |
| 16S rRNA (guanine(966)-N(2))-methyltransferase RsmD | TRNA_RS30020 | BL14DL4_04207 | BLDA23_09400 | CK945_RS09335 | ACH97_217885 |
| phosphopantetheine adenylyltransferase | TRNA_RS30025 | BL14DL4_04208 | BLDA23_09405 | CK945_RS09340 | ACH97_217880 |
| sporulation integral membrane protein YlbJ | TRNA_RS30030 | BL14DL4_04209 | BLDA23_09410 | CK945_RS09345 | ACH97_217875 |
| PDZ domain-containing protein | TRNA_RS30035 | BL14DL4_04210 | BLDA23_09415 | CK945_RS09350 | ACH97_217870 |
| nucleotidyltransferase | TRNA_RS30040 | BL14DL4_04211 | BLDA23_09420 | CK945_RS09355 | ACH97_217865 |
| DUF177 domain-containing protein | TRNA_RS30045 | BL14DL4_04212 | BLDA23_09425 | CK945_RS09360 | ACH97_217860 |
| RsfA family transcriptional regulator | TRNA_RS30050 | BL14DL4_04214 | BLDA23_09435 | CK945_RS09370 | ACH97_217855 |
| N-acetyltransferase | TRNA_RS30055 | BL14DL4_04215 | BLDA23_09440 | CK945_RS09375 | ACH97_217850 |
| 2-dehydropantoate 2-reductase | TRNA_RS30060 | BL14DL4_04216 | BLDA23_09445 | CK945_RS09380 | ACH97_217845 |
| bacillithiol biosynthesis cysteine-adding enzyme BshC | TRNA_RS30065 | BL14DL4_04217 | BLDA23_09450 | CK945_RS09385 | ACH97_217840 |
| transcriptional regulator MraZ | TRNA_RS30070 | BL14DL4_04218 | BLDA23_09455 | CK945_RS09390 | ACH97_217835 |
| 16S rRNA (cytosine(1402)-N(4))-methyltransferase | TRNA_RS30075 | BL14DL4_04219 | BLDA23_09460 | CK945_RS09395 | ACH97_217830 |
| cell division protein FtsL | TRNA_RS30080 | BL14DL4_04220 | BLDA23_09465 | CK945_RS09400 | ACH97_217825 |
| penicillin-binding protein | TRNA_RS30085 | BL14DL4_04221 | BLDA23_09470 | CK945_RS09405 | ACH97_217820 |
| stage V sporulation protein D | TRNA_RS30090 | BL14DL4_04222 | BLDA23_09475 | CK945_RS09410 | ACH97_217815 |
| UDP-N-acetylmuramoyl-L-alanyl-D-glutamate--2, 6-diaminopimelate ligase | TRNA_RS30095 | BL14DL4_04224 | BLDA23_09480 | CK945_RS09415 | ACH97_217810 |
| phospho-N-acetylmuramoyl-pentapeptide- transferase | TRNA_RS30100 | BL14DL4_04225 | BLDA23_09485 | CK945_RS09420 | ACH97_217805 |
| UDP-N-acetylmuramoyl-L-alanine--D-glutamate ligase | TRNA_RS30105 | BL14DL4_04226 | BLDA23_09490 | CK945_RS09425 | ACH97_217800 |
| stage V sporulation protein E | TRNA_RS30110 | BL14DL4_04227 | BLDA23_09495 | CK945_RS09430 | ACH97_217795 |
| undecaprenyldiphospho-muramoylpentapeptide beta-N-acetylglucosaminyltransferase | TRNA_RS30115 | BL14DL4_04228 | BLDA23_09500 | CK945_RS09435 | ACH97_217790 |
| UDP-N-acetylenolpyruvoylglucosamine reductase | TRNA_RS30120 | BL14DL4_04229 | BLDA23_09505 | CK945_RS09440 | ACH97_217785 |
| cell division protein FtsQ/DivIB | TRNA_RS30125 | BL14DL4_04230 | BLDA23_09510 | CK945_RS09445 | ACH97_217780 |
| DUF881 domain-containing protein | TRNA_RS30130 | BL14DL4_04231 | BLDA23_09515 | CK945_RS09450 | ACH97_217775 |
| DUF881 domain-containing protein | TRNA_RS30135 | BL14DL4_04232 | BLDA23_09520 | CK945_RS09455 | ACH97_217770 |
| DUF1290 domain-containing protein | TRNA_RS30140 | BL14DL4_04233 | BLDA23_09525 | CK945_RS09460 | ACH97_217765 |
| cell division protein FtsA | TRNA_RS30145 | BL14DL4_04234 | BLDA23_09530 | CK945_RS09465 | ACH97_217760 |
| cell division protein FtsZ | TRNA_RS30150 | BL14DL4_04235 | BLDA23_09535 | CK945_RS09470 | ACH97_217755 |
| peptidase S8 | TRNA_RS30155 | BL14DL4_04236 | BLDA23_09540 | CK945_RS09475 | ACH97_217750 |
| sigma-E processing peptidase SpoIIGA | TRNA_RS30165 | BL14DL4_04238 | BLDA23_09555 | CK945_RS09485 | ACH97_206060 |
| RNA polymerase sporulation sigma factor SigE | TRNA_RS30170 | BL14DL4_04239 | BLDA23_09560 | CK945_RS09490 | ACH97_206065 |
| RNA polymerase sporulation sigma factor SigG | TRNA_RS30175 | BL14DL4_04240 | BLDA23_09565 | CK945_RS09495 | ACH97_206070 |
| methyltransferase domain-containing protein | TRNA_RS30190 | BL14DL4_04242 | BLDA23_09575 | CK945_RS09505 | ACH97_206080 |
| YlmC/YmxH family sporulation protein | TRNA_RS30195 | BL14DL4_04243 | BLDA23_09580 | CK945_RS09510 | ACH97_206085 |
| peptidoglycan editing factor PgeF | TRNA_RS30200 | BL14DL4_04244 | BLDA23_09585 | CK945_RS09515 | ACH97_206090 |
| YggS family pyridoxal phosphate-dependent enzyme | TRNA_RS30205 | BL14DL4_04245 | BLDA23_09590 | CK945_RS09520 | ACH97_206095 |
| cell division protein SepF | TRNA_RS30210 | BL14DL4_04246 | BLDA23_09595 | CK945_RS09525 | ACH97_206100 |
| YggT family protein | TRNA_RS30215 | BL14DL4_04247 | BLDA23_09600 | CK945_RS09530 | ACH97_206105 |
| RNA-binding protein S4 | TRNA_RS30220 | BL14DL4_04248 | BLDA23_09605 | CK945_RS09535 | ACH97_206110 |
| DivIVA domain-containing protein | TRNA_RS30225 | BL14DL4_04249 | BLDA23_09610 | CK945_RS09540 | ACH97_206115 |
| isoleucine--tRNA ligase | TRNA_RS30230 | BL14DL4_04250 | BLDA23_09615 | CK945_RS09545 | ACH97_206120 |
| hypothetical protein | TRNA_RS30235 | BL14DL4_04251 | BLDA23_09620 | CK945_RS09550 | ACH97_206125 |
| lipoprotein signal peptidase | TRNA_RS30240 | BL14DL4_04252 | BLDA23_09625 | CK945_RS09555 | ACH97_206130 |
| RluA family pseudouridine synthase | TRNA_RS30245 | BL14DL4_04253 | BLDA23_09630 | CK945_RS09560 | ACH97_206135 |
| bifunctional pyr operon transcriptional regulator/uracil phosphoribosyltransferase PyrR | TRNA_RS30250 | BL14DL4_04254 | BLDA23_09635 | CK945_RS09565 | ACH97_206140 |
| uracil transporter | TRNA_RS30255 | BL14DL4_04255 | BLDA23_09640 | CK945_RS09570 | ACH97_206145 |
| aspartate carbamoyltransferase | TRNA_RS30260 | BL14DL4_04256 | BLDA23_09645 | CK945_RS09575 | ACH97_206150 |
| dihydroorotase | TRNA_RS30265 | BL14DL4_04257 | BLDA23_09650 | CK945_RS09580 | ACH97_206155 |
| carbamoyl-phosphate synthase small subunit | TRNA_RS30270 | BL14DL4_04258 | BLDA23_09655 | CK945_RS09585 | ACH97_206160 |
| carbamoyl-phosphate synthase large subunit | TRNA_RS30275 | BL14DL4_04259 | BLDA23_09660 | CK945_RS09590 | ACH97_206165 |
| dihydroorotate dehydrogenase electron transfer subunit | TRNA_RS30280 | BL14DL4_04260 | BLDA23_09665 | CK945_RS09595 | ACH97_206170 |
| dihydroorotate dehydrogenase | TRNA_RS30285 | BL14DL4_04261 | BLDA23_09670 | CK945_RS09600 | ACH97_206175 |
| orotidine-5-phosphate decarboxylase | TRNA_RS30290 | BL14DL4_04262 | BLDA23_09675 | CK945_RS09605 | ACH97_206180 |
| orotate phosphoribosyltransferase | TRNA_RS30295 | BL14DL4_04263 | BLDA23_09680 | CK945_RS09610 | ACH97_206185 |
| phosphoadenylyl-sulfate reductase | TRNA_RS30300 | BL14DL4_04265 | BLDA23_09690 | CK945_RS09620 | ACH97_206190 |
| inorganic phosphate transporter | TRNA_RS30305 | BL14DL4_04266 | BLDA23_09695 | CK945_RS09625 | ACH97_206195 |
| sulfate adenylyltransferase | TRNA_RS30310 | BL14DL4_04267 | BLDA23_09700 | CK945_RS09630 | ACH97_206200 |
| adenylyl-sulfate kinase | TRNA_RS30315 | BL14DL4_04268 | BLDA23_09705 | CK945_RS09635 | ACH97_206205 |
| uroporphyrinogen-III C-methyltransferase | TRNA_RS30320 | BL14DL4_04269 | BLDA23_09710 | CK945_RS09640 | ACH97_206210 |
| sirohydrochlorin chelatase | TRNA_RS30325 | BL14DL4_04270 | BLDA23_09715 | CK945_RS09645 | ACH97_206215 |
| precorrin-2 dehydrogenase | TRNA_RS30330 | BL14DL4_04271 | BLDA23_09720 | CK945_RS09650 | ACH97_206220 |
| fibronectin/fibrinogen-binding protein | TRNA_RS30335 | BL14DL4_04272 | BLDA23_09725 | CK945_RS09655 | ACH97_206225 |
| calcium-translocating P-type ATPase, SERCA-type | TRNA_RS30340 | BL14DL4_04273 | BLDA23_09730 | CK945_RS09660 | ACH97_206230 |
| YicC family protein | TRNA_RS30345 | BL14DL4_04274 | BLDA23_09735 | CK945_RS09665 | ACH97_206235 |
| DUF370 domain-containing protein | TRNA_RS30350 | BL14DL4_04275 | BLDA23_09740 | CK945_RS09670 | ACH97_206240 |
| guanylate kinase | TRNA_RS30355 | BL14DL4_04276 | BLDA23_09745 | CK945_RS09675 | ACH97_206245 |
| DNA-directed RNA polymerase subunit omega | TRNA_RS30360 | BL14DL4_04277 | BLDA23_09750 | CK945_RS09680 | ACH97_206250 |
| bifunctional phosphopantothenoylcysteine decarboxylase/phosphopantothenate--cysteine ligase CoaBC | TRNA_RS30365 | BL14DL4_04278 | BLDA23_09755 | CK945_RS09685 | ACH97_206255 |
| primosomal protein N | TRNA_RS30370 | BL14DL4_04279 | BLDA23_09760 | CK945_RS09690 | ACH97_206260 |
| peptide deformylase | TRNA_RS30375 | BL14DL4_04280 | BLDA23_09765 | CK945_RS09695 | ACH97_206265 |
| methionyl-tRNA formyltransferase | TRNA_RS30380 | BL14DL4_04281 | BLDA23_09770 | CK945_RS09700 | ACH97_206270 |
| 16S rRNA (cytosine(967)-C(5))-methyltransferase RsmB | TRNA_RS30385 | BL14DL4_04282 | BLDA23_09775 | CK945_RS09705 | ACH97_206275 |
| 23S rRNA (adenine(2503)-C(2))-methyltransferase RlmN | TRNA_RS30390 | BL14DL4_04283 | BLDA23_09780 | CK945_RS09710 | ACH97_206280 |
| Stp1/IreP family PP2C-type Ser/Thr phosphatase | TRNA_RS30395 | BL14DL4_04284 | BLDA23_09785 | CK945_RS09715 | ACH97_206285 |
| Stk1 family PASTA domain-containing Ser/Thr kinase | TRNA_RS30400 | BL14DL4_04285 | BLDA23_09790 | CK945_RS09720 | ACH97_206290 |
| ribosome small subunit-dependent GTPase A | TRNA_RS30405 | BL14DL4_04286 | BLDA23_09795 | CK945_RS09725 | ACH97_206295 |
| ribulose-phosphate 3-epimerase | TRNA_RS30410 | BL14DL4_04287 | BLDA23_09800 | CK945_RS09730 | ACH97_206300 |
| thiamine diphosphokinase | TRNA_RS30415 | BL14DL4_04288 | BLDA23_09805 | CK945_RS09735 | ACH97_206305 |
| 50S ribosomal protein L28 | TRNA_RS30425 | BL14DL4_04289 | BLDA23_09815 | CK945_RS09745 | ACH97_206315 |
| Asp23/Gls24 family envelope stress response protein | TRNA_RS30430 | BL14DL4_04290 | BLDA23_09820 | CK945_RS09750 | ACH97_206320 |
| DAK2 domain-containing protein | TRNA_RS30435 | BL14DL4_04291 | BLDA23_09825 | CK945_RS09755 | ACH97_206325 |
| L-serine ammonia-lyase, iron-sulfur-dependent, subunit beta | TRNA_RS30440 | BL14DL4_04292 | BLDA23_09830 | CK945_RS09760 | ACH97_206330 |
| L-serine ammonia-lyase, iron-sulfur-dependent, subunit alpha | TRNA_RS30445 | BL14DL4_04293 | BLDA23_09835 | CK945_RS09765 | ACH97_206335 |
| ATP-dependent DNA helicase RecG | TRNA_RS30450 | BL14DL4_04294 | BLDA23_09840 | CK945_RS09770 | ACH97_206340 |
| transcription factor FapR | TRNA_RS30455 | BL14DL4_04295 | BLDA23_09845 | CK945_RS09775 | ACH97_206345 |
| phosphate acyltransferase | TRNA_RS30460 | BL14DL4_04296 | BLDA23_09850 | CK945_RS09780 | ACH97_206350 |
| [acyl-carrier-protein] S-malonyltransferase | TRNA_RS30465 | BL14DL4_04297 | BLDA23_09855 | CK945_RS09785 | ACH97_206355 |
| 3-oxoacyl-[acyl-carrier-protein] reductase | TRNA_RS30470 | BL14DL4_04298 | BLDA23_09860 | CK945_RS09790 | ACH97_206360 |
| acyl carrier protein | TRNA_RS30475 | BL14DL4_04299 | BLDA23_09865 | CK945_RS09795 | ACH97_206365 |
| ribonuclease 3 | TRNA_RS30480 | BL14DL4_04300 | BLDA23_09870 | CK945_RS09800 | ACH97_206370 |
| chromosome segregation protein SMC | TRNA_RS30485 | BL14DL4_04301 | BLDA23_09875 | CK945_RS09805 | ACH97_206375 |
| signal recognition particle-docking protein FtsY | TRNA_RS30490 | BL14DL4_04302 | BLDA23_09880 | CK945_RS09810 | ACH97_206380 |
| putative DNA-binding protein | TRNA_RS30495 | BL14DL4_04303 | BLDA23_09885 | CK945_RS09815 | ACH97_206385 |
| signal recognition particle protein | TRNA_RS30500 | BL14DL4_04304 | BLDA23_09890 | CK945_RS09820 | ACH97_206390 |
| 30S ribosomal protein S16 | TRNA_RS30505 | BL14DL4_04305 | BLDA23_09895 | CK945_RS09825 | ACH97_206395 |
| KH domain-containing protein | TRNA_RS30510 | BL14DL4_04306 | BLDA23_09900 | CK945_RS09830 | ACH97_206400 |
| hypothetical protein | TRNA_RS30515 | BL14DL4_04307 | BLDA23_09905 | CK945_RS09835 | ACH97_206405 |
| ribosome maturation factor RimM | TRNA_RS30520 | BL14DL4_04308 | BLDA23_09910 | CK945_RS09840 | ACH97_206410 |
| tRNA (guanosine(37)-N1)-methyltransferase TrmD | TRNA_RS30525 | BL14DL4_04309 | BLDA23_09915 | CK945_RS09845 | ACH97_206415 |
| 50S ribosomal protein L19 | TRNA_RS30530 | BL14DL4_04310 | BLDA23_09920 | CK945_RS09850 | ACH97_206420 |
| ribosome biogenesis GTPase A | TRNA_RS30535 | BL14DL4_04311 | BLDA23_09925 | CK945_RS09855 | ACH97_206425 |
| ribonuclease HII | TRNA_RS30540 | BL14DL4_04312 | BLDA23_09930 | CK945_RS09860 | ACH97_206430 |
| hypothetical protein | TRNA_RS30545 | BL14DL4_04313 | BLDA23_09935 | CK945_RS09865 | ACH97_206435 |
| FlhB-like flagellar biosynthesis protein | TRNA_RS30550 | BL14DL4_04314 | BLDA23_09940 | CK945_RS09870 | ACH97_206440 |
| succinyl-CoA ligase subunit beta | TRNA_RS30555 | BL14DL4_04315 | BLDA23_09945 | CK945_RS09875 | ACH97_206445 |
| succinate--CoA ligase subunit alpha | TRNA_RS30560 | BL14DL4_04316 | BLDA23_09950 | CK945_RS09880 | ACH97_206450 |
| DNA-protecting protein DprA | TRNA_RS30565 | BL14DL4_04317 | BLDA23_09955 | CK945_RS09885 | ACH97_206455 |
| type I DNA topoisomerase | TRNA_RS30570 | BL14DL4_04318 | BLDA23_09960 | CK945_RS09890 | ACH97_206460 |
| FADH(2)-oxidizing methylenetetrahydrofolate--tRNA-(uracil(54)-C(5))- methyltransferase TrmFO | TRNA_RS30575 | BL14DL4_04319 | BLDA23_09965 | CK945_RS09895 | ACH97_206465 |
| tyrosine recombinase XerC | TRNA_RS30580 | BL14DL4_04320 | BLDA23_09970 | CK945_RS09900 | ACH97_206470 |
| ATP-dependent protease subunit HslV | TRNA_RS30585 | BL14DL4_04321 | BLDA23_09975 | CK945_RS09905 | ACH97_206475 |
| HslU--HslV peptidase ATPase subunit | TRNA_RS30590 | BL14DL4_04322 | BLDA23_09980 | CK945_RS09910 | ACH97_206480 |
| GTP-sensing pleiotropic transcriptional regulator CodY | TRNA_RS30595 | BL14DL4_04323 | BLDA23_09985 | CK945_RS09915 | ACH97_206485 |
| flagellar basal body rod protein FlgB | TRNA_RS30600 | BL14DL4_04325 | BLDA23_09990 | CK945_RS09920 | ACH97_206490 |
| flagellar basal body rod protein FlgC | TRNA_RS30605 | BL14DL4_04326 | BLDA23_09995 | CK945_RS09925 | ACH97_206495 |
| flagellar hook-basal body complex protein FliE | TRNA_RS30610 | BL14DL4_04327 | BLDA23_10000 | CK945_RS09930 | ACH97_206500 |
| flagellar basal body M-ring protein FliF | TRNA_RS30615 | BL14DL4_04328 | BLDA23_10005 | CK945_RS09935 | ACH97_206505 |
| flagellar motor switch protein FliG | TRNA_RS30620 | BL14DL4_04329 | BLDA23_10010 | CK945_RS09940 | ACH97_206510 |
| flagellar assembly protein FliH | TRNA_RS30625 | BL14DL4_04330 | BLDA23_10015 | CK945_RS09945 | ACH97_206515 |
| flagellar protein export ATPase FliI | TRNA_RS30630 | BL14DL4_04331 | BLDA23_10020 | CK945_RS09950 | ACH97_206520 |
| flagellar biosynthesis chaperone FliJ | TRNA_RS30635 | BL14DL4_04332 | BLDA23_10025 | CK945_RS09955 | ACH97_206525 |
| flagellar hook-length control protein FliK | TRNA_RS30645 | BL14DL4_04334 | BLDA23_10035 | CK945_RS09965 | ACH97_206535 |
| flagellar hook assembly protein FlgD | TRNA_RS30650 | BL14DL4_04335 | BLDA23_10040 | CK945_RS09970 | ACH97_206540 |
| flagellar basal body rod protein FlgG | TRNA_RS30655 | BL14DL4_04336 | BLDA23_10045 | CK945_RS09975 | ACH97_206545 |
| flagellar basal body-associated protein FliL | TRNA_RS30665 | BL14DL4_04337 | BLDA23_10055 | CK945_RS09985 | ACH97_206555 |
| flagellar motor switch protein FliM | TRNA_RS30670 | BL14DL4_04338 | BLDA23_10060 | CK945_RS09990 | ACH97_206560 |
| flagellar motor switch phosphatase FliY | TRNA_RS30675 | BL14DL4_04339 | BLDA23_10065 | CK945_RS09995 | ACH97_206565 |
| response regulator | TRNA_RS30680 | BL14DL4_04340 | BLDA23_10070 | CK945_RS10000 | ACH97_206570 |
| flagella biosynthesis protein FliZ | TRNA_RS30685 | BL14DL4_04341 | BLDA23_10075 | CK945_RS10005 | ACH97_206575 |
| flagellar biosynthetic protein FliP | TRNA_RS30690 | BL14DL4_04342 | BLDA23_10080 | CK945_RS10010 | ACH97_206580 |
| flagellar biosynthetic protein FliQ | TRNA_RS30695 | BL14DL4_04343 | BLDA23_10085 | CK945_RS10015 | ACH97_206585 |
| flagellar type III secretion system protein FliR | TRNA_RS30700 | BL14DL4_04344 | BLDA23_10090 | CK945_RS10020 | ACH97_206590 |
| flagellar biosynthesis protein FlhB | TRNA_RS30705 | BL14DL4_04345 | BLDA23_10095 | CK945_RS10025 | ACH97_206595 |
| flagellar biosynthesis protein FlhA | TRNA_RS30710 | BL14DL4_04346 | BLDA23_10100 | CK945_RS10030 | ACH97_206600 |
| flagellar biosynthesis protein FlhF | TRNA_RS30715 | BL14DL4_04347 | BLDA23_10105 | CK945_RS10035 | ACH97_206605 |
| MinD/ParA family protein | TRNA_RS30720 | BL14DL4_04348 | BLDA23_10110 | CK945_RS10040 | ACH97_206610 |
| chemotaxis response regulator protein-glutamate methylesterase | TRNA_RS30725 | BL14DL4_04349 | BLDA23_10115 | CK945_RS10045 | ACH97_206615 |
| chemotaxis protein CheA | TRNA_RS30730 | BL14DL4_04350 | BLDA23_10120 | CK945_RS10050 | ACH97_206620 |
| chemotaxis protein CheW | TRNA_RS30735 | BL14DL4_04351 | BLDA23_10125 | CK945_RS10055 | ACH97_206625 |
| chemotaxis protein CheC | TRNA_RS30740 | BL14DL4_04352 | BLDA23_10130 | CK945_RS10060 | ACH97_206630 |
| chemoreceptor glutamine deamidase CheD | TRNA_RS30745 | BL14DL4_04353 | BLDA23_10135 | CK945_RS10065 | ACH97_206635 |
| hypothetical protein | TRNA_RS30755 | BL14DL4_04355 | BLDA23_10145 | CK945_RS10075 | ACH97_206645 |
| 30S ribosomal protein S2 | TRNA_RS30760 | BL14DL4_04356 | BLDA23_10150 | CK945_RS10080 | ACH97_206650 |
| elongation factor Ts | TRNA_RS30765 | BL14DL4_04357 | BLDA23_10155 | CK945_RS10085 | ACH97_206655 |
| UMP kinase | TRNA_RS30770 | BL14DL4_04358 | BLDA23_10160 | CK945_RS10090 | ACH97_206660 |
| ribosome recycling factor | TRNA_RS30775 | BL14DL4_04359 | BLDA23_10165 | CK945_RS10095 | ACH97_206665 |
| isoprenyl transferase | TRNA_RS30780 | BL14DL4_04360 | BLDA23_10170 | CK945_RS10100 | ACH97_206670 |
| phosphatidate cytidylyltransferase | TRNA_RS30785 | BL14DL4_04361 | BLDA23_10175 | CK945_RS10105 | ACH97_206675 |
| 1-deoxy-D-xylulose-5-phosphate reductoisomerase | TRNA_RS30790 | BL14DL4_04362 | BLDA23_10180 | CK945_RS10110 | ACH97_206680 |
| RIP metalloprotease RseP | TRNA_RS30795 | BL14DL4_04363 | BLDA23_10185 | CK945_RS10115 | ACH97_206685 |
| proline--tRNA ligase | TRNA_RS30800 | BL14DL4_04364 | BLDA23_10190 | CK945_RS10120 | ACH97_206690 |
| glycoside hydrolase | TRNA_RS30820 | BL14DL4_04367 | BLDA23_10210 | CK945_RS10135 | ACH97_206705 |
| glycoside hydrolase | TRNA_RS30830 | BL14DL4_04369 | BLDA23_10220 | CK945_RS10145 | ACH97_206715 |
| ribosome maturation factor RimP | TRNA_RS30835 | BL14DL4_04370 | BLDA23_10225 | CK945_RS10150 | ACH97_206720 |
| transcription termination/antitermination protein NusA | TRNA_RS30840 | BL14DL4_04371 | BLDA23_10230 | CK945_RS10155 | ACH97_206725 |
| YlxR family protein | TRNA_RS30845 | BL14DL4_04372 | BLDA23_10235 | CK945_RS10160 | ACH97_206730 |
| YlxQ family RNA-binding protein | TRNA_RS30850 | BL14DL4_04373 | BLDA23_10240 | CK945_RS10165 | ACH97_206735 |
| translation initiation factor IF-2 | TRNA_RS30855 | BL14DL4_04374 | BLDA23_10245 | CK945_RS10170 | ACH97_206740 |
| DUF503 domain-containing protein | TRNA_RS30860 | BL14DL4_04375 | BLDA23_10250 | CK945_RS10175 | ACH97_206745 |
| 30S ribosome-binding factor RbfA | TRNA_RS30865 | BL14DL4_04376 | BLDA23_10255 | CK945_RS10180 | ACH97_206750 |
| tRNA pseudouridine(55) synthase TruB | TRNA_RS30870 | BL14DL4_04377 | BLDA23_10260 | CK945_RS10185 | ACH97_206755 |
| bifunctional riboflavin kinase/FAD synthetase | TRNA_RS30875 | BL14DL4_04378 | BLDA23_10265 | CK945_RS10190 | ACH97_206760 |
| 30S ribosomal protein S15 | TRNA_RS30880 | BL14DL4_04379 | BLDA23_10270 | CK945_RS10195 | ACH97_206765 |
| polyribonucleotide nucleotidyltransferase | TRNA_RS30885 | BL14DL4_04380 | BLDA23_10275 | CK945_RS10200 | ACH97_206770 |
| hypothetical protein | TRNA_RS30890 | BL14DL4_04381 | BLDA23_10280 | CK945_RS10205 | ACH97_206775 |
| insulinase family protein | TRNA_RS30895 | BL14DL4_04382 | BLDA23_10285 | CK945_RS10210 | ACH97_206780 |
| YlmC/YmxH family sporulation protein | TRNA_RS30900 | BL14DL4_04383 | BLDA23_10290 | CK945_RS10215 | ACH97_206785 |
| dipicolinic acid synthetase subunit A | TRNA_RS30905 | BL14DL4_04384 | BLDA23_10295 | CK945_RS10220 | ACH97_206790 |
| dipicolinate synthase subunit B | TRNA_RS30910 | BL14DL4_04385 | BLDA23_10300 | CK945_RS10225 | ACH97_206795 |
| aspartate-semialdehyde dehydrogenase | TRNA_RS30915 | BL14DL4_04386 | BLDA23_10305 | CK945_RS10230 | ACH97_206800 |
| aspartate kinase | TRNA_RS30920 | BL14DL4_04387 | BLDA23_10310 | CK945_RS10235 | ACH97_206805 |
| 4-hydroxy-tetrahydrodipicolinate synthase | TRNA_RS30925 | BL14DL4_04388 | BLDA23_10315 | CK945_RS10240 | ACH97_206810 |
| ribonuclease J | TRNA_RS30930 | BL14DL4_04389 | BLDA23_10320 | CK945_RS10245 | ACH97_206815 |
| translocation-enhancing protein TepA | TRNA_RS30935 | BL14DL4_04390 | BLDA23_10325 | CK945_RS10250 | ACH97_206820 |
| hypothetical protein | TRNA_RS30940 | BL14DL4_04391 | BLDA23_10330 | CK945_RS10255 | ACH97_206825 |
| DNA translocase FtsK | TRNA_RS30945 | BL14DL4_04392 | BLDA23_10335 | CK945_RS10260 | ACH97_206830 |
| GntR family transcriptional regulator | TRNA_RS30950 | BL14DL4_04393 | BLDA23_10340 | CK945_RS10265 | ACH97_206835 |
| MFS transporter | TRNA_RS30955 | BL14DL4_04394 | BLDA23_10345 | CK945_RS10270 | ACH97_206840 |
| insulinase family protein | TRNA_RS30960 | BL14DL4_04395 | BLDA23_10350 | CK945_RS10275 | ACH97_206845 |
| insulinase family protein | TRNA_RS30965 | BL14DL4_04396 | BLDA23_10355 | CK945_RS10280 | ACH97_206850 |
| SDR family oxidoreductase | TRNA_RS30970 | BL14DL4_04397 | BLDA23_10360 | CK945_RS10285 | ACH97_206855 |
| DUF3243 domain-containing protein | TRNA_RS30975 | BL14DL4_04398 | BLDA23_10365 | CK945_RS10290 | ACH97_206860 |
| DUF3388 domain-containing protein | TRNA_RS30980 | BL14DL4_04399 | BLDA23_10370 | CK945_RS10295 | ACH97_206865 |
| helix-turn-helix domain-containing protein | TRNA_RS30985 | BL14DL4_04400 | BLDA23_10375 | CK945_RS10300 | ACH97_206870 |
| CDP-diacylglycerol--glycerol-3-phosphate 3-phosphatidyltransferase | TRNA_RS30990 | BL14DL4_04401 | BLDA23_10380 | CK945_RS10305 | ACH97_206875 |
| competence/damage-inducible protein A | TRNA_RS30995 | BL14DL4_04402 | BLDA23_10385 | CK945_RS10310 | ACH97_206880 |
| recombinase RecA | TRNA_RS31000 | BL14DL4_04403 | BLDA23_10390 | CK945_RS10315 | ACH97_206885 |
| ribonuclease Y | TRNA_RS31005 | BL14DL4_04404 | BLDA23_10395 | CK945_RS10320 | ACH97_206890 |
| TIGR00282 family metallophosphoesterase | TRNA_RS31010 | BL14DL4_04405 | BLDA23_10400 | CK945_RS10325 | ACH97_206895 |
| stage V sporulation protein S | TRNA_RS31015 | BL14DL4_04406 | BLDA23_10405 | CK945_RS10330 | ACH97_206900 |
| membrane dipeptidase | TRNA_RS31020 | BL14DL4_04407 | BLDA23_10410 | CK945_RS10335 | ACH97_206905 |
| L-threonine 3-dehydrogenase | TRNA_RS31025 | BL14DL4_04408 | BLDA23_10415 | CK945_RS10340 | ACH97_206910 |
| 8-amino-7-oxononanoate synthase | TRNA_RS31030 | BL14DL4_04409 | BLDA23_10420 | CK945_RS10345 | ACH97_206915 |
| tRNA (N6-isopentenyl adenosine(37)-C2)-methylthiotransferase MiaB | TRNA_RS31035 | BL14DL4_04410 | BLDA23_10425 | CK945_RS10350 | ACH97_206920 |
| hypothetical protein | TRNA_RS31040 | BL14DL4_04411 | BLDA23_10430 | CK945_RS10355 | ACH97_206925 |
| outer spore coat protein CotE | TRNA_RS31045 | BL14DL4_04412 | BLDA23_10435 | CK945_RS10360 | ACH97_206930 |
| DNA mismatch repair protein MutS | TRNA_RS31050 | BL14DL4_04413 | BLDA23_10440 | CK945_RS10365 | ACH97_206935 |
| DNA mismatch repair endonuclease MutL | TRNA_RS31055 | BL14DL4_04414 | BLDA23_10445 | CK945_RS10370 | ACH97_206940 |
| antibiotic biosynthesis monooxygenase | TRNA_RS31060 | BL14DL4_04415 | BLDA23_10450 | CK945_RS10375 | ACH97_206945 |
| GTP-binding protein | TRNA_RS31080 | BL14DL4_04419 | BLDA23_10475 | CK945_RS10415 | ACH97_206975 |
| (2Fe-2S) ferredoxin domain-containing protein | TRNA_RS31085 | BL14DL4_04420 | BLDA23_10480 | CK945_RS10420 | ACH97_206980 |
| DUF1275 domain-containing protein | TRNA_RS31105 | BL14DL4_04425 | BLDA23_10505 | CK945_RS10440 | ACH97_206995 |
| potassium/proton antiporter | TRNA_RS31110 | BL14DL4_04426 | BLDA23_10510 | CK945_RS10445 | ACH97_207000 |
| YbjQ family protein | TRNA_RS31125 | BL14DL4_044430 | BLDA23_10530 | CK945_RS10460 | ACH97_207015 |
| TVP38/TMEM64 family protein | TRNA_RS31130 | BL14DL4_04431 | BLDA23_10535 | CK945_RS10465 | ACH97_207020 |
| UDP-glucosyltransferase | TRNA_RS31140 | BL14DL4_04433 | BLDA23_10545 | CK945_RS10475 | ACH97_207030 |
| APC family permease | TRNA_RS31145 | BL14DL4_04434 | BLDA23_10550 | CK945_RS10480 | ACH97_207035 |
| spore coat protein | TRNA_RS31150 | BL14DL4_04435 | BLDA23_10555 | CK945_RS10485 | ACH97_207040 |
| hypothetical protein | TRNA_RS31155 | BL14DL4_04436 | BLDA23_10560 | CK945_RS10490 | ACH97_207045 |
| LysM peptidoglycan-binding domain-containing protein | TRNA_RS31160 | BL14DL4_04437 | BLDA23_10565 | CK945_RS10495 | ACH97_207050 |
| TetR/AcrR family transcriptional regulator | TRNA_RS31165 | BL14DL4_04438 | BLDA23_10570 | CK945_RS10500 | ACH97_207055 |
| nuclear transport factor 2 family protein | TRNA_RS31170 | BL14DL4_04439 | BLDA23_10575 | CK945_RS10505 | ACH97_207060 |
| hypothetical protein | TRNA_RS31180 | BL14DL4_04441 | BLDA23_10585 | CK945_RS10515 | ACH97_207070 |
| OsmC family peroxiredoxin | TRNA_RS31185 | BL14DL4_04442 | BLDA23_10590 | CK945_RS10520 | ACH97_207075 |
| QacE family quaternary ammonium compound efflux SMR transporter | TRNA_RS31190 | BL14DL4_04443 | BLDA23_10595 | CK945_RS10525 | ACH97_207080 |
| QacE family quaternary ammonium compound efflux SMR transporter | TRNA_RS31195 | BL14DL4_04444 | BLDA23_10600 | CK945_RS10530 | ACH97_207085 |
| hypothetical protein | TRNA_RS31200 | BL14DL4_04445 | BLDA23_10605 | CK945_RS10535 | ACH97_207090 |
| hypothetical protein | TRNA_RS31205 | BL14DL4_04446 | BLDA23_10610 | CK945_RS10540 | ACH97_207095 |
| tRNA (adenosine(37)-N6)-dimethylallyltransferase MiaA | TRNA_RS31210 | BL14DL4_04447 | BLDA23_10615 | CK945_RS10545 | ACH97_207100 |
| RNA-binding protein Hfq | TRNA_RS31215 | BL14DL4_04448 | BLDA23_10620 | CK945_RS10550 | ACH97_207105 |
| hypothetical protein | TRNA_RS31225 | BL14DL4_04449 | BLDA23_10630 | CK945_RS10560 | ACH97_207110 |
| class Ib ribonucleoside-diphosphate reductase assembly flavoprotein NrdI | TRNA_RS31230 | BL14DL4_04450 | BLDA23_10635 | CK945_RS10565 | ACH97_207115 |
| class 1b ribonucleoside-diphosphate reductase subunit alpha | TRNA_RS31235 | BL14DL4_04451 | BLDA23_10640 | CK945_RS10570 | ACH97_207120 |
| class 1b ribonucleoside-diphosphate reductase subunit beta | TRNA_RS31240 | BL14DL4_04452 | BLDA23_10645 | CK945_RS10575 | ACH97_207125 |
| hypothetical protein | TRNA_RS31245 | BL14DL4_04453 | BLDA23_10650 | CK945_RS10580 | ACH97_207130 |
| SUF system NifU family Fe-S cluster assembly protein | TRNA_RS31255 | BL14DL4_04455 | BLDA23_10660 | CK945_RS10590 | ACH97_207140 |
| transcriptional repressor | TRNA_RS31260 | BL14DL4_04456 | BLDA23_10665 | CK945_RS10595 | ACH97_207145 |
| GntR family transcriptional regulator | TRNA_RS31265 | BL14DL4_04457 | BLDA23_10670 | CK945_RS10600 | ACH97_207150 |
| multidrug resistance efflux transporter family protein | TRNA_RS31270 | BL14DL4_04458 | BLDA23_10675 | CK945_RS10605 | ACH97_207155 |
| DMT family transporter | TRNA_RS31280 | BL14DL4_04460 | BLDA23_10690 | CK945_RS10620 | ACH97_207165 |
| DMT family transporter | TRNA_RS31285 | BL14DL4_04461 | BLDA23_10695 | CK945_RS10625 | ACH97_207170 |
| hypothetical protein | TRNA_RS31295 | BL14DL4_04463 | BLDA23_10705 | CK945_RS10630 | ACH97_207175 |
| TetR/AcrR family transcriptional regulator | TRNA_RS31300 | BL14DL4_04464 | BLDA23_10710 | CK945_RS10635 | ACH97_207180 |
| OsmC family peroxiredoxin | TRNA_RS31305 | BL14DL4_04465 | BLDA23_10715 | CK945_RS10640 | ACH97_207185 |
| cytosine permease | TRNA_RS31310 | BL14DL4_04466 | BLDA23_10720 | CK945_RS10645 | ACH97_207190 |
| hydantoinase/oxoprolinase family protein | TRNA_RS31315 | BL14DL4_04467 | BLDA23_10725 | CK945_RS10650 | ACH97_207195 |
| N-methylhydantoinase | TRNA_RS31320 | BL14DL4_04468 | BLDA23_10730 | CK945_RS10655 | ACH97_207200 |
| hydantoinase B/oxoprolinase family protein | TRNA_RS31325 | BL14DL4_04469 | BLDA23_10735 | CK945_RS10660 | ACH97_207205 |
| DUF2243 domain-containing protein | TRNA_RS31330 | BL14DL4_04470 | BLDA23_10740 | CK945_RS10665 | ACH97_207210 |
| hypothetical protein | TRNA_RS31345 | BL14DL4_04474 | BLDA23_10770 | CK945_RS12200 | ACH97_207305 |
| stage V sporulation protein K | TRNA_RS31350 | BL14DL4_04475 | BLDA23_10775 | CK945_RS12195 | ACH97_207310 |
| GTPase HflX | TRNA_RS31355 | BL14DL4_00210 | BLDA23_10780 | CK945_RS12190 | ACH97_207315 |
| hypothetical protein | TRNA_RS31360 | BL14DL4_00211 | BLDA23_10785 | CK945_RS12185 | ACH97_207320 |
| MerR family transcriptional regulator | TRNA_RS31365 | BL14DL4_00212 | BLDA23_10790 | CK945_RS12180 | ACH97_207325 |
| type I glutamate--ammonia ligase | TRNA_RS31370 | BL14DL4_00213 | BLDA23_10795 | CK945_RS12175 | ACH97_207330 |
| hypothetical protein | TRNA_RS31375 | BL14DL4_00214 | BLDA23_10800 | CK945_RS11890 | ACH97_207355 |
| hypothetical protein | TRNA_RS31380 | BL14DL4_00216 | BLDA23_10805 | CK945_RS11880 | ACH97_207360 |
| hypothetical protein | TRNA_RS31400 | BL14DL4_00220 | BLDA23_10825 | CK945_RS11845 | ACH97_207385 |
| abhydrolase domain-containing 18 | TRNA_RS31410 | BL14DL4_00222 | BLDA23_10835 | CK945_RS11840 | ACH97_207390 |
| methyltransferase domain-containing protein | TRNA_RS31425 | BL14DL4_00225 | BLDA23_10850 | CK945_RS11825 | ACH97_207405 |
| DUF4944 domain-containing protein | TRNA_RS31430 | BL14DL4_00226 | BLDA23_10855 | CK945_RS11820 | ACH97_207410 |
| GNAT family N-acetyltransferase | TRNA_RS31435 | BL14DL4_00227 | BLDA23_10860 | CK945_RS11815 | ACH97_207415 |
| hypothetical protein | TRNA_RS31440 | BL14DL4_00228 | BLDA23_10865 | CK945_RS11810 | ACH97_207420 |
| TetR/AcrR family transcriptional regulator | TRNA_RS31450 | BL14DL4_00231 | BLDA23_10875 | CK945_RS11800 | ACH97_207430 |
| QacE family quaternary ammonium compound efflux SMR transporter | TRNA_RS31455 | BL14DL4_00232 | BLDA23_10880 | CK945_RS11795 | ACH97_207435 |
| QacE family quaternary ammonium compound efflux SMR transporter | TRNA_RS31460 | BL14DL4_00233 | BLDA23_10885 | CK945_RS11790 | ACH97_207440 |
| YxeA family protein | TRNA_RS31470 | BL14DL4_00235 | BLDA23_10895 | CK945_RS11780 | ACH97_207450 |
| hypothetical protein | TRNA_RS31475 | BL14DL4_00236 | BLDA23_10900 | CK945_RS11775 | ACH97_207455 |
| manganese catalase | TRNA_RS31485 | BL14DL4_00238 | BLDA23_10910 | CK945_RS11765 | ACH97_207465 |
| DUF2935 domain-containing protein | TRNA_RS31490 | BL14DL4_00239 | BLDA23_10915 | CK945_RS11760 | ACH97_207470 |
| N-acetyltransferase | TRNA_RS31495 | BL14DL4_00240 | BLDA23_10920 | CK945_RS11755 | ACH97_207475 |
| hypothetical protein | TRNA_RS31500 | BL14DL4_00241 | BLDA23_10925 | CK945_RS11750 | ACH97_207480 |
| TetR/AcrR family transcriptional regulator | TRNA_RS31505 | BL14DL4_00242 | BLDA23_10930 | CK945_RS11745 | ACH97_207485 |
| MarR family transcriptional regulator | TRNA_RS31510 | BL14DL4_00243 | BLDA23_10935 | CK945_RS11740 | ACH97_207490 |
| ring-cleaving dioxygenase | TRNA_RS31520 | BL14DL4_00245 | BLDA23_10945 | CK945_RS11735 | ACH97_207495 |
| flavin reductase family protein | TRNA_RS31525 | BL14DL4_00246 | BLDA23_10950 | CK945_RS11730 | ACH97_207500 |
| NAD(P)H:quinone oxidoreductase | TRNA_RS31530 | BL14DL4_00247 | BLDA23_10955 | CK945_RS11725 | ACH97_207505 |
| prohibitin family protein | TRNA_RS31535 | BL14DL4_00248 | BLDA23_10960 | CK945_RS11720 | ACH97_207510 |
| hypothetical protein | TRNA_RS31540 | BL14DL4_00249 | BLDA23_10965 | CK945_RS11715 | ACH97_207515 |
| thymidylate synthase | TRNA_RS31545 | BL14DL4_00250 | BLDA23_10970 | CK945_RS11710 | ACH97_207520 |
| sporulation protein | TRNA_RS31550 | BL14DL4_00251 | BLDA23_10975 | CK945_RS11700 | ACH97_207525 |
| LexA repressor | TRNA_RS31555 | BL14DL4_00252 | BLDA23_10980 | CK945_RS11690 | ACH97_207530 |
| cell division suppressor protein YneA | TRNA_RS31560 | BL14DL4_00253 | BLDA23_10985 | CK945_RS11685 | ACH97_207535 |
| serine recombinase | TRNA_RS31565 | BL14DL4_00254 | BLDA23_10990 | CK945_RS11680 | ACH97_207540 |
| DUF896 domain-containing protein | TRNA_RS31570 | BL14DL4_00255 | BLDA23_10995 | CK945_RS11675 | ACH97_207545 |
| transketolase | TRNA_RS31575 | BL14DL4_00256 | BLDA23_11000 | CK945_RS11670 | ACH97_207550 |
| sporulation inhibitor of replication protein SirA | TRNA_RS31580 | BL14DL4_00257 | BLDA23_11005 | CK945_RS11665 | ACH97_207555 |
| YneF family protein | TRNA_RS31585 | BL14DL4_00258 | BLDA23_11010 | CK945_RS11660 | ACH97_207560 |
| aspartyl-phosphate phosphatase Spo0E family protein | TRNA_RS31590 | BL14DL4_00259 | BLDA23_11015 | CK945_RS11655 | ACH97_207565 |
| cytochrome c biogenesis protein CcdA | TRNA_RS31595 | BL14DL4_00260 | BLDA23_11020 | CK945_RS11650 | ACH97_207570 |
| response regulator | TRNA_RS31600 | BL14DL4_00261 | BLDA23_11025 | CK945_RS11645 | ACH97_207575 |
| DUF1453 family protein | TRNA_RS31605 | BL14DL4_00262 | BLDA23_11030 | CK945_RS11640 | ACH97_207580 |
| DUF2621 domain-containing protein | TRNA_RS31610 | BL14DL4_00263 | BLDA23_11035 | CK945_RS11635 | ACH97_207585 |
| Hsp20/alpha crystallin family protein | TRNA_RS31615 | BL14DL4_00264 | BLDA23_11040 | CK945_RS11630 | ACH97_207590 |
| aconitate hydratase AcnA | TRNA_RS31630 | BL14DL4_00265 | BLDA23_11055 | CK945_RS11615 | ACH97_207605 |
| thioredoxin | TRNA_RS31635 | BL14DL4_00266 | BLDA23_11060 | CK945_RS11610 | ACH97_207610 |
| small, acid-soluble spore protein N | TRNA_RS31640 | BL14DL4_00268 | BLDA23_11070 | CK945_RS11600 | ACH97_207615 |
| small acid-soluble spore protein Tlp | TRNA_RS31645 | BL14DL4_00269 | BLDA23_11075 | CK945_RS11595 | ACH97_207620 |
| YbgC/FadM family acyl-CoA thioesterase | TRNA_RS31650 | BL14DL4_00270 | BLDA23_11080 | CK945_RS11590 | ACH97_207625 |
| hypothetical protein | TRNA_RS31655 | BL14DL4_00271 | BLDA23_11085 | CK945_RS11585 | ACH97_207630 |
| YxeA family protein | TRNA_RS31665 | BL14DL4_00272 | BLDA23_11095 | CK945_RS11580 | ACH97_207635 |
| membrane protein | TRNA_RS31670 | BL14DL4_00273 | BLDA23_11100 | CK945_RS11575 | ACH97_207640 |
| hypothetical protein | TRNA_RS31675 | BL14DL4_00274 | BLDA23_11105 | CK945_RS11570 | ACH97_207645 |
| glycerol-3-phosphate 1-O-acyltransferase PlsY | TRNA_RS31680 | BL14DL4_00275 | BLDA23_11110 | CK945_RS11565 | ACH97_207650 |
| CoA-binding protein | TRNA_RS31685 | BL14DL4_00276 | BLDA23_11115 | CK945_RS11560 | ACH97_207655 |
| DNA topoisomerase IV subunit B | TRNA_RS31690 | BL14DL4_00277 | BLDA23_11120 | CK945_RS11555 | ACH97_207660 |
| DNA topoisomerase IV subunit A | TRNA_RS31695 | BL14DL4_00278 | BLDA23_11125 | CK945_RS11550 | ACH97_207665 |
| GntR family transcriptional regulator | TRNA_RS31700 | BL14DL4_00279 | BLDA23_11130 | CK945_RS11545 | ACH97_207670 |
| ATPase | TRNA_RS31705 | BL14DL4_00280 | BLDA23_11135 | CK945_RS11540 | ACH97_207675 |
| L-ribulose-5-phosphate 4-epimerase | TRNA_RS31710 | BL14DL4_00281 | BLDA23_11140 | CK945_RS11535 | ACH97_207680 |
| L-arabinose isomerase 2 | TRNA_RS31715 | BL14DL4_00282 | BLDA23_11145 | CK945_RS11530 | ACH97_207685 |
| sugar porter family MFS transporter | TRNA_RS31720 | BL14DL4_00283 | BLDA23_11150 | CK945_RS11525 | ACH97_207690 |
| (S)-acetoin forming diacetyl reductase | TRNA_RS31725 | BL14DL4_00284 | BLDA23_11155 | CK945_RS11520 | ACH97_207695 |
| hypothetical protein | TRNA_RS31730 | BL14DL4_00286 | BLDA23_11160 | CK945_RS11515 | ACH97_207700 |
| alanine:cation symporter family protein | TRNA_RS31735 | BL14DL4_00287 | BLDA23_11165 | CK945_RS11510 | ACH97_207705 |
| hypothetical protein | TRNA_RS31740 | BL14DL4_00288 | BLDA23_11170 | CK945_RS11505 | ACH97_207710 |
| respiratory nitrate reductase subunit gamma | TRNA_RS31745 | BL14DL4_00289 | BLDA23_11175 | CK945_RS11500 | ACH97_207715 |
| nitrate reductase molybdenum cofactor assembly chaperone | TRNA_RS31750 | BL14DL4_00290 | BLDA23_11180 | CK945_RS11495 | ACH97_207720 |
| nitrate reductase subunit beta | TRNA_RS31755 | BL14DL4_00291 | BLDA23_11185 | CK945_RS11490 | ACH97_207725 |
| nitrate reductase subunit alpha | TRNA_RS31760 | BL14DL4_00292 | BLDA23_11190 | CK945_RS11485 | ACH97_207730 |
| DUF438 domain-containing protein | TRNA_RS31765 | BL14DL4_00293 | BLDA23_11195 | CK945_RS11480 | ACH97_207735 |
| DUF1858 domain-containing protein | TRNA_RS31770 | BL14DL4_00294 | BLDA23_11200 | CK945_RS11475 | ACH97_207740 |
| radical SAM/SPASM domain-containing protein | TRNA_RS31775 | BL14DL4_00295 | BLDA23_11205 | CK945_RS11470 | ACH97_207745 |
| hypothetical protein | TRNA_RS31780 | BL14DL4_00296 | BLDA23_11210 | CK945_RS11465 | ACH97_207750 |
| membrane protein | TRNA_RS31785 | BL14DL4_00297 | BLDA23_11215 | CK945_RS11460 | ACH97_207755 |
| Crp/Fnr family transcriptional regulator | TRNA_RS31790 | BL14DL4_00298 | BLDA23_11220 | CK945_RS11455 | ACH97_207760 |
| NarK/NasA family nitrate transporter | TRNA_RS31795 | BL14DL4_00299 | BLDA23_11225 | CK945_RS11450 | ACH97_207765 |
| DUF2164 domain-containing protein | TRNA_RS31800 | BL14DL4_00300 | BLDA23_11230 | CK945_RS11445 | ACH97_207770 |
| Crp/Fnr family transcriptional regulator | TRNA_RS31805 | BL14DL4_00301 | BLDA23_11235 | CK945_RS11440 | ACH97_207775 |
| iron-sulfur cluster repair di-iron protein | TRNA_RS31815 | BL14DL4_00303 | BLDA23_11245 | CK945_RS11430 | ACH97_207785 |
| DUF1360 domain-containing protein | TRNA_RS31820 | BL14DL4_00304 | BLDA23_11250 | CK945_RS11425 | ACH97_207790 |
| endoglucanase | TRNA_RS31825 | BL14DL4_00305 | BLDA23_11255 | CK945_RS11420 | ACH97_207795 |
| hypothetical protein | TRNA_RS31830 | BL14DL4_00306 | BLDA23_11260 | CK945_RS11415 | ACH97_207800 |
| DUF1565 domain-containing protein | TRNA_RS31860 | BL14DL4_00313 | BLDA23_11290 | CK945_RS11380 | ACH97_207825 |
| branched-chain amino acid transport system II carrier protein | TRNA_RS31865 | BL14DL4_00314 | BLDA23_11295 | CK945_RS11375 | ACH97_207830 |
| membrane protein | TRNA_RS31885 | BL14DL4_00318 | BLDA23_11315 | CK945_RS11365 | ACH97_207860 |
| enoyl-CoA hydratase/isomerase family protein | TRNA_RS31895 | BL14DL4_00320 | BLDA23_11325 | CK945_RS11355 | ACH97_207875 |
| enoyl-CoA hydratase | TRNA_RS31900 | BL14DL4_00321 | BLDA23_11330 | CK945_RS11350 | ACH97_207880 |
| methylmalonate-semialdehyde dehydrogenase (CoA acylating) | TRNA_RS31905 | BL14DL4_00322 | BLDA23_11335 | CK945_RS11345 | ACH97_207885 |
| NAD(P)-dependent oxidoreductase | TRNA_RS31910 | BL14DL4_00323 | BLDA23_11340 | CK945_RS11340 | ACH97_207890 |
| acyl-CoA dehydrogenase | TRNA_RS31915 | BL14DL4_00324 | BLDA23_11345 | CK945_RS11335 | ACH97_207895 |
| mannose-6-phosphate isomerase, class I | TRNA_RS31920 | BL14DL4_00325 | BLDA23_11350 | CK945_RS11330 | ACH97_207900 |
| PTS mannose transporter subunit IIABC | TRNA_RS31925 | BL14DL4_00326 | BLDA23_11355 | CK945_RS11325 | ACH97_207905 |
| PRD domain-containing protein | TRNA_RS31945 | BL14DL4_00330 | BLDA23_11380 | CK945_RS11295 | ACH97_207915 |
| ABC-2 transporter permease | TRNA_RS31950 | BL14DL4_00331 | BLDA23_11385 | CK945_RS11290 | ACH97_207920 |
| ABC transporter ATP-binding protein | TRNA_RS31955 | BL14DL4_00332 | BLDA23_11390 | CK945_RS11285 | ACH97_207925 |
| GntR family transcriptional regulator | TRNA_RS31960 | BL14DL4_00333 | BLDA23_11395 | CK945_RS11280 | ACH97_207930 |
| SDR family oxidoreductase | TRNA_RS31965 | BL14DL4_00334 | BLDA23_11400 | CK945_RS11275 | ACH97_207935 |
| Mannonate dehydratase 2 | TRNA_RS31970 | BL14DL4_00335 | BLDA23_11405 | CK945_RS11270 | ACH97_207940 |
| glycoside hydrolase family 31 protein | TRNA_RS31975 | BL14DL4_00336 | BLDA23_11410 | CK945_RS11265 | ACH97_207945 |
| TRAP transporter large permease | TRNA_RS31980 | BL14DL4_00337 | BLDA23_11415 | CK945_RS11260 | ACH97_207950 |
| TRAP transporter small permease | TRNA_RS31985 | BL14DL4_00338 | BLDA23_11420 | CK945_RS11255 | ACH97_207955 |
| TRAP transporter substrate-binding protein | TRNA_RS31990 | BL14DL4_00339 | BLDA23_11425 | CK945_RS11250 | ACH97_207960 |
| LacI family DNA-binding transcriptional regulator | TRNA_RS31995 | BL14DL4_00340 | BLDA23_11430 | CK945_RS11245 | ACH97_207965 |
| protein ArsC | TRNA_RS32000 | BL14DL4_00341 | BLDA23_11435 | CK945_RS11240 | ACH97_207970 |
| arsenical efflux pump membrane protein ArsB | TRNA_RS32005 | BL14DL4_00342 | BLDA23_11440 | CK945_RS11235 | ACH97_207975 |
| ArsR family transcriptional regulator | TRNA_RS32010 | BL14DL4_00343 | BLDA23_11445 | CK945_RS11230 | ACH97_207980 |
| hypothetical protein | TRNA_RS32020 | BL14DL4_00344 | BLDA23_11455 | CK945_RS11225 | ACH97_207985 |
| hypothetical protein | TRNA_RS32025 | BL14DL4_00345 | BLDA23_11460 | CK945_RS11220 | ACH97_207990 |
| glucose transporter GlcU | TRNA_RS32030 | BL14DL4_00346 | BLDA23_11465 | CK945_RS11215 | ACH97_207995 |
| hypothetical protein | TRNA_RS32035 | BL14DL4_00347 | BLDA23_11470 | CK945_RS11210 | ACH97_208000 |
| pyruvate formate lyase-activating protein | TRNA_RS32045 | BL14DL4_00349 | BLDA23_11480 | CK945_RS11200 | ACH97_208010 |
| formate C-acetyltransferase | TRNA_RS32050 | BL14DL4_00350 | BLDA23_11485 | CK945_RS11195 | ACH97_208015 |
| D-alanyl-D-alanine carboxypeptidase/D-alanyl-D-alanine-endopeptidase | TRNA_RS32055 | BL14DL4_00351 | BLDA23_11490 | CK945_RS11185 | ACH97_208025 |
| NAD(P)/FAD-dependent oxidoreductase | TRNA_RS32060 | BL14DL4_00352 | BLDA23_11580 | CK945_RS11160 | ACH97_208050 |
| DUF1641 domain-containing protein | TRNA_RS32065 | BL14DL4_00353 | BLDA23_11585 | CK945_RS11155 | ACH97_208055 |
| helix-turn-helix domain-containing protein | TRNA_RS32075 | BL14DL4_00355 | BLDA23_11595 | CK945_RS11145 | ACH97_208065 |
| hypothetical protein | TRNA_RS32080 | BL14DL4_00356 | BLDA23_11600 | CK945_RS11140 | ACH97_208070 |
| AMP-dependent synthetase | TRNA_RS32085 | BL14DL4_00357 | BLDA23_11605 | CK945_RS11135 | ACH97_208075 |
| acyl-CoA carboxylase subunit beta | TRNA_RS32090 | BL14DL4_00358 | BLDA23_11610 | CK945_RS11130 | ACH97_208080 |
| enoyl-CoA hydratase | TRNA_RS32095 | BL14DL4_00359 | BLDA23_11615 | CK945_RS11125 | ACH97_208085 |
| hydroxymethylglutaryl-CoA lyase | TRNA_RS32100 | BL14DL4_00360 | BLDA23_11620 | CK945_RS11120 | ACH97_208090 |
| acetyl-CoA carboxylase biotin carboxyl carrier protein subunit | TRNA_RS32105 | BL14DL4_00361 | BLDA23_11625 | CK945_RS11115 | ACH97_208095 |
| acetyl-CoA carboxylase biotin carboxylase subunit | TRNA_RS32110 | BL14DL4_00362 | BLDA23_11630 | CK945_RS11110 | ACH97_208100 |
| AMP-binding protein | TRNA_RS32115 | BL14DL4_00363 | BLDA23_11635 | CK945_RS11080 | ACH97_210770 |
| acyl-CoA dehydrogenase | TRNA_RS32120 | BL14DL4_00364 | BLDA23_11640 | CK945_RS11075 | ACH97_210775 |
| flavodoxin family protein | TRNA_RS32125 | BL14DL4_00365 | BLDA23_11645 | CK945_RS11070 | ACH97_210780 |
| transcriptional regulator | TRNA_RS32130 | BL14DL4_00366 | BLDA23_11650 | CK945_RS11065 | ACH97_210785 |
| gamma-glutamyl-phosphate reductase | TRNA_RS32135 | BL14DL4_00367 | BLDA23_11655 | CK945_RS11060 | ACH97_210790 |
| glutamate 5-kinase | TRNA_RS32140 | BL14DL4_00368 | BLDA23_11660 | CK945_RS11055 | ACH97_210795 |
| pyrroline-5-carboxylate reductase | TRNA_RS32145 | BL14DL4_00369 | BLDA23_11665 | CK945_RS11050 | ACH97_210800 |
| DUF421 domain-containing protein | TRNA_RS32150 | BL14DL4_00371 | BLDA23_11670 | CK945_RS11045 | ACH97_210805 |
| inorganic phosphate transporter | TRNA_RS32160 | BL14DL4_00372 | BLDA23_11675 | CK945_RS11040 | ACH97_210810 |
| phosphoadenylyl-sulfate reductase | TRNA_RS32165 | BL14DL4_00373 | BLDA23_11680 | CK945_RS11035 | ACH97_210815 |
| phosphosulfolactate synthase | TRNA_RS32170 | BL14DL4_00374 | BLDA23_11685 | CK945_RS11030 | ACH97_210820 |
| MFS transporter | TRNA_RS32185 | BL14DL4_00377 | BLDA23_11700 | CK945_RS11015 | ACH97_210835 |
| glycosyltransferase | TRNA_RS32190 | BL14DL4_00378 | BLDA23_11705 | CK945_RS11010 | ACH97_210840 |
| hypothetical protein | TRNA_RS32195 | BL14DL4_00379 | BLDA23_11710 | CK945_RS11005 | ACH97_210845 |
| glutamate synthase subunit beta | TRNA_RS32205 | BL14DL4_00381 | BLDA23_11720 | CK945_RS10995 | ACH97_210850 |
| glutamate synthase large subunit | TRNA_RS32210 | BL14DL4_00382 | BLDA23_11725 | CK945_RS10990 | ACH97_210855 |
| LysR family transcriptional regulator | TRNA_RS32215 | BL14DL4_00383 | BLDA23_11730 | CK945_RS10985 | ACH97_210860 |
| hypothetical protein | TRNA_RS32225 | BL14DL4_00385 | BLDA23_11740 | CK945_RS10980 | ACH97_210865 |
| ABC transporter ATP-binding protein | TRNA_RS32240 | BL14DL4_00388 | BLDA23_11755 | CK945_RS10965 | ACH97_210880 |
| ABC transporter ATP-binding protein | TRNA_RS32245 | BL14DL4_00389 | BLDA23_11760 | CK945_RS10960 | ACH97_210885 |
| cytochrome P450 | TRNA_RS32250 | BL14DL4_00390 | BLDA23_11765 | CK945_RS10955 | ACH97_210890 |
| 3-ketoacyl-ACP reductase | TRNA_RS32255 | BL14DL4_00391 | BLDA23_11770 | CK945_RS10950 | ACH97_210895 |
| DHA2 family efflux MFS transporter permease subunit | TRNA_RS32270 | BL14DL4_00393 | BLDA23_11785 | CK945_RS10940 | ACH97_210905 |
| molybdopterin oxidoreductase family protein | TRNA_RS32275 | BL14DL4_00394 | BLDA23_11790 | CK945_RS10935 | ACH97_210910 |
| MarR family transcriptional regulator | TRNA_RS32280 | BL14DL4_00395 | BLDA23_11795 | CK945_RS10930 | ACH97_210915 |
| germination protein | TRNA_RS32290 | BL14DL4_00397 | BLDA23_11805 | CK945_RS10920 | ACH97_210925 |
| Ger(x)C family spore germination protein | TRNA_RS32295 | BL14DL4_00398 | BLDA23_11810 | CK945_RS10915 | ACH97_210930 |
| germination protein | TRNA_RS32300 | BL14DL4_00399 | BLDA23_11815 | CK945_RS10910 | ACH97_210935 |
| hypothetical protein | TRNA_RS32305 | BL14DL4_00400 | BLDA23_11820 | CK945_RS10905 | ACH97_210940 |
| spore germination protein | TRNA_RS32310 | BL14DL4_00401 | BLDA23_11825 | CK945_RS10900 | ACH97_210945 |
| HAMP domain-containing protein | TRNA_RS32320 | BL14DL4_00403 | BLDA23_11835 | CK945_RS10885 | ACH97_210955 |
| tRNA-binding protein | TRNA_RS32325 | BL14DL4_00404 | BLDA23_11840 | CK945_RS10880 | ACH97_210960 |
| serine protease | TRNA_RS32330 | BL14DL4_00405 | BLDA23_11845 | CK945_RS10875 | ACH97_210965 |
| hypothetical protein | TRNA_RS32335 | BL14DL4_00406 | BLDA23_11850 | CK945_RS10870 | ACH97_210970 |
| TetR/AcrR family transcriptional regulator | TRNA_RS32340 | BL14DL4_00407 | BLDA23_11855 | CK945_RS10865 | ACH97_210975 |
| MarR family transcriptional regulator | TRNA_RS32345 | BL14DL4_00408 | BLDA23_11860 | CK945_RS10860 | ACH97_210980 |
| DinB family protein | TRNA_RS32355 | BL14DL4_00410 | BLDA23_11870 | CK945_RS10850 | ACH97_210990 |
| membrane protein | TRNA_RS32360 | BL14DL4_00411 | BLDA23_11875 | CK945_RS10845 | ACH97_210995 |
| DUF4166 domain-containing protein | TRNA_RS32365 | BL14DL4_00412 | BLDA23_11880 | CK945_RS10840 | ACH97_211000 |
| membrane protein | TRNA_RS32370 | BL14DL4_00413 | BLDA23_11885 | CK945_RS10835 | ACH97_211005 |
| TetR/AcrR family transcriptional regulator | TRNA_RS32375 | BL14DL4_00414 | BLDA23_11890 | CK945_RS10830 | ACH97_211010 |
| MBL fold metallo-hydrolase | TRNA_RS32380 | BL14DL4_00415 | BLDA23_11895 | CK945_RS10825 | ACH97_211015 |
| AraC family transcriptional regulator | TRNA_RS32385 | BL14DL4_00416 | BLDA23_11900 | CK945_RS10820 | ACH97_211020 |
| transcriptional regulator | TRNA_RS32390 | BL14DL4_00417 | BLDA23_11905 | CK945_RS10815 | ACH97_211025 |
| YafY family transcriptional regulator | TRNA_RS32395 | BL14DL4_00418 | BLDA23_11910 | CK945_RS10810 | ACH97_211030 |
| hypothetical protein | TRNA_RS32400 | BL14DL4_00419 | BLDA23_11915 | CK945_RS10805 | ACH97_211035 |
| ArsR family transcriptional regulator | TRNA_RS32405 | BL14DL4_00420 | BLDA23_11920 | CK945_RS10800 | ACH97_211040 |
| hypothetical protein | TRNA_RS32415 | BL14DL4_00422 | BLDA23_11930 | CK945_RS10790 | ACH97_211050 |
| APC family permease | TRNA_RS32420 | BL14DL4_00423 | BLDA23_11935 | CK945_RS10785 | ACH97_211055 |
| lysozyme family protein | TRNA_RS32425 | BL14DL4_00424 | BLDA23_11940 | CK945_RS10780 | ACH97_211060 |
| peptidase | TRNA_RS32435 | BL14DL4_00426 | BLDA23_11950 | CK945_RS12395 | ACH97_211215 |
| DUF420 domain-containing protein | TRNA_RS32450 | BL14DL4_00429 | BLDA23_11965 | CK945_RS10755 | ACH97_211085 |
| hypothetical protein | TRNA_RS32455 | BL14DL4_00430 | BLDA23_11970 | CK945_RS10750 | ACH97_211090 |
| GNAT family N-acetyltransferase | TRNA_RS32460 | BL14DL4_00431 | BLDA23_11975 | CK945_RS10745 | ACH97_211095 |
| solute:sodium symporter family transporter | TRNA_RS32465 | BL14DL4_00432 | BLDA23_11980 | CK945_RS10735 | ACH97_211105 |
| LysM peptidoglycan-binding domain-containing protein | TRNA_RS32470 | BL14DL4_00433 | BLDA23_11985 | CK945_RS10730 | ACH97_211110 |
| DNA helicase RecQ | TRNA_RS32475 | BL14DL4_00434 | BLDA23_11990 | CK945_RS10725 | ACH97_211115 |
| hypothetical protein | TRNA_RS32480 | BL14DL4_00435 | BLDA23_11995 | CK945_RS10720 | ACH97_211120 |
| MmcQ/YjbR family DNA-binding protein | TRNA_RS32490 | BL14DL4_00439 | BLDA23_12010 | CK945_RS10705 | ACH97_211130 |
| DUF1641 domain-containing protein | TRNA_RS32505 | BL14DL4_00441 | BLDA23_12030 | CK945_RS10695 | ACH97_211135 |
| formate dehydrogenase subunit alpha | TRNA_RS32510 | BL14DL4_00442 | BLDA23_12035 | CK945_RS10690 | ACH97_211140 |
| pilus assembly protein PilZ | TRNA_RS32530 | BL14DL4_00445 | BLDA23_12065 | CK945_RS12335 | ACH97_211165 |
| DUF5082 domain-containing protein | TRNA_RS32550 | BL14DL4_00449 | BLDA23_12080 | CK945_RS12350 | ACH97_211170 |
| hypothetical protein | TRNA_RS32555 | BL14DL4_00450 | BLDA23_12085 | CK945_RS12355 | ACH97_211175 |
| DUF4025 domain-containing protein | TRNA_RS32560 | BL14DL4_00451 | BLDA23_12090 | CK945_RS12365 | ACH97_211185 |
| SMI1/KNR4 family protein | TRNA_RS32575 | BL14DL4_00454 | BLDA23_12105 | CK945_RS12390 | ACH97_211210 |
| hypothetical protein | TRNA_RS32580 | BL14DL4_00455 | BLDA23_12110 | CK945_RS12400 | ACH97_211220 |
| spore coat protein | TRNA_RS32585 | BL14DL4_00456 | BLDA23_12115 | CK945_RS12405 | ACH97_211225 |
| hypothetical protein | TRNA_RS32590 | BL14DL4_00457 | BLDA23_12120 | CK945_RS12410 | ACH97_211230 |
| alpha/beta hydrolase | TRNA_RS32595 | BL14DL4_00458 | BLDA23_12125 | CK945_RS12415 | ACH97_211235 |
| hypothetical protein | TRNA_RS32600 | BL14DL4_00459 | BLDA23_12130 | CK945_RS12420 | ACH97_211240 |
| LysE family translocator | TRNA_RS32605 | BL14DL4_00461 | BLDA23_12140 | CK945_RS12430 | ACH97_211245 |
| 30S ribosomal protein S14 type Z 2 | TRNA_RS32610 | BL14DL4_00462 | BLDA23_12145 | CK945_RS12440 | ACH97_208120 |
| NUDIX domain-containing protein | TRNA_RS32615 | BL14DL4_00463 | BLDA23_12150 | CK945_RS12445 | ACH97_208125 |
| phosphoenolpyruvate synthase | TRNA_RS32620 | BL14DL4_00464 | BLDA23_12155 | CK945_RS12450 | ACH97_208130 |
| TetR/AcrR family transcriptional regulator | TRNA_RS32625 | BL14DL4_00465 | BLDA23_12160 | CK945_RS12455 | ACH97_208135 |
| hypothetical protein | TRNA_RS32630 | BL14DL4_00466 | BLDA23_12165 | CK945_RS12460 | ACH97_208140 |
| DUF2512 family protein | TRNA_RS32635 | BL14DL4_00467 | BLDA23_12170 | CK945_RS12465 | ACH97_208145 |
| DUF664 domain-containing protein | TRNA_RS32640 | BL14DL4_00468 | BLDA23_12175 | CK945_RS12470 | ACH97_208150 |
| FMN-dependent NADH-azoreductase 1 | TRNA_RS32645 | BL14DL4_00469 | BLDA23_12180 | CK945_RS12475 | ACH97_208155 |
| general stress protein | TRNA_RS32650 | BL14DL4_00470 | BLDA23_12185 | CK945_RS12500 | ACH97_208175 |
| hypothetical protein | TRNA_RS32655 | BL14DL4_00471 | BLDA23_12190 | CK945_RS12505 | ACH97_208180 |
| hypothetical protein | TRNA_RS32660 | BL14DL4_00472 | BLDA23_12195 | CK945_RS12510 | ACH97_208185 |
| aldehyde dehydrogenase family protein | TRNA_RS32665 | BL14DL4_00473 | BLDA23_12200 | CK945_RS12515 | ACH97_208190 |
| N-acetyltransferase | TRNA_RS32675 | BL14DL4_00475 | BLDA23_12210 | CK945_RS12525 | ACH97_208200 |
| cytidine deaminase | TRNA_RS32680 | BL14DL4_00476 | BLDA23_12215 | CK945_RS12530 | ACH97_208205 |
| squalene--hopene cyclase | TRNA_RS32685 | BL14DL4_00477 | BLDA23_12220 | CK945_RS12535 | ACH97_208210 |
| superoxide dismutase | TRNA_RS32690 | BL14DL4_00478 | BLDA23_12225 | CK945_RS12540 | ACH97_208215 |
| hypothetical protein | TRNA_RS32695 | BL14DL4_00479 | BLDA23_12230 | CK945_RS12545 | ACH97_208220 |
| sodium-dependent transporter | TRNA_RS32700 | BL14DL4_00480 | BLDA23_12235 | CK945_RS12550 | ACH97_208225 |
| bile acid:sodium symporter family protein | TRNA_RS32705 | BL14DL4_00481 | BLDA23_12240 | CK945_RS12555 | ACH97_208230 |
| 2-oxoglutarate dehydrogenase complex dihydrolipoyllysine-residue succinyltransferase | TRNA_RS32710 | BL14DL4_00482 | BLDA23_12245 | CK945_RS12560 | ACH97_208235 |
| 2-oxoglutarate dehydrogenase E1 component | TRNA_RS32715 | BL14DL4_00483 | BLDA23_12250 | CK945_RS12565 | ACH97_208240 |
| VWA domain-containing protein | TRNA_RS32720 | BL14DL4_00484 | BLDA23_12255 | CK945_RS12570 | ACH97_208245 |
| MoxR family ATPase | TRNA_RS32725 | BL14DL4_00485 | BLDA23_12260 | CK945_RS12575 | ACH97_208250 |
| superoxide dismutase | TRNA_RS32730 | BL14DL4_00486 | BLDA23_12265 | CK945_RS12580 | ACH97_208255 |
| peptidoglycan endopeptidase | TRNA_RS32735 | BL14DL4_00487 | BLDA23_12270 | CK945_RS12585 | ACH97_208260 |
| MATE family efflux transporter | TRNA_RS32745 | BL14DL4_00488 | BLDA23_12280 | CK945_RS12595 | ACH97_208265 |
| bacillithiol biosynthesis deacetylase BshB2 | TRNA_RS32750 | BL14DL4_00489 | BLDA23_12285 | CK945_RS12600 | ACH97_208270 |
| DUF1806 family protein | TRNA_RS32755 | BL14DL4_00490 | BLDA23_12290 | CK945_RS12605 | ACH97_208275 |
| hypothetical protein | TRNA_RS32760 | BL14DL4_00491 | BLDA23_12295 | CK945_RS12610 | ACH97_208280 |
| EamA family transporter RarD | TRNA_RS32765 | BL14DL4_00492 | BLDA23_12300 | CK945_RS12615 | ACH97_208285 |
| germination protein GerT | TRNA_RS32770 | BL14DL4_00493 | BLDA23_12305 | CK945_RS12620 | ACH97_208290 |
| hypothetical protein | TRNA_RS32775 | BL14DL4_00495 | BLDA23_12310 | CK945_RS12625 | ACH97_208295 |
| hypothetical protein | TRNA_RS32780 | BL14DL4_00496 | BLDA23_12315 | CK945_RS12630 | ACH97_208300 |
| HxlR family transcriptional regulator | TRNA_RS32790 | BL14DL4_00498 | BLDA23_12325 | CK945_RS12640 | ACH97_208310 |
| nitroreductase family protein | TRNA_RS32795 | BL14DL4_00499 | BLDA23_12330 | CK945_RS12645 | ACH97_208315 |
| alpha/beta hydrolase | TRNA_RS32800 | BL14DL4_00500 | BLDA23_12335 | CK945_RS12650 | ACH97_208320 |
| DUF3311 domain-containing protein | TRNA_RS32805 | BL14DL4_00501 | BLDA23_12340 | CK945_RS12655 | ACH97_208325 |
| symporter | TRNA_RS32810 | BL14DL4_00502 | BLDA23_12345 | CK945_RS12660 | ACH97_208330 |
| PDZ domain-containing protein | TRNA_RS32825 | BL14DL4_00503 | BLDA23_12355 | CK945_RS12665 | ACH97_208335 |
| SAM-dependent methyltransferase | TRNA_RS32830 | BL14DL4_00504 | BLDA23_12360 | CK945_RS12670 | ACH97_208340 |
| hypothetical protein | TRNA_RS32835 | BL14DL4_00505 | BLDA23_12365 | CK945_RS12675 | ACH97_208345 |
| D-alanyl-D-alanine carboxypeptidase family protein | TRNA_RS32840 | BL14DL4_00506 | BLDA23_12370 | CK945_RS12680 | ACH97_208350 |
| purine-nucleoside phosphorylase | TRNA_RS32845 | BL14DL4_00507 | BLDA23_12375 | CK945_RS12685 | ACH97_208355 |
| permease | TRNA_RS32850 | BL14DL4_00508 | BLDA23_12380 | CK945_RS12690 | ACH97_208360 |
| TIGR03943 family protein | TRNA_RS32855 | BL14DL4_00509 | BLDA23_12385 | CK945_RS12695 | ACH97_208365 |
| hypothetical protein | TRNA_RS32860 | BL14DL4_00511 | BLDA23_12395 | CK945_RS12705 | ACH97_208370 |
| PAP2 family protein | TRNA_RS32865 | BL14DL4_00512 | BLDA23_12400 | CK945_RS12710 | ACH97_208375 |
| hypothetical protein | TRNA_RS32875 | BL14DL4_00515 | BLDA23_12410 | CK945_RS12725 | ACH97_208380 |
| YozE family protein | TRNA_RS32880 | BL14DL4_00516 | BLDA23_12415 | CK945_RS12730 | ACH97_208385 |
| YokU family protein | TRNA_RS32885 | BL14DL4_00517 | BLDA23_12420 | CK945_RS12735 | ACH97_208390 |
| putative beta-lysine N-acetyltransferase | TRNA_RS32895 | BL14DL4_00519 | BLDA23_12430 | CK945_RS12745 | ACH97_208565 |
| peptidase | TRNA_RS32900 | BL14DL4_00520 | BLDA23_12435 | CK945_RS12750 | ACH97_208570 |
| 3-oxoacid CoA-transferase subunit B | TRNA_RS32905 | BL14DL4_00521 | BLDA23_12440 | CK945_RS12755 | ACH97_208575 |
| CoA transferase subunit A | TRNA_RS32910 | BL14DL4_00522 | BLDA23_12445 | CK945_RS12760 | ACH97_208580 |
| MFS transporter | TRNA_RS32920 | BL14DL4_00524 | BLDA23_12455 | CK945_RS12770 | ACH97_208590 |
| GNAT family N-acetyltransferase | TRNA_RS32925 | BL14DL4_00525 | BLDA23_12465 | CK945_RS12780 | ACH97_208595 |
| peptide-methionine (R)-S-oxide reductase | TRNA_RS32930 | BL14DL4_00526 | BLDA23_12470 | CK945_RS12785 | ACH97_208610 |
| peptide-methionine (S)-S-oxide reductase | TRNA_RS32935 | BL14DL4_00527 | BLDA23_12475 | CK945_RS12790 | ACH97_208615 |
| MarR family transcriptional regulator | TRNA_RS32940 | BL14DL4_00528 | BLDA23_12480 | CK945_RS12795 | ACH97_208620 |
| hypothetical protein | TRNA_RS32945 | BL14DL4_00529 | BLDA23_12485 | CK945_RS12800 | ACH97_208625 |
| GGDEF domain-containing protein | TRNA_RS32950 | BL14DL4_00530 | BLDA23_12490 | CK945_RS12805 | ACH97_208630 |
| DUF4397 domain-containing protein | TRNA_RS32960 | BL14DL4_00532 | BLDA23_12500 | CK945_RS12815 | ACH97_208640 |
| hypothetical protein | TRNA_RS32965 | BL14DL4_00533 | BLDA23_12505 | CK945_RS12820 | ACH97_208645 |
| DUF2140 family protein | TRNA_RS32970 | BL14DL4_00534 | BLDA23_12510 | CK945_RS12825 | ACH97_208650 |
| hypothetical protein | TRNA_RS32975 | BL14DL4_00535 | BLDA23_12515 | CK945_RS12830 | ACH97_208655 |
| photosynthetic protein synthase I | TRNA_RS32980 | BL14DL4_00536 | BLDA23_12520 | CK945_RS12835 | ACH97_208660 |
| DegV family protein | TRNA_RS32985 | BL14DL4_00537 | BLDA23_12525 | CK945_RS12840 | ACH97_208665 |
| DUF2535 family protein | TRNA_RS32990 | BL14DL4_00538 | BLDA23_12530 | CK945_RS12850 | ACH97_208670 |
| threonine dehydratase | TRNA_RS32995 | BL14DL4_00539 | BLDA23_12535 | CK945_RS12855 | ACH97_208675 |
| hemolysin III family protein | TRNA_RS33005 | BL14DL4_00541 | BLDA23_12545 | CK945_RS12865 | ACH97_208685 |
| hypothetical protein | TRNA_RS33010 | BL14DL4_00542 | BLDA23_12550 | CK945_RS12870 | ACH97_208690 |
| dihydrofolate reductase | TRNA_RS33015 | BL14DL4_00543 | BLDA23_12555 | CK945_RS12875 | ACH97_208695 |
| phosphatidylglycerophosphatase A | TRNA_RS33020 | BL14DL4_00544 | BLDA23_12565 | CK945_RS12885 | ACH97_208705 |
| hypothetical protein | TRNA_RS33025 | BL14DL4_00545 | BLDA23_12570 | CK945_RS12890 | ACH97_208710 |
| hypothetical protein | TRNA_RS33030 | BL14DL4_00546 | BLDA23_12575 | CK945_RS12895 | ACH97_208715 |
| BrxA/BrxB family bacilliredoxin | TRNA_RS33035 | BL14DL4_00547 | BLDA23_12580 | CK945_RS12900 | ACH97_208720 |
| dihydroxy-acid dehydratase | TRNA_RS33040 | BL14DL4_00548 | BLDA23_12585 | CK945_RS12905 | ACH97_208725 |
| PBS lyase | TRNA_RS33045 | BL14DL4_00549 | BLDA23_12590 | CK945_RS12910 | ACH97_208730 |
| glutathione peroxidase | TRNA_RS33055 | BL14DL4_00551 | BLDA23_12600 | CK945_RS12920 | ACH97_208740 |
| formate--tetrahydrofolate ligase | TRNA_RS33060 | BL14DL4_00552 | BLDA23_12605 | CK945_RS12925 | ACH97_208745 |
| homoserine O-succinyltransferase | TRNA_RS33065 | BL14DL4_00553 | BLDA23_12610 | CK945_RS12930 | ACH97_208750 |
| diglucosyl diacylglycerol synthase | TRNA_RS33070 | BL14DL4_00554 | BLDA23_12615 | CK945_RS12935 | ACH97_208755 |
| hypothetical protein | TRNA_RS33075 | BL14DL4_00555 | BLDA23_12620 | CK945_RS12940 | ACH97_208760 |
| cold-shock protein CspD | TRNA_RS33080 | BL14DL4_00556 | BLDA23_12625 | CK945_RS12945 | ACH97_208765 |
| hypothetical protein | TRNA_RS33085 | BL14DL4_00557 | BLDA23_12630 | CK945_RS12950 | ACH97_208770 |
| zinc-finger domain-containing protein | TRNA_RS33095 | BL14DL4_00559 | BLDA23_12640 | CK945_RS12960 | ACH97_208780 |
| hypothetical protein | TRNA_RS33100 | BL14DL4_00560 | BLDA23_12645 | CK945_RS12965 | ACH97_208785 |
| VUT family protein | TRNA_RS33105 | BL14DL4_00561 | BLDA23_12650 | CK945_RS12970 | ACH97_208790 |
| ribonuclease H | TRNA_RS33110 | BL14DL4_00562 | BLDA23_12655 | CK945_RS12975 | ACH97_208795 |
| small, acid-soluble spore protein L | TRNA_RS33115 | BL14DL4_00563 | BLDA23_12660 | CK945_RS12980 | ACH97_208800 |
| 5-3 exonuclease | TRNA_RS33120 | BL14DL4_00564 | BLDA23_12665 | CK945_RS12985 | ACH97_208805 |
| DUF2533 family protein | TRNA_RS33125 | BL14DL4_00566 | BLDA23_12670 | CK945_RS12995 | ACH97_208810 |
| hypothetical protein | TRNA_RS33140 | BL14DL4_00568 | BLDA23_12685 | CK945_RS13010 | ACH97_208825 |
| chalcone synthase | TRNA_RS33145 | BL14DL4_00569 | BLDA23_12690 | CK945_RS13015 | ACH97_208830 |
| N-acetyltransferase | TRNA_RS33150 | BL14DL4_00570 | BLDA23_12695 | CK945_RS13020 | ACH97_208835 |
| purine permease | TRNA_RS33155 | BL14DL4_00571 | BLDA23_12700 | CK945_RS13025 | ACH97_208840 |
| xanthine phosphoribosyltransferase | TRNA_RS33160 | BL14DL4_00572 | BLDA23_12705 | CK945_RS13030 | ACH97_208845 |
| carboxypeptidase M32 | TRNA_RS33165 | BL14DL4_00573 | BLDA23_12710 | CK945_RS13035 | ACH97_208850 |
| ATP-dependent DNA helicase | TRNA_RS33170 | BL14DL4_00574 | BLDA23_12715 | CK945_RS13040 | ACH97_208855 |
| hypothetical protein | TRNA_RS33175 | BL14DL4_00576 | BLDA23_12720 | CK945_RS13055 | ACH97_208870 |
| class I SAM-dependent RNA methyltransferase | TRNA_RS33180 | BL14DL4_00577 | BLDA23_12725 | CK945_RS13060 | ACH97_208875 |
| cell division regulator GpsB | TRNA_RS33185 | BL14DL4_00578 | BLDA23_12735 | CK945_RS13070 | ACH97_208880 |
| DUF1273 domain-containing protein | TRNA_RS33190 | BL14DL4_00579 | BLDA23_12740 | CK945_RS13075 | ACH97_208885 |
| spore coat protein CotH | TRNA_RS33195 | BL14DL4_00580 | BLDA23_12745 | CK945_RS13080 | ACH97_208890 |
| hypothetical protein | TRNA_RS33200 | BL14DL4_00582 | BLDA23_12750 | CK945_RS13085 | ACH97_208895 |
| DEAD/DEAH box helicase | TRNA_RS33205 | BL14DL4_00583 | BLDA23_12755 | CK945_RS13090 | ACH97_208900 |
| PTS glucose transporter subunit IIA | TRNA_RS33210 | BL14DL4_00584 | BLDA23_12760 | CK945_RS13095 | ACH97_208905 |
| hypothetical protein | TRNA_RS33215 | BL14DL4_00585 | BLDA23_12765 | CK945_RS13100 | ACH97_208910 |
| hypothetical protein | TRNA_RS33220 | BL14DL4_00586 | BLDA23_12770 | CK945_RS13105 | ACH97_208915 |
| hypothetical protein | TRNA_RS33225 | BL14DL4_00587 | BLDA23_12775 | CK945_RS13110 | ACH97_208920 |
| DUF1798 family protein | TRNA_RS33230 | BL14DL4_00588 | BLDA23_12780 | CK945_RS13115 | ACH97_208925 |
| hypothetical protein | TRNA_RS33235 | BL14DL4_00589 | BLDA23_12785 | CK945_RS13120 | ACH97_208930 |
| hypothetical protein | TRNA_RS33240 | BL14DL4_00590 | BLDA23_12790 | CK945_RS13125 | ACH97_208935 |
| DUF2515 domain-containing protein | TRNA_RS33245 | BL14DL4_00591 | BLDA23_12795 | CK945_RS13130 | ACH97_208940 |
| Holliday junction resolvase RecU | TRNA_RS33250 | BL14DL4_00592 | BLDA23_12800 | CK945_RS13135 | ACH97_208945 |
| PBP1A family penicillin-binding protein | TRNA_RS33255 | BL14DL4_00593 | BLDA23_12805 | CK945_RS13140 | ACH97_208950 |
| hypothetical protein | TRNA_RS33260 | BL14DL4_00594 | BLDA23_12810 | CK945_RS13145 | ACH97_208955 |
| endonuclease III | TRNA_RS33265 | BL14DL4_00595 | BLDA23_12815 | CK945_RS13150 | ACH97_208960 |
| DnaD domain protein | TRNA_RS33270 | BL14DL4_00596 | BLDA23_12820 | CK945_RS13155 | ACH97_208965 |
| asparagine--tRNA ligase | TRNA_RS33275 | BL14DL4_00597 | BLDA23_12825 | CK945_RS13160 | ACH97_208970 |
| pyridoxal phosphate-dependent aminotransferase | TRNA_RS33280 | BL14DL4_00598 | BLDA23_12830 | CK945_RS13165 | ACH97_208975 |
| hypothetical protein | TRNA_RS33285 | BL14DL4_00599 | BLDA23_12835 | CK945_RS13170 | ACH97_208980 |
| ATP-dependent helicase DinG | TRNA_RS33290 | BL14DL4_00601 | BLDA23_12845 | CK945_RS13180 | ACH97_208985 |
| aspartate 1-decarboxylase | TRNA_RS33295 | BL14DL4_00602 | BLDA23_12850 | CK945_RS13185 | ACH97_208990 |
| pantoate--beta-alanine ligase | TRNA_RS33300 | BL14DL4_00603 | BLDA23_12855 | CK945_RS13190 | ACH97_208995 |
| 3-methyl-2-oxobutanoate hydroxymethyltransferase | TRNA_RS33305 | BL14DL4_00604 | BLDA23_12860 | CK945_RS13195 | ACH97_209000 |
| bifunctional biotin--[acetyl-CoA-carboxylase] synthetase/biotin operon repressor | TRNA_RS33315 | BL14DL4_00605 | BLDA23_12870 | CK945_RS13205 | ACH97_209010 |
| CCA tRNA nucleotidyltransferase | TRNA_RS33320 | BL14DL4_00606 | BLDA23_12875 | CK945_RS13210 | ACH97_209015 |
| N-acetyl-alpha-D-glucosaminyl L-malate synthase BshA | TRNA_RS33325 | BL14DL4_00607 | BLDA23_12880 | CK945_RS13215 | ACH97_209020 |
| bacillithiol biosynthesis deacetylase BshB1 | TRNA_RS33330 | BL14DL4_00608 | BLDA23_12885 | CK945_RS13220 | ACH97_209025 |
| methylglyoxal synthase | TRNA_RS33335 | BL14DL4_00609 | BLDA23_12890 | CK945_RS13225 | ACH97_209030 |
| 4-hydroxy-tetrahydrodipicolinate reductase | TRNA_RS33340 | BL14DL4_00610 | BLDA23_12895 | CK945_RS13230 | ACH97_209035 |
| hypothetical protein | TRNA_RS33345 | BL14DL4_00611 | BLDA23_12900 | CK945_RS13235 | ACH97_209040 |
| YitT family protein | TRNA_RS33350 | BL14DL4_00612 | BLDA23_12905 | CK945_RS13240 | ACH97_209045 |
| sporulation protein YpjB | TRNA_RS33355 | BL14DL4_00613 | BLDA23_12910 | CK945_RS13245 | ACH97_209050 |
| DUF1405 domain-containing protein | TRNA_RS33360 | BL14DL4_00614 | BLDA23_12915 | CK945_RS13250 | ACH97_209055 |
| cytochrome CBB3 | TRNA_RS33365 | BL14DL4_00615 | BLDA23_12920 | CK945_RS13255 | ACH97_209060 |
| cytochrome b6 | TRNA_RS33370 | BL14DL4_00616 | BLDA23_12925 | CK945_RS13260 | ACH97_209065 |
| ubiquinol-cytochrome c reductase iron-sulfur subunit | TRNA_RS33375 | BL14DL4_00617 | BLDA23_12930 | CK945_RS13265 | ACH97_209070 |
| DUF2487 family protein | TRNA_RS33380 | BL14DL4_00618 | BLDA23_12935 | CK945_RS13270 | ACH97_209075 |
| YpiB family protein | TRNA_RS33385 | BL14DL4_00619 | BLDA23_12940 | CK945_RS13275 | ACH97_209080 |
| tetratricopeptide repeat protein | TRNA_RS33390 | BL14DL4_00620 | BLDA23_12945 | CK945_RS13280 | ACH97_209085 |
| 3-phosphoshikimate 1-carboxyvinyltransferase | TRNA_RS33395 | BL14DL4_00621 | BLDA23_12950 | CK945_RS13285 | ACH97_209090 |
| prephenate dehydrogenase | TRNA_RS33400 | BL14DL4_00622 | BLDA23_12955 | CK945_RS13290 | ACH97_209095 |
| histidinol-phosphate transaminase | TRNA_RS33405 | BL14DL4_00623 | BLDA23_12960 | CK945_RS13295 | ACH97_209100 |
| tryptophan synthase subunit alpha | TRNA_RS33410 | BL14DL4_00624 | BLDA23_12965 | CK945_RS13300 | ACH97_209105 |
| tryptophan synthase subunit beta | TRNA_RS33415 | BL14DL4_00625 | BLDA23_12970 | CK945_RS13305 | ACH97_209110 |
| phosphoribosylanthranilate isomerase | TRNA_RS33420 | BL14DL4_00626 | BLDA23_12975 | CK945_RS13310 | ACH97_209115 |
| indole-3-glycerol phosphate synthase TrpC | TRNA_RS33425 | BL14DL4_00627 | BLDA23_12980 | CK945_RS13315 | ACH97_209120 |
| anthranilate phosphoribosyltransferase | TRNA_RS33430 | BL14DL4_00628 | BLDA23_12985 | CK945_RS13320 | ACH97_209125 |
| anthranilate synthase component I | TRNA_RS33435 | BL14DL4_00629 | BLDA23_12990 | CK945_RS13325 | ACH97_209130 |
| chorismate mutase | TRNA_RS33440 | BL14DL4_00630 | BLDA23_12995 | CK945_RS13330 | ACH97_209135 |
| 3-dehydroquinate synthase | TRNA_RS33445 | BL14DL4_00631 | BLDA23_13000 | CK945_RS13335 | ACH97_209140 |
| chorismate synthase | TRNA_RS33450 | BL14DL4_00632 | BLDA23_13005 | CK945_RS13340 | ACH97_209145 |
| protein-glutamate O-methyltransferase CheR | TRNA_RS33455 | BL14DL4_00633 | BLDA23_13010 | CK945_RS13345 | ACH97_209150 |
| nucleoside-diphosphate kinase | TRNA_RS33460 | BL14DL4_00634 | BLDA23_13015 | CK945_RS13350 | ACH97_209155 |
| heptaprenyl diphosphate synthase component II | TRNA_RS33465 | BL14DL4_00635 | BLDA23_13020 | CK945_RS13355 | ACH97_209160 |
| demethylmenaquinone methyltransferase | TRNA_RS33470 | BL14DL4_00636 | BLDA23_13025 | CK945_RS13360 | ACH97_209165 |
| heptaprenyl diphosphate synthase component 1 | TRNA_RS33475 | BL14DL4_00637 | BLDA23_13030 | CK945_RS13365 | ACH97_209170 |
| trp RNA-binding attenuation protein MtrB | TRNA_RS33480 | BL14DL4_00638 | BLDA23_13035 | CK945_RS13370 | ACH97_209175 |
| GTP cyclohydrolase I FolE | TRNA_RS33485 | BL14DL4_00639 | BLDA23_13040 | CK945_RS13375 | ACH97_209180 |
| DNA-binding protein HU 1 | TRNA_RS33490 | BL14DL4_00640 | BLDA23_13045 | CK945_RS13380 | ACH97_209185 |
| stage IV sporulation protein A | TRNA_RS33495 | BL14DL4_00641 | BLDA23_13050 | CK945_RS13385 | ACH97_209190 |
| hypothetical protein | TRNA_RS33500 | BL14DL4_00642 | BLDA23_13055 | CK945_RS13390 | ACH97_209195 |
| DUF2768 domain-containing protein | TRNA_RS33505 | BL14DL4_00643 | BLDA23_13060 | CK945_RS13395 | ACH97_209200 |
| ribosome biogenesis GTPase Der | TRNA_RS33520 | BL14DL4_00645 | BLDA23_13075 | CK945_RS13410 | ACH97_209210 |
| hypothetical protein | TRNA_RS33525 | BL14DL4_00646 | BLDA23_13080 | CK945_RS13415 | ACH97_209215 |
| membrane protein | TRNA_RS33530 | BL14DL4_00647 | BLDA23_13085 | CK945_RS13420 | ACH97_209220 |
| hypothetical protein | TRNA_RS33535 | BL14DL4_00648 | BLDA23_13090 | CK945_RS13425 | ACH97_209225 |
| type 2 isopentenyl-diphosphate Delta-isomerase | TRNA_RS33540 | BL14DL4_00650 | BLDA23_13100 | CK945_RS13435 | ACH97_209230 |
| 30S ribosomal protein S1 | TRNA_RS33545 | BL14DL4_00651 | BLDA23_13105 | CK945_RS13440 | ACH97_209235 |
| (d)CMP kinase | TRNA_RS33550 | BL14DL4_00652 | BLDA23_13110 | CK945_RS13445 | ACH97_209240 |
| germination protein YpeB | TRNA_RS33555 | BL14DL4_00654 | BLDA23_13115 | CK945_RS13455 | ACH97_209245 |
| spore cortex-lytic enzyme | TRNA_RS33560 | BL14DL4_00655 | BLDA23_13120 | CK945_RS13460 | ACH97_209250 |
| protease PrsW | TRNA_RS33565 | BL14DL4_00656 | BLDA23_13125 | CK945_RS13465 | ACH97_209255 |
| asparaginase | TRNA_RS33570 | BL14DL4_00657 | BLDA23_13130 | CK945_RS13470 | ACH97_209260 |
| YpdA family putative bacillithiol disulfide reductase | TRNA_RS33575 | BL14DL4_00658 | BLDA23_13135 | CK945_RS13475 | ACH97_209265 |
| Glu/Leu/Phe/Val dehydrogenase | TRNA_RS33580 | BL14DL4_00659 | BLDA23_13140 | CK945_RS13480 | ACH97_209270 |
| genetic competence negative regulator | TRNA_RS33585 | BL14DL4_00660 | BLDA23_13145 | CK945_RS13485 | ACH97_209275 |
| hypothetical protein | TRNA_RS33590 | BL14DL4_00661 | BLDA23_13150 | CK945_RS13490 | ACH97_209280 |
| DUF2663 family protein | TRNA_RS33595 | BL14DL4_00662 | BLDA23_13155 | CK945_RS13495 | ACH97_209285 |
| LysM peptidoglycan-binding domain-containing protein | TRNA_RS33600 | BL14DL4_00663 | BLDA23_13160 | CK945_RS13500 | ACH97_209290 |
| CPBP family intramembrane metalloprotease | TRNA_RS33605 | BL14DL4_00664 | BLDA23_13165 | CK945_RS13505 | ACH97_209295 |
| ATP-dependent DNA helicase RecQ | TRNA_RS33610 | BL14DL4_00665 | BLDA23_13170 | CK945_RS13510 | ACH97_209300 |
| protein YpbB | TRNA_RS33615 | BL14DL4_00666 | BLDA23_13175 | CK945_RS13515 | ACH97_209305 |
| ferredoxin | TRNA_RS33620 | BL14DL4_00667 | BLDA23_13180 | CK945_RS13520 | ACH97_209310 |
| ECF transporter S component | TRNA_RS33625 | BL14DL4_00668 | BLDA23_13185 | CK945_RS13525 | ACH97_209315 |
| phosphoglycerate dehydrogenase | TRNA_RS33630 | BL14DL4_00669 | BLDA23_13190 | CK945_RS13530 | ACH97_209320 |
| LysM peptidoglycan-binding domain-containing protein | TRNA_RS33635 | BL14DL4_00670 | BLDA23_13195 | CK945_RS13535 | ACH97_209325 |
| hypothetical protein | TRNA_RS33640 | BL14DL4_00671 | BLDA23_13200 | CK945_RS13540 | ACH97_209330 |
| RNA polymerase sigma factor SigX | TRNA_RS33645 | BL14DL4_00672 | BLDA23_13205 | CK945_RS13545 | ACH97_209335 |
| transcriptional regulator | TRNA_RS33650 | BL14DL4_00673 | BLDA23_13210 | CK945_RS13550 | ACH97_209340 |
| WD40 repeat domain-containing protein | TRNA_RS33660 | BL14DL4_00675 | BLDA23_13220 | CK945_RS13560 | ACH97_209350 |
| sensor histidine kinase | TRNA_RS33665 | BL14DL4_00676 | BLDA23_13225 | CK945_RS13565 | ACH97_209355 |
| DNA-binding response regulator | TRNA_RS33670 | BL14DL4_00677 | BLDA23_13230 | CK945_RS13570 | ACH97_209360 |
| PAS domain-containing sensor histidine kinase | TRNA_RS33675 | BL14DL4_00678 | BLDA23_13235 | CK945_RS13575 | ACH97_209365 |
| DNA-binding response regulator | TRNA_RS33680 | BL14DL4_00679 | BLDA23_13240 | CK945_RS13580 | ACH97_209370 |
| c-type cytochrome biogenesis protein CcsB | TRNA_RS33685 | BL14DL4_00680 | BLDA23_13245 | CK945_RS13585 | ACH97_209375 |
| cytochrome c biogenesis protein | TRNA_RS33690 | BL14DL4_00681 | BLDA23_13250 | CK945_RS13590 | ACH97_209380 |
| thiol-disulfide oxidoreductase ResA | TRNA_RS33695 | BL14DL4_00682 | BLDA23_13255 | CK945_RS13595 | ACH97_209385 |
| rRNA pseudouridine synthase | TRNA_RS33700 | BL14DL4_00683 | BLDA23_13260 | CK945_RS13600 | ACH97_209390 |
| spore maturation protein | TRNA_RS33705 | BL14DL4_00684 | BLDA23_13265 | CK945_RS13605 | ACH97_209395 |
| spore maturation protein | TRNA_RS33710 | BL14DL4_00685 | BLDA23_13270 | CK945_RS13610 | ACH97_209400 |
| D-alanyl-D-alanine carboxypeptidase | TRNA_RS33715 | BL14DL4_00686 | BLDA23_13275 | CK945_RS13615 | ACH97_209405 |
| DUF3907 family protein | TRNA_RS33720 | BL14DL4_00687 | BLDA23_13280 | CK945_RS13620 | ACH97_209410 |
| SMC-Scp complex subunit ScpB | TRNA_RS33725 | BL14DL4_00688 | BLDA23_13285 | CK945_RS13625 | ACH97_209415 |
| segregation/condensation protein A | TRNA_RS33730 | BL14DL4_00689 | BLDA23_13290 | CK945_RS13630 | ACH97_209420 |
| DUF309 domain-containing protein | TRNA_RS33735 | BL14DL4_00691 | BLDA23_13300 | CK945_RS13640 | ACH97_209425 |
| GNAT family N-acetyltransferase | TRNA_RS33740 | BL14DL4_00692 | BLDA23_13305 | CK945_RS13645 | ACH97_209430 |
| 6,7-dimethyl-8-ribityllumazine synthase | TRNA_RS33745 | BL14DL4_00693 | BLDA23_13310 | CK945_RS13650 | ACH97_209435 |
| bifunctional 3,4-dihydroxy-2-butanone-4-phosphate synthase/GTP cyclohydrolase II | TRNA_RS33750 | BL14DL4_00694 | BLDA23_13315 | CK945_RS13655 | ACH97_209440 |
| riboflavin synthase | TRNA_RS33755 | BL14DL4_00695 | BLDA23_13320 | CK945_RS13660 | ACH97_209445 |
| bifunctional diaminohydroxyphosphoribosylaminopyrimidine deaminase/5-amino-6-(5-phosphoribosylamino)uracil reductase RibD | TRNA_RS33760 | BL14DL4_00696 | BLDA23_13325 | CK945_RS13665 | ACH97_209450 |
| hypothetical protein | TRNA_RS33765 | BL14DL4_00697 | BLDA23_13330 | CK945_RS13670 | ACH97_209455 |
| phosphatase | TRNA_RS33770 | BL14DL4_00698 | BLDA23_13335 | CK945_RS13675 | ACH97_209460 |
| hypothetical protein | TRNA_RS33780 | BL14DL4_00700 | BLDA23_13345 | CK945_RS13680 | ACH97_209465 |
| tetratricopeptide repeat protein | TRNA_RS33785 | BL14DL4_00701 | BLDA23_13350 | CK945_RS13685 | ACH97_209470 |
| peptidylprolyl isomerase | TRNA_RS33790 | BL14DL4_00702 | BLDA23_13355 | CK945_RS13690 | ACH97_209475 |
| DUF1002 domain-containing protein | TRNA_RS33795 | BL14DL4_00703 | BLDA23_13360 | CK945_RS13695 | ACH97_209480 |
| diaminopimelate decarboxylase | TRNA_RS33815 | BL14DL4_00705 | BLDA23_13370 | CK945_RS13710 | ACH97_209495 |
| hypothetical protein | TRNA_RS33820 | BL14DL4_00706 | BLDA23_13375 | CK945_RS13715 | ACH97_209500 |
| spore germination protein | TRNA_RS33825 | BL14DL4_00707 | BLDA23_13380 | CK945_RS13720 | ACH97_209505 |
| stage V sporulation protein AE | TRNA_RS33830 | BL14DL4_00708 | BLDA23_13385 | CK945_RS13725 | ACH97_209510 |
| stage V sporulation protein AE | TRNA_RS33835 | BL14DL4_00709 | BLDA23_13390 | CK945_RS13730 | ACH97_209515 |
| stage V sporulation protein AD | TRNA_RS33840 | BL14DL4_00710 | BLDA23_13395 | CK945_RS13735 | ACH97_209520 |
| stage V sporulation protein AC | TRNA_RS33845 | BL14DL4_00711 | BLDA23_13400 | CK945_RS13740 | ACH97_209525 |
| stage V sporulation protein AB | TRNA_RS33850 | BL14DL4_00712 | BLDA23_13405 | CK945_RS13745 | ACH97_209530 |
| stage V sporulation protein AA | TRNA_RS33855 | BL14DL4_00713 | BLDA23_13410 | CK945_RS13750 | ACH97_209535 |
| RNA polymerase sporulation sigma factor SigF | TRNA_RS33860 | BL14DL4_00714 | BLDA23_13415 | CK945_RS13755 | ACH97_209540 |
| anti-sigma F factor | TRNA_RS33865 | BL14DL4_00715 | BLDA23_13420 | CK945_RS13760 | ACH97_209545 |
| anti-sigma F factor antagonist | TRNA_RS33870 | BL14DL4_00716 | BLDA23_13425 | CK945_RS13765 | ACH97_209550 |
| D-alanyl-D-alanine carboxypeptidase | TRNA_RS33875 | BL14DL4_00717 | BLDA23_13430 | CK945_RS13770 | ACH97_209555 |
| purine-nucleoside phosphorylase | TRNA_RS33880 | BL14DL4_00718 | BLDA23_13435 | CK945_RS13775 | ACH97_209560 |
| phosphopentomutase | TRNA_RS33885 | BL14DL4_00719 | BLDA23_13440 | CK945_RS13780 | ACH97_209565 |
| site-specific tyrosine recombinase XerD | TRNA_RS33890 | BL14DL4_00720 | BLDA23_13445 | CK945_RS13785 | ACH97_209570 |
| DUF4227 family protein | TRNA_RS33895 | BL14DL4_00721 | BLDA23_13450 | CK945_RS13790 | ACH97_209575 |
| transcriptional repressor | TRNA_RS33900 | BL14DL4_00722 | BLDA23_13455 | CK945_RS13795 | ACH97_209580 |
| stage II sporulation protein M | TRNA_RS33905 | BL14DL4_00723 | BLDA23_13460 | CK945_RS13800 | ACH97_209585 |
| PTS sugar transporter subunit IIC | TRNA_RS33910 | BL14DL4_00724 | BLDA23_13465 | CK945_RS13805 | ACH97_209590 |
| PTS sugar transporter subunit IIB | TRNA_RS33915 | BL14DL4_00725 | BLDA23_13470 | CK945_RS13810 | ACH97_209595 |
| N-acetyltransferase | TRNA_RS33930 | BL14DL4_00728 | BLDA23_13480 | CK945_RS13825 | ACH97_209610 |
| GNAT family N-acetyltransferase | TRNA_RS33935 | BL14DL4_00730 | BLDA23_13485 | CK945_RS13830 | ACH97_209615 |
| TIGR00375 family protein | TRNA_RS33940 | BL14DL4_00731 | BLDA23_13490 | CK945_RS13835 | ACH97_209620 |
| NUDIX hydrolase | TRNA_RS33945 | BL14DL4_00732 | BLDA23_13495 | CK945_RS13840 | ACH97_209625 |
| TetR/AcrR family transcriptional regulator | TRNA_RS33950 | BL14DL4_00734 | BLDA23_13505 | CK945_RS13850 | ACH97_209630 |
| DoxX family protein | TRNA_RS33955 | BL14DL4_00735 | BLDA23_13510 | CK945_RS13855 | ACH97_209635 |
| aldo/keto reductase | TRNA_RS33960 | BL14DL4_00736 | BLDA23_13515 | CK945_RS13860 | ACH97_209640 |
| DUF3886 domain-containing protein | TRNA_RS33965 | BL14DL4_00737 | BLDA23_13520 | CK945_RS13865 | ACH97_209645 |
| alpha/beta hydrolase | TRNA_RS33970 | BL14DL4_00738 | BLDA23_13525 | CK945_RS13870 | ACH97_209650 |
| DUF2552 domain-containing protein | TRNA_RS33975 | BL14DL4_00739 | BLDA23_13530 | CK945_RS13875 | ACH97_209655 |
| hypothetical protein | TRNA_RS33980 | BL14DL4_00740 | BLDA23_13535 | CK945_RS13880 | ACH97_209660 |
| antibiotic biosynthesis monooxygenase | TRNA_RS33995 | BL14DL4_00743 | BLDA23_13550 | CK945_RS13895 | ACH97_209675 |
| triacylglycerol lipase | TRNA_RS34005 | BL14DL4_00744 | BLDA23_13560 | CK945_RS13900 | ACH97_209680 |
| hypothetical protein | TRNA_RS34010 | BL14DL4_00745 | BLDA23_13565 | CK945_RS13905 | ACH97_209685 |
| ABC transporter substrate-binding protein | TRNA_RS34015 | BL14DL4_00746 | BLDA23_13570 | CK945_RS13910 | ACH97_209690 |
| ABC transporter permease | TRNA_RS34020 | BL14DL4_00747 | BLDA23_13575 | CK945_RS13915 | ACH97_209695 |
| ABC transporter permease subunit | TRNA_RS34025 | BL14DL4_00748 | BLDA23_13580 | CK945_RS13920 | ACH97_209700 |
| MATE family efflux transporter | TRNA_RS34040 | BL14DL4_00751 | BLDA23_13590 | CK945_RS13935 | ACH97_209715 |
| elongation factor G-binding protein | TRNA_RS34045 | BL14DL4_00752 | BLDA23_13595 | CK945_RS13940 | ACH97_209720 |
| YolD-like family protein | TRNA_RS34050 | BL14DL4_00754 | BLDA23_13600 | CK945_RS13945 | ACH97_209725 |
| UV-damage repair protein uvrX | TRNA_RS34055 | BL14DL4_00755 | BLDA23_13605 | CK945_RS13950 | ACH97_209730 |
| hypothetical protein | TRNA_RS34060 | BL14DL4_00756 | BLDA23_13610 | CK945_RS13955 | ACH97_209735 |
| MFS transporter | TRNA_RS34065 | BL14DL4_00757 | BLDA23_13615 | CK945_RS13960 | ACH97_209740 |
| hypothetical protein | TRNA_RS34070 | BL14DL4_00758 | BLDA23_13620 | CK945_RS13965 | ACH97_209745 |
| type I pantothenate kinase | TRNA_RS34075 | BL14DL4_00759 | BLDA23_13625 | CK945_RS13970 | ACH97_209750 |
| ABC transporter permease | TRNA_RS34085 | BL14DL4_00760 | BLDA23_13635 | CK945_RS13975 | ACH97_209755 |
| ATP-binding cassette domain-containing protein | TRNA_RS34090 | BL14DL4_00761 | BLDA23_13640 | CK945_RS13980 | ACH97_209760 |
| hypothetical protein | TRNA_RS34095 | BL14DL4_00762 | BLDA23_13645 | CK945_RS13985 | ACH97_209765 |
| hypothetical protein | TRNA_RS34100 | BL14DL4_00763 | BLDA23_13650 | CK945_RS12270 | ACH97_207240 |
| DUF3189 family protein | TRNA_RS34105 | BL14DL4_00764 | BLDA23_13655 | CK945_RS14050 | ACH97_209820 |
| hypothetical protein | TRNA_RS34110 | BL14DL4_00765 | BLDA23_13660 | CK945_RS14055 | ACH97_209825 |
| SDR family oxidoreductase | TRNA_RS34120 | BL14DL4_00766 | BLDA23_13670 | CK945_RS14065 | ACH97_209860 |
| MBL fold metallo-hydrolase | TRNA_RS34125 | BL14DL4_00767 | BLDA23_13675 | CK945_RS14070 | ACH97_209865 |
| pyrroline-5-carboxylate reductase | TRNA_RS34130 | BL14DL4_00768 | BLDA23_13680 | CK945_RS14075 | ACH97_209870 |
| M20/M25/M40 family metallo-hydrolase | TRNA_RS34135 | BL14DL4_00769 | BLDA23_13685 | CK945_RS14080 | ACH97_209875 |
| NADPH dehydrogenase NamA | TRNA_RS34140 | BL14DL4_00770 | BLDA23_13690 | CK945_RS14085 | ACH97_209880 |
| alpha/beta hydrolase | TRNA_RS34145 | BL14DL4_00771 | BLDA23_13700 | CK945_RS14090 | ACH97_209885 |
| 50S ribosomal protein L33 | TRNA_RS34150 | BL14DL4_00772 | BLDA23_13705 | CK945_RS14095 | ACH97_209890 |
| ribonuclease Z | TRNA_RS34155 | BL14DL4_00773 | BLDA23_13710 | CK945_RS14100 | ACH97_209895 |
| glucose-6-phosphate dehydrogenase | TRNA_RS34160 | BL14DL4_00774 | BLDA23_13715 | CK945_RS14105 | ACH97_209900 |
| TRAP transporter permease | TRNA_RS34165 | BL14DL4_00775 | BLDA23_13720 | CK945_RS14110 | ACH97_209905 |
| DUF1850 domain-containing protein | TRNA_RS34170 | BL14DL4_00776 | BLDA23_13725 | CK945_RS14115 | ACH97_209910 |
| hypothetical protein | TRNA_RS34175 | BL14DL4_00777 | BLDA23_13730 | CK945_RS14120 | ACH97_209915 |
| GntR family transcriptional regulator | TRNA_RS34180 | BL14DL4_00778 | BLDA23_13735 | CK945_RS14125 | ACH97_209920 |
| glycoside hydrolase family 1 protein | TRNA_RS34185 | BL14DL4_00779 | BLDA23_13740 | CK945_RS14130 | ACH97_209925 |
| PTS lactose/cellobiose transporter subunit IIA | TRNA_RS34195 | BL14DL4_00781 | BLDA23_13750 | CK945_RS14140 | ACH97_209935 |
| PTS sugar transporter subunit IIB | TRNA_RS34200 | BL14DL4_00782 | BLDA23_13755 | CK945_RS14145 | ACH97_209940 |
| SDR family oxidoreductase | TRNA_RS34210 | BL14DL4_00784 | BLDA23_13765 | CK945_RS14155 | ACH97_209950 |
| NADP-dependent phosphogluconate dehydrogenase | TRNA_RS34215 | BL14DL4_00785 | BLDA23_13770 | CK945_RS14160 | ACH97_209955 |
| DNA polymerase IV | TRNA_RS34220 | BL14DL4_00786 | BLDA23_13775 | CK945_RS14165 | ACH97_209960 |
| hypothetical protein | TRNA_RS34225 | BL14DL4_00787 | BLDA23_13780 | CK945_RS14170 | ACH97_209965 |
| membrane protein insertase YidC | TRNA_RS34230 | BL14DL4_00788 | BLDA23_13785 | CK945_RS14175 | ACH97_209970 |
| M20/M25/M40 family metallo-hydrolase | TRNA_RS34235 | BL14DL4_00789 | BLDA23_13790 | CK945_RS14180 | ACH97_209975 |
| acyl-CoA carboxylase subunit beta | TRNA_RS34240 | BL14DL4_00790 | BLDA23_13795 | CK945_RS14185 | ACH97_209980 |
| aromatic acid exporter family protein | TRNA_RS34245 | BL14DL4_00791 | BLDA23_13800 | CK945_RS14190 | ACH97_209985 |
| amino acid ABC transporter ATP-binding protein | TRNA_RS34250 | BL14DL4_00792 | BLDA23_13805 | CK945_RS14195 | ACH97_209990 |
| amino acid ABC transporter permease | TRNA_RS34255 | BL14DL4_00793 | BLDA23_13810 | CK945_RS14200 | ACH97_209995 |
| BrxA/BrxB family bacilliredoxin | TRNA_RS34265 | BL14DL4_00796 | BLDA23_13820 | CK945_RS14210 | ACH97_210005 |
| YegS/Rv2252/BmrU family lipid kinase | TRNA_RS34270 | BL14DL4_00797 | BLDA23_13825 | CK945_RS14215 | ACH97_210010 |
| 2-oxo acid dehydrogenase subunit E2 | TRNA_RS34275 | BL14DL4_00798 | BLDA23_13830 | CK945_RS14220 | ACH97_210015 |
| alpha-ketoacid dehydrogenase subunit beta | TRNA_RS34280 | BL14DL4_00799 | BLDA23_13835 | CK945_RS14225 | ACH97_210020 |
| thiamine pyrophosphate-dependent dehydrogenase E1 component subunit alpha | TRNA_RS34285 | BL14DL4_00800 | BLDA23_13840 | CK945_RS14230 | ACH97_210025 |
| dihydrolipoyl dehydrogenase | TRNA_RS34290 | BL14DL4_00801 | BLDA23_13845 | CK945_RS14235 | ACH97_210030 |
| butyrate kinase | TRNA_RS34295 | BL14DL4_00802 | BLDA23_13850 | CK945_RS14240 | ACH97_210035 |
| Glu/Leu/Phe/Val dehydrogenase | TRNA_RS34300 | BL14DL4_00803 | BLDA23_13855 | CK945_RS14245 | ACH97_210040 |
| phosphate butyryltransferase | TRNA_RS34305 | BL14DL4_00804 | BLDA23_13860 | CK945_RS14250 | ACH97_210045 |
| sigma-54-dependent Fis family transcriptional regulator | TRNA_RS34310 | BL14DL4_00805 | BLDA23_13865 | CK945_RS14255 | ACH97_210050 |
| DUF2627 domain-containing protein | TRNA_RS34315 | BL14DL4_00806 | BLDA23_13870 | CK945_RS14260 | ACH97_210055 |
| glycerophosphodiester phosphodiesterase | TRNA_RS34320 | BL14DL4_00807 | BLDA23_13875 | CK945_RS14265 | ACH97_210060 |
| N-acetylmuramoyl-L-alanine amidase | TRNA_RS34325 | BL14DL4_00808 | BLDA23_13880 | CK945_RS14270 | ACH97_210065 |
| hypothetical protein | TRNA_RS34330 | BL14DL4_00809 | BLDA23_13885 | CK945_RS14275 | ACH97_210070 |
| sporulation transcription factor Spo0A | TRNA_RS34350 | BL14DL4_00813 | BLDA23_13905 | CK945_RS14295 | ACH97_210080 |
| SpoIVB peptidase | TRNA_RS34355 | BL14DL4_00814 | BLDA23_13910 | CK945_RS14300 | ACH97_210085 |
| DNA repair protein RecN | TRNA_RS34360 | BL14DL4_00815 | BLDA23_13915 | CK945_RS14305 | ACH97_210090 |
| arginine repressor | TRNA_RS34365 | BL14DL4_00816 | BLDA23_13920 | CK945_RS14310 | ACH97_210095 |
| TlyA family rRNA (cytidine-2-O)-methyltransferase | TRNA_RS34370 | BL14DL4_00817 | BLDA23_13925 | CK945_RS14315 | ACH97_210100 |
| 1-deoxy-D-xylulose-5-phosphate synthase | TRNA_RS34375 | BL14DL4_00818 | BLDA23_13930 | CK945_RS14320 | ACH97_210105 |
| polyprenyl synthetase family protein | TRNA_RS34380 | BL14DL4_00819 | BLDA23_13935 | CK945_RS14325 | ACH97_210110 |
| exodeoxyribonuclease 7 small subunit | TRNA_RS34385 | BL14DL4_00820 | BLDA23_13940 | CK945_RS14330 | ACH97_210115 |
| exodeoxyribonuclease VII large subunit | TRNA_RS34390 | BL14DL4_00821 | BLDA23_13945 | CK945_RS14335 | ACH97_210120 |
| bifunctional methylenetetrahydrofolate dehydrogenase/methenyltetrahydrofolate cyclohydrolase | TRNA_RS34395 | BL14DL4_00822 | BLDA23_13950 | CK945_RS14340 | ACH97_210125 |
| N utilization substance protein B | TRNA_RS34400 | BL14DL4_00823 | BLDA23_13955 | CK945_RS14345 | ACH97_210130 |
| Asp23/Gls24 family envelope stress response protein | TRNA_RS34405 | BL14DL4_00824 | BLDA23_13960 | CK945_RS14350 | ACH97_210135 |
| acetyl-CoA carboxylase biotin carboxylase subunit | TRNA_RS34410 | BL14DL4_00825 | BLDA23_13965 | CK945_RS14355 | ACH97_210140 |
| acetyl-CoA carboxylase biotin carboxyl carrier protein | TRNA_RS34415 | BL14DL4_00826 | BLDA23_13970 | CK945_RS14360 | ACH97_210145 |
| SpoIIIAH-like family protein | TRNA_RS34420 | BL14DL4_00827 | BLDA23_13975 | CK945_RS14365 | ACH97_210150 |
| stage III sporulation protein AG | TRNA_RS34425 | BL14DL4_00828 | BLDA23_13980 | CK945_RS14370 | ACH97_210155 |
| stage III sporulation protein AF | TRNA_RS34430 | BL14DL4_00829 | BLDA23_13985 | CK945_RS14375 | ACH97_210160 |
| stage III sporulation protein AE | TRNA_RS34435 | BL14DL4_00830 | BLDA23_13990 | CK945_RS14380 | ACH97_210165 |
| stage III sporulation protein AD | TRNA_RS34440 | BL14DL4_00831 | BLDA23_13995 | CK945_RS14385 | ACH97_210170 |
| stage III sporulation protein AC | TRNA_RS34445 | BL14DL4_00832 | BLDA23_14000 | CK945_RS14390 | ACH97_210175 |
| stage III sporulation protein SpoAB | TRNA_RS34450 | BL14DL4_00833 | BLDA23_14005 | CK945_RS14395 | ACH97_210180 |
| stage III sporulation protein AA | TRNA_RS34455 | BL14DL4_00834 | BLDA23_14010 | CK945_RS14400 | ACH97_210185 |
| DUF2619 domain-containing protein | TRNA_RS34460 | BL14DL4_00835 | BLDA23_14015 | CK945_RS14405 | ACH97_210190 |
| elongation factor P | TRNA_RS34465 | BL14DL4_00836 | BLDA23_14020 | CK945_RS14410 | ACH97_210195 |
| aminopeptidase P family protein | TRNA_RS34470 | BL14DL4_00837 | BLDA23_14025 | CK945_RS14415 | ACH97_210200 |
| type II 3-dehydroquinate dehydratase | TRNA_RS34475 | BL14DL4_00838 | BLDA23_14030 | CK945_RS14420 | ACH97_210205 |
| hypothetical protein | TRNA_RS34480 | BL14DL4_00839 | BLDA23_14035 | CK945_RS14425 | ACH97_210210 |
| DUF1385 domain-containing protein | TRNA_RS34485 | BL14DL4_00840 | BLDA23_14040 | CK945_RS14430 | ACH97_210215 |
| hypothetical protein | TRNA_RS34490 | BL14DL4_00841 | BLDA23_14045 | CK945_RS14435 | ACH97_210220 |
| hypothetical protein | TRNA_RS34495 | BL14DL4_00842 | BLDA23_14050 | CK945_RS14440 | ACH97_210225 |
| transcriptional regulator MntR | TRNA_RS34505 | BL14DL4_00843 | BLDA23_14055 | CK945_RS14445 | ACH97_210230 |
| lipoate--protein ligase family protein | TRNA_RS34510 | BL14DL4_00844 | BLDA23_14060 | CK945_RS14450 | ACH97_210235 |
| rhodanese-like domain-containing protein | TRNA_RS34515 | BL14DL4_00845 | BLDA23_14065 | CK945_RS14455 | ACH97_210240 |
| glycine/betaine ABC transporter | TRNA_RS34520 | BL14DL4_00846 | BLDA23_14070 | CK945_RS14460 | ACH97_210245 |
| proline/glycine betaine ABC transporter permease | TRNA_RS34525 | BL14DL4_00847 | BLDA23_14075 | CK945_RS14465 | ACH97_210250 |
| glycine betaine/L-proline ABC transporter ATP-binding protein | TRNA_RS34530 | BL14DL4_00848 | BLDA23_14080 | CK945_RS14470 | ACH97_210255 |
| glycine dehydrogenase | TRNA_RS34535 | BL14DL4_00849 | BLDA23_14085 | CK945_RS14475 | ACH97_210260 |
| aminomethyl-transferring glycine dehydrogenase subunit GcvPA | TRNA_RS34540 | BL14DL4_00850 | BLDA23_14090 | CK945_RS14480 | ACH97_210265 |
| glycine cleavage system aminomethyltransferase GcvT | TRNA_RS34545 | BL14DL4_00851 | BLDA23_14095 | CK945_RS14485 | ACH97_210270 |
| DEAD/DEAH box helicase | TRNA_RS34555 | BL14DL4_00852 | BLDA23_14100 | CK945_RS14495 | ACH97_210280 |
| hypothetical protein | TRNA_RS34560 | BL14DL4_00853 | BLDA23_14105 | CK945_RS14500 | ACH97_210285 |
| DNA-binding anti-repressor SinI | TRNA_RS34565 | BL14DL4_00854 | BLDA23_14110 | CK945_RS14505 | ACH97_210290 |
| transcriptional regulator | TRNA_RS34570 | BL14DL4_00855 | BLDA23_14115 | CK945_RS14510 | ACH97_210295 |
| spore coat protein | TRNA_RS34575 | BL14DL4_00856 | BLDA23_14120 | CK945_RS14515 | ACH97_210300 |
| signal peptidase I | TRNA_RS34580 | BL14DL4_00857 | BLDA23_14125 | CK945_RS14520 | ACH97_210305 |
| amyloid fiber anchoring/assembly protein TapA | TRNA_RS34585 | BL14DL4_00858 | BLDA23_14130 | CK945_RS14525 | ACH97_210310 |
| DUF3889 domain-containing protein | TRNA_RS34590 | BL14DL4_00859 | BLDA23_14135 | CK945_RS14530 | ACH97_210315 |
| YqzE family protein | TRNA_RS34595 | BL14DL4_00860 | BLDA23_14140 | CK945_RS14535 | ACH97_210320 |
| DNA segregation ATPase | TRNA_RS34600 | BL14DL4_00861 | BLDA23_14145 | CK945_RS14540 | ACH97_210325 |
| DNA segregation ATPase | TRNA_RS34605 | BL14DL4_00862 | BLDA23_14150 | CK945_RS14545 | ACH97_210330 |
| prepilin-type N-terminal cleavage/methylation domain-containing protein | TRNA_RS34615 | BL14DL4_00863 | BLDA23_14160 | CK945_RS14555 | ACH97_210340 |
| prepilin-type N-terminal cleavage/methylation domain-containing protein | TRNA_RS34620 | BL14DL4_00864 | BLDA23_14165 | CK945_RS14560 | ACH97_210345 |
| type II secretion system F family protein | TRNA_RS34625 | BL14DL4_00865 | BLDA23_14170 | CK945_RS14925 | ACH97_210350 |
| type II/IV secretion system protein | TRNA_RS34630 | BL14DL4_00866 | BLDA23_14175 | CK945_RS14930 | ACH97_210355 |
| STAS domain-containing protein | TRNA_RS34635 | BL14DL4_00867 | BLDA23_14180 | CK945_RS14935 | ACH97_210360 |
| transcriptional regulator Spx | TRNA_RS34645 | BL14DL4_00869 | BLDA23_14190 | CK945_RS14945 | ACH97_210370 |
| DUF2626 domain-containing protein | TRNA_RS34650 | BL14DL4_00870 | BLDA23_14195 | CK945_RS14950 | ACH97_210375 |
| MBL fold metallo-hydrolase | TRNA_RS34655 | BL14DL4_00871 | BLDA23_14200 | CK945_RS14955 | ACH97_210380 |
| thiamine-binding protein | TRNA_RS34665 | BL14DL4_00873 | BLDA23_14210 | CK945_RS14965 | ACH97_210385 |
| hypothetical protein | TRNA_RS34670 | BL14DL4_00874 | BLDA23_14215 | CK945_RS14970 | ACH97_210390 |
| hypothetical protein | TRNA_RS34675 | BL14DL4_00875 | BLDA23_14220 | CK945_RS14975 | ACH97_210395 |
| iron-hydroxamate ABC transporter substrate-binding protein | TRNA_RS34680 | BL14DL4_00876 | BLDA23_14225 | CK945_RS14980 | ACH97_210400 |
| LTA synthase family protein | TRNA_RS34685 | BL14DL4_00878 | BLDA23_14230 | CK945_RS14985 | ACH97_210405 |
| ROK family protein | TRNA_RS34690 | BL14DL4_00879 | BLDA23_14235 | CK945_RS14990 | ACH97_210410 |
| spore germination protein | TRNA_RS34700 | BL14DL4_00880 | BLDA23_14245 | CK945_RS15000 | ACH97_210420 |
| rhomboid family intramembrane serine protease | TRNA_RS34705 | BL14DL4_00881 | BLDA23_14250 | CK945_RS15005 | ACH97_210425 |
| 5-formyltetrahydrofolate cyclo-ligase | TRNA_RS34710 | BL14DL4_00883 | BLDA23_14255 | CK945_RS15015 | ACH97_210430 |
| glycosyltransferase family 1 protein | TRNA_RS34715 | BL14DL4_00885 | BLDA23_14265 | CK945_RS15025 | ACH97_210435 |
| hypothetical protein | TRNA_RS34720 | BL14DL4_00886 | BLDA23_14270 | CK945_RS15030 | ACH97_210440 |
| hypothetical protein | TRNA_RS34725 | BL14DL4_00887 | BLDA23_14275 | CK945_RS15035 | ACH97_210445 |
| hypothetical protein | TRNA_RS34730 | BL14DL4_00888 | BLDA23_14280 | CK945_RS15040 | ACH97_210450 |
| phosphate ABC transporter ATP-binding protein | TRNA_RS34735 | BL14DL4_00889 | BLDA23_14285 | CK945_RS15090 | ACH97_210455 |
| phosphate ABC transporter ATP-binding protein | TRNA_RS34740 | BL14DL4_00890 | BLDA23_14290 | CK945_RS15095 | ACH97_210460 |
| phosphate ABC transporter permease PstA | TRNA_RS34745 | BL14DL4_00891 | BLDA23_14295 | CK945_RS15100 | ACH97_210465 |
| phosphate ABC transporter permease subunit PstC | TRNA_RS34750 | BL14DL4_00892 | BLDA23_14300 | CK945_RS15105 | ACH97_210470 |
| phosphate ABC transporter substrate-binding protein | TRNA_RS34755 | BL14DL4_00893 | BLDA23_14305 | CK945_RS15110 | ACH97_210475 |
| hypothetical protein | TRNA_RS34760 | BL14DL4_00894 | BLDA23_14310 | CK945_RS15115 | ACH97_210480 |
| MFS transporter | TRNA_RS34765 | BL14DL4_00895 | BLDA23_14315 | CK945_RS15120 | ACH97_210485 |
| superoxide dismutase | TRNA_RS34770 | BL14DL4_00896 | BLDA23_14320 | CK945_RS15125 | ACH97_210490 |
| DUF456 domain-containing protein | TRNA_RS34775 | BL14DL4_00897 | BLDA23_14325 | CK945_RS15130 | ACH97_210495 |
| DUF1189 domain-containing protein | TRNA_RS34780 | BL14DL4_00898 | BLDA23_14330 | CK945_RS15135 | ACH97_210500 |
| LysM domain-containing protein | TRNA_RS34785 | BL14DL4_00899 | BLDA23_14335 | CK945_RS15140 | ACH97_210505 |
| flavodoxin-dependent (E)-4-hydroxy-3-methylbut-2-enyl-diphosphate synthase | TRNA_RS34790 | BL14DL4_00900 | BLDA23_14340 | CK945_RS15145 | ACH97_210510 |
| hypothetical protein | TRNA_RS34795 | BL14DL4_00901 | BLDA23_14345 | CK945_RS15150 | ACH97_210515 |
| nucleotidase | TRNA_RS34800 | BL14DL4_00902 | BLDA23_14350 | CK945_RS15155 | ACH97_210520 |
| transcriptional repressor | TRNA_RS34805 | BL14DL4_00903 | BLDA23_14355 | CK945_RS15160 | ACH97_210525 |
| metal ABC transporter permease | TRNA_RS34810 | BL14DL4_00904 | BLDA23_14360 | CK945_RS15165 | ACH97_210530 |
| ABC transporter ATP-binding protein | TRNA_RS34815 | BL14DL4_00905 | BLDA23_14365 | CK945_RS15170 | ACH97_210535 |
| YitT family protein | TRNA_RS34820 | BL14DL4_00906 | BLDA23_14370 | CK945_RS15175 | ACH97_210540 |
| DUF2624 domain-containing protein | TRNA_RS34825 | BL14DL4_00907 | BLDA23_14375 | CK945_RS15180 | ACH97_210545 |
| deoxyribonuclease IV | TRNA_RS34830 | BL14DL4_00908 | BLDA23_14380 | CK945_RS15185 | ACH97_210550 |
| DEAD/DEAH box helicase | TRNA_RS34835 | BL14DL4_00909 | BLDA23_14385 | CK945_RS15190 | ACH97_210555 |
| hypothetical protein | TRNA_RS34840 | BL14DL4_00910 | BLDA23_14390 | CK945_RS15195 | ACH97_210560 |
| 4-hydroxy-3-methylbut-2-enyl diphosphate reductase | TRNA_RS34845 | BL14DL4_00911 | BLDA23_14395 | CK945_RS15200 | ACH97_210565 |
| Nif3-like dinuclear metal center hexameric protein | TRNA_RS34850 | BL14DL4_00912 | BLDA23_14400 | CK945_RS15205 | ACH97_210570 |
| tRNA (adenine-N(1))-methyltransferase | TRNA_RS34855 | BL14DL4_00913 | BLDA23_14405 | CK945_RS15210 | ACH97_210575 |
| hypothetical protein | TRNA_RS34870 | BL14DL4_00915 | BLDA23_14420 | CK945_RS15220 | ACH97_210585 |
| hypothetical protein | TRNA_RS34875 | BL14DL4_00916 | BLDA23_14425 | CK945_RS15225 | ACH97_210590 |
| hypothetical protein | TRNA_RS34890 | BL14DL4_00919 | BLDA23_14440 | CK945_RS15230 | ACH97_210595 |
| transposase | TRNA_RS34910 | BL14DL4_00922 | BLDA23_14460 | CK945_RS15235 | ACH97_210600 |
| hypothetical protein | TRNA_RS34915 | BL14DL4_00923 | BLDA23_14465 | CK945_RS15240 | ACH97_210605 |
| DUF5082 domain-containing protein | TRNA_RS34920 | BL14DL4_00924 | BLDA23_14470 | CK945_RS15245 | ACH97_210610 |
| cytochrome c | TRNA_RS34925 | BL14DL4_00925 | BLDA23_14475 | CK945_RS15250 | ACH97_210615 |
| RNA polymerase sigma factor RpoD | TRNA_RS34930 | BL14DL4_00926 | BLDA23_14480 | CK945_RS15255 | ACH97_210620 |
| DNA primase | TRNA_RS34935 | BL14DL4_00927 | BLDA23_14485 | CK945_RS15260 | ACH97_210625 |
| YaiI/YqxD family protein | TRNA_RS34940 | BL14DL4_00928 | BLDA23_14490 | CK945_RS15265 | ACH97_210630 |
| phosphoenolpyruvate synthase regulatory protein | TRNA_RS34945 | BL14DL4_00929 | BLDA23_14495 | CK945_RS15270 | ACH97_210635 |
| transcriptional regulator | TRNA_RS34950 | BL14DL4_00930 | BLDA23_14500 | CK945_RS15275 | ACH97_210640 |
| glycine--tRNA ligase subunit beta | TRNA_RS34955 | BL14DL4_00931 | BLDA23_14505 | CK945_RS15280 | ACH97_210645 |
| glycine--tRNA ligase subunit alpha | TRNA_RS34960 | BL14DL4_00932 | BLDA23_14510 | CK945_RS15285 | ACH97_210650 |
| DNA repair protein RecO | TRNA_RS34965 | BL14DL4_00933 | BLDA23_14515 | CK945_RS15290 | ACH97_210655 |
| YqzL family protein | TRNA_RS43355 | BL14DL4_00934 | BLDA23_14520 | CK945_RS15295 | ACH97_210660 |
| GTPase Era | TRNA_RS34970 | BL14DL4_00935 | BLDA23_14525 | CK945_RS15300 | ACH97_210665 |
| cytidine deaminase | TRNA_RS34975 | BL14DL4_00936 | BLDA23_14530 | CK945_RS15305 | ACH97_210670 |
| diacylglycerol kinase family protein | TRNA_RS34980 | BL14DL4_00937 | BLDA23_14535 | CK945_RS15310 | ACH97_210675 |
| rRNA maturation RNase YbeY | TRNA_RS34985 | BL14DL4_00938 | BLDA23_14540 | CK945_RS15315 | ACH97_210680 |
| HDIG domain-containing protein | TRNA_RS34990 | BL14DL4_00939 | BLDA23_14545 | CK945_RS15320 | ACH97_210685 |
| PhoH family protein | TRNA_RS34995 | BL14DL4_00940 | BLDA23_14550 | CK945_RS15325 | ACH97_210690 |
| sporulation protein YqfD | TRNA_RS35000 | BL14DL4_00941 | BLDA23_14555 | CK945_RS15330 | ACH97_210695 |
| sporulation protein YqfC | TRNA_RS35005 | BL14DL4_00942 | BLDA23_14560 | CK945_RS15335 | ACH97_210700 |
| hypothetical protein | TRNA_RS35010 | BL14DL4_00943 | BLDA23_14565 | CK945_RS15340 | ACH97_210705 |
| UPF0365 family protein | TRNA_RS35015 | BL14DL4_00944 | BLDA23_14570 | CK945_RS15345 | ACH97_210710 |
| nodulation protein NfeD | TRNA_RS35020 | BL14DL4_00945 | BLDA23_14575 | CK945_RS15350 | ACH97_210715 |
| GatB/YqeY domain-containing protein | TRNA_RS35025 | BL14DL4_00946 | BLDA23_14580 | CK945_RS15355 | ACH97_219060 |
| 30S ribosomal protein S21 | TRNA_RS35030 | BL14DL4_00947 | BLDA23_14585 | CK945_RS15360 | ACH97_219065 |
| hypothetical protein | TRNA_RS35035 | BL14DL4_00948 | BLDA23_14590 | CK945_RS15365 | ACH97_219070 |
| deoxyribose-phosphate aldolase | TRNA_RS35040 | BL14DL4_00949 | BLDA23_14595 | CK945_RS15370 | ACH97_219075 |
| tRNA (N(6)-L-threonylcarbamoyladenosine(37)-C(2))- methylthiotransferase MtaB | TRNA_RS35045 | BL14DL4_00950 | BLDA23_14600 | CK945_RS15375 | ACH97_219080 |
| 16S rRNA (uracil(1498)-N(3))-methyltransferase | TRNA_RS35050 | BL14DL4_00951 | BLDA23_14605 | CK945_RS15380 | ACH97_219085 |
| 50S ribosomal protein L11 methyltransferase | TRNA_RS35055 | BL14DL4_00952 | BLDA23_14610 | CK945_RS15385 | ACH97_219090 |
| molecular chaperone DnaJ | TRNA_RS35060 | BL14DL4_00953 | BLDA23_14615 | CK945_RS15390 | ACH97_219095 |
| molecular chaperone DnaK | TRNA_RS35065 | BL14DL4_00954 | BLDA23_14620 | CK945_RS15395 | ACH97_219100 |
| nucleotide exchange factor GrpE | TRNA_RS35070 | BL14DL4_00955 | BLDA23_14625 | CK945_RS15400 | ACH97_219105 |
| HrcA family transcriptional regulator | TRNA_RS35075 | BL14DL4_00956 | BLDA23_14630 | CK945_RS15405 | ACH97_219110 |
| elongation factor 4 | TRNA_RS35085 | BL14DL4_00958 | BLDA23_14640 | CK945_RS15415 | ACH97_219120 |
| DUF3679 domain-containing protein | TRNA_RS35090 | BL14DL4_00959 | BLDA23_14645 | CK945_RS15420 | ACH97_219125 |
| stage II sporulation protein P | TRNA_RS35095 | BL14DL4_00960 | BLDA23_14650 | CK945_RS15425 | ACH97_219130 |
| GPR endopeptidase | TRNA_RS35100 | BL14DL4_00961 | BLDA23_14655 | CK945_RS15430 | ACH97_219135 |
| 30S ribosomal protein S20 | TRNA_RS35105 | BL14DL4_00962 | BLDA23_14660 | CK945_RS15435 | ACH97_219140 |
| DNA polymerase III subunit delta | TRNA_RS35110 | BL14DL4_00963 | BLDA23_14665 | CK945_RS15455 | ACH97_219165 |
| DNA internalization-related competence protein ComEC/Rec2 | TRNA_RS35120 | BL14DL4_00965 | BLDA23_14675 | CK945_RS15465 | ACH97_219170 |
| ComE operon protein 2 | TRNA_RS35125 | BL14DL4_00966 | BLDA23_14680 | CK945_RS15470 | ACH97_219175 |
| competence protein ComE | TRNA_RS35130 | BL14DL4_00967 | BLDA23_14685 | CK945_RS15475 | ACH97_219180 |
| late competence protein ComER | TRNA_RS35135 | BL14DL4_00968 | BLDA23_14690 | CK945_RS15480 | ACH97_219185 |
| class I SAM-dependent methyltransferase | TRNA_RS35140 | BL14DL4_00969 | BLDA23_14695 | CK945_RS15485 | ACH97_219190 |
| ribosome silencing factor | TRNA_RS35145 | BL14DL4_00970 | BLDA23_14700 | CK945_RS15490 | ACH97_219195 |
| HD domain-containing protein | TRNA_RS35150 | BL14DL4_00971 | BLDA23_14705 | CK945_RS15495 | ACH97_219200 |
| nicotinate-nucleotide adenylyltransferase | TRNA_RS35155 | BL14DL4_00972 | BLDA23_14710 | CK945_RS15500 | ACH97_219205 |
| ribosome assembly RNA-binding protein YhbY | TRNA_RS35160 | BL14DL4_00973 | BLDA23_14715 | CK945_RS15505 | ACH97_219210 |
| shikimate dehydrogenase | TRNA_RS35165 | BL14DL4_00974 | BLDA23_14720 | CK945_RS15510 | ACH97_219215 |
| ribosome biogenesis GTPase YqeH | TRNA_RS35170 | BL14DL4_00975 | BLDA23_14725 | CK945_RS15515 | ACH97_219220 |
| YqeG family HAD IIIA-type phosphatase | TRNA_RS35175 | BL14DL4_00976 | BLDA23_14730 | CK945_RS15520 | ACH97_219225 |
| SGNH/GDSL hydrolase family protein | TRNA_RS35185 | BL14DL4_00978 | BLDA23_14740 | CK945_RS15530 | ACH97_219235 |
| GNAT family N-acetyltransferase | TRNA_RS35190 | BL14DL4_00979 | BLDA23_14745 | CK945_RS15535 | ACH97_219240 |
| DUF2294 domain-containing protein | TRNA_RS35195 | BL14DL4_00980 | BLDA23_14750 | CK945_RS15540 | ACH97_219245 |
| formate dehydrogenase subunit alpha | TRNA_RS35200 | BL14DL4_00981 | BLDA23_14755 | CK945_RS15545 | ACH97_219250 |
| DUF1641 domain-containing protein | TRNA_RS35205 | BL14DL4_00982 | BLDA23_14760 | CK945_RS15550 | ACH97_219255 |
| RNA polymerase sporulation sigma factor SigK | TRNA_RS35215 | BL14DL4_00984 | BLDA23_14770 | CK945_RS15565 | ACH97_219265 |
| MBL fold metallo-hydrolase | TRNA_RS35285 | BL14DL4_00999 | BLDA23_14925 | CK945_RS15715 | ACH97_219400 |
| NAD(P)/FAD-dependent oxidoreductase | TRNA_RS35290 | BL14DL4_01000 | BLDA23_14930 | CK945_RS15720 | ACH97_219405 |
| DNA-binding response regulator | TRNA_RS35295 | BL14DL4_01001 | BLDA23_14935 | CK945_RS15725 | ACH97_219410 |
| PAS domain-containing sensor histidine kinase | TRNA_RS35300 | BL14DL4_01002 | BLDA23_14940 | CK945_RS15730 | ACH97_219415 |
| thioredoxin | TRNA_RS35305 | BL14DL4_01003 | BLDA23_14945 | CK945_RS15735 | ACH97_219420 |
| sulfite exporter TauE/SafE family protein | TRNA_RS35310 | BL14DL4_01004 | BLDA23_14950 | CK945_RS15740 | ACH97_219425 |
| hypothetical protein | TRNA_RS35315 | BL14DL4_01005 | BLDA23_14955 | CK945_RS15745 | ACH97_219430 |
| hypothetical protein | TRNA_RS35325 | BL14DL4_01007 | BLDA23_14965 | CK945_RS15755 | ACH97_219440 |
| hypothetical protein | TRNA_RS35330 | BL14DL4_01008 | BLDA23_14970 | CK945_RS15760 | ACH97_219445 |
| metal-sensitive transcriptional regulator | TRNA_RS35335 | BL14DL4_01009 | BLDA23_14975 | CK945_RS15765 | ACH97_219450 |
| methyltransferase domain-containing protein | TRNA_RS35340 | BL14DL4_01010 | BLDA23_14980 | CK945_RS15770 | ACH97_219455 |
| hypothetical protein | TRNA_RS35345 | BL14DL4_01011 | BLDA23_14985 | CK945_RS15775 | ACH97_219460 |
| SIS domain-containing protein | TRNA_RS35375 | BL14DL4_01015 | BLDA23_15030 | CK945_RS15800 | ACH97_219475 |
| 3-hexulose-6-phosphate synthase | TRNA_RS35380 | BL14DL4_01016 | BLDA23_15035 | CK945_RS15805 | ACH97_219480 |
| HxlR family transcriptional regulator | TRNA_RS35385 | BL14DL4_01017 | BLDA23_15040 | CK945_RS15810 | ACH97_219485 |
| fatty acid desaturase | TRNA_RS35390 | BL14DL4_01018 | BLDA23_15045 | CK945_RS15815 | ACH97_219490 |
| sensor histidine kinase | TRNA_RS35395 | BL14DL4_01019 | BLDA23_15050 | CK945_RS15820 | ACH97_219495 |
| DNA-binding response regulator | TRNA_RS35400 | BL14DL4_01020 | BLDA23_15055 | CK945_RS15825 | ACH97_219500 |
| NAD(P)/FAD-dependent oxidoreductase | TRNA_RS35405 | BL14DL4_01021 | BLDA23_15060 | CK945_RS15830 | ACH97_219505 |
| nickel ABC transporter, nickel/metallophore periplasmic binding protein | TRNA_RS35410 | BL14DL4_01022 | BLDA23_15065 | CK945_RS15835 | ACH97_219510 |
| nickel ABC transporter permease subunit NikB | TRNA_RS35415 | BL14DL4_01023 | BLDA23_15070 | CK945_RS15840 | ACH97_219515 |
| nickel ABC transporter permease subunit NikC | TRNA_RS35420 | BL14DL4_01024 | BLDA23_15075 | CK945_RS15845 | ACH97_219520 |
| nickel import ATP-binding protein NikD | TRNA_RS35425 | BL14DL4_01025 | BLDA23_15080 | CK945_RS15850 | ACH97_219525 |
| nickel import ATP-binding protein NikE | TRNA_RS35430 | BL14DL4_01026 | BLDA23_15085 | CK945_RS15855 | ACH97_219530 |
| MerR family transcriptional regulator | TRNA_RS35435 | BL14DL4_01027 | BLDA23_15090 | CK945_RS15860 | ACH97_219535 |
| MATE family efflux transporter | TRNA_RS35440 | BL14DL4_01028 | BLDA23_15095 | CK945_RS15865 | ACH97_219540 |
| SDR family oxidoreductase | TRNA_RS35450 | BL14DL4_01030 | BLDA23_15105 | CK945_RS15875 | ACH97_219550 |
| alpha/beta hydrolase | TRNA_RS35460 | BL14DL4_01032 | BLDA23_15115 | CK945_RS15885 | ACH97_219560 |
| cytochrome P450 | TRNA_RS35465 | BL14DL4_01033 | BLDA23_15120 | CK945_RS15890 | ACH97_219565 |
| hypothetical protein | TRNA_RS35480 | BL14DL4_01035 | BLDA23_15135 | CK945_RS15905 | ACH97_219585 |
| SDR family oxidoreductase | TRNA_RS35485 | BL14DL4_01036 | BLDA23_15140 | CK945_RS15910 | ACH97_219590 |
| glycoside hydrolase family 32 protein | TRNA_RS35490 | BL14DL4_01037 | BLDA23_15145 | CK945_RS15915 | ACH97_219595 |
| PTS mannose transporter subunit IID | TRNA_RS35495 | BL14DL4_01038 | BLDA23_15150 | CK945_RS15920 | ACH97_219600 |
| PTS mannose/fructose/sorbose transporter subunit IIC | TRNA_RS35500 | BL14DL4_01039 | BLDA23_15155 | CK945_RS15925 | ACH97_219605 |
| PTS fructose transporter subunit IIB | TRNA_RS35505 | BL14DL4_01040 | BLDA23_15160 | CK945_RS15930 | ACH97_219610 |
| PTS fructose transporter subunit IIA | TRNA_RS35510 | BL14DL4_01041 | BLDA23_15165 | CK945_RS15935 | ACH97_219615 |
| hypothetical protein | TRNA_RS35520 | BL14DL4_01043 | BLDA23_15175 | CK945_RS15945 | ACH97_219625 |
| hypothetical protein | TRNA_RS35525 | BL14DL4_01044 | BLDA23_15180 | CK945_RS15950 | ACH97_219630 |
| type A chloramphenicol O-acetyltransferase | TRNA_RS35530 | BL14DL4_01045 | BLDA23_15185 | CK945_RS15955 | ACH97_219635 |
| helix-turn-helix domain-containing protein | TRNA_RS35535 | BL14DL4_01046 | BLDA23_15190 | CK945_RS15960 | ACH97_219640 |
| methylated-DNA--[protein]-cysteine S-methyltransferase | TRNA_RS35540 | BL14DL4_01047 | BLDA23_15195 | CK945_RS15965 | ACH97_219645 |
| aldo/keto reductase | TRNA_RS35545 | BL14DL4_01048 | BLDA23_15200 | CK945_RS15995 | ACH97_219670 |
| MFS transporter | TRNA_RS35550 | BL14DL4_01049 | BLDA23_15205 | CK945_RS16000 | ACH97_219675 |
| transcriptional regulator | TRNA_RS35555 | BL14DL4_01050 | BLDA23_15210 | CK945_RS16005 | ACH97_219680 |
| peptidase E | TRNA_RS35560 | BL14DL4_01051 | BLDA23_15215 | CK945_RS16010 | ACH97_219685 |
| uroporphyrinogen-III synthase | TRNA_RS35565 | BL14DL4_01052 | BLDA23_15220 | CK945_RS16015 | ACH97_219695 |
| iron ABC transporter substrate-binding protein | TRNA_RS35575 | BL14DL4_01054 | BLDA23_15230 | CK945_RS16025 | ACH97_219705 |
| LysR family transcriptional regulator | TRNA_RS35580 | BL14DL4_01055 | BLDA23_15235 | CK945_RS16030 | ACH97_219710 |
| MFS transporter | TRNA_RS35585 | BL14DL4_01056 | BLDA23_15240 | CK945_RS16035 | ACH97_219715 |
| hypothetical protein | TRNA_RS35590 | BL14DL4_01057 | BLDA23_15245 | CK945_RS16040 | ACH97_219720 |
| cytochrome P450 | TRNA_RS35595 | BL14DL4_01058 | BLDA23_15250 | CK945_RS16045 | ACH97_219725 |
| TetR/AcrR family transcriptional regulator | TRNA_RS35600 | BL14DL4_01059 | BLDA23_15255 | CK945_RS16050 | ACH97_219730 |
| peptidase S8 | TRNA_RS35605 | BL14DL4_01060 | BLDA23_15260 | CK945_RS16055 | ACH97_219735 |
| class I SAM-dependent methyltransferase | TRNA_RS35610 | BL14DL4_01061 | BLDA23_15265 | CK945_RS16060 | ACH97_219740 |
| YjcZ family sporulation protein | TRNA_RS35615 | BL14DL4_01062 | BLDA23_15270 | CK945_RS16065 | ACH97_219745 |
| hypothetical protein | TRNA_RS35620 | BL14DL4_01064 | BLDA23_15285 | CK945_RS16075 | ACH97_219750 |
| bifunctional cystathionine gamma-lyase/homocysteine desulfhydrase | TRNA_RS35625 | BL14DL4_01065 | BLDA23_15290 | CK945_RS16080 | ACH97_219755 |
| cysteine synthase family protein | TRNA_RS35630 | BL14DL4_01066 | BLDA23_15295 | CK945_RS16085 | ACH97_219760 |
| 5-methylthioadenosine/S-adenosylhomocysteine nucleosidase | TRNA_RS35635 | BL14DL4_01067 | BLDA23_15300 | CK945_RS16090 | ACH97_219765 |
| SAM-dependent methyltransferase | TRNA_RS35640 | BL14DL4_01068 | BLDA23_15305 | CK945_RS16095 | ACH97_219770 |
| DUF2536 family protein | TRNA_RS35645 | BL14DL4_01069 | BLDA23_15310 | CK945_RS16100 | ACH97_219775 |
| DUF1510 family protein | TRNA_RS35650 | BL14DL4_01070 | BLDA23_15315 | CK945_RS16105 | ACH97_219780 |
| penicillin-binding protein 2 | TRNA_RS35655 | BL14DL4_01071 | BLDA23_15320 | CK945_RS16110 | ACH97_219785 |
| transcription elongation factor GreA | TRNA_RS35660 | BL14DL4_01072 | BLDA23_15325 | CK945_RS16115 | ACH97_219790 |
| uridine kinase | TRNA_RS35665 | BL14DL4_01073 | BLDA23_15330 | CK945_RS16120 | ACH97_219795 |
| collagenase-like protease | TRNA_RS35670 | BL14DL4_01074 | BLDA23_15335 | CK945_RS16125 | ACH97_219800 |
| U32 family peptidase | TRNA_RS35675 | BL14DL4_01075 | BLDA23_15340 | CK945_RS16130 | ACH97_219805 |
| O-methyltransferase | TRNA_RS35680 | BL14DL4_01076 | BLDA23_15345 | CK945_RS16135 | ACH97_219810 |
| endolytic transglycosylase MltG | TRNA_RS35685 | BL14DL4_01077 | BLDA23_15350 | CK945_RS16140 | ACH97_219815 |
| DUF1292 domain-containing protein | TRNA_RS35690 | BL14DL4_01078 | BLDA23_15355 | CK945_RS16145 | ACH97_219820 |
| IreB family regulatory phosphoprotein | TRNA_RS35700 | BL14DL4_01080 | BLDA23_15365 | CK945_RS16155 | ACH97_219830 |
| alanine--tRNA ligase | TRNA_RS35705 | BL14DL4_01081 | BLDA23_15370 | CK945_RS16160 | ACH97_219835 |
| AI-2E family transporter | TRNA_RS35710 | BL14DL4_01082 | BLDA23_15375 | CK945_RS16165 | ACH97_219840 |
| hypothetical protein | TRNA_RS35715 | BL14DL4_01083 | BLDA23_15385 | CK945_RS16175 | ACH97_219845 |
| photosynthetic reaction centre H-chain cytoplasmic | TRNA_RS35720 | BL14DL4_01084 | BLDA23_15390 | CK945_RS16180 | ACH97_219850 |
| ATP-dependent RecD-like DNA helicase | TRNA_RS35725 | BL14DL4_01085 | BLDA23_15395 | CK945_RS16185 | ACH97_219855 |
| tetratricopeptide repeat protein | TRNA_RS35730 | BL14DL4_01086 | BLDA23_15400 | CK945_RS16190 | ACH97_219860 |
| tRNA 2-thiouridine(34) synthase MnmA | TRNA_RS35735 | BL14DL4_01087 | BLDA23_15405 | CK945_RS16195 | ACH97_219865 |
| cysteine desulfurase | TRNA_RS35740 | BL14DL4_01088 | BLDA23_15410 | CK945_RS16200 | ACH97_219870 |
| Rrf2 family transcriptional regulator | TRNA_RS35745 | BL14DL4_01089 | BLDA23_15415 | CK945_RS16205 | ACH97_219875 |
| replication-associated recombination protein A | TRNA_RS35750 | BL14DL4_01090 | BLDA23_15420 | CK945_RS16210 | ACH97_219880 |
| tRNA threonylcarbamoyladenosine dehydratase | TRNA_RS35755 | BL14DL4_01091 | BLDA23_15425 | CK945_RS16215 | ACH97_219885 |
| aspartate--tRNA ligase | TRNA_RS35760 | BL14DL4_01092 | BLDA23_15435 | CK945_RS16225 | ACH97_219890 |
| histidine--tRNA ligase | TRNA_RS35765 | BL14DL4_01093 | BLDA23_15440 | CK945_RS16230 | ACH97_219895 |
| N-acetylmuramoyl-L-alanine amidase | TRNA_RS35770 | BL14DL4_01095 | BLDA23_15445 | CK945_RS16240 | ACH97_219900 |
| D-tyrosyl-tRNA(Tyr) deacylase | TRNA_RS35775 | BL14DL4_01096 | BLDA23_15450 | CK945_RS16245 | ACH97_219905 |
| bifunctional (p)ppGpp synthetase/guanosine-3,5-bis(diphosphate) 3-pyrophosphohydrolase | TRNA_RS35780 | BL14DL4_01097 | BLDA23_15455 | CK945_RS16250 | ACH97_219910 |
| adenine phosphoribosyltransferase | TRNA_RS35785 | BL14DL4_01098 | BLDA23_15460 | CK945_RS16255 | ACH97_219915 |
| single-stranded-DNA-specific exonuclease RecJ | TRNA_RS35790 | BL14DL4_01099 | BLDA23_15465 | CK945_RS16260 | ACH97_219920 |
| DUF1049 domain-containing protein | TRNA_RS35795 | BL14DL4_01100 | BLDA23_15470 | CK945_RS16265 | ACH97_219925 |
| protein translocase subunit SecDF | TRNA_RS35800 | BL14DL4_01101 | BLDA23_15475 | CK945_RS16275 | ACH97_219930 |
| hypothetical protein | TRNA_RS35805 | BL14DL4_01102 | BLDA23_15480 | CK945_RS16280 | ACH97_219935 |
| stage V sporulation protein B | TRNA_RS35810 | BL14DL4_01103 | BLDA23_15485 | CK945_RS16285 | ACH97_219940 |
| DUF421 domain-containing protein | TRNA_RS35815 | BL14DL4_01104 | BLDA23_15490 | CK945_RS16290 | ACH97_219945 |
| TIGR04086 family membrane protein | TRNA_RS35820 | BL14DL4_01105 | BLDA23_15495 | CK945_RS16295 | ACH97_219950 |
| preprotein translocase subunit YajC | TRNA_RS35825 | BL14DL4_01106 | BLDA23_15500 | CK945_RS16300 | ACH97_219955 |
| tRNA guanosine(34) transglycosylase Tgt | TRNA_RS35830 | BL14DL4_01107 | BLDA23_15505 | CK945_RS16305 | ACH97_219960 |
| tRNA preQ1(34) S-adenosylmethionine ribosyltransferase-isomerase QueA | TRNA_RS35835 | BL14DL4_01108 | BLDA23_15510 | CK945_RS16310 | ACH97_219965 |
| DUF2905 domain-containing protein | TRNA_RS35840 | BL14DL4_01109 | BLDA23_15515 | CK945_RS16315 | ACH97_219970 |
| Holliday junction branch migration DNA helicase RuvB | TRNA_RS35845 | BL14DL4_01110 | BLDA23_15520 | CK945_RS16320 | ACH97_219975 |
| Holliday junction branch migration protein RuvA | TRNA_RS35850 | BL14DL4_01111 | BLDA23_15525 | CK945_RS16325 | ACH97_219980 |
| intercompartmental signaling factor BofC | TRNA_RS35855 | BL14DL4_01112 | BLDA23_15530 | CK945_RS16330 | ACH97_219985 |
| serine/threonine protein kinase | TRNA_RS35860 | BL14DL4_01113 | BLDA23_15535 | CK945_RS16335 | ACH97_219990 |
| small, acid-soluble spore protein H | TRNA_RS35865 | BL14DL4_01115 | BLDA23_15540 | CK945_RS16360 | ACH97_219995 |
| DNA damage-inducible protein DinB | TRNA_RS35870 | BL14DL4_01116 | BLDA23_15545 | CK945_RS16365 | ACH97_220000 |
| hypothetical protein | TRNA_RS35875 | BL14DL4_01117 | BLDA23_15550 | CK945_RS16370 | ACH97_220005 |
| hypothetical protein | TRNA_RS35880 | BL14DL4_01118 | BLDA23_15555 | CK945_RS16375 | ACH97_220010 |
| YebC/PmpR family DNA-binding transcriptional regulator | TRNA_RS35890 | BL14DL4_01120 | BLDA23_15565 | CK945_RS16385 | ACH97_220020 |
| spore cortex protein | TRNA_RS35895 | BL14DL4_01121 | BLDA23_15575 | CK945_RS16390 | ACH97_220025 |
| LysM peptidoglycan-binding domain-containing protein | TRNA_RS35900 | BL14DL4_01122 | BLDA23_15580 | CK945_RS16395 | ACH97_220030 |
| quinolinate synthase NadA | TRNA_RS35905 | BL14DL4_01123 | BLDA23_15585 | CK945_RS16400 | ACH97_220035 |
| carboxylating nicotinate-nucleotide diphosphorylase | TRNA_RS35910 | BL14DL4_01124 | BLDA23_15590 | CK945_RS16405 | ACH97_220040 |
| L-aspartate oxidase | TRNA_RS35915 | BL14DL4_01125 | BLDA23_15595 | CK945_RS16410 | ACH97_220045 |
| aminotransferase class V-fold PLP-dependent enzyme | TRNA_RS35920 | BL14DL4_01126 | BLDA23_15600 | CK945_RS16415 | ACH97_220050 |
| transcription repressor NadR | TRNA_RS35925 | BL14DL4_01127 | BLDA23_15605 | CK945_RS16420 | ACH97_220055 |
| prephenate dehydratase | TRNA_RS35930 | BL14DL4_01128 | BLDA23_15610 | CK945_RS16425 | ACH97_220060 |
| hypothetical protein | TRNA_RS35935 | BL14DL4_01129 | BLDA23_15615 | CK945_RS16430 | ACH97_220065 |
| GTPase ObgE | TRNA_RS35940 | BL14DL4_01130 | BLDA23_15620 | CK945_RS16435 | ACH97_220070 |
| sporulation protein | TRNA_RS35945 | BL14DL4_01131 | BLDA23_15625 | CK945_RS16440 | ACH97_220075 |
| 50S ribosomal protein L27 | TRNA_RS35950 | BL14DL4_01132 | BLDA23_15630 | CK945_RS16445 | ACH97_220080 |
| ribosomal-processing cysteine protease Prp | TRNA_RS35955 | BL14DL4_01133 | BLDA23_15635 | CK945_RS16450 | ACH97_220085 |
| 50S ribosomal protein L21 | TRNA_RS35960 | BL14DL4_01134 | BLDA23_15640 | CK945_RS16455 | ACH97_220090 |
| stage IV sporulation protein FB | TRNA_RS35965 | BL14DL4_01135 | BLDA23_15645 | CK945_RS16460 | ACH97_220095 |
| M23 family peptidase | TRNA_RS35970 | BL14DL4_01136 | BLDA23_15650 | CK945_RS16465 | ACH97_220100 |
| SRPBCC domain-containing protein | TRNA_RS35975 | BL14DL4_01137 | BLDA23_15655 | CK945_RS16470 | ACH97_220105 |
| ArsR family transcriptional regulator | TRNA_RS35980 | BL14DL4_01138 | BLDA23_15660 | CK945_RS16475 | ACH97_220110 |
| septum site-determining protein MinD | TRNA_RS35985 | BL14DL4_01139 | BLDA23_15665 | CK945_RS16480 | ACH97_220115 |
| septum site-determining protein MinC | TRNA_RS35990 | BL14DL4_01140 | BLDA23_15670 | CK945_RS16485 | ACH97_220120 |
| rod shape-determining protein MreD | TRNA_RS35995 | BL14DL4_01141 | BLDA23_15675 | CK945_RS16490 | ACH97_220125 |
| rod shape-determining protein MreC | TRNA_RS36000 | BL14DL4_01142 | BLDA23_15680 | CK945_RS16495 | ACH97_220130 |
| rod shape-determining protein | TRNA_RS36005 | BL14DL4_01143 | BLDA23_15685 | CK945_RS16500 | ACH97_220135 |
| JAB domain-containing protein | TRNA_RS36010 | BL14DL4_01144 | BLDA23_15940 | CK945_RS16505 | ACH97_220140 |
| septum formation inhibitor Maf | TRNA_RS36015 | BL14DL4_01145 | BLDA23_15945 | CK945_RS16510 | ACH97_220145 |
| SPOR domain-containing protein | TRNA_RS36020 | BL14DL4_01146 | BLDA23_15950 | CK945_RS16515 | ACH97_220150 |
| bifunctional folylpolyglutamate synthase/dihydrofolate synthase | TRNA_RS36030 | BL14DL4_01148 | BLDA23_15960 | CK945_RS16525 | ACH97_220160 |
| valine--tRNA ligase | TRNA_RS36035 | BL14DL4_01149 | BLDA23_15965 | CK945_RS16530 | ACH97_220165 |
| hypothetical protein | TRNA_RS36040 | BL14DL4_01150 | BLDA23_15970 | CK945_RS16535 | ACH97_220170 |
| spore coat protein YsxE | TRNA_RS36045 | BL14DL4_01151 | BLDA23_15975 | CK945_RS16540 | ACH97_220175 |
| glutamate-1-semialdehyde 2,1-aminomutase | TRNA_RS36055 | BL14DL4_01153 | BLDA23_15985 | CK945_RS16550 | ACH97_217090 |
| porphobilinogen synthase | TRNA_RS36060 | BL14DL4_01154 | BLDA23_15990 | CK945_RS16555 | ACH97_217095 |
| uroporphyrinogen-III synthase | TRNA_RS36065 | BL14DL4_01155 | BLDA23_15995 | CK945_RS16560 | ACH97_217100 |
| hydroxymethylbilane synthase | TRNA_RS36070 | BL14DL4_01156 | BLDA23_16000 | CK945_RS16565 | ACH97_217105 |
| cytochrome c | TRNA_RS36075 | BL14DL4_01157 | BLDA23_16005 | CK945_RS16570 | ACH97_217110 |
| glutamyl-tRNA reductase | TRNA_RS36080 | BL14DL4_01158 | BLDA23_16010 | CK945_RS16575 | ACH97_217115 |
| hypothetical protein | TRNA_RS36085 | BL14DL4_01159 | BLDA23_16015 | CK945_RS16580 | ACH97_217120 |
| GTP-binding protein | TRNA_RS36090 | BL14DL4_01160 | BLDA23_16020 | CK945_RS16585 | ACH97_217125 |
| endopeptidase La | TRNA_RS36095 | BL14DL4_01161 | BLDA23_16025 | CK945_RS16590 | ACH97_217130 |
| ATP-dependent protease LonB | TRNA_RS36100 | BL14DL4_01162 | BLDA23_16030 | CK945_RS16595 | ACH97_217135 |
| ATP-dependent Clp protease ATP-binding subunit ClpX | TRNA_RS36105 | BL14DL4_01163 | BLDA23_16035 | CK945_RS16600 | ACH97_217140 |
| trigger factor | TRNA_RS36110 | BL14DL4_01164 | BLDA23_16040 | CK945_RS16605 | ACH97_217145 |
| tetratricopeptide repeat protein | TRNA_RS36115 | BL14DL4_01165 | BLDA23_16045 | CK945_RS16610 | ACH97_217150 |
| 3-isopropylmalate dehydratase small subunit | TRNA_RS36120 | BL14DL4_01166 | BLDA23_16050 | CK945_RS16615 | ACH97_217155 |
| 3-isopropylmalate dehydratase large subunit | TRNA_RS36125 | BL14DL4_01167 | BLDA23_16055 | CK945_RS16620 | ACH97_217160 |
| 3-isopropylmalate dehydrogenase | TRNA_RS36130 | BL14DL4_01168 | BLDA23_16060 | CK945_RS16625 | ACH97_217165 |
| 2-isopropylmalate synthase | TRNA_RS36135 | BL14DL4_01169 | BLDA23_16065 | CK945_RS16630 | ACH97_217170 |
| ketol-acid reductoisomerase | TRNA_RS36140 | BL14DL4_01170 | BLDA23_16070 | CK945_RS16635 | ACH97_217175 |
| acetolactate synthase small subunit | TRNA_RS36145 | BL14DL4_01171 | BLDA23_16075 | CK945_RS16640 | ACH97_217180 |
| acetolactate synthase large subunit | TRNA_RS36150 | BL14DL4_01172 | BLDA23_16080 | CK945_RS16645 | ACH97_217185 |
| branched-chain-amino-acid transaminase | TRNA_RS36155 | BL14DL4_01173 | BLDA23_16085 | CK945_RS16650 | ACH97_217190 |
| hypothetical protein | TRNA_RS36160 | BL14DL4_01174 | BLDA23_16090 | CK945_RS16655 | ACH97_217195 |
| NADP-specific glutamate dehydrogenase | TRNA_RS36165 | BL14DL4_01175 | BLDA23_16095 | CK945_RS16660 | ACH97_217200 |
| hypothetical protein | TRNA_RS36170 | BL14DL4_01176 | BLDA23_16100 | CK945_RS16665 | ACH97_217205 |
| hypothetical protein | TRNA_RS36175 | BL14DL4_01177 | BLDA23_16105 | CK945_RS16670 | ACH97_217210 |
| metallophosphoesterase | TRNA_RS36255 | BL14DL4_01193 | BLDA23_16180 | CK945_RS16680 | ACH97_217220 |
| non-canonical purine NTP pyrophosphatase | TRNA_RS36260 | BL14DL4_01194 | BLDA23_16185 | CK945_RS16685 | ACH97_217225 |
| ribonuclease PH | TRNA_RS36265 | BL14DL4_01195 | BLDA23_16190 | CK945_RS16690 | ACH97_217230 |
| sporulation protein | TRNA_RS36270 | BL14DL4_01196 | BLDA23_16195 | CK945_RS16695 | ACH97_217235 |
| glutamate racemase | TRNA_RS36275 | BL14DL4_01197 | BLDA23_16200 | CK945_RS16700 | ACH97_217240 |
| MarR family transcriptional regulator | TRNA_RS36280 | BL14DL4_01198 | BLDA23_16205 | CK945_RS16705 | ACH97_217245 |
| helix-turn-helix transcriptional regulator | TRNA_RS36285 | BL14DL4_01199 | BLDA23_16210 | CK945_RS16710 | ACH97_217250 |
| acyl-CoA thioesterase | TRNA_RS36295 | BL14DL4_01201 | BLDA23_16220 | CK945_RS16720 | ACH97_217260 |
| succinate dehydrogenase iron-sulfur subunit | TRNA_RS36300 | BL14DL4_01202 | BLDA23_16225 | CK945_RS16725 | ACH97_217265 |
| succinate dehydrogenase flavoprotein subunit | TRNA_RS36305 | BL14DL4_01203 | BLDA23_16230 | CK945_RS16730 | ACH97_217270 |
| succinate dehydrogenase cytochrome B558 | TRNA_RS36310 | BL14DL4_01204 | BLDA23_16235 | CK945_RS16735 | ACH97_217275 |
| DUF2507 domain-containing protein | TRNA_RS36315 | BL14DL4_01205 | BLDA23_16240 | CK945_RS16740 | ACH97_217280 |
| aspartate kinase | TRNA_RS36320 | BL14DL4_01206 | BLDA23_16245 | CK945_RS16745 | ACH97_217285 |
| excinuclease ABC subunit C | TRNA_RS36325 | BL14DL4_01207 | BLDA23_16255 | CK945_RS16750 | ACH97_217290 |
| thioredoxin | TRNA_RS36330 | BL14DL4_01208 | BLDA23_16260 | CK945_RS16755 | ACH97_217295 |
| electron transfer flavoprotein subunit alpha/FixB family protein | TRNA_RS36335 | BL14DL4_01209 | BLDA23_16265 | CK945_RS16765 | ACH97_217305 |
| electron transfer flavoprotein subunit beta/FixA family protein | TRNA_RS36340 | BL14DL4_01210 | BLDA23_16270 | CK945_RS16770 | ACH97_217310 |
| enoyl-CoA hydratase | TRNA_RS36345 | BL14DL4_01211 | BLDA23_16275 | CK945_RS16775 | ACH97_217315 |
| TetR family transcriptional regulator | TRNA_RS36350 | BL14DL4_01212 | BLDA23_16280 | CK945_RS16780 | ACH97_217320 |
| long-chain fatty acid--CoA ligase | TRNA_RS36355 | BL14DL4_01213 | BLDA23_16285 | CK945_RS16785 | ACH97_217325 |
| DUF350 domain-containing protein | TRNA_RS36360 | BL14DL4_01214 | BLDA23_16290 | CK945_RS16790 | ACH97_217330 |
| endonuclease MutS2 | TRNA_RS36365 | BL14DL4_01215 | BLDA23_16295 | CK945_RS16795 | ACH97_217335 |
| DNA polymerase/3-5 exonuclease PolX | TRNA_RS36370 | BL14DL4_01216 | BLDA23_16300 | CK945_RS16800 | ACH97_217340 |
| CvpA family protein | TRNA_RS36375 | BL14DL4_01217 | BLDA23_16305 | CK945_RS16805 | ACH97_217345 |
| cell division protein ZapA | TRNA_RS36380 | BL14DL4_01218 | BLDA23_16310 | CK945_RS16810 | ACH97_217350 |
| ribonuclease HIII | TRNA_RS36385 | BL14DL4_01219 | BLDA23_16315 | CK945_RS16815 | ACH97_217355 |
| phenylalanine--tRNA ligase subunit beta | TRNA_RS36425 | BL14DL4_01226 | BLDA23_16360 | CK945_RS16830 | ACH97_217370 |
| phenylalanine--tRNA ligase subunit alpha | TRNA_RS36430 | BL14DL4_01227 | BLDA23_16365 | CK945_RS16835 | ACH97_217375 |
| RNA methyltransferase | TRNA_RS36435 | BL14DL4_01228 | BLDA23_16370 | CK945_RS16840 | ACH97_217380 |
| small, acid-soluble spore protein I | TRNA_RS36440 | BL14DL4_01229 | BLDA23_16375 | CK945_RS16845 | ACH97_217385 |
| carbon starvation protein A | TRNA_RS36445 | BL14DL4_01230 | BLDA23_16380 | CK945_RS16850 | ACH97_217390 |
| carbohydrate ABC transporter permease | TRNA_RS36460 | BL14DL4_01233 | BLDA23_16395 | CK945_RS16880 | ACH97_217420 |
| sugar ABC transporter permease | TRNA_RS36465 | BL14DL4_01234 | BLDA23_16400 | CK945_RS16885 | ACH97_217425 |
| carbohydrate ABC transporter substrate-binding protein | TRNA_RS36470 | BL14DL4_01235 | BLDA23_16405 | CK945_RS16890 | ACH97_217430 |
| sn-glycerol-1-phosphate dehydrogenase | TRNA_RS36475 | BL14DL4_01236 | BLDA23_16410 | CK945_RS16895 | ACH97_217435 |
| L-ribulose-5-phosphate 4-epimerase | TRNA_RS36480 | BL14DL4_01237 | BLDA23_16415 | CK945_RS16900 | ACH97_217440 |
| ribulokinase | TRNA_RS36485 | BL14DL4_01238 | BLDA23_16420 | CK945_RS16905 | ACH97_217445 |
| L-arabinose isomerase | TRNA_RS36490 | BL14DL4_01239 | BLDA23_16425 | CK945_RS16910 | ACH97_217450 |
| M42 family peptidase | TRNA_RS36500 | BL14DL4_01241 | BLDA23_16435 | CK945_RS16920 | ACH97_217460 |
| hypothetical protein | TRNA_RS36505 | BL14DL4_01242 | BLDA23_16440 | CK945_RS16925 | ACH97_217465 |
| DUF1294 domain-containing protein | TRNA_RS36510 | BL14DL4_01243 | BLDA23_16445 | CK945_RS16930 | ACH97_217470 |
| 50S ribosomal protein L20 | TRNA_RS36515 | BL14DL4_01244 | BLDA23_16450 | CK945_RS16935 | ACH97_217475 |
| 50S ribosomal protein L35 | TRNA_RS36520 | BL14DL4_01245 | BLDA23_16455 | CK945_RS16940 | ACH97_217480 |
| translation initiation factor IF-3 | TRNA_RS36525 | BL14DL4_01246 | BLDA23_16460 | CK945_RS16945 | ACH97_217485 |
| antiholin-like protein LrgB | TRNA_RS36530 | BL14DL4_01247 | BLDA23_16465 | CK945_RS16950 | ACH97_217490 |
| antiholin-like protein LrgA | TRNA_RS36535 | BL14DL4_01248 | BLDA23_16470 | CK945_RS16955 | ACH97_217495 |
| alkaline phosphatase | TRNA_RS36540 | BL14DL4_01249 | BLDA23_16475 | CK945_RS16960 | ACH97_217500 |
| DNA-binding response regulator | TRNA_RS36545 | BL14DL4_01250 | BLDA23_16480 | CK945_RS16965 | ACH97_217505 |
| sensor histidine kinase | TRNA_RS36550 | BL14DL4_01251 | BLDA23_16485 | CK945_RS16970 | ACH97_217510 |
| DUF3923 family protein | TRNA_RS36555 | BL14DL4_01252 | BLDA23_16490 | CK945_RS16975 | ACH97_217515 |
| HAD family hydrolase | TRNA_RS36560 | BL14DL4_01253 | BLDA23_16495 | CK945_RS16980 | ACH97_217520 |
| threonine--tRNA ligase | TRNA_RS36565 | BL14DL4_01254 | BLDA23_16500 | CK945_RS16985 | ACH97_217525 |
| putative sporulation protein YtxC | TRNA_RS36570 | BL14DL4_01255 | BLDA23_16505 | CK945_RS16990 | ACH97_217530 |
| primosomal protein DnaI | TRNA_RS36580 | BL14DL4_01257 | BLDA23_16515 | CK945_RS17000 | ACH97_217540 |
| membrane attachment protein | TRNA_RS36585 | BL14DL4_01258 | BLDA23_16520 | CK945_RS17005 | ACH97_217545 |
| transcriptional regulator NrdR | TRNA_RS36590 | BL14DL4_01259 | BLDA23_16525 | CK945_RS17010 | ACH97_217550 |
| hypothetical protein | TRNA_RS36595 | BL14DL4_01260 | BLDA23_16530 | CK945_RS17015 | ACH97_217555 |
| S-adenosylmethionine decarboxylase proenzyme | TRNA_RS36605 | BL14DL4_01261 | BLDA23_16540 | CK945_RS17025 | ACH97_217565 |
| glyceraldehyde-3-phosphate dehydrogenase | TRNA_RS36610 | BL14DL4_01262 | BLDA23_16545 | CK945_RS17030 | ACH97_217570 |
| dephospho-CoA kinase | TRNA_RS36620 | BL14DL4_01264 | BLDA23_16555 | CK945_RS17040 | ACH97_217580 |
| sporulation membrane protein YtaF | TRNA_RS36625 | BL14DL4_01265 | BLDA23_16560 | CK945_RS17045 | ACH97_217585 |
| DNA-formamidopyrimidine glycosylase | TRNA_RS36630 | BL14DL4_01266 | BLDA23_16565 | CK945_RS17050 | ACH97_217590 |
| DNA polymerase I | TRNA_RS36635 | BL14DL4_01267 | BLDA23_16570 | CK945_RS17055 | ACH97_217595 |
| PAS domain-containing protein | TRNA_RS36640 | BL14DL4_01268 | BLDA23_16575 | CK945_RS17060 | ACH97_217600 |
| DNA-binding response regulator | TRNA_RS36645 | BL14DL4_01269 | BLDA23_16580 | CK945_RS17065 | ACH97_217605 |
| malate dehydrogenase | TRNA_RS36650 | BL14DL4_01270 | BLDA23_16585 | CK945_RS17070 | ACH97_217610 |
| NADP-dependent isocitrate dehydrogenase | TRNA_RS36655 | BL14DL4_01271 | BLDA23_16590 | CK945_RS17075 | ACH97_217615 |
| citrate synthase | TRNA_RS36660 | BL14DL4_01272 | BLDA23_16595 | CK945_RS17080 | ACH97_217620 |
| DUF441 domain-containing protein | TRNA_RS36665 | BL14DL4_01273 | BLDA23_16600 | CK945_RS17100 | ACH97_217640 |
| sporulation integral membrane protein YtvI | TRNA_RS36670 | BL14DL4_01274 | BLDA23_16605 | CK945_RS17105 | ACH97_217645 |
| membrane protein FxsA | TRNA_RS36675 | BL14DL4_01275 | BLDA23_16610 | CK945_RS17110 | ACH97_217650 |
| pyruvate kinase | TRNA_RS36680 | BL14DL4_01276 | BLDA23_16615 | CK945_RS17115 | ACH97_217655 |
| ATP-dependent 6-phosphofructokinase | TRNA_RS36685 | BL14DL4_01277 | BLDA23_16620 | CK945_RS17120 | ACH97_217660 |
| acetyl-CoA carboxylase carboxyl transferase subunit alpha | TRNA_RS36690 | BL14DL4_01278 | BLDA23_16625 | CK945_RS17125 | ACH97_217665 |
| acetyl-CoA carboxylase carboxyl transferase subunit beta | TRNA_RS36695 | BL14DL4_01279 | BLDA23_16630 | CK945_RS17130 | ACH97_217670 |
| NAD-dependent malic enzyme 4 | TRNA_RS36700 | BL14DL4_01280 | BLDA23_16635 | CK945_RS17135 | ACH97_217675 |
| sporulation protein | TRNA_RS36710 | BL14DL4_01282 | BLDA23_16645 | CK945_RS17145 | ACH97_217685 |
| hypothetical protein | TRNA_RS36715 | BL14DL4_01283 | BLDA23_16650 | CK945_RS17150 | ACH97_217690 |
| hypothetical protein | TRNA_RS36720 | BL14DL4_01284 | BLDA23_16655 | CK945_RS17155 | ACH97_217695 |
| bifunctional oligoribonuclease/PAP phosphatase NrnA | TRNA_RS36725 | BL14DL4_01285 | BLDA23_16660 | CK945_RS17160 | ACH97_217700 |
| membrane protein | TRNA_RS36730 | BL14DL4_01286 | BLDA23_16665 | CK945_RS17165 | ACH97_217705 |
| CBS domain-containing protein | TRNA_RS36735 | BL14DL4_01287 | BLDA23_16670 | CK945_RS17170 | ACH97_217710 |
| PadR family transcriptional regulator | TRNA_RS36740 | BL14DL4_01288 | BLDA23_16675 | CK945_RS17175 | ACH97_217715 |
| metal-dependent hydrolase | TRNA_RS36745 | BL14DL4_01289 | BLDA23_16680 | CK945_RS17180 | ACH97_217720 |
| SDR family oxidoreductase | TRNA_RS36750 | BL14DL4_01290 | BLDA23_16685 | CK945_RS17185 | ACH97_217725 |
| argininosuccinate lyase | TRNA_RS36755 | BL14DL4_01292 | BLDA23_16690 | CK945_RS17190 | ACH97_217730 |
| argininosuccinate synthase | TRNA_RS36760 | BL14DL4_01293 | BLDA23_16695 | CK945_RS17195 | ACH97_217735 |
| molybdenum cofactor biosynthesis protein MoaB | TRNA_RS36765 | BL14DL4_01294 | BLDA23_16700 | CK945_RS17200 | ACH97_217740 |
| acetate kinase | TRNA_RS36770 | BL14DL4_01295 | BLDA23_16705 | CK945_RS17205 | ACH97_221430 |
| thiol peroxidase | TRNA_RS36780 | BL14DL4_01297 | BLDA23_16715 | CK945_RS17215 | ACH97_221420 |
| sporulation protein YtfJ | TRNA_RS36785 | BL14DL4_01298 | BLDA23_16720 | CK945_RS17220 | ACH97_221415 |
| DUF2953 domain-containing protein | TRNA_RS36790 | BL14DL4_01299 | BLDA23_16725 | CK945_RS17225 | ACH97_221410 |
| RDD family protein | TRNA_RS36795 | BL14DL4_01300 | BLDA23_16730 | CK945_RS17230 | ACH97_221405 |
| signal peptide peptidase SppA | TRNA_RS36800 | BL14DL4_01301 | BLDA23_16735 | CK945_RS17235 | ACH97_221400 |
| NAD kinase | TRNA_RS36805 | BL14DL4_01302 | BLDA23_16740 | CK945_RS17240 | ACH97_221395 |
| MarR family transcriptional regulator | TRNA_RS36820 | BL14DL4_01305 | BLDA23_16755 | CK945_RS17245 | ACH97_221390 |
| acyl--CoA ligase | TRNA_RS36825 | BL14DL4_01306 | BLDA23_16760 | CK945_RS17250 | ACH97_221385 |
| small acid-soluble spore protein | TRNA_RS36830 | BL14DL4_01307 | BLDA23_16765 | CK945_RS17255 | ACH97_221380 |
| tRNA 4-thiouridine(8) synthase ThiI | TRNA_RS36835 | BL14DL4_01308 | BLDA23_16770 | CK945_RS17260 | ACH97_221375 |
| cysteine desulfurase | TRNA_RS36840 | BL14DL4_01309 | BLDA23_16775 | CK945_RS17265 | ACH97_221370 |
| branched-chain amino acid transport system II carrier protein | TRNA_RS36845 | BL14DL4_01310 | BLDA23_16780 | CK945_RS17270 | ACH97_221365 |
| septation ring formation regulator EzrA | TRNA_RS36860 | BL14DL4_01311 | BLDA23_16790 | CK945_RS17275 | ACH97_221360 |
| histidinol-phosphatase HisJ | TRNA_RS36865 | BL14DL4_01312 | BLDA23_16795 | CK945_RS17280 | ACH97_221355 |
| TetR/AcrR family transcriptional regulator | TRNA_RS36870 | BL14DL4_01313 | BLDA23_16800 | CK945_RS17285 | ACH97_221350 |
| GAF domain-containing protein | TRNA_RS36875 | BL14DL4_01314 | BLDA23_16805 | CK945_RS17290 | ACH97_221345 |
| 30S ribosomal protein S4 | TRNA_RS36885 | BL14DL4_01316 | BLDA23_16815 | CK945_RS17305 | ACH97_221335 |
| Tyrosine--tRNA ligase 1 | TRNA_RS36915 | BL14DL4_01323 | BLDA23_16845 | CK945_RS17320 | ACH97_221325 |
| acetate--CoA ligase | TRNA_RS36920 | BL14DL4_01324 | BLDA23_16850 | CK945_RS17325 | ACH97_221320 |
| GNAT family N-acetyltransferase | TRNA_RS36925 | BL14DL4_01325 | BLDA23_16855 | CK945_RS17330 | ACH97_221315 |
| CBS domain-containing protein | TRNA_RS36930 | BL14DL4_01326 | BLDA23_16860 | CK945_RS17335 | ACH97_221310 |
| acetoin utilization protein AcuC | TRNA_RS36935 | BL14DL4_01327 | BLDA23_16865 | CK945_RS17340 | ACH97_221305 |
| flagellar motor protein MotB | TRNA_RS36940 | BL14DL4_01328 | BLDA23_16870 | CK945_RS17345 | ACH97_221300 |
| flagellar motor protein MotP | TRNA_RS36945 | BL14DL4_01329 | BLDA23_16875 | CK945_RS17350 | ACH97_221295 |
| catabolite control protein A | TRNA_RS36950 | BL14DL4_01330 | BLDA23_16880 | CK945_RS17355 | ACH97_221290 |
| 3-deoxy-7-phosphoheptulonate synthase | TRNA_RS36955 | BL14DL4_01331 | BLDA23_16885 | CK945_RS17360 | ACH97_221285 |
| bacillithiol system redox-active protein YtxJ | TRNA_RS36965 | BL14DL4_01334 | BLDA23_16895 | CK945_RS17370 | ACH97_221275 |
| YtxH domain-containing protein | TRNA_RS36970 | BL14DL4_01335 | BLDA23_16900 | CK945_RS17375 | ACH97_221270 |
| DUF948 domain-containing protein | TRNA_RS36975 | BL14DL4_01336 | BLDA23_16905 | CK945_RS17380 | ACH97_221265 |
| UDP-N-acetylmuramate--L-alanine ligase | TRNA_RS36980 | BL14DL4_01337 | BLDA23_16910 | CK945_RS17385 | ACH97_221260 |
| DNA translocase FtsK | TRNA_RS36985 | BL14DL4_01338 | BLDA23_16915 | CK945_RS17390 | ACH97_221255 |
| DUF4479 domain-containing protein | TRNA_RS36990 | BL14DL4_01339 | BLDA23_16920 | CK945_RS17395 | ACH97_221250 |
| DUF1444 domain-containing protein | TRNA_RS36995 | BL14DL4_01340 | BLDA23_16925 | CK945_RS17400 | ACH97_221245 |
| thioredoxin | TRNA_RS37000 | BL14DL4_01341 | BLDA23_16930 | CK945_RS17405 | ACH97_221240 |
| YtoQ family protein | TRNA_RS37005 | BL14DL4_01342 | BLDA23_16935 | CK945_RS17410 | ACH97_221235 |
| M42 family peptidase | TRNA_RS37010 | BL14DL4_01343 | BLDA23_16940 | CK945_RS17415 | ACH97_221230 |
| hypothetical protein | TRNA_RS37015 | BL14DL4_01344 | BLDA23_16945 | CK945_RS17420 | ACH97_221225 |
| NAD-dependent malic enzyme | TRNA_RS37020 | BL14DL4_01345 | BLDA23_16950 | CK945_RS17425 | ACH97_221220 |
| MBL fold metallo-hydrolase | TRNA_RS37025 | BL14DL4_01346 | BLDA23_16955 | CK945_RS17430 | ACH97_221215 |
| tRNA (guanosine(46)-N7)-methyltransferase TrmB | TRNA_RS37030 | BL14DL4_01347 | BLDA23_16960 | CK945_RS17435 | ACH97_221210 |
| hypothetical protein | TRNA_RS37035 | BL14DL4_01348 | BLDA23_16965 | CK945_RS17440 | ACH97_221205 |
| phosphotransferase | TRNA_RS37040 | BL14DL4_01349 | BLDA23_16970 | CK945_RS17445 | ACH97_221200 |
| YegS/Rv2252/BmrU family lipid kinase | TRNA_RS37050 | BL14DL4_01351 | BLDA23_16980 | CK945_RS17455 | ACH97_221190 |
| hypothetical protein | TRNA_RS37055 | BL14DL4_01352 | BLDA23_16985 | CK945_RS17460 | ACH97_221185 |
| RNA 2,3-cyclic phosphodiesterase | TRNA_RS37060 | BL14DL4_01353 | BLDA23_16990 | CK945_RS17465 | ACH97_221180 |
| cysteine synthase A | TRNA_RS37065 | BL14DL4_01354 | BLDA23_16995 | CK945_RS17470 | ACH97_221175 |
| dipeptidase PepV | TRNA_RS37080 | BL14DL4_01356 | BLDA23_17010 | CK945_RS17485 | ACH97_221160 |
| NCS2 family permease | TRNA_RS37085 | BL14DL4_01357 | BLDA23_17015 | CK945_RS17490 | ACH97_221155 |
| DeoR family transcriptional regulator | TRNA_RS37090 | BL14DL4_01358 | BLDA23_17020 | CK945_RS17505 | ACH97_221140 |
| rRNA pseudouridine synthase | TRNA_RS37095 | BL14DL4_01359 | BLDA23_17025 | CK945_RS17510 | ACH97_221135 |
| polysaccharide biosynthesis protein | TRNA_RS37100 | BL14DL4_01360 | BLDA23_17030 | CK945_RS17515 | ACH97_221130 |
| NAD(P)/FAD-dependent oxidoreductase | TRNA_RS37105 | BL14DL4_01361 | BLDA23_17035 | CK945_RS17520 | ACH97_221125 |
| BCCT family transporter | TRNA_RS37110 | BL14DL4_01362 | BLDA23_17040 | CK945_RS17525 | ACH97_221120 |
| DUF2758 domain-containing protein | TRNA_RS37115 | BL14DL4_01363 | BLDA23_17045 | CK945_RS17530 | ACH97_221115 |
| rhodanese-like domain-containing protein | TRNA_RS37120 | BL14DL4_01364 | BLDA23_17050 | CK945_RS17535 | ACH97_221110 |
| DUF1992 domain-containing protein | TRNA_RS37130 | BL14DL4_01367 | BLDA23_17060 | CK945_RS17545 | ACH97_221100 |
| DUF624 domain-containing protein | TRNA_RS37135 | BL14DL4_01368 | BLDA23_17065 | CK945_RS17550 | ACH97_221095 |
| gfo/Idh/MocA family oxidoreductase | TRNA_RS37140 | BL14DL4_01369 | BLDA23_17070 | CK945_RS17555 | ACH97_221090 |
| hypothetical protein | TRNA_RS37145 | BL14DL4_01370 | BLDA23_17075 | CK945_RS17560 | ACH97_221085 |
| glycoside hydrolase family 105 protein | TRNA_RS37150 | BL14DL4_01371 | BLDA23_17080 | CK945_RS17565 | ACH97_221080 |
| sugar ABC transporter permease | TRNA_RS37155 | BL14DL4_01372 | BLDA23_17085 | CK945_RS17570 | ACH97_221075 |
| helix-turn-helix domain-containing protein | TRNA_RS37160 | BL14DL4_01373 | BLDA23_17090 | CK945_RS17575 | ACH97_221070 |
| extracellular solute-binding protein | TRNA_RS37165 | BL14DL4_01374 | BLDA23_17095 | CK945_RS17580 | ACH97_221065 |
| carbohydrate ABC transporter permease | TRNA_RS37170 | BL14DL4_01375 | BLDA23_17100 | CK945_RS17585 | ACH97_221060 |
| NAD(P)-dependent oxidoreductase | TRNA_RS37175 | BL14DL4_01376 | BLDA23_17105 | CK945_RS17590 | ACH97_221055 |
| hydrolase | TRNA_RS37180 | BL14DL4_01377 | BLDA23_17110 | CK945_RS17595 | ACH97_221050 |
| LysR family transcriptional regulator | TRNA_RS37190 | BL14DL4_01379 | BLDA23_17120 | CK945_RS17605 | ACH97_221040 |
| hypothetical protein | TRNA_RS37195 | BL14DL4_01380 | BLDA23_17125 | CK945_RS17610 | ACH97_221035 |
| leucine--tRNA ligase | TRNA_RS37200 | BL14DL4_01381 | BLDA23_17130 | CK945_RS17615 | ACH97_221030 |
| DUF4257 domain-containing protein | TRNA_RS37205 | BL14DL4_01382 | BLDA23_17135 | CK945_RS17620 | ACH97_221025 |
| MFS transporter | TRNA_RS37210 | BL14DL4_01383 | BLDA23_17140 | CK945_RS17625 | ACH97_221020 |
| hypothetical protein | TRNA_RS37215 | BL14DL4_01384 | BLDA23_17145 | CK945_RS17630 | ACH97_221015 |
| hypothetical protein | TRNA_RS37220 | BL14DL4_01385 | BLDA23_17150 | CK945_RS17635 | ACH97_221010 |
| ABC transporter permease | TRNA_RS37225 | BL14DL4_01386 | BLDA23_17155 | CK945_RS17640 | ACH97_221005 |
| ABC transporter ATP-binding protein | TRNA_RS37230 | BL14DL4_01387 | BLDA23_17160 | CK945_RS17645 | ACH97_221000 |
| permease | TRNA_RS37235 | BL14DL4_01388 | BLDA23_17165 | CK945_RS17650 | ACH97_220995 |
| ABC transporter ATP-binding protein | TRNA_RS37240 | BL14DL4_01389 | BLDA23_17170 | CK945_RS17655 | ACH97_220990 |
| GntR family transcriptional regulator | TRNA_RS37245 | BL14DL4_01390 | BLDA23_17175 | CK945_RS17660 | ACH97_220985 |
| DUF2524 family protein | TRNA_RS37250 | BL14DL4_01392 | BLDA23_17180 | CK945_RS17665 | ACH97_220980 |
| TIGR01212 family radical SAM protein | TRNA_RS37255 | BL14DL4_01393 | BLDA23_17185 | CK945_RS17670 | ACH97_220975 |
| methyltransferase domain-containing protein | TRNA_RS37260 | BL14DL4_01394 | BLDA23_17190 | CK945_RS17675 | ACH97_220970 |
| tetraprenyl-beta-curcumene synthase family protein | TRNA_RS37270 | BL14DL4_01396 | BLDA23_17200 | CK945_RS17685 | ACH97_220965 |
| alpha/beta hydrolase | TRNA_RS37275 | BL14DL4_01397 | BLDA23_17205 | CK945_RS17690 | ACH97_220960 |
| gamma carbonic anhydrase family protein | TRNA_RS37280 | BL14DL4_01398 | BLDA23_17210 | CK945_RS17695 | ACH97_220955 |
| phosphatase PAP2 family protein | TRNA_RS37285 | BL14DL4_01399 | BLDA23_17215 | CK945_RS17700 | ACH97_220950 |
| glycosyltransferase family 1 protein | TRNA_RS37290 | BL14DL4_01400 | BLDA23_17220 | CK945_RS17705 | ACH97_220945 |
| asparagine synthase (glutamine-hydrolyzing) | TRNA_RS37295 | BL14DL4_01401 | BLDA23_17225 | CK945_RS17710 | ACH97_220940 |
| S-adenosylmethionine synthase | TRNA_RS37300 | BL14DL4_01402 | BLDA23_17230 | CK945_RS17715 | ACH97_220935 |
| phosphoenolpyruvate carboxykinase (ATP) | TRNA_RS37305 | BL14DL4_01403 | BLDA23_17235 | CK945_RS17720 | ACH97_220930 |
| dicarboxylate/amino acid:cation symporter | TRNA_RS37310 | BL14DL4_01404 | BLDA23_17240 | CK945_RS17725 | ACH97_220925 |
| DUF2584 domain-containing protein | TRNA_RS37315 | BL14DL4_01405 | BLDA23_17245 | CK945_RS17730 | ACH97_220920 |
| S9 family peptidase | TRNA_RS37320 | BL14DL4_01406 | BLDA23_17250 | CK945_RS17735 | ACH97_220915 |
| ABC transporter ATP-binding protein | TRNA_RS37330 | BL14DL4_01408 | BLDA23_17260 | CK945_RS17745 | ACH97_220905 |
| ABC transporter permease | TRNA_RS37335 | BL14DL4_01409 | BLDA23_17265 | CK945_RS17750 | ACH97_220900 |
| nucleoside triphosphatase YtkD | TRNA_RS37340 | BL14DL4_01410 | BLDA23_17270 | CK945_RS17755 | ACH97_220895 |
| hypothetical protein | TRNA_RS37345 | BL14DL4_01411 | BLDA23_17275 | CK945_RS17760 | ACH97_220890 |
| hypothetical protein | TRNA_RS37350 | BL14DL4_01412 | BLDA23_17280 | CK945_RS17765 | ACH97_220885 |
| DNA starvation/stationary phase protection protein | TRNA_RS37355 | BL14DL4_01413 | BLDA23_17285 | CK945_RS17770 | ACH97_220880 |
| hypothetical protein | TRNA_RS37360 | BL14DL4_01415 | BLDA23_17295 | CK945_RS17780 | ACH97_220875 |
| S-ribosylhomocysteine lyase | TRNA_RS37365 | BL14DL4_01416 | BLDA23_17300 | CK945_RS17785 | ACH97_220870 |
| membrane protein insertion efficiency factor YidD | TRNA_RS37370 | BL14DL4_01417 | BLDA23_17305 | CK945_RS17790 | ACH97_220865 |
| carbonic anhydrase | TRNA_RS37375 | BL14DL4_01418 | BLDA23_17310 | CK945_RS17795 | ACH97_220860 |
| GTP-binding protein | TRNA_RS37380 | BL14DL4_01419 | BLDA23_17315 | CK945_RS17800 | ACH97_220855 |
| adhesin | TRNA_RS37385 | BL14DL4_01420 | BLDA23_17320 | CK945_RS17805 | ACH97_220850 |
| 50S ribosomal protein L31 type B | TRNA_RS37390 | BL14DL4_01421 | BLDA23_17325 | CK945_RS17810 | ACH97_220845 |
| cytochrome ubiquinol oxidase subunit I | TRNA_RS37395 | BL14DL4_01422 | BLDA23_17330 | CK945_RS17815 | ACH97_220840 |
| membrane protein | TRNA_RS37400 | BL14DL4_01423 | BLDA23_17335 | CK945_RS17820 | ACH97_220835 |
| hypothetical protein | TRNA_RS37405 | BL14DL4_01425 | BLDA23_17345 | CK945_RS17830 | ACH97_220825 |
| 2-succinylbenzoate-CoA ligase | TRNA_RS37415 | BL14DL4_01427 | BLDA23_17355 | CK945_RS17840 | ACH97_220815 |
| 1,4-dihydroxy-2-naphthoyl-CoA synthase | TRNA_RS37420 | BL14DL4_01428 | BLDA23_17360 | CK945_RS17845 | ACH97_220810 |
| 2-succinyl-6-hydroxy-2, 4-cyclohexadiene-1-carboxylate synthase | TRNA_RS37425 | BL14DL4_01429 | BLDA23_17365 | CK945_RS17850 | ACH97_220805 |
| 2-succinyl-5-enolpyruvyl-6-hydroxy-3- cyclohexene-1-carboxylate synthase | TRNA_RS37430 | BL14DL4_01430 | BLDA23_17370 | CK945_RS17855 | ACH97_220800 |
| isochorismate synthase | TRNA_RS37435 | BL14DL4_01431 | BLDA23_17375 | CK945_RS17860 | ACH97_220795 |
| 1,4-dihydroxy-2-naphthoate polyprenyltransferase | TRNA_RS37440 | BL14DL4_01432 | BLDA23_17380 | CK945_RS17865 | ACH97_220790 |
| hypothetical protein | TRNA_RS37445 | BL14DL4_01433 | BLDA23_17385 | CK945_RS17870 | ACH97_220785 |
| glycogen synthase GlgA | TRNA_RS37455 | BL14DL4_01435 | BLDA23_17395 | CK945_RS17880 | ACH97_220775 |
| glucose-1-phosphate adenylyltransferase | TRNA_RS37460 | BL14DL4_01436 | BLDA23_17400 | CK945_RS17885 | ACH97_220770 |
| glycogen-branching enzyme | TRNA_RS37470 | BL14DL4_01438 | BLDA23_17410 | CK945_RS17895 | ACH97_220760 |
| GntR family transcriptional regulator | TRNA_RS37595 | BL14DL4_01463 | BLDA23_17535 | CK945_RS18020 | ACH97_202255 |
| energy-coupled thiamine transporter ThiT | TRNA_RS37600 | BL14DL4_01464 | BLDA23_17540 | CK945_RS18025 | ACH97_202250 |
| class D sortase | TRNA_RS37605 | BL14DL4_01465 | BLDA23_17545 | CK945_RS18030 | ACH97_202245 |
| processed acidic surface protein | TRNA_RS37610 | BL14DL4_01466 | BLDA23_17550 | CK945_RS18035 | ACH97_202240 |
| hypothetical protein | TRNA_RS37615 | BL14DL4_01467 | BLDA23_17555 | CK945_RS18040 | ACH97_202235 |
| hypothetical protein | TRNA_RS37620 | BL14DL4_01468 | BLDA23_17560 | CK945_RS18045 | ACH97_202230 |
| pyroglutamyl-peptidase I | TRNA_RS37625 | BL14DL4_01470 | BLDA23_17565 | CK945_RS18050 | ACH97_202225 |
| DinB family protein | TRNA_RS37645 | BL14DL4_01474 | BLDA23_17585 | CK945_RS18070 | ACH97_202205 |
| molybdenum cofactor sulfurase | TRNA_RS37650 | BL14DL4_01475 | BLDA23_17590 | CK945_RS18075 | ACH97_202200 |
| iron-containing alcohol dehydrogenase | TRNA_RS37655 | BL14DL4_01476 | BLDA23_17595 | CK945_RS18080 | ACH97_202195 |
| betaine-aldehyde dehydrogenase | TRNA_RS37660 | BL14DL4_01477 | BLDA23_17600 | CK945_RS18085 | ACH97_202190 |
| GbsR/MarR family transcriptional regulator | TRNA_RS37665 | BL14DL4_01478 | BLDA23_17605 | CK945_RS18090 | ACH97_202185 |
| sodium:solute symporter | TRNA_RS37670 | BL14DL4_01479 | BLDA23_17610 | CK945_RS18095 | ACH97_202180 |
| SAM-dependent methyltransferase | TRNA_RS37675 | BL14DL4_01480 | BLDA23_17615 | CK945_RS18100 | ACH97_202175 |
| alanine racemase | TRNA_RS37680 | BL14DL4_01481 | BLDA23_17620 | CK945_RS18105 | ACH97_202170 |
| hypothetical protein | TRNA_RS37705 | BL14DL4_01491 | BLDA23_17680 | CK945_RS18155 | ACH97_202085 |
| DUF5081 family protein | TRNA_RS37710 | BL14DL4_01492 | BLDA23_17685 | CK945_RS18160 | ACH97_202080 |
| WXG100 family type VII secretion target | TRNA_RS37715 | BL14DL4_01493 | BLDA23_17690 | CK945_RS18165 | ACH97_202075 |
| hypothetical protein | TRNA_RS37720 | BL14DL4_01494 | BLDA23_17695 | CK945_RS18170 | ACH97_202070 |
| hypothetical protein | TRNA_RS37725 | BL14DL4_01495 | BLDA23_17700 | CK945_RS18175 | ACH97_202065 |
| phosphodiesterase | TRNA_RS37730 | BL14DL4_01497 | BLDA23_17705 | CK945_RS18180 | ACH97_202060 |
| glycosyl transferase | TRNA_RS37735 | BL14DL4_01498 | BLDA23_17710 | CK945_RS18185 | ACH97_202055 |
| hypothetical protein | TRNA_RS37740 | BL14DL4_01499 | BLDA23_17715 | CK945_RS18190 | ACH97_202050 |
| metallophosphoesterase | TRNA_RS37745 | BL14DL4_01500 | BLDA23_17720 | CK945_RS18195 | ACH97_202045 |
| undecaprenyl-diphosphate phosphatase | TRNA_RS37750 | BL14DL4_01501 | BLDA23_17725 | CK945_RS18200 | ACH97_202040 |
| AI-2E family transporter | TRNA_RS37755 | BL14DL4_01502 | BLDA23_17730 | CK945_RS18205 | ACH97_202035 |
| gfo/Idh/MocA family oxidoreductase | TRNA_RS37760 | BL14DL4_01503 | BLDA23_17735 | CK945_RS18210 | ACH97_202030 |
| HAMP domain-containing protein | TRNA_RS37770 | BL14DL4_01505 | BLDA23_17745 | CK945_RS18220 | ACH97_202020 |
| hypothetical protein | TRNA_RS37795 | BL14DL4_01510 | BLDA23_17770 | CK945_RS18235 | ACH97_201995 |
| alpha-glucosidase | TRNA_RS37800 | BL14DL4_01511 | BLDA23_17775 | CK945_RS18240 | ACH97_201990 |
| TetR/AcrR family transcriptional regulator | TRNA_RS37805 | BL14DL4_01512 | BLDA23_17780 | CK945_RS18245 | ACH97_201985 |
| hypothetical protein | TRNA_RS37810 | BL14DL4_01513 | BLDA23_17785 | CK945_RS18250 | ACH97_201980 |
| glycoside hydrolase | TRNA_RS37815 | BL14DL4_01514 | BLDA23_17790 | CK945_RS18255 | ACH97_201975 |
| HlyC/CorC family transporter | TRNA_RS37820 | BL14DL4_01515 | BLDA23_17795 | CK945_RS18260 | ACH97_201970 |
| peptidase | TRNA_RS37825 | BL14DL4_01516 | BLDA23_17800 | CK945_RS18265 | ACH97_201965 |
| protein mistic | TRNA_RS37830 | BL14DL4_01518 | BLDA23_17810 | CK945_RS18275 | ACH97_201955 |
| potassium channel protein | TRNA_RS37835 | BL14DL4_01519 | BLDA23_17815 | CK945_RS18280 | ACH97_201950 |
| hypothetical protein | TRNA_RS37840 | BL14DL4_01520 | BLDA23_17820 | CK945_RS18285 | ACH97_201945 |
| tryptophan-rich sensory protein | TRNA_RS37850 | BL14DL4_01522 | BLDA23_17830 | CK945_RS18295 | ACH97_201935 |
| N-acetyltransferase | TRNA_RS37855 | BL14DL4_01523 | BLDA23_17835 | CK945_RS18300 | ACH97_201930 |
| glucose-6-phosphate isomerase | TRNA_RS37860 | BL14DL4_01575 | BLDA23_18110 | CK945_RS18305 | ACH97_201925 |
| iron-containing alcohol dehydrogenase | TRNA_RS37865 | BL14DL4_01576 | BLDA23_18115 | CK945_RS18310 | ACH97_201920 |
| DUF378 domain-containing protein | TRNA_RS37870 | BL14DL4_01577 | BLDA23_18120 | CK945_RS18315 | ACH97_201915 |
| general stress protein 13 | TRNA_RS37875 | BL14DL4_01578 | BLDA23_18125 | CK945_RS18320 | ACH97_201910 |
| aminotransferase | TRNA_RS37880 | BL14DL4_01579 | BLDA23_18130 | CK945_RS18325 | ACH97_201905 |
| Lrp/AsnC family transcriptional regulator | TRNA_RS37885 | BL14DL4_01580 | BLDA23_18135 | CK945_RS18330 | ACH97_201900 |
| DUF1871 family protein | TRNA_RS37895 | BL14DL4_01582 | BLDA23_18145 | CK945_RS18340 | ACH97_201890 |
| hypothetical protein | TRNA_RS37900 | BL14DL4_01583 | BLDA23_18150 | CK945_RS18345 | ACH97_201885 |
| pyridoxal phosphate-dependent aminotransferase | TRNA_RS37905 | BL14DL4_01584 | BLDA23_18155 | CK945_RS18350 | ACH97_201880 |
| hypothetical protein | TRNA_RS37910 | BL14DL4_01585 | BLDA23_18160 | CK945_RS18355 | ACH97_201875 |
| kinase | TRNA_RS37915 | BL14DL4_01586 | BLDA23_18165 | CK945_RS18360 | ACH97_201870 |
| putative 3-5 exonuclease KapD | TRNA_RS37920 | BL14DL4_01587 | BLDA23_18170 | CK945_RS18365 | ACH97_201865 |
| MFS transporter | TRNA_RS37925 | BL14DL4_01588 | BLDA23_18175 | CK945_RS18370 | ACH97_201860 |
| penicillin-binding protein | TRNA_RS37930 | BL14DL4_01589 | BLDA23_18180 | CK945_RS18375 | ACH97_201855 |
| thiol-disulfide oxidoreductase DCC family protein | TRNA_RS37935 | BL14DL4_01590 | BLDA23_18185 | CK945_RS18380 | ACH97_201850 |
| hypothetical protein | TRNA_RS37940 | BL14DL4_01591 | BLDA23_18190 | CK945_RS18385 | ACH97_201845 |
| two-component system sensor histidine kinase DcuS | TRNA_RS37945 | BL14DL4_01592 | BLDA23_18195 | CK945_RS18390 | ACH97_201840 |
| two-component system response regulator DcuR | TRNA_RS37950 | BL14DL4_01593 | BLDA23_18200 | CK945_RS18395 | ACH97_201835 |
| CDP-diacylglycerol--serine O-phosphatidyltransferase | TRNA_RS37955 | BL14DL4_01594 | BLDA23_18210 | CK945_RS18400 | ACH97_201830 |
| DedA family protein | TRNA_RS37960 | BL14DL4_01595 | BLDA23_18215 | CK945_RS18405 | ACH97_201825 |
| phosphatidylserine decarboxylase | TRNA_RS37965 | BL14DL4_01596 | BLDA23_18220 | CK945_RS18410 | ACH97_201820 |
| L-malate permease | TRNA_RS37980 | BL14DL4_01597 | BLDA23_18225 | CK945_RS18415 | ACH97_201815 |
| hypothetical protein | TRNA_RS37985 | BL14DL4_01598 | BLDA23_18230 | CK945_RS18420 | ACH97_201810 |
| Na+/H+ antiporter subunit A | TRNA_RS37990 | BL14DL4_01599 | BLDA23_18235 | CK945_RS18425 | ACH97_201805 |
| Na(+)/H(+) antiporter subunit B | TRNA_RS37995 | BL14DL4_01600 | BLDA23_18240 | CK945_RS18430 | ACH97_201800 |
| Na(+)/H(+) antiporter subunit C | TRNA_RS38000 | BL14DL4_01601 | BLDA23_18245 | CK945_RS18435 | ACH97_201795 |
| Na+/H+ antiporter subunit D | TRNA_RS38005 | BL14DL4_01602 | BLDA23_18250 | CK945_RS18440 | ACH97_201790 |
| Na+/H+ antiporter subunit E | TRNA_RS38010 | BL14DL4_01603 | BLDA23_18255 | CK945_RS18445 | ACH97_201785 |
| Na(+)/H(+) antiporter subunit F1 | TRNA_RS38015 | BL14DL4_01604 | BLDA23_18260 | CK945_RS18450 | ACH97_201780 |
| Na+/H+ antiporter subunit G | TRNA_RS38020 | BL14DL4_01605 | BLDA23_18265 | CK945_RS18455 | ACH97_201775 |
| hotdog fold thioesterase | TRNA_RS38025 | BL14DL4_01606 | BLDA23_18270 | CK945_RS18460 | ACH97_201770 |
| DNA-binding response regulator | TRNA_RS38030 | BL14DL4_01607 | BLDA23_18275 | CK945_RS18465 | ACH97_201765 |
| competence pheromone ComX | TRNA_RS38055 | BL14DL4_01609 | BLDA23_18285 | CK945_RS18475 | ACH97_201755 |
| isoprenyl transferase | TRNA_RS38060 | BL14DL4_01610 | BLDA23_18290 | CK945_RS18480 | ACH97_201750 |
| degradation enzyme regulation protein DegQ | TRNA_RS38065 | BL14DL4_01611 | BLDA23_18295 | CK945_RS18485 | ACH97_201745 |
| hypothetical protein | TRNA_RS38075 | BL14DL4_01613 | BLDA23_18305 | CK945_RS18495 | ACH97_201740 |
| HDOD domain-containing protein | TRNA_RS38080 | BL14DL4_01614 | BLDA23_18310 | CK945_RS18500 | ACH97_201735 |
| nicotinate phosphoribosyltransferase | TRNA_RS38085 | BL14DL4_01615 | BLDA23_18315 | CK945_RS18505 | ACH97_201730 |
| cysteine hydrolase | TRNA_RS38090 | BL14DL4_01616 | BLDA23_18320 | CK945_RS18510 | ACH97_201725 |
| DUF1694 domain-containing protein | TRNA_RS38095 | BL14DL4_01617 | BLDA23_18325 | CK945_RS18515 | ACH97_201720 |
| hypothetical protein | TRNA_RS38100 | BL14DL4_01618 | BLDA23_18330 | CK945_RS18520 | ACH97_201715 |
| AI-2E family transporter | TRNA_RS38110 | BL14DL4_01620 | BLDA23_18340 | CK945_RS18530 | ACH97_201705 |
| hypothetical protein | TRNA_RS38125 | BL14DL4_01623 | BLDA23_18355 | CK945_RS18535 | ACH97_201690 |
| HD domain-containing protein | TRNA_RS38130 | BL14DL4_01624 | BLDA23_18360 | CK945_RS18540 | ACH97_201685 |
| (S)-benzoin forming benzil reductase | TRNA_RS38135 | BL14DL4_01625 | BLDA23_18365 | CK945_RS18545 | ACH97_201680 |
| type VII secretion protein EssA | TRNA_RS38140 | BL14DL4_01626 | BLDA23_18370 | CK945_RS18555 | ACH97_201670 |
| type VII secretion protein EsaA | TRNA_RS38145 | BL14DL4_01627 | BLDA23_18375 | CK945_RS18560 | ACH97_201665 |
| type VII secretion protein EssC | TRNA_RS38150 | BL14DL4_01628 | BLDA23_18380 | CK945_RS18565 | ACH97_201660 |
| type VII secretion protein EssB | TRNA_RS38155 | BL14DL4_01629 | BLDA23_18385 | CK945_RS18570 | ACH97_201655 |
| hypothetical protein | TRNA_RS38160 | BL14DL4_01630 | BLDA23_18390 | CK945_RS18575 | ACH97_201650 |
| WXG100 family type VII secretion target | TRNA_RS38165 | BL14DL4_01631 | BLDA23_18395 | CK945_RS18580 | ACH97_201645 |
| sodium:alanine symporter family protein | TRNA_RS38170 | BL14DL4_01632 | BLDA23_18400 | CK945_RS18585 | ACH97_201640 |
| PucR family transcriptional regulator | TRNA_RS38175 | BL14DL4_01633 | BLDA23_18405 | CK945_RS18590 | ACH97_201635 |
| alanine dehydrogenase | TRNA_RS38180 | BL14DL4_01634 | BLDA23_18410 | CK945_RS18595 | ACH97_201630 |
| sulfite oxidase-like oxidoreductase | TRNA_RS38190 | BL14DL4_01636 | BLDA23_18420 | CK945_RS18605 | ACH97_201620 |
| biotin transporter BioY | TRNA_RS38195 | BL14DL4_01637 | BLDA23_18425 | CK945_RS18610 | ACH97_201615 |
| sodium:proton antiporter | TRNA_RS38200 | BL14DL4_01638 | BLDA23_18430 | CK945_RS18615 | ACH97_201610 |
| leucyl aminopeptidase | TRNA_RS38205 | BL14DL4_01639 | BLDA23_18435 | CK945_RS18620 | ACH97_201605 |
| divergent PAP2 family protein | TRNA_RS38210 | BL14DL4_01640 | BLDA23_18440 | CK945_RS18625 | ACH97_201600 |
| hypothetical protein | TRNA_RS38215 | BL14DL4_01641 | BLDA23_18445 | CK945_RS18630 | ACH97_201595 |
| membrane protein | TRNA_RS38220 | BL14DL4_01642 | BLDA23_18450 | CK945_RS18635 | ACH97_201590 |
| NAD(P)/FAD-dependent oxidoreductase | TRNA_RS38235 | BL14DL4_01644 | BLDA23_18460 | CK945_RS18645 | ACH97_201575 |
| hypothetical protein | TRNA_RS38240 | BL14DL4_01645 | BLDA23_18465 | CK945_RS18650 | ACH97_201570 |
| GNAT family N-acetyltransferase | TRNA_RS38250 | BL14DL4_01647 | BLDA23_18475 | CK945_RS18660 | ACH97_201565 |
| iron-sulfur cluster assembly accessory protein | TRNA_RS38255 | BL14DL4_01648 | BLDA23_18480 | CK945_RS18790 | ACH97_201560 |
| diaminopimelate epimerase | TRNA_RS38260 | BL14DL4_01649 | BLDA23_18485 | CK945_RS18795 | ACH97_201555 |
| NupC/NupG family nucleoside CNT transporter | TRNA_RS38265 | BL14DL4_01650 | BLDA23_18490 | CK945_RS18800 | ACH97_201550 |
| DUF1450 domain-containing protein | TRNA_RS38270 | BL14DL4_01651 | BLDA23_18495 | CK945_RS18805 | ACH97_201545 |
| NAD(P)/FAD-dependent oxidoreductase | TRNA_RS38275 | BL14DL4_01652 | BLDA23_18500 | CK945_RS18810 | ACH97_201540 |
| iron chaperone | TRNA_RS38280 | BL14DL4_01653 | BLDA23_18505 | CK945_RS18815 | ACH97_201535 |
| hypothetical protein | TRNA_RS38285 | BL14DL4_01654 | BLDA23_18510 | CK945_RS18825 | ACH97_201525 |
| tetratricopeptide repeat protein | TRNA_RS38290 | BL14DL4_01655 | BLDA23_18515 | CK945_RS18830 | ACH97_201520 |
| phenolic acid decarboxylase | TRNA_RS38295 | BL14DL4_01656 | BLDA23_18520 | CK945_RS18835 | ACH97_201515 |
| hypothetical protein | TRNA_RS38300 | BL14DL4_01657 | BLDA23_18525 | CK945_RS18840 | ACH97_201510 |
| DUF1462 family protein | TRNA_RS38305 | BL14DL4_01658 | BLDA23_18530 | CK945_RS18845 | ACH97_201505 |
| NifU family protein | TRNA_RS38310 | BL14DL4_01659 | BLDA23_18535 | CK945_RS18850 | ACH97_201500 |
| S9 family peptidase | TRNA_RS38315 | BL14DL4_01660 | BLDA23_18540 | CK945_RS18855 | ACH97_201495 |
| homoserine kinase | TRNA_RS38320 | BL14DL4_01661 | BLDA23_18545 | CK945_RS18860 | ACH97_201490 |
| threonine synthase | TRNA_RS38325 | BL14DL4_01662 | BLDA23_18550 | CK945_RS18865 | ACH97_201485 |
| homoserine dehydrogenase | TRNA_RS38330 | BL14DL4_01663 | BLDA23_18555 | CK945_RS18870 | ACH97_201480 |
| D-glycerate dehydrogenase | TRNA_RS38335 | BL14DL4_01664 | BLDA23_18560 | CK945_RS18875 | ACH97_201475 |
| spore coat protein YutH | TRNA_RS38340 | BL14DL4_01665 | BLDA23_18565 | CK945_RS18880 | ACH97_201470 |
| phosphatidylglycerophosphatase A | TRNA_RS38345 | BL14DL4_01666 | BLDA23_18570 | CK945_RS18885 | ACH97_201465 |
| TIGR01457 family HAD-type hydrolase | TRNA_RS38350 | BL14DL4_01667 | BLDA23_18575 | CK945_RS18890 | ACH97_201460 |
| DUF86 domain-containing protein | TRNA_RS38355 | BL14DL4_01668 | BLDA23_18580 | CK945_RS18895 | ACH97_201455 |
| DUF1027 domain-containing protein | TRNA_RS38360 | BL14DL4_01669 | BLDA23_18585 | CK945_RS18900 | ACH97_201450 |
| hypothetical protein | TRNA_RS38365 | BL14DL4_01670 | BLDA23_18590 | CK945_RS18905 | ACH97_201445 |
| lipoyl synthase | TRNA_RS38370 | BL14DL4_01671 | BLDA23_18595 | CK945_RS18910 | ACH97_201440 |
| M23 family peptidase | TRNA_RS38375 | BL14DL4_01672 | BLDA23_18600 | CK945_RS18915 | ACH97_201435 |
| sodium-dependent transporter | TRNA_RS38380 | BL14DL4_01674 | BLDA23_18610 | CK945_RS18925 | ACH97_201430 |
| sporulation protein YunB | TRNA_RS38385 | BL14DL4_01675 | BLDA23_18615 | CK945_RS18930 | ACH97_201425 |
| DUF1805 domain-containing protein | TRNA_RS38390 | BL14DL4_01676 | BLDA23_18620 | CK945_RS18935 | ACH97_201420 |
| bifunctional metallophosphatase/5-nucleotidase | TRNA_RS38395 | BL14DL4_01677 | BLDA23_18625 | CK945_RS18940 | ACH97_201415 |
| sulfite exporter TauE/SafE family protein | TRNA_RS38400 | BL14DL4_01678 | BLDA23_18630 | CK945_RS18945 | ACH97_201410 |
| DUF72 domain-containing protein | TRNA_RS38405 | BL14DL4_01679 | BLDA23_18635 | CK945_RS18950 | ACH97_201405 |
| hypothetical protein | TRNA_RS38410 | BL14DL4_01680 | BLDA23_18640 | CK945_RS18955 | ACH97_201400 |
| TetR family transcriptional regulator | TRNA_RS38415 | BL14DL4_01681 | BLDA23_18645 | CK945_RS18960 | ACH97_201395 |
| alanine--glyoxylate aminotransferase family protein | TRNA_RS38425 | BL14DL4_01683 | BLDA23_18655 | CK945_RS18965 | ACH97_201390 |
| tetracycline resistance MFS efflux pump | TRNA_RS38440 | BL14DL4_01686 | BLDA23_18670 | CK945_RS18975 | ACH97_201380 |
| YfcC family protein | TRNA_RS38445 | BL14DL4_01687 | BLDA23_18675 | CK945_RS18980 | ACH97_201375 |
| ribonuclease | TRNA_RS38460 | BL14DL4_01690 | BLDA23_18690 | CK945_RS18995 | ACH97_201365 |
| xanthine dehydrogenase | TRNA_RS38465 | BL14DL4_01691 | BLDA23_18695 | CK945_RS19000 | ACH97_201360 |
| hypothetical protein | TRNA_RS38480 | BL14DL4_01694 | BLDA23_18710 | CK945_RS19015 | ACH97_201345 |
| aspartate phosphatase | TRNA_RS38485 | BL14DL4_01696 | BLDA23_18715 | CK945_RS19020 | ACH97_201340 |
| Fe-S cluster assembly protein SufB | TRNA_RS38490 | BL14DL4_01697 | BLDA23_18720 | CK945_RS19030 | ACH97_201330 |
| SUF system NifU family Fe-S cluster assembly protein | TRNA_RS38495 | BL14DL4_01698 | BLDA23_18725 | CK945_RS19035 | ACH97_201325 |
| cysteine desulfurase | TRNA_RS38500 | BL14DL4_01699 | BLDA23_18730 | CK945_RS19040 | ACH97_201320 |
| Fe-S cluster assembly protein SufD | TRNA_RS38505 | BL14DL4_01700 | BLDA23_18735 | CK945_RS19045 | ACH97_201315 |
| Fe-S cluster assembly ATPase SufC | TRNA_RS38510 | BL14DL4_01701 | BLDA23_18740 | CK945_RS19050 | ACH97_201310 |
| cation transporter | TRNA_RS38515 | BL14DL4_01702 | BLDA23_18745 | CK945_RS19055 | ACH97_201305 |
| carboxymuconolactone decarboxylase family protein | TRNA_RS38520 | BL14DL4_01703 | BLDA23_18750 | CK945_RS19060 | ACH97_201300 |
| ABC transporter substrate-binding protein | TRNA_RS38525 | BL14DL4_01704 | BLDA23_18755 | CK945_RS19065 | ACH97_201295 |
| ABC transporter permease | TRNA_RS38530 | BL14DL4_01705 | BLDA23_18760 | CK945_RS19070 | ACH97_201290 |
| methionine ABC transporter ATP-binding protein | TRNA_RS38535 | BL14DL4_01706 | BLDA23_18765 | CK945_RS19075 | ACH97_201285 |
| hypothetical protein | TRNA_RS38540 | BL14DL4_01707 | BLDA23_18770 | CK945_RS19080 | ACH97_201280 |
| thioredoxin | TRNA_RS38545 | BL14DL4_01708 | BLDA23_18775 | CK945_RS19085 | ACH97_201275 |
| hypothetical protein | TRNA_RS38550 | BL14DL4_01709 | BLDA23_18780 | CK945_RS19090 | ACH97_201270 |
| DUF2553 family protein | TRNA_RS38555 | BL14DL4_01710 | BLDA23_18785 | CK945_RS19095 | ACH97_201265 |
| glycine cleavage system protein GcvH | TRNA_RS38560 | BL14DL4_01711 | BLDA23_18790 | CK945_RS19100 | ACH97_201260 |
| arsenate reductase family protein | TRNA_RS38565 | BL14DL4_01712 | BLDA23_18795 | CK945_RS19105 | ACH97_201255 |
| acyl-CoA dehydrogenase | TRNA_RS38570 | BL14DL4_01713 | BLDA23_18800 | CK945_RS19110 | ACH97_201250 |
| acetyl-CoA C-acetyltransferase | TRNA_RS38575 | BL14DL4_01714 | BLDA23_18805 | CK945_RS19115 | ACH97_201245 |
| 3-hydroxyacyl-CoA dehydrogenase | TRNA_RS38580 | BL14DL4_01715 | BLDA23_18810 | CK945_RS19120 | ACH97_201240 |
| hypothetical protein | TRNA_RS38585 | BL14DL4_01717 | BLDA23_18820 | CK945_RS19130 | ACH97_201235 |
| spore coat protein | TRNA_RS38590 | BL14DL4_01718 | BLDA23_18825 | CK945_RS19135 | ACH97_201230 |
| DUF2573 family protein | TRNA_RS38595 | BL14DL4_01719 | BLDA23_18830 | CK945_RS19140 | ACH97_201225 |
| siderophore biosynthesis protein | TRNA_RS38600 | BL14DL4_01720 | BLDA23_18835 | CK945_RS19145 | ACH97_201220 |
| ABC transporter ATP-binding protein | TRNA_RS38605 | BL14DL4_01721 | BLDA23_18840 | CK945_RS19150 | ACH97_201215 |
| iron ABC transporter permease | TRNA_RS38610 | BL14DL4_01722 | BLDA23_18845 | CK945_RS19155 | ACH97_201210 |
| iron ABC transporter permease | TRNA_RS38615 | BL14DL4_01723 | BLDA23_18850 | CK945_RS19160 | ACH97_201205 |
| iron-siderophore ABC transporter substrate-binding protein | TRNA_RS38620 | BL14DL4_01724 | BLDA23_18855 | CK945_RS19165 | ACH97_201200 |
| hypothetical protein | TRNA_RS38625 | BL14DL4_01725 | BLDA23_18860 | CK945_RS19170 | ACH97_201195 |
| M3 family oligoendopeptidase | TRNA_RS38630 | BL14DL4_01726 | BLDA23_18865 | CK945_RS19175 | ACH97_201190 |
| D-alanyl-D-alanine carboxypeptidase | TRNA_RS38635 | BL14DL4_01727 | BLDA23_18870 | CK945_RS19180 | ACH97_201185 |
| SDR family oxidoreductase | TRNA_RS38640 | BL14DL4_01728 | BLDA23_18875 | CK945_RS19185 | ACH97_201180 |
| DNA starvation/stationary phase protection protein | TRNA_RS38645 | BL14DL4_01729 | BLDA23_18880 | CK945_RS19190 | ACH97_201175 |
| serine protease | TRNA_RS38650 | BL14DL4_01730 | BLDA23_18885 | CK945_RS19200 | ACH97_201170 |
| DNA-binding response regulator | TRNA_RS38655 | BL14DL4_01731 | BLDA23_18890 | CK945_RS19205 | ACH97_201165 |
| sensor histidine kinase | TRNA_RS38660 | BL14DL4_01732 | BLDA23_18895 | CK945_RS19210 | ACH97_201160 |
| TetR/AcrR family transcriptional regulator | TRNA_RS38665 | BL14DL4_01734 | BLDA23_18900 | CK945_RS19220 | ACH97_201155 |
| class II fumarate hydratase | TRNA_RS38670 | BL14DL4_01735 | BLDA23_18905 | CK945_RS19225 | ACH97_201150 |
| spore germination protein | TRNA_RS38675 | BL14DL4_01737 | BLDA23_18915 | CK945_RS19235 | ACH97_201145 |
| germination protein | TRNA_RS38680 | BL14DL4_01738 | BLDA23_18920 | CK945_RS19240 | ACH97_201140 |
| Ger(x)C family spore germination protein | TRNA_RS38685 | BL14DL4_01739 | BLDA23_18925 | CK945_RS19245 | ACH97_201135 |
| hypothetical protein | TRNA_RS38690 | BL14DL4_01740 | BLDA23_18930 | CK945_RS19250 | ACH97_201130 |
| DNA-binding response regulator | TRNA_RS38695 | BL14DL4_01741 | BLDA23_18935 | CK945_RS19255 | ACH97_201125 |
| sensor histidine kinase | TRNA_RS38700 | BL14DL4_01742 | BLDA23_18940 | CK945_RS19260 | ACH97_201120 |
| hypothetical protein | TRNA_RS38705 | BL14DL4_01743 | BLDA23_18945 | CK945_RS19265 | ACH97_201115 |
| DUF4097 domain-containing protein | TRNA_RS38710 | BL14DL4_01744 | BLDA23_18950 | CK945_RS19270 | ACH97_201110 |
| PspA/IM30 family protein | TRNA_RS38715 | BL14DL4_01745 | BLDA23_18955 | CK945_RS19275 | ACH97_201105 |
| protein liaI | TRNA_RS38720 | BL14DL4_01746 | BLDA23_18960 | CK945_RS19280 | ACH97_201100 |
| carbohydrate esterase | TRNA_RS38725 | BL14DL4_01747 | BLDA23_18965 | CK945_RS19285 | ACH97_201095 |
| cob(I)yrinic acid a,c-diamide adenosyltransferase | TRNA_RS38730 | BL14DL4_01748 | BLDA23_18970 | CK945_RS19290 | ACH97_201090 |
| 4-hydroxy-tetrahydrodipicolinate synthase | TRNA_RS38760 | BL14DL4_01752 | BLDA23_18990 | CK945_RS19320 | ACH97_201060 |
| ABC transporter ATP-binding protein | TRNA_RS38765 | BL14DL4_01753 | BLDA23_18995 | CK945_RS19325 | ACH97_201055 |
| iron ABC transporter permease | TRNA_RS38770 | BL14DL4_01754 | BLDA23_19000 | CK945_RS19330 | ACH97_201050 |
| ABC transporter substrate-binding protein | TRNA_RS38775 | BL14DL4_01755 | BLDA23_19005 | CK945_RS19335 | ACH97_201045 |
| GntR family transcriptional regulator | TRNA_RS38780 | BL14DL4_01756 | BLDA23_19010 | CK945_RS19340 | ACH97_201040 |
| SDR family oxidoreductase | TRNA_RS38785 | BL14DL4_01757 | BLDA23_19015 | CK945_RS19345 | ACH97_201035 |
| MOSC domain-containing protein | TRNA_RS38790 | BL14DL4_01758 | BLDA23_19020 | CK945_RS19350 | ACH97_201030 |
| LysR family transcriptional regulator | TRNA_RS38795 | BL14DL4_01759 | BLDA23_19025 | CK945_RS19355 | ACH97_201025 |
| glycoside hydrolase family 28 protein | TRNA_RS38800 | BL14DL4_01760 | BLDA23_19030 | CK945_RS19360 | ACH97_201020 |
| tagaturonate reductase | TRNA_RS38810 | BL14DL4_01762 | BLDA23_19040 | CK945_RS19370 | ACH97_201010 |
| LacI family transcriptional regulator | TRNA_RS38815 | BL14DL4_01763 | BLDA23_19045 | CK945_RS19375 | ACH97_201005 |
| MFS transporter | TRNA_RS38820 | BL14DL4_01764 | BLDA23_19050 | CK945_RS19380 | ACH97_201000 |
| glucuronate isomerase | TRNA_RS38825 | BL14DL4_01765 | BLDA23_19055 | CK945_RS19385 | ACH97_200995 |
| sensor histidine kinase | TRNA_RS38830 | BL14DL4_01766 | BLDA23_19060 | CK945_RS19390 | ACH97_200990 |
| DNA-binding response regulator | TRNA_RS38835 | BL14DL4_01767 | BLDA23_19065 | CK945_RS19395 | ACH97_200985 |
| ABC transporter ATP-binding protein | TRNA_RS38840 | BL14DL4_01768 | BLDA23_19070 | CK945_RS19400 | ACH97_200980 |
| iron ABC transporter permease | TRNA_RS38845 | BL14DL4_01769 | BLDA23_19075 | CK945_RS19405 | ACH97_200975 |
| iron ABC transporter permease | TRNA_RS38850 | BL14DL4_01770 | BLDA23_19080 | CK945_RS19410 | ACH97_200970 |
| metal-dependent hydrolase | TRNA_RS38865 | BL14DL4_01772 | BLDA23_19095 | CK945_RS19425 | ACH97_200955 |
| aldo/keto reductase | TRNA_RS38880 | BL14DL4_01775 | BLDA23_19110 | CK945_RS19440 | ACH97_200940 |
| ROK family protein | TRNA_RS38885 | BL14DL4_01776 | BLDA23_19115 | CK945_RS19445 | ACH97_200935 |
| aminotransferase class V-fold PLP-dependent enzyme | TRNA_RS38890 | BL14DL4_01777 | BLDA23_19120 | CK945_RS19450 | ACH97_200930 |
| hypothetical protein | TRNA_RS38895 | BL14DL4_01778 | BLDA23_19125 | CK945_RS19455 | ACH97_200925 |
| helicase | TRNA_RS38900 | BL14DL4_01779 | BLDA23_19130 | CK945_RS19465 | ACH97_200915 |
| trimeric intracellular cation channel family protein | TRNA_RS38905 | BL14DL4_01780 | BLDA23_19135 | CK945_RS19470 | ACH97_200910 |
| GNAT family N-acetyltransferase | TRNA_RS38915 | BL14DL4_01783 | BLDA23_19145 | CK945_RS19480 | ACH97_200900 |
| disulfide bond formation protein B | TRNA_RS38920 | BL14DL4_01784 | BLDA23_19150 | CK945_RS19485 | ACH97_200895 |
| DsbA family protein | TRNA_RS38925 | BL14DL4_01785 | BLDA23_19155 | CK945_RS19490 | ACH97_200890 |
| cadmium-translocating P-type ATPase | TRNA_RS38930 | BL14DL4_01786 | BLDA23_19160 | CK945_RS19495 | ACH97_200885 |
| ABC transporter substrate-binding protein | TRNA_RS38935 | BL14DL4_01787 | BLDA23_19165 | CK945_RS19500 | ACH97_200880 |
| sugar ABC transporter permease | TRNA_RS38940 | BL14DL4_01788 | BLDA23_19170 | CK945_RS19505 | ACH97_200875 |
| carbohydrate ABC transporter permease | TRNA_RS38945 | BL14DL4_01789 | BLDA23_19175 | CK945_RS19510 | ACH97_200870 |
| glycoside hydrolase family 3 | TRNA_RS38955 | BL14DL4_01791 | BLDA23_19185 | CK945_RS19520 | ACH97_200860 |
| metal ABC transporter ATP-binding protein | TRNA_RS38960 | BL14DL4_01792 | BLDA23_19190 | CK945_RS19525 | ACH97_200855 |
| metal ABC transporter permease | TRNA_RS38965 | BL14DL4_01793 | BLDA23_19195 | CK945_RS19530 | ACH97_200850 |
| metal ABC transporter substrate-binding protein | TRNA_RS38970 | BL14DL4_01794 | BLDA23_19200 | CK945_RS19535 | ACH97_200845 |
| four-helix bundle copper-binding protein | TRNA_RS38975 | BL14DL4_01795 | BLDA23_19205 | CK945_RS19540 | ACH97_200840 |
| 1-phosphofructokinase | TRNA_RS38985 | BL14DL4_01797 | BLDA23_19215 | CK945_RS19550 | ACH97_200830 |
| PTS fructose transporter subunit IIC | TRNA_RS38990 | BL14DL4_01798 | BLDA23_19220 | CK945_RS19555 | ACH97_200825 |
| PTS sugar transporter subunit IIA | TRNA_RS38995 | BL14DL4_01799 | BLDA23_19225 | CK945_RS19560 | ACH97_200820 |
| tagatose bisphosphate family class II aldolase | TRNA_RS39000 | BL14DL4_01800 | BLDA23_19230 | CK945_RS19565 | ACH97_200815 |
| copper-translocating P-type ATPase | TRNA_RS39005 | BL14DL4_01802 | BLDA23_19235 | CK945_RS19570 | ACH97_200810 |
| copper chaperone | TRNA_RS39010 | BL14DL4_01803 | BLDA23_19240 | CK945_RS19575 | ACH97_200805 |
| transcriptional regulator | TRNA_RS39015 | BL14DL4_01804 | BLDA23_19245 | CK945_RS19580 | ACH97_200800 |
| DJ-1/PfpI family protein | TRNA_RS39020 | BL14DL4_01805 | BLDA23_19250 | CK945_RS19585 | ACH97_200795 |
| DeoR/GlpR transcriptional regulator | TRNA_RS39025 | BL14DL4_01806 | BLDA23_19255 | CK945_RS19590 | ACH97_200790 |
| rhamnulokinase | TRNA_RS39030 | BL14DL4_01807 | BLDA23_19260 | CK945_RS19595 | ACH97_200785 |
| L-rhamnose isomerase | TRNA_RS39035 | BL14DL4_01808 | BLDA23_19265 | CK945_RS19600 | ACH97_200780 |
| hypothetical protein | TRNA_RS39040 | BL14DL4_01809 | BLDA23_19270 | CK945_RS19605 | ACH97_200775 |
| bifunctional rhamnulose-1-phosphate aldolase/short-chain dehydrogenase | TRNA_RS39045 | BL14DL4_01810 | BLDA23_19275 | CK945_RS19610 | ACH97_200770 |
| MFS transporter | TRNA_RS39055 | BL14DL4_01812 | BLDA23_19285 | CK945_RS19625 | ACH97_200760 |
| tRNA-dependent cyclodipeptide synthase | TRNA_RS39060 | BL14DL4_01813 | BLDA23_19290 | CK945_RS19630 | ACH97_200755 |
| cytochrome P450, cyclodipeptide synthase-associated | TRNA_RS39065 | BL14DL4_01814 | BLDA23_19295 | CK945_RS19635 | ACH97_200750 |
| nuclear transport factor 2 family protein | TRNA_RS39070 | BL14DL4_01815 | BLDA23_19300 | CK945_RS19640 | ACH97_200745 |
| transcriptional regulator | TRNA_RS39075 | BL14DL4_01816 | BLDA23_19305 | CK945_RS19645 | ACH97_200740 |
| oxidoreductase | TRNA_RS39085 | BL14DL4_01818 | BLDA23_19315 | CK945_RS19655 | ACH97_200730 |
| FMN-dependent NADH-azoreductase 2 | TRNA_RS39090 | BL14DL4_01819 | BLDA23_19320 | CK945_RS19660 | ACH97_200725 |
| rhodanese-related sulfurtransferase | TRNA_RS39095 | BL14DL4_01821 | BLDA23_19325 | CK945_RS19665 | ACH97_200720 |
| hypothetical protein | TRNA_RS39100 | BL14DL4_01822 | BLDA23_19330 | CK945_RS19670 | ACH97_200715 |
| sigma-70 family RNA polymerase sigma factor | TRNA_RS39105 | BL14DL4_01823 | BLDA23_19335 | CK945_RS19675 | ACH97_200710 |
| cupin domain-containing protein | TRNA_RS39110 | BL14DL4_01825 | BLDA23_19345 | CK945_RS19685 | ACH97_200705 |
| peptidase | TRNA_RS39115 | BL14DL4_01826 | BLDA23_19350 | CK945_RS19690 | ACH97_200700 |
| hypothetical protein | TRNA_RS39120 | BL14DL4_01827 | BLDA23_19355 | CK945_RS19695 | ACH97_200695 |
| hypothetical protein | TRNA_RS39130 | BL14DL4_01829 | BLDA23_19380 | CK945_RS19730 | ACH97_200660 |
| XkdX family protein | TRNA_RS39205 | BL14DL4_01532 | BLDA23_15735 | CK945_RS05635 | ACH97_210730 |
| hypothetical protein | TRNA_RS39210 | BL14DL4_01533 | BLDA23_15740 | CK945_RS05630 | ACH97_210725 |
| alkaline phosphatase | TRNA_RS39225 | BL14DL4_01844 | BLDA23_17920 | CK945_RS05615 | ACH97_200570 |
| phage tail family protein | TRNA_RS39230 | BL14DL4_01845 | BLDA23_17925 | CK945_RS05610 | ACH97_200565 |
| phage tail tape measure protein | TRNA_RS39235 | BL14DL4_01846 | BLDA23_17930 | CK945_RS05605 | ACH97_200560 |
| hypothetical protein | TRNA_RS39240 | BL14DL4_01848 | BLDA23_17935 | CK945_RS05600 | ACH97_200555 |
| tail protein | TRNA_RS39245 | BL14DL4_01849 | BLDA23_17940 | CK945_RS05595 | ACH97_200550 |
| hypothetical protein | TRNA_RS39250 | BL14DL4_01850 | BLDA23_17945 | CK945_RS05590 | ACH97_200545 |
| hypothetical protein | TRNA_RS39255 | BL14DL4_01851 | BLDA23_17950 | CK945_RS05585 | ACH97_200540 |
| head-tail adaptor protein | TRNA_RS39260 | BL14DL4_01852 | BLDA23_17955 | CK945_RS05580 | ACH97_200535 |
| phage gp6-like head-tail connector protein | TRNA_RS39265 | BL14DL4_01853 | BLDA23_17960 | CK945_RS05575 | ACH97_200530 |
| collagen-like protein | TRNA_RS39270 | BL14DL4_01854 | BLDA23_17965 | CK945_RS12010 | ACH97_200525 |
| phage major capsid protein | TRNA_RS39275 | BL14DL4_01855 | BLDA23_17970 | CK945_RS05565 | ACH97_200520 |
| Clp protease ClpP | TRNA_RS39280 | BL14DL4_01856 | BLDA23_17975 | CK945_RS05560 | ACH97_200515 |
| phage portal protein | TRNA_RS39285 | BL14DL4_01857 | BLDA23_17980 | CK945_RS05555 | ACH97_200510 |
| DUF1056 family protein | TRNA_RS39290 | BL14DL4_01858 | BLDA23_17985 | CK945_RS05550 | ACH97_200505 |
| terminase large subunit | TRNA_RS39295 | BL14DL4_01859 | BLDA23_17990 | CK945_RS05545 | ACH97_200500 |
| phage terminase small subunit P27 family | TRNA_RS39300 | BL14DL4_01860 | BLDA23_17995 | CK945_RS05540 | ACH97_200495 |
| HNH endonuclease | TRNA_RS39305 | BL14DL4_01861 | BLDA23_18000 | CK945_RS05535 | ACH97_200490 |
| hypothetical protein | TRNA_RS39310 | BL14DL4_01862 | BLDA23_18005 | CK945_RS05530 | ACH97_200485 |
| ArpU family transcriptional regulator | TRNA_RS39315 | BL14DL4_01866 | BLDA23_15850 | CK945_RS12075 | ACH97_200480 |
| hypothetical protein | TRNA_RS39330 | BL14DL4_01871 | BLDA23_18045 | CK945_RS12110 | ACH97_200465 |
| hypothetical protein | TRNA_RS39335 | BL14DL4_01872 | BLDA23_18050 | CK945_RS12115 | ACH97_200460 |
| primase | TRNA_RS39345 | BL14DL4_01562 | BLDA23_15885 | CK945_RS12120 | ACH97_200450 |
| DUF669 domain-containing protein | TRNA_RS39350 | BL14DL4_01875 | BLDA23_15890 | CK945_RS12125 | ACH97_200445 |
| hypothetical protein | TRNA_RS39355 | BL14DL4_01876 | BLDA23_18065 | CK945_RS12130 | ACH97_200440 |
| hypothetical protein | TRNA_RS39360 | BL14DL4_01877 | BLDA23_18070 | CK945_RS12135 | ACH97_200435 |
| hypothetical protein | TRNA_RS39365 | BL14DL4_01878 | BLDA23_18075 | CK945_RS12140 | ACH97_200430 |
| hypothetical protein | TRNA_RS39370 | BL14DL4_01880 | BLDA23_18080 | CK945_RS12145 | ACH97_200425 |
| SsrA-binding protein | TRNA_RS39415 | BL14DL4_01890 | BLDA23_19395 | CK945_RS19745 | ACH97_200380 |
| ribonuclease R | TRNA_RS39420 | BL14DL4_01891 | BLDA23_19400 | CK945_RS19750 | ACH97_200375 |
| carboxylesterase | TRNA_RS39425 | BL14DL4_01892 | BLDA23_19405 | CK945_RS19755 | ACH97_200370 |
| preprotein translocase subunit SecG | TRNA_RS39430 | BL14DL4_01893 | BLDA23_19410 | CK945_RS19760 | ACH97_200365 |
| AbrB/MazE/SpoVT family DNA-binding domain-containing protein | TRNA_RS39435 | BL14DL4_01894 | BLDA23_19415 | CK945_RS19765 | ACH97_200360 |
| XRE family transcriptional regulator | TRNA_RS39440 | BL14DL4_01895 | BLDA23_19420 | CK945_RS19770 | ACH97_200355 |
| transcriptional regulator | TRNA_RS39445 | BL14DL4_01896 | BLDA23_19425 | CK945_RS19775 | ACH97_200350 |
| XRE family transcriptional regulator | TRNA_RS39450 | BL14DL4_01897 | BLDA23_19430 | CK945_RS19780 | ACH97_200345 |
| ABC transporter permease | TRNA_RS39455 | BL14DL4_01898 | BLDA23_19435 | CK945_RS19785 | ACH97_200340 |
| osmoprotectant ABC transporter substrate-binding protein | TRNA_RS39460 | BL14DL4_01899 | BLDA23_19440 | CK945_RS19790 | ACH97_200335 |
| ABC transporter permease | TRNA_RS39465 | BL14DL4_01900 | BLDA23_19445 | CK945_RS19795 | ACH97_200330 |
| ATP-binding cassette domain-containing protein | TRNA_RS39470 | BL14DL4_01901 | BLDA23_19450 | CK945_RS19800 | ACH97_200325 |
| GbsR/MarR family transcriptional regulator | TRNA_RS39475 | BL14DL4_01902 | BLDA23_19455 | CK945_RS19805 | ACH97_200320 |
| NAAT family transporter | TRNA_RS39480 | BL14DL4_01903 | BLDA23_19460 | CK945_RS19810 | ACH97_200315 |
| zinc ribbon domain-containing protein | TRNA_RS39485 | BL14DL4_01904 | BLDA23_19465 | CK945_RS19815 | ACH97_200310 |
| iron-siderophore ABC transporter substrate-binding protein | TRNA_RS39495 | BL14DL4_01907 | BLDA23_19475 | CK945_RS19825 | ACH97_200300 |
| hypothetical protein | TRNA_RS39500 | BL14DL4_01908 | BLDA23_19480 | CK945_RS19835 | ACH97_200290 |
| phosphopyruvate hydratase | TRNA_RS39510 | BL14DL4_01910 | BLDA23_19495 | CK945_RS19850 | ACH97_200280 |
| 2,3-bisphosphoglycerate-independent phosphoglycerate mutase | TRNA_RS39515 | BL14DL4_01911 | BLDA23_19500 | CK945_RS19855 | ACH97_200275 |
| triose-phosphate isomerase | TRNA_RS39520 | BL14DL4_01912 | BLDA23_19505 | CK945_RS19860 | ACH97_200270 |
| phosphoglycerate kinase | TRNA_RS39525 | BL14DL4_01913 | BLDA23_19510 | CK945_RS19865 | ACH97_200265 |
| type I glyceraldehyde-3-phosphate dehydrogenase | TRNA_RS39530 | BL14DL4_01914 | BLDA23_19520 | CK945_RS19870 | ACH97_200260 |
| sugar-binding transcriptional regulator | TRNA_RS39535 | BL14DL4_01915 | BLDA23_19525 | CK945_RS19875 | ACH97_200255 |
| LLM class flavin-dependent oxidoreductase | TRNA_RS39540 | BL14DL4_01916 | BLDA23_19530 | CK945_RS19880 | ACH97_200250 |
| amino acid permease | TRNA_RS39545 | BL14DL4_01917 | BLDA23_19535 | CK945_RS19885 | ACH97_200245 |
| hypothetical protein | TRNA_RS39550 | BL14DL4_01918 | BLDA23_19540 | CK945_RS19895 | ACH97_200235 |
| ABC transporter ATP-binding protein | TRNA_RS39555 | BL14DL4_01919 | BLDA23_19545 | CK945_RS19900 | ACH97_200230 |
| membrane protein | TRNA_RS39560 | BL14DL4_01920 | BLDA23_19550 | CK945_RS19905 | ACH97_200225 |
| lactate utilization protein C | TRNA_RS39565 | BL14DL4_01921 | BLDA23_19555 | CK945_RS19910 | ACH97_200220 |
| iron-sulfur cluster-binding protein | TRNA_RS39570 | BL14DL4_01922 | BLDA23_19560 | CK945_RS19915 | ACH97_200215 |
| lactate utilization protein A | TRNA_RS39575 | BL14DL4_01923 | BLDA23_19565 | CK945_RS19920 | ACH97_200210 |
| FadR family transcriptional regulator | TRNA_RS39580 | BL14DL4_01924 | BLDA23_19570 | CK945_RS19925 | ACH97_200205 |
| L-lactate permease | TRNA_RS39585 | BL14DL4_01925 | BLDA23_19575 | CK945_RS19930 | ACH97_200200 |
| RNA polymerase sigma-54 factor | TRNA_RS39590 | BL14DL4_01926 | BLDA23_19580 | CK945_RS19935 | ACH97_200195 |
| hypothetical protein | TRNA_RS39595 | BL14DL4_01927 | BLDA23_19585 | CK945_RS19940 | ACH97_200190 |
| pyruvyl transferase | TRNA_RS39600 | BL14DL4_01928 | BLDA23_19590 | CK945_RS19945 | ACH97_200185 |
| aminotransferase class I/II-fold pyridoxal phosphate-dependent enzyme | TRNA_RS39605 | BL14DL4_01929 | BLDA23_19595 | CK945_RS19950 | ACH97_200180 |
| acetyltransferase | TRNA_RS39610 | BL14DL4_01930 | BLDA23_19600 | CK945_RS19955 | ACH97_200175 |
| sugar transferase | TRNA_RS39615 | BL14DL4_01931 | BLDA23_19605 | CK945_RS19960 | ACH97_200170 |
| membrane protein | TRNA_RS39620 | BL14DL4_01932 | BLDA23_19610 | CK945_RS19965 | ACH97_200165 |
| glycosyltransferase | TRNA_RS39625 | BL14DL4_01933 | BLDA23_19615 | CK945_RS19970 | ACH97_200160 |
| pyruvyl transferase | TRNA_RS39630 | BL14DL4_01934 | BLDA23_19620 | CK945_RS19975 | ACH97_200155 |
| glycosyltransferase | TRNA_RS39635 | BL14DL4_01935 | BLDA23_19625 | CK945_RS19980 | ACH97_200150 |
| EpsG family protein | TRNA_RS39640 | BL14DL4_01936 | BLDA23_19630 | CK945_RS19985 | ACH97_200145 |
| glycosyltransferase family 1 protein | TRNA_RS39645 | BL14DL4_01937 | BLDA23_19635 | CK945_RS19990 | ACH97_200140 |
| glycosyltransferase | TRNA_RS39650 | BL14DL4_01938 | BLDA23_19640 | CK945_RS19995 | ACH97_200135 |
| glycosyltransferase family 1 protein | TRNA_RS39655 | BL14DL4_01939 | BLDA23_19645 | CK945_RS20000 | ACH97_200130 |
| polysaccharide biosynthesis protein | TRNA_RS39660 | BL14DL4_01940 | BLDA23_19650 | CK945_RS20005 | ACH97_200125 |
| tyrosine-protein kinase family protein | TRNA_RS39665 | BL14DL4_01941 | BLDA23_19655 | CK945_RS20010 | ACH97_200120 |
| capsular polysaccharide biosynthesis protein | TRNA_RS39670 | BL14DL4_01942 | BLDA23_19660 | CK945_RS20015 | ACH97_200115 |
| helix-turn-helix domain-containing protein | TRNA_RS39675 | BL14DL4_01943 | BLDA23_19665 | CK945_RS20020 | ACH97_200110 |
| general stress protein | TRNA_RS39680 | BL14DL4_01945 | BLDA23_19670 | CK945_RS20025 | ACH97_200105 |
| MFS transporter | TRNA_RS39685 | BL14DL4_01946 | BLDA23_19675 | CK945_RS20030 | ACH97_200100 |
| hydroxyacid dehydrogenase | TRNA_RS39690 | BL14DL4_01947 | BLDA23_19680 | CK945_RS20035 | ACH97_200095 |
| 4-hydroxythreonine-4-phosphate dehydrogenase PdxA | TRNA_RS39695 | BL14DL4_01948 | BLDA23_19685 | CK945_RS20040 | ACH97_200090 |
| hypothetical protein | TRNA_RS39700 | BL14DL4_01949 | BLDA23_19690 | CK945_RS20045 | ACH97_200085 |
| iron-containing alcohol dehydrogenase | TRNA_RS39705 | BL14DL4_01950 | BLDA23_19695 | CK945_RS20050 | ACH97_200080 |
| 4-hydroxy-tetrahydrodipicolinate synthase | TRNA_RS39710 | BL14DL4_01951 | BLDA23_19700 | CK945_RS20055 | ACH97_200075 |
| propionate catabolism operon regulatory protein PrpR | TRNA_RS39715 | BL14DL4_01952 | BLDA23_19705 | CK945_RS20060 | ACH97_200070 |
| transcriptional regulator | TRNA_RS39720 | BL14DL4_01953 | BLDA23_19710 | CK945_RS20065 | ACH97_200065 |
| NADH-dependent flavin oxidoreductase | TRNA_RS39725 | BL14DL4_01954 | BLDA23_19715 | CK945_RS20070 | ACH97_200060 |
| glycoside hydrolase 68 family protein | TRNA_RS39730 | BL14DL4_01955 | BLDA23_19720 | CK945_RS20075 | ACH97_200055 |
| ATP-dependent Clp protease proteolytic subunit | TRNA_RS39750 | BL14DL4_01959 | BLDA23_19740 | CK945_RS20095 | ACH97_215745 |
| TIGR00730 family Rossman fold protein | TRNA_RS39755 | BL14DL4_01960 | BLDA23_19745 | CK945_RS20100 | ACH97_215750 |
| hypothetical protein | TRNA_RS39760 | BL14DL4_01961 | BLDA23_19750 | CK945_RS20105 | ACH97_215755 |
| HD domain-containing protein | TRNA_RS39765 | BL14DL4_01962 | BLDA23_19755 | CK945_RS20110 | ACH97_215760 |
| STAS domain-containing protein | TRNA_RS39770 | BL14DL4_01963 | BLDA23_19760 | CK945_RS20115 | ACH97_215765 |
| carbonic anhydrase | TRNA_RS39775 | BL14DL4_01964 | BLDA23_19765 | CK945_RS20120 | ACH97_215770 |
| D-glycerate dehydrogenase | TRNA_RS39780 | BL14DL4_01965 | BLDA23_19770 | CK945_RS20125 | ACH97_215775 |
| flavin reductase family protein | TRNA_RS39785 | BL14DL4_01966 | BLDA23_19775 | CK945_RS20130 | ACH97_215780 |
| ribonuclease | TRNA_RS39795 | BL14DL4_01968 | BLDA23_19785 | CK945_RS20140 | ACH97_215790 |
| barnase inhibitor | TRNA_RS39800 | BL14DL4_01969 | BLDA23_19790 | CK945_RS20145 | ACH97_215795 |
| HPr family phosphocarrier protein | TRNA_RS39810 | BL14DL4_01971 | BLDA23_19800 | CK945_RS20155 | ACH97_215805 |
| DNA-binding protein WhiA | TRNA_RS39815 | BL14DL4_01972 | BLDA23_19805 | CK945_RS20160 | ACH97_215810 |
| RNase adapter RapZ | TRNA_RS39825 | BL14DL4_01974 | BLDA23_19815 | CK945_RS20170 | ACH97_215820 |
| 8-oxo-dGTP diphosphatase | TRNA_RS39830 | BL14DL4_01975 | BLDA23_19820 | CK945_RS20175 | ACH97_215825 |
| thioredoxin-disulfide reductase | TRNA_RS39840 | BL14DL4_01976 | BLDA23_19830 | CK945_RS20185 | ACH97_215835 |
| hypothetical protein | TRNA_RS39845 | BL14DL4_01977 | BLDA23_19835 | CK945_RS20190 | ACH97_215840 |
| GNAT family N-acetyltransferase | TRNA_RS39850 | BL14DL4_01978 | BLDA23_19840 | CK945_RS20195 | ACH97_215845 |
| bifunctional phosphoribosyl-AMP cyclohydrolase/phosphoribosyl-ATP diphosphatase HisIE | TRNA_RS39855 | BL14DL4_01979 | BLDA23_19845 | CK945_RS20200 | ACH97_215850 |
| imidazole glycerol phosphate synthase cyclase subunit | TRNA_RS39860 | BL14DL4_01980 | BLDA23_19850 | CK945_RS20205 | ACH97_215855 |
| 1-(5-phosphoribosyl)-5-((5- phosphoribosylamino)methylideneamino)imidazole-4- carboxamide isomerase | TRNA_RS39865 | BL14DL4_01981 | BLDA23_19855 | CK945_RS20210 | ACH97_215860 |
| imidazole glycerol phosphate synthase subunit HisH | TRNA_RS39870 | BL14DL4_01982 | BLDA23_19860 | CK945_RS20215 | ACH97_215865 |
| imidazoleglycerol-phosphate dehydratase | TRNA_RS39875 | BL14DL4_01983 | BLDA23_19865 | CK945_RS20220 | ACH97_215870 |
| histidinol dehydrogenase | TRNA_RS39880 | BL14DL4_01984 | BLDA23_19870 | CK945_RS20225 | ACH97_215875 |
| ATP phosphoribosyltransferase | TRNA_RS39885 | BL14DL4_01985 | BLDA23_19875 | CK945_RS20230 | ACH97_215880 |
| ATP phosphoribosyltransferase regulatory subunit | TRNA_RS39890 | BL14DL4_01986 | BLDA23_19880 | CK945_RS20235 | ACH97_215885 |
| hypothetical protein | TRNA_RS39895 | BL14DL4_01987 | BLDA23_19885 | CK945_RS20240 | ACH97_215890 |
| pectate lyase | TRNA_RS39900 | BL14DL4_01988 | BLDA23_19890 | CK945_RS20245 | ACH97_215895 |
| acyltransferase | TRNA_RS39905 | BL14DL4_01989 | BLDA23_19895 | CK945_RS20250 | ACH97_215900 |
| pyrophosphatase PpaX | TRNA_RS39910 | BL14DL4_01990 | BLDA23_19900 | CK945_RS20255 | ACH97_215905 |
| membrane protein | TRNA_RS39915 | BL14DL4_01991 | BLDA23_19905 | CK945_RS20260 | ACH97_215910 |
| prolipoprotein diacylglyceryl transferase | TRNA_RS39920 | BL14DL4_01992 | BLDA23_19910 | CK945_RS20265 | ACH97_215915 |
| HPr kinase/phosphorylase | TRNA_RS39925 | BL14DL4_01993 | BLDA23_19915 | CK945_RS20270 | ACH97_215920 |
| MarR family transcriptional regulator | TRNA_RS39930 | BL14DL4_01994 | BLDA23_19920 | CK945_RS20275 | ACH97_215925 |
| MFS transporter | TRNA_RS39935 | BL14DL4_01995 | BLDA23_19925 | CK945_RS20280 | ACH97_215930 |
| hypothetical protein | TRNA_RS39940 | BL14DL4_01996 | BLDA23_19930 | CK945_RS20285 | ACH97_215935 |
| phage holin family protein | TRNA_RS39945 | BL14DL4_01997 | BLDA23_19935 | CK945_RS20290 | ACH97_215940 |
| PspC domain-containing protein | TRNA_RS39950 | BL14DL4_01998 | BLDA23_19940 | CK945_RS20295 | ACH97_215945 |
| DUF4097 domain-containing protein | TRNA_RS39955 | BL14DL4_01999 | BLDA23_19945 | CK945_RS20300 | ACH97_215950 |
| hypothetical protein | TRNA_RS39960 | BL14DL4_02000 | BLDA23_19950 | CK945_RS20305 | ACH97_215955 |
| hypothetical protein | TRNA_RS39965 | BL14DL4_02001 | BLDA23_19955 | CK945_RS20310 | ACH97_215960 |
| excinuclease ABC subunit UvrA | TRNA_RS39980 | BL14DL4_02003 | BLDA23_19970 | CK945_RS20320 | ACH97_215975 |
| excinuclease ABC subunit B | TRNA_RS39985 | BL14DL4_02004 | BLDA23_19975 | CK945_RS20325 | ACH97_215980 |
| DUF2198 family protein | TRNA_RS39990 | BL14DL4_02005 | BLDA23_19980 | CK945_RS20330 | ACH97_215985 |
| hypothetical protein | TRNA_RS39995 | BL14DL4_02006 | BLDA23_19985 | CK945_RS20335 | ACH97_215990 |
| PDZ domain-containing protein | TRNA_RS40000 | BL14DL4_02007 | BLDA23_19990 | CK945_RS20340 | ACH97_215995 |
| hypothetical protein | TRNA_RS40005 | BL14DL4_02008 | BLDA23_19995 | CK945_RS20345 | ACH97_216000 |
| S41 family peptidase | TRNA_RS40010 | BL14DL4_02009 | BLDA23_20000 | CK945_RS20350 | ACH97_216005 |
| tartrate dehydrogenase | TRNA_RS40015 | BL14DL4_02010 | BLDA23_20005 | CK945_RS20355 | ACH97_216010 |
| peptidase M23 | TRNA_RS40020 | BL14DL4_02011 | BLDA23_20010 | CK945_RS20360 | ACH97_216015 |
| ABC transporter permease | TRNA_RS40025 | BL14DL4_02012 | BLDA23_20015 | CK945_RS20365 | ACH97_216020 |
| cell division ATP-binding protein FtsE | TRNA_RS40030 | BL14DL4_02013 | BLDA23_20020 | CK945_RS20370 | ACH97_216025 |
| cytochrome c | TRNA_RS40040 | BL14DL4_02014 | BLDA23_20030 | CK945_RS20380 | ACH97_216030 |
| YitT family protein | TRNA_RS40045 | BL14DL4_02015 | BLDA23_20035 | CK945_RS20385 | ACH97_216035 |
| protein translocase subunit SecA | TRNA_RS40055 | BL14DL4_02017 | BLDA23_20045 | CK945_RS20395 | ACH97_216045 |
| ribosome-associated translation inhibitor RaiA | TRNA_RS40060 | BL14DL4_02018 | BLDA23_20050 | CK945_RS20400 | ACH97_216050 |
| hypothetical protein | TRNA_RS40065 | BL14DL4_02019 | BLDA23_20055 | CK945_RS20405 | ACH97_216055 |
| flagellar protein FliT | TRNA_RS40070 | BL14DL4_02020 | BLDA23_20060 | CK945_RS20410 | ACH97_216060 |
| flagella export chaperone FliS | TRNA_RS40075 | BL14DL4_02021 | BLDA23_20065 | CK945_RS20415 | ACH97_216065 |
| flagellar hook-associated protein 2 | TRNA_RS40080 | BL14DL4_02022 | BLDA23_20070 | CK945_RS20420 | ACH97_216070 |
| flagellar protein FlaG | TRNA_RS40085 | BL14DL4_02023 | BLDA23_20075 | CK945_RS20425 | ACH97_216075 |
| flagellin | TRNA_RS40090 | BL14DL4_02024 | BLDA23_20080 | CK945_RS20435 | ACH97_216080 |
| carbon storage regulator | TRNA_RS40095 | BL14DL4_02025 | BLDA23_20085 | CK945_RS20440 | ACH97_216085 |
| flagellar assembly protein FliW | TRNA_RS40100 | BL14DL4_02026 | BLDA23_20090 | CK945_RS20445 | ACH97_216090 |
| hypothetical protein | TRNA_RS40105 | BL14DL4_02027 | BLDA23_20095 | CK945_RS20450 | ACH97_216095 |
| flagellar hook-associated protein FlgL | TRNA_RS40110 | BL14DL4_02028 | BLDA23_20100 | CK945_RS20455 | ACH97_216100 |
| flagellar hook-associated protein FlgK | TRNA_RS40115 | BL14DL4_02029 | BLDA23_20105 | CK945_RS20460 | ACH97_216105 |
| flagellar protein FlgN | TRNA_RS40120 | BL14DL4_02030 | BLDA23_20110 | CK945_RS20465 | ACH97_216110 |
| flagellar biosynthesis anti-sigma factor FlgM | TRNA_RS40125 | BL14DL4_02031 | BLDA23_20115 | CK945_RS20470 | ACH97_216115 |
| membrane protein | TRNA_RS40130 | BL14DL4_02032 | BLDA23_20120 | CK945_RS20475 | ACH97_216120 |
| competence protein ComFB | TRNA_RS40140 | BL14DL4_02033 | BLDA23_20130 | CK945_RS20485 | ACH97_216130 |
| DegV family protein | TRNA_RS40150 | BL14DL4_02035 | BLDA23_20140 | CK945_RS20495 | ACH97_216140 |
| DNA-binding response regulator | TRNA_RS40155 | BL14DL4_02036 | BLDA23_20145 | CK945_RS20500 | ACH97_216145 |
| histidine kinase | TRNA_RS40160 | BL14DL4_02037 | BLDA23_20150 | CK945_RS20505 | ACH97_216150 |
| YigZ family protein | TRNA_RS40165 | BL14DL4_02038 | BLDA23_20155 | CK945_RS20510 | ACH97_216155 |
| LytR family transcriptional regulator | TRNA_RS40170 | BL14DL4_02039 | BLDA23_20160 | CK945_RS20515 | ACH97_216160 |
| hypothetical protein | TRNA_RS40175 | BL14DL4_02040 | BLDA23_20165 | CK945_RS20520 | ACH97_216165 |
| undecaprenyl/decaprenyl-phosphate alpha-N-acetylglucosaminyl 1-phosphate transferase | TRNA_RS40180 | BL14DL4_02041 | BLDA23_20170 | CK945_RS20525 | ACH97_216170 |
| glycosyltransferase family 1 protein | TRNA_RS40185 | BL14DL4_02042 | BLDA23_20175 | CK945_RS20530 | ACH97_216175 |
| glycosyltransferase | TRNA_RS40190 | BL14DL4_02043 | BLDA23_20180 | CK945_RS20535 | ACH97_216180 |
| hypothetical protein | TRNA_RS40195 | BL14DL4_02044 | BLDA23_20185 | CK945_RS20540 | ACH97_216185 |
| O-antigen ligase domain-containing protein | TRNA_RS40200 | BL14DL4_02045 | BLDA23_20190 | CK945_RS20545 | ACH97_216190 |
| NAD-dependent epimerase/dehydratase family protein | TRNA_RS40205 | BL14DL4_02046 | BLDA23_20195 | CK945_RS20550 | ACH97_216195 |
| UDP-glucose/GDP-mannose dehydrogenase family protein | TRNA_RS40210 | BL14DL4_02047 | BLDA23_20200 | CK945_RS20555 | ACH97_216200 |
| glycosyltransferase family 4 protein | TRNA_RS40215 | BL14DL4_02048 | BLDA23_20205 | CK945_RS20560 | ACH97_216205 |
| colanic acid exporter | TRNA_RS40220 | BL14DL4_02049 | BLDA23_20210 | CK945_RS20565 | ACH97_216210 |
| sugar transferase | TRNA_RS40225 | BL14DL4_02050 | BLDA23_20215 | CK945_RS20570 | ACH97_216215 |
| N-acetylmuramoyl-L-alanine amidase | TRNA_RS40230 | BL14DL4_02051 | BLDA23_20220 | CK945_RS20575 | ACH97_216220 |
| SpoIID/LytB domain-containing protein | TRNA_RS40235 | BL14DL4_02052 | BLDA23_20225 | CK945_RS20580 | ACH97_216225 |
| hypothetical protein | TRNA_RS40240 | BL14DL4_02053 | BLDA23_20230 | CK945_RS20585 | ACH97_216230 |
| LytR family transcriptional regulator | TRNA_RS40245 | BL14DL4_02054 | BLDA23_20235 | CK945_RS20590 | ACH97_216235 |
| UDP-N-acetylglucosamine 2-epimerase (non-hydrolyzing) | TRNA_RS40250 | BL14DL4_02055 | BLDA23_20245 | CK945_RS20595 | ACH97_221985 |
| UTP--glucose-1-phosphate uridylyltransferase | TRNA_RS40255 | BL14DL4_02056 | BLDA23_20250 | CK945_RS20600 | ACH97_221990 |
| teichoic acids export ABC transporter ATP-binding subunit TagH | TRNA_RS40260 | BL14DL4_02057 | BLDA23_20255 | CK945_RS20630 | ACH97_214620 |
| teichoic acids export ABC transporter permease subunit TagG | TRNA_RS40265 | BL14DL4_02058 | BLDA23_20260 | CK945_RS20635 | ACH97_214615 |
| CDP-glycerol glycerophosphotransferase family protein | TRNA_RS40275 | BL14DL4_02061 | BLDA23_20280 | CK945_RS20645 | ACH97_214605 |
| glycerol-3-phosphate cytidylyltransferase | TRNA_RS40280 | BL14DL4_02062 | BLDA23_20285 | CK945_RS20650 | ACH97_214600 |
| glycosyltransferase | TRNA_RS40285 | BL14DL4_02063 | BLDA23_20290 | CK945_RS20655 | ACH97_214595 |
| CDP-glycerol--glycerophosphate glycerophosphotransferase | TRNA_RS40290 | BL14DL4_02064 | BLDA23_20295 | CK945_RS20660 | ACH97_214590 |
| beta-N-acetylglucosaminidase | TRNA_RS40295 | BL14DL4_02065 | BLDA23_20300 | CK945_RS20665 | ACH97_214585 |
| mannose-6-phosphate isomerase, class I | TRNA_RS40300 | BL14DL4_02066 | BLDA23_20305 | CK945_RS20670 | ACH97_214580 |
| anaerobic ribonucleoside-triphosphate reductase activating protein | TRNA_RS40305 | BL14DL4_02067 | BLDA23_20310 | CK945_RS20675 | ACH97_214575 |
| anaerobic ribonucleoside triphosphate reductase | TRNA_RS40310 | BL14DL4_02068 | BLDA23_20315 | CK945_RS20680 | ACH97_214570 |
| 2-keto-3-deoxygluconate transporter | TRNA_RS40315 | BL14DL4_02069 | BLDA23_20320 | CK945_RS20685 | ACH97_214565 |
| bifunctional 4-hydroxy-2-oxoglutarate aldolase/2-dehydro-3-deoxy-phosphogluconate aldolase | TRNA_RS40320 | BL14DL4_02070 | BLDA23_20325 | CK945_RS20690 | ACH97_214560 |
| sugar kinase | TRNA_RS40325 | BL14DL4_02071 | BLDA23_20330 | CK945_RS20695 | ACH97_214555 |
| LacI family DNA-binding transcriptional regulator | TRNA_RS40330 | BL14DL4_02072 | BLDA23_20335 | CK945_RS20700 | ACH97_214550 |
| 5-dehydro-4-deoxy-D-glucuronate isomerase | TRNA_RS40335 | BL14DL4_02073 | BLDA23_20340 | CK945_RS20705 | ACH97_214545 |
| 2-dehydro-3-deoxy-D-gluconate 5-dehydrogenase KduD | TRNA_RS40340 | BL14DL4_02074 | BLDA23_20345 | CK945_RS20710 | ACH97_214540 |
| glutathione-dependent formaldehyde dehydrogenase | TRNA_RS40345 | BL14DL4_02075 | BLDA23_20350 | CK945_RS20715 | ACH97_214535 |
| LytR family transcriptional regulator | TRNA_RS40355 | BL14DL4_02078 | BLDA23_20360 | CK945_RS20725 | ACH97_214525 |
| Cof-type HAD-IIB family hydrolase | TRNA_RS40360 | BL14DL4_02079 | BLDA23_20365 | CK945_RS20730 | ACH97_214520 |
| bifunctional murein DD-endopeptidase/murein LD-carboxypeptidase | TRNA_RS40365 | BL14DL4_02080 | BLDA23_20370 | CK945_RS20735 | ACH97_214515 |
| CapA family protein | TRNA_RS40370 | BL14DL4_02082 | BLDA23_20375 | CK945_RS20745 | ACH97_214510 |
| poly-gamma-glutamate biosynthesis protein PgsC | TRNA_RS40375 | BL14DL4_02083 | BLDA23_20380 | CK945_RS20750 | ACH97_214505 |
| poly-gamma-glutamate synthase PgsB | TRNA_RS40380 | BL14DL4_02084 | BLDA23_20385 | CK945_RS20755 | ACH97_214500 |
| LacI family transcriptional regulator | TRNA_RS40385 | BL14DL4_02085 | BLDA23_20390 | CK945_RS20760 | ACH97_214495 |
| ribokinase | TRNA_RS40390 | BL14DL4_02086 | BLDA23_20395 | CK945_RS20765 | ACH97_214490 |
| D-ribose pyranase | TRNA_RS40395 | BL14DL4_02087 | BLDA23_20400 | CK945_RS20770 | ACH97_214485 |
| sugar ABC transporter ATP-binding protein | TRNA_RS40400 | BL14DL4_02088 | BLDA23_20405 | CK945_RS20775 | ACH97_214480 |
| ribose ABC transporter permease | TRNA_RS40405 | BL14DL4_02089 | BLDA23_20410 | CK945_RS20780 | ACH97_214475 |
| ribose ABC transporter substrate-binding protein RbsB | TRNA_RS40410 | BL14DL4_02090 | BLDA23_20415 | CK945_RS20785 | ACH97_214470 |
| acetolactate decarboxylase | TRNA_RS40420 | BL14DL4_02091 | BLDA23_20425 | CK945_RS20800 | ACH97_214455 |
| acetolactate synthase AlsS | TRNA_RS40425 | BL14DL4_02092 | BLDA23_20430 | CK945_RS20805 | ACH97_214450 |
| gamma-glutamyltransferase | TRNA_RS40435 | BL14DL4_02094 | BLDA23_20445 | CK945_RS20815 | ACH97_214440 |
| Lrp/AsnC family transcriptional regulator | TRNA_RS40440 | BL14DL4_02095 | BLDA23_20450 | CK945_RS20820 | ACH97_214435 |
| chromate transporter | TRNA_RS40445 | BL14DL4_02096 | BLDA23_20455 | CK945_RS20825 | ACH97_214430 |
| chromate transporter | TRNA_RS40450 | BL14DL4_02097 | BLDA23_20460 | CK945_RS20830 | ACH97_214425 |
| tyrosine protein phosphatase | TRNA_RS40455 | BL14DL4_02098 | BLDA23_20465 | CK945_RS20835 | ACH97_214420 |
| modulator protein | TRNA_RS40460 | BL14DL4_02099 | BLDA23_20470 | CK945_RS20840 | ACH97_214415 |
| SWIM zinc finger family protein | TRNA_RS40465 | BL14DL4_02101 | BLDA23_20480 | CK945_RS20850 | ACH97_214410 |
| DEAD/DEAH box helicase | TRNA_RS40470 | BL14DL4_02102 | BLDA23_20485 | CK945_RS20855 | ACH97_214405 |
| Cof-type HAD-IIB family hydrolase | TRNA_RS40475 | BL14DL4_02104 | BLDA23_20490 | CK945_RS20880 | ACH97_214380 |
| DeoR/GlpR transcriptional regulator | TRNA_RS40480 | BL14DL4_02105 | BLDA23_20495 | CK945_RS20885 | ACH97_214375 |
| transaldolase | TRNA_RS40485 | BL14DL4_02106 | BLDA23_20500 | CK945_RS20890 | ACH97_214370 |
| sorbitol-6-phosphate dehydrogenase | TRNA_RS40490 | BL14DL4_02107 | BLDA23_20505 | CK945_RS20895 | ACH97_214365 |
| PTS sorbitol transporter subunit IIA | TRNA_RS40495 | BL14DL4_02108 | BLDA23_20510 | CK945_RS20900 | ACH97_214360 |
| PTS sorbitol transporter subunit IIB | TRNA_RS40500 | BL14DL4_02109 | BLDA23_20515 | CK945_RS20905 | ACH97_214355 |
| PTS sorbitol transporter subunit IIC | TRNA_RS40505 | BL14DL4_02110 | BLDA23_20520 | CK945_RS20910 | ACH97_214350 |
| hypothetical protein | TRNA_RS40510 | BL14DL4_02111 | BLDA23_20525 | CK945_RS20915 | ACH97_214345 |
| sugar-binding transcriptional regulator | TRNA_RS40515 | BL14DL4_02112 | BLDA23_20530 | CK945_RS20920 | ACH97_214340 |
| single-stranded DNA-binding protein | TRNA_RS40520 | BL14DL4_02113 | BLDA23_20535 | CK945_RS20925 | ACH97_214335 |
| hypothetical protein | TRNA_RS40525 | BL14DL4_02114 | BLDA23_20540 | CK945_RS20930 | ACH97_214330 |
| hypothetical protein | TRNA_RS40530 | BL14DL4_02115 | BLDA23_20545 | CK945_RS20935 | ACH97_214325 |
| large conductance mechanosensitive channel protein MscL | TRNA_RS40535 | BL14DL4_02116 | BLDA23_20550 | CK945_RS20940 | ACH97_214320 |
| beta-hydroxyacyl-ACP dehydratase | TRNA_RS40540 | BL14DL4_02117 | BLDA23_20555 | CK945_RS20945 | ACH97_214315 |
| flagellar hook-basal body protein | TRNA_RS40545 | BL14DL4_02118 | BLDA23_20560 | CK945_RS20950 | ACH97_214310 |
| flagellar hook-basal body protein | TRNA_RS40550 | BL14DL4_02119 | BLDA23_20565 | CK945_RS20955 | ACH97_214305 |
| rod shape-determining protein | TRNA_RS40555 | BL14DL4_02120 | BLDA23_20570 | CK945_RS20960 | ACH97_214300 |
| sporulation transcriptional regulator SpoIIID | TRNA_RS40560 | BL14DL4_02121 | BLDA23_20575 | CK945_RS20965 | ACH97_214295 |
| MarR family transcriptional regulator | TRNA_RS40565 | BL14DL4_02122 | BLDA23_20585 | CK945_RS20975 | ACH97_214290 |
| MFS transporter | TRNA_RS40570 | BL14DL4_02123 | BLDA23_20590 | CK945_RS20980 | ACH97_214285 |
| undecaprenyl-diphosphatase | TRNA_RS40575 | BL14DL4_02124 | BLDA23_20595 | CK945_RS20985 | ACH97_214280 |
| manganese-dependent inorganic pyrophosphatase | TRNA_RS40580 | BL14DL4_02125 | BLDA23_20600 | CK945_RS20990 | ACH97_214275 |
| manganese catalase | TRNA_RS40585 | BL14DL4_02126 | BLDA23_20605 | CK945_RS20995 | ACH97_214270 |
| DUF2071 domain-containing protein | TRNA_RS40590 | BL14DL4_02127 | BLDA23_20610 | CK945_RS21000 | ACH97_214265 |
| magnesium transporter | TRNA_RS40600 | BL14DL4_02129 | BLDA23_20620 | CK945_RS21020 | ACH97_214245 |
| HlyC/CorC family transporter | TRNA_RS40605 | BL14DL4_02130 | BLDA23_20625 | CK945_RS21025 | ACH97_214240 |
| ammonium transporter | TRNA_RS40610 | BL14DL4_02131 | BLDA23_20630 | CK945_RS21030 | ACH97_214235 |
| P-II family nitrogen regulator | TRNA_RS40615 | BL14DL4_02132 | BLDA23_20635 | CK945_RS21035 | ACH97_214230 |
| ATP-dependent Clp protease proteolytic subunit | TRNA_RS40620 | BL14DL4_02133 | BLDA23_20640 | CK945_RS21040 | ACH97_214225 |
| RNA polymerase sigma factor | TRNA_RS40625 | BL14DL4_02134 | BLDA23_20645 | CK945_RS21045 | ACH97_214220 |
| M23 family peptidase | TRNA_RS40630 | BL14DL4_02135 | BLDA23_20650 | CK945_RS21050 | ACH97_214215 |
| N-acetyltransferase | TRNA_RS40635 | BL14DL4_02136 | BLDA23_20655 | CK945_RS21055 | ACH97_214210 |
| hypothetical protein | TRNA_RS40640 | BL14DL4_02137 | BLDA23_20660 | CK945_RS21060 | ACH97_214205 |
| cardiolipin synthase | TRNA_RS40645 | BL14DL4_02138 | BLDA23_20665 | CK945_RS21065 | ACH97_214200 |
| DHA2 family efflux MFS transporter permease subunit | TRNA_RS40650 | BL14DL4_02140 | BLDA23_20675 | CK945_RS21070 | ACH97_214195 |
| non-ribosomal peptide synthetase | TRNA_RS40660 | BL14DL4_02142 | BLDA23_20685 | CK945_RS21080 | ACH97_214185 |
| isochorismatase family protein | TRNA_RS40665 | BL14DL4_02143 | BLDA23_20690 | CK945_RS21085 | ACH97_214180 |
| (2,3-dihydroxybenzoyl)adenylate synthase | TRNA_RS40670 | BL14DL4_02144 | BLDA23_20695 | CK945_RS21090 | ACH97_214175 |
| isochorismate synthase DhbC | TRNA_RS40675 | BL14DL4_02145 | BLDA23_20700 | CK945_RS21095 | ACH97_214170 |
| 2,3-dihydro-2,3-dihydroxybenzoate dehydrogenase | TRNA_RS40680 | BL14DL4_02146 | BLDA23_20705 | CK945_RS21100 | ACH97_214165 |
| alpha/beta hydrolase | TRNA_RS40685 | BL14DL4_02147 | BLDA23_20710 | CK945_RS21105 | ACH97_214160 |
| iron ABC transporter permease | TRNA_RS40690 | BL14DL4_02148 | BLDA23_20715 | CK945_RS21110 | ACH97_214155 |
| iron ABC transporter permease | TRNA_RS40695 | BL14DL4_02149 | BLDA23_20720 | CK945_RS21115 | ACH97_214150 |
| iron-hydroxamate ABC transporter substrate-binding protein | TRNA_RS40700 | BL14DL4_02150 | BLDA23_20725 | CK945_RS21120 | ACH97_214145 |
| helix-turn-helix domain-containing protein | TRNA_RS40705 | BL14DL4_02151 | BLDA23_20730 | CK945_RS21125 | ACH97_214140 |
| hypothetical protein | TRNA_RS40710 | BL14DL4_02152 | BLDA23_20735 | CK945_RS21130 | ACH97_214135 |
| Rrf2 family transcriptional regulator | TRNA_RS40730 | BL14DL4_02154 | BLDA23_20745 | CK945_RS21140 | ACH97_214125 |
| GTP 3,8-cyclase MoaA | TRNA_RS40740 | BL14DL4_02157 | BLDA23_20755 | CK945_RS21155 | ACH97_214110 |
| sulfurtransferase FdhD | TRNA_RS40745 | BL14DL4_02158 | BLDA23_20760 | CK945_RS21160 | ACH97_214105 |
| MFS transporter | TRNA_RS40750 | BL14DL4_02159 | BLDA23_20765 | CK945_RS21165 | ACH97_214100 |
| formate dehydrogenase | TRNA_RS40755 | BL14DL4_02160 | BLDA23_20770 | CK945_RS21170 | ACH97_214095 |
| VWA domain-containing protein | TRNA_RS40760 | BL14DL4_02161 | BLDA23_20775 | CK945_RS21175 | ACH97_214090 |
| VWA domain-containing protein | TRNA_RS40765 | BL14DL4_02162 | BLDA23_20780 | CK945_RS21180 | ACH97_214085 |
| stage II sporulation protein D | TRNA_RS40770 | BL14DL4_02163 | BLDA23_20785 | CK945_RS21185 | ACH97_214080 |
| hypothetical protein | TRNA_RS40780 | BL14DL4_02165 | BLDA23_20795 | CK945_RS21195 | ACH97_214070 |
| DUF1146 domain-containing protein | TRNA_RS40785 | BL14DL4_02166 | BLDA23_20800 | CK945_RS21200 | ACH97_214065 |
| F0F1 ATP synthase subunit epsilon | TRNA_RS40790 | BL14DL4_02167 | BLDA23_20805 | CK945_RS21205 | ACH97_214060 |
| F0F1 ATP synthase subunit beta | TRNA_RS40795 | BL14DL4_02168 | BLDA23_20810 | CK945_RS21210 | ACH97_214055 |
| ATP synthase subunit gamma | TRNA_RS40800 | BL14DL4_02169 | BLDA23_20815 | CK945_RS21215 | ACH97_214050 |
| ATP synthase subunit alpha | TRNA_RS40805 | BL14DL4_02170 | BLDA23_20820 | CK945_RS21220 | ACH97_214045 |
| F0F1 ATP synthase subunit delta | TRNA_RS40810 | BL14DL4_02171 | BLDA23_20825 | CK945_RS21225 | ACH97_214040 |
| ATP synthase subunit B | TRNA_RS40815 | BL14DL4_02172 | BLDA23_20830 | CK945_RS21230 | ACH97_214035 |
| F0F1 ATP synthase subunit C | TRNA_RS40820 | BL14DL4_02173 | BLDA23_20835 | CK945_RS21235 | ACH97_214030 |
| F0F1 ATP synthase subunit A | TRNA_RS40825 | BL14DL4_02174 | BLDA23_20840 | CK945_RS21240 | ACH97_214025 |
| ATP synthase subunit I | TRNA_RS40830 | BL14DL4_02175 | BLDA23_20845 | CK945_RS21245 | ACH97_214020 |
| uracil phosphoribosyltransferase | TRNA_RS40840 | BL14DL4_02176 | BLDA23_20855 | CK945_RS21255 | ACH97_214010 |
| serine hydroxymethyltransferase | TRNA_RS40845 | BL14DL4_02177 | BLDA23_20860 | CK945_RS21260 | ACH97_214005 |
| ribose 5-phosphate isomerase B | TRNA_RS40855 | BL14DL4_02179 | BLDA23_20870 | CK945_RS21270 | ACH97_213995 |
| low molecular weight protein arginine phosphatase | TRNA_RS40860 | BL14DL4_02180 | BLDA23_20875 | CK945_RS21275 | ACH97_213990 |
| manganese efflux pump | TRNA_RS40865 | BL14DL4_02181 | BLDA23_20880 | CK945_RS21280 | ACH97_213985 |
| threonylcarbamoyl-AMP synthase | TRNA_RS40870 | BL14DL4_02182 | BLDA23_20885 | CK945_RS21285 | ACH97_213980 |
| hypothetical protein | TRNA_RS40875 | BL14DL4_02183 | BLDA23_20890 | CK945_RS21290 | ACH97_213975 |
| stage II sporulation protein R | TRNA_RS40880 | BL14DL4_02184 | BLDA23_20895 | CK945_RS21295 | ACH97_213970 |
| MFS transporter | TRNA_RS40885 | BL14DL4_02185 | BLDA23_20900 | CK945_RS21300 | ACH97_213965 |
| gfo/Idh/MocA family oxidoreductase | TRNA_RS40890 | BL14DL4_02186 | BLDA23_20905 | CK945_RS21305 | ACH97_213960 |
| HAD-IIB family hydrolase | TRNA_RS40895 | BL14DL4_02187 | BLDA23_20910 | CK945_RS21310 | ACH97_213955 |
| LacI family DNA-binding transcriptional regulator | TRNA_RS40905 | BL14DL4_02189 | BLDA23_20920 | CK945_RS21320 | ACH97_213945 |
| peptide chain release factor N(5)-glutamine methyltransferase | TRNA_RS40910 | BL14DL4_02190 | BLDA23_20925 | CK945_RS21335 | ACH97_213930 |
| peptide chain release factor 1 | TRNA_RS40915 | BL14DL4_02191 | BLDA23_20930 | CK945_RS21340 | ACH97_213925 |
| VOC family protein | TRNA_RS40920 | BL14DL4_02192 | BLDA23_20935 | CK945_RS21345 | ACH97_213920 |
| chromosome-anchoring protein RacA | TRNA_RS40925 | BL14DL4_02193 | BLDA23_20940 | CK945_RS21350 | ACH97_213915 |
| AEC family transporter | TRNA_RS40930 | BL14DL4_02194 | BLDA23_20945 | CK945_RS21355 | ACH97_213910 |
| NAD-dependent malic enzyme | TRNA_RS40935 | BL14DL4_02195 | BLDA23_20950 | CK945_RS21360 | ACH97_213905 |
| thymidine kinase | TRNA_RS40940 | BL14DL4_02196 | BLDA23_20955 | CK945_RS21365 | ACH97_213900 |
| 50S ribosomal protein L31 | TRNA_RS40945 | BL14DL4_02197 | BLDA23_20960 | CK945_RS21370 | ACH97_213895 |
| transcription termination factor Rho | TRNA_RS40950 | BL14DL4_02198 | BLDA23_20965 | CK945_RS21375 | ACH97_213890 |
| class II fructose-bisphosphatase | TRNA_RS40955 | BL14DL4_02199 | BLDA23_20970 | CK945_RS21380 | ACH97_213885 |
| UDP-N-acetylglucosamine 1-carboxyvinyltransferase | TRNA_RS40960 | BL14DL4_02200 | BLDA23_20975 | CK945_RS21385 | ACH97_213880 |
| fructose-6-phosphate aldolase | TRNA_RS40965 | BL14DL4_02201 | BLDA23_20980 | CK945_RS21390 | ACH97_213875 |
| fructose-bisphosphate aldolase | TRNA_RS40970 | BL14DL4_02202 | BLDA23_20985 | CK945_RS21395 | ACH97_213870 |
| response regulator | TRNA_RS40975 | BL14DL4_02203 | BLDA23_20990 | CK945_RS21400 | ACH97_213865 |
| DUF2529 family protein | TRNA_RS40980 | BL14DL4_02204 | BLDA23_20995 | CK945_RS21405 | ACH97_213860 |
| CTP synthetase | TRNA_RS40985 | BL14DL4_02205 | BLDA23_21005 | CK945_RS21410 | ACH97_213855 |
| DNA-directed RNA polymerase subunit delta | TRNA_RS40990 | BL14DL4_02206 | BLDA23_21010 | CK945_RS21415 | ACH97_213850 |
| acyl-CoA dehydrogenase | TRNA_RS40995 | BL14DL4_02207 | BLDA23_21015 | CK945_RS21420 | ACH97_213845 |
| acyl-CoA dehydrogenase | TRNA_RS41000 | BL14DL4_02208 | BLDA23_21020 | CK945_RS21425 | ACH97_213840 |
| 3-hydroxybutyryl-CoA dehydrogenase | TRNA_RS41005 | BL14DL4_02209 | BLDA23_21025 | CK945_RS21430 | ACH97_213835 |
| acetyl-CoA C-acetyltransferase | TRNA_RS41010 | BL14DL4_02210 | BLDA23_21030 | CK945_RS21435 | ACH97_213830 |
| 4Fe-4S dicluster domain-containing protein | TRNA_RS41015 | BL14DL4_02211 | BLDA23_21035 | CK945_RS21440 | ACH97_213825 |
| cardiolipin synthase | TRNA_RS41020 | BL14DL4_02212 | BLDA23_21040 | CK945_RS21445 | ACH97_213820 |
| hypothetical protein | TRNA_RS41025 | BL14DL4_02213 | BLDA23_21045 | CK945_RS21450 | ACH97_213815 |
| arginine--tRNA ligase | TRNA_RS41030 | BL14DL4_02214 | BLDA23_21050 | CK945_RS21455 | ACH97_213810 |
| DUF1934 family protein | TRNA_RS41035 | BL14DL4_02215 | BLDA23_21055 | CK945_RS21460 | ACH97_213805 |
| agmatinase | TRNA_RS41040 | BL14DL4_02216 | BLDA23_21060 | CK945_RS21465 | ACH97_213800 |
| spermidine synthase | TRNA_RS41045 | BL14DL4_02217 | BLDA23_21065 | CK945_RS21470 | ACH97_213795 |
| penicillin-binding protein | TRNA_RS41060 | BL14DL4_02219 | BLDA23_21080 | CK945_RS21475 | ACH97_213790 |
| hypothetical protein | TRNA_RS41065 | BL14DL4_02220 | BLDA23_21085 | CK945_RS21480 | ACH97_213785 |
| site-2 protease family protein | TRNA_RS41070 | BL14DL4_02221 | BLDA23_21090 | CK945_RS21485 | ACH97_213780 |
| 4-oxalocrotonate tautomerase | TRNA_RS41075 | BL14DL4_02222 | BLDA23_21095 | CK945_RS21490 | ACH97_213775 |
| peptidase M84 | TRNA_RS41080 | BL14DL4_02223 | BLDA23_21100 | CK945_RS21495 | ACH97_213770 |
| YwgA family protein | TRNA_RS41085 | BL14DL4_02224 | BLDA23_21105 | CK945_RS21500 | ACH97_213765 |
| HD domain-containing protein | TRNA_RS41090 | BL14DL4_02225 | BLDA23_21110 | CK945_RS21505 | ACH97_213760 |
| DUF1450 domain-containing protein | TRNA_RS41095 | BL14DL4_02226 | BLDA23_21115 | CK945_RS21510 | ACH97_213755 |
| RsfA family transcriptional regulator | TRNA_RS41100 | BL14DL4_02227 | BLDA23_21120 | CK945_RS21515 | ACH97_213750 |
| DUF4306 domain-containing protein | TRNA_RS41105 | BL14DL4_02228 | BLDA23_21125 | CK945_RS21520 | ACH97_213745 |
| lipoate--protein ligase family protein | TRNA_RS41110 | BL14DL4_02229 | BLDA23_21130 | CK945_RS21525 | ACH97_213740 |
| 4-hydroxybenzoate 3-monooxygenase | TRNA_RS41115 | BL14DL4_02230 | BLDA23_21135 | CK945_RS21530 | ACH97_213735 |
| MFS transporter | TRNA_RS41120 | BL14DL4_02231 | BLDA23_21140 | CK945_RS21535 | ACH97_213730 |
| extradiol ring-cleavage dioxygenase | TRNA_RS41125 | BL14DL4_02232 | BLDA23_21145 | CK945_RS21540 | ACH97_213725 |
| 4-oxalocrotonate tautomerase | TRNA_RS41130 | BL14DL4_02233 | BLDA23_21150 | CK945_RS21545 | ACH97_213720 |
| 4-oxalocrotonate decarboxylase | TRNA_RS41135 | BL14DL4_02234 | BLDA23_21155 | CK945_RS21550 | ACH97_213715 |
| aldehyde dehydrogenase family protein | TRNA_RS41140 | BL14DL4_02235 | BLDA23_21160 | CK945_RS21555 | ACH97_213710 |
| IclR family transcriptional regulator | TRNA_RS41145 | BL14DL4_02236 | BLDA23_21165 | CK945_RS21560 | ACH97_213705 |
| amidohydrolase | TRNA_RS41150 | BL14DL4_02237 | BLDA23_21170 | CK945_RS21565 | ACH97_213700 |
| phosphate acetyltransferase | TRNA_RS41155 | BL14DL4_02238 | BLDA23_21175 | CK945_RS21570 | ACH97_213695 |
| heme-binding protein | TRNA_RS41160 | BL14DL4_02239 | BLDA23_21180 | CK945_RS21575 | ACH97_213690 |
| hypothetical protein | TRNA_RS41165 | BL14DL4_02240 | BLDA23_21185 | CK945_RS21580 | ACH97_213685 |
| spore coat protein GerQ | TRNA_RS41180 | BL14DL4_02242 | BLDA23_21200 | CK945_RS21595 | ACH97_213675 |
| ABC transporter permease | TRNA_RS41185 | BL14DL4_02243 | BLDA23_21205 | CK945_RS21600 | ACH97_213670 |
| ABC transporter permease | TRNA_RS41190 | BL14DL4_02244 | BLDA23_21210 | CK945_RS21605 | ACH97_213665 |
| ABC transporter ATP-binding protein | TRNA_RS41195 | BL14DL4_02245 | BLDA23_21215 | CK945_RS21610 | ACH97_213660 |
| two-component sensor histidine kinase | TRNA_RS41200 | BL14DL4_02246 | BLDA23_21220 | CK945_RS21615 | ACH97_213655 |
| DNA-binding response regulator | TRNA_RS41205 | BL14DL4_02247 | BLDA23_21225 | CK945_RS21620 | ACH97_213650 |
| DUF423 domain-containing protein | TRNA_RS41210 | BL14DL4_02248 | BLDA23_21230 | CK945_RS21625 | ACH97_213645 |
| purine permease | TRNA_RS41215 | BL14DL4_02249 | BLDA23_21235 | CK945_RS21630 | ACH97_213640 |
| hypothetical protein | TRNA_RS41220 | BL14DL4_02250 | BLDA23_21240 | CK945_RS21635 | ACH97_213635 |
| aldehyde dehydrogenase | TRNA_RS41225 | BL14DL4_02251 | BLDA23_21245 | CK945_RS21640 | ACH97_213630 |
| uracil-DNA glycosylase | TRNA_RS41230 | BL14DL4_02252 | BLDA23_21250 | CK945_RS21645 | ACH97_213625 |
| glycosyltransferase family 2 protein | TRNA_RS41235 | BL14DL4_02253 | BLDA23_21255 | CK945_RS21650 | ACH97_213620 |
| bifunctional hydroxymethylpyrimidine kinase/phosphomethylpyrimidine kinase | TRNA_RS41240 | BL14DL4_02254 | BLDA23_21260 | CK945_RS21655 | ACH97_213615 |
| sucrose-6-phosphate hydrolase | TRNA_RS41250 | BL14DL4_02256 | BLDA23_21270 | CK945_RS21665 | ACH97_213605 |
| PTS sugar transporter | TRNA_RS41255 | BL14DL4_02257 | BLDA23_21275 | CK945_RS21670 | ACH97_213600 |
| PRD domain-containing protein | TRNA_RS41260 | BL14DL4_02258 | BLDA23_21280 | CK945_RS21675 | ACH97_213595 |
| hypothetical protein | TRNA_RS41265 | BL14DL4_02259 | BLDA23_21285 | CK945_RS21680 | ACH97_213590 |
| peptidase S8 | TRNA_RS41270 | BL14DL4_02261 | BLDA23_21290 | CK945_RS21685 | ACH97_213585 |
| hypothetical protein | TRNA_RS41275 | BL14DL4_02262 | BLDA23_21295 | CK945_RS21690 | ACH97_213580 |
| LLM class flavin-dependent oxidoreductase | TRNA_RS41280 | BL14DL4_02263 | BLDA23_21300 | CK945_RS21695 | ACH97_213575 |
| oxygen-insensitive NADPH nitroreductase | TRNA_RS41285 | BL14DL4_02265 | BLDA23_21305 | CK945_RS21700 | ACH97_213570 |
| amidohydrolase | TRNA_RS41290 | BL14DL4_02266 | BLDA23_21310 | CK945_RS21705 | ACH97_213565 |
| rod shape-determining protein RodA | TRNA_RS41295 | BL14DL4_02267 | BLDA23_21315 | CK945_RS21710 | ACH97_213560 |
| FtsW/RodA/SpoVE family cell cycle protein | TRNA_RS41300 | BL14DL4_02268 | BLDA23_21320 | CK945_RS21715 | ACH97_213555 |
| YjiH family protein | TRNA_RS41305 | BL14DL4_02269 | BLDA23_21325 | CK945_RS21720 | ACH97_213550 |
| germination protein | TRNA_RS41315 | BL14DL4_02271 | BLDA23_21335 | CK945_RS21725 | ACH97_213545 |
| hypothetical protein | TRNA_RS41320 | BL14DL4_02272 | BLDA23_21340 | CK945_RS21730 | ACH97_213540 |
| GNAT family N-acetyltransferase | TRNA_RS41325 | BL14DL4_02273 | BLDA23_21345 | CK945_RS21735 | ACH97_213535 |
| ABC transporter ATP-binding protein | TRNA_RS41330 | BL14DL4_02274 | BLDA23_21350 | CK945_RS21740 | ACH97_213530 |
| ABC transporter ATP-binding protein | TRNA_RS41335 | BL14DL4_02275 | BLDA23_21355 | CK945_RS21745 | ACH97_213525 |
| lasso peptide biosynthesis B2 protein | TRNA_RS41345 | BL14DL4_02277 | BLDA23_21365 | CK945_RS21755 | ACH97_213515 |
| hypothetical protein | TRNA_RS41350 | BL14DL4_02278 | BLDA23_21370 | CK945_RS21760 | ACH97_213510 |
| DNA-binding response regulator | TRNA_RS41355 | BL14DL4_02279 | BLDA23_21375 | CK945_RS21765 | ACH97_213505 |
| cytochrome aa3 quinol oxidase subunit IV | TRNA_RS41360 | BL14DL4_02281 | BLDA23_21380 | CK945_RS21770 | ACH97_213500 |
| cytochrome aa3 quinol oxidase subunit III | TRNA_RS41365 | BL14DL4_02282 | BLDA23_21385 | CK945_RS21775 | ACH97_213495 |
| cytochrome aa3 quinol oxidase subunit I | TRNA_RS41370 | BL14DL4_02283 | BLDA23_21390 | CK945_RS21780 | ACH97_213490 |
| cytochrome aa3 quinol oxidase subunit II | TRNA_RS41375 | BL14DL4_02284 | BLDA23_21395 | CK945_RS21785 | ACH97_213485 |
| UDP-glucose 4-epimerase GalE | TRNA_RS41380 | BL14DL4_02285 | BLDA23_21400 | CK945_RS21790 | ACH97_213480 |
| DNA-binding anti-repressor SinI | TRNA_RS41385 | BL14DL4_02286 | BLDA23_21405 | CK945_RS21795 | ACH97_213475 |
| cation acetate symporter | TRNA_RS41405 | BL14DL4_02291 | BLDA23_21425 | CK945_RS21815 | ACH97_213455 |
| xylose isomerase | TRNA_RS41415 | BL14DL4_02293 | BLDA23_21435 | CK945_RS21825 | ACH97_213445 |
| ROK family transcriptional regulator | TRNA_RS41420 | BL14DL4_02294 | BLDA23_21440 | CK945_RS21830 | ACH97_213440 |
| thiamine phosphate synthase | TRNA_RS41425 | BL14DL4_02295 | BLDA23_21445 | CK945_RS21835 | ACH97_213435 |
| hydroxyethylthiazole kinase | TRNA_RS41430 | BL14DL4_02296 | BLDA23_21450 | CK945_RS21840 | ACH97_213430 |
| hypothetical protein | TRNA_RS41440 | BL14DL4_02298 | BLDA23_21460 | CK945_RS21850 | ACH97_213420 |
| DUF5082 domain-containing protein | TRNA_RS41445 | BL14DL4_02299 | BLDA23_21465 | CK945_RS21855 | ACH97_213415 |
| CidA/LrgA family holin-like protein | TRNA_RS41450 | BL14DL4_02300 | BLDA23_21470 | CK945_RS21860 | ACH97_213410 |
| CidB/LrgB family autolysis modulator | TRNA_RS41455 | BL14DL4_02301 | BLDA23_21475 | CK945_RS21865 | ACH97_213405 |
| hypothetical protein | TRNA_RS41460 | BL14DL4_02302 | BLDA23_21480 | CK945_RS21870 | ACH97_213400 |
| sodium/proline symporter PutP | TRNA_RS41465 | BL14DL4_02303 | BLDA23_21485 | CK945_RS21875 | ACH97_213395 |
| class I SAM-dependent rRNA methyltransferase | TRNA_RS41470 | BL14DL4_02304 | BLDA23_21490 | CK945_RS21880 | ACH97_213390 |
| VOC family protein | TRNA_RS41475 | BL14DL4_02305 | BLDA23_21495 | CK945_RS21885 | ACH97_213385 |
| glycosyltransferase family 8 protein | TRNA_RS41485 | BL14DL4_02307 | BLDA23_21505 | CK945_RS21895 | ACH97_213375 |
| sensor histidine kinase | TRNA_RS41495 | BL14DL4_02310 | BLDA23_21515 | CK945_RS21910 | ACH97_213370 |
| DNA-binding response regulator | TRNA_RS41500 | BL14DL4_02311 | BLDA23_21520 | CK945_RS21915 | ACH97_213365 |
| MMPL family transporter | TRNA_RS41505 | BL14DL4_02312 | BLDA23_21525 | CK945_RS21920 | ACH97_213360 |
| TIGR02206 family membrane protein | TRNA_RS41510 | BL14DL4_02313 | BLDA23_21530 | CK945_RS21925 | ACH97_213355 |
| MFS transporter | TRNA_RS41515 | BL14DL4_02314 | BLDA23_21535 | CK945_RS21930 | ACH97_213350 |
| galactonate dehydratase | TRNA_RS41520 | BL14DL4_02315 | BLDA23_21540 | CK945_RS21935 | ACH97_213345 |
| bifunctional 4-hydroxy-2-oxoglutarate aldolase/2-dehydro-3-deoxy-phosphogluconate aldolase | TRNA_RS41525 | BL14DL4_02316 | BLDA23_21545 | CK945_RS21940 | ACH97_213340 |
| sugar kinase | TRNA_RS41530 | BL14DL4_02317 | BLDA23_21550 | CK945_RS21945 | ACH97_213335 |
| IclR family transcriptional regulator | TRNA_RS41535 | BL14DL4_02318 | BLDA23_21555 | CK945_RS21950 | ACH97_213330 |
| aminopeptidase | TRNA_RS41540 | BL14DL4_02319 | BLDA23_21560 | CK945_RS21955 | ACH97_213325 |
| MFS transporter | TRNA_RS41545 | BL14DL4_02320 | BLDA23_21565 | CK945_RS21965 | ACH97_213320 |
| hypothetical protein | TRNA_RS41550 | BL14DL4_02321 | BLDA23_21570 | CK945_RS21970 | ACH97_213315 |
| GTP pyrophosphokinase family protein | TRNA_RS41555 | BL14DL4_02322 | BLDA23_21575 | CK945_RS21980 | ACH97_213305 |
| teichoic acid D-Ala incorporation-associated protein DltX | TRNA_RS41565 | BL14DL4_02324 | BLDA23_21585 | CK945_RS21990 | ACH97_213300 |
| D-alanine--poly(phosphoribitol) ligase | TRNA_RS41570 | BL14DL4_02325 | BLDA23_21590 | CK945_RS21995 | ACH97_213295 |
| D-alanyl-lipoteichoic acid biosynthesis protein DltB | TRNA_RS41575 | BL14DL4_02326 | BLDA23_21595 | CK945_RS22000 | ACH97_213290 |
| D-alanine--poly(phosphoribitol) ligase subunit 2 | TRNA_RS41580 | BL14DL4_02327 | BLDA23_21600 | CK945_RS22005 | ACH97_213285 |
| D-alanyl-lipoteichoic acid biosynthesis protein DltD | TRNA_RS41585 | BL14DL4_02328 | BLDA23_21605 | CK945_RS22010 | ACH97_213280 |
| branched-chain amino acid aminotransferase | TRNA_RS41590 | BL14DL4_02329 | BLDA23_21610 | CK945_RS22015 | ACH97_213275 |
| MFS transporter | TRNA_RS41595 | BL14DL4_02330 | BLDA23_21615 | CK945_RS22020 | ACH97_213270 |
| 6-phospho-beta-glucosidase | TRNA_RS41600 | BL14DL4_02331 | BLDA23_21620 | CK945_RS22025 | ACH97_213265 |
| PTS lactose/cellobiose transporter subunit IIA | TRNA_RS41605 | BL14DL4_02332 | BLDA23_21625 | CK945_RS22030 | ACH97_213260 |
| PTS cellobiose transporter subunit IIC | TRNA_RS41610 | BL14DL4_02333 | BLDA23_21630 | CK945_RS22035 | ACH97_213255 |
| PTS sugar transporter subunit IIB | TRNA_RS41615 | BL14DL4_02334 | BLDA23_21635 | CK945_RS22040 | ACH97_213250 |
| DNA-3-methyladenine glycosylase | TRNA_RS41625 | BL14DL4_02337 | BLDA23_21645 | CK945_RS22050 | ACH97_213240 |
| GntR family transcriptional regulator | TRNA_RS41630 | BL14DL4_02338 | BLDA23_21650 | CK945_RS22055 | ACH97_213235 |
| citrate synthase | TRNA_RS41635 | BL14DL4_02339 | BLDA23_21655 | CK945_RS22060 | ACH97_213230 |
| bifunctional 2-methylcitrate dehydratase/aconitate hydratase | TRNA_RS41640 | BL14DL4_02340 | BLDA23_21660 | CK945_RS22065 | ACH97_213225 |
| methylisocitrate lyase | TRNA_RS41645 | BL14DL4_02341 | BLDA23_21665 | CK945_RS22070 | ACH97_213220 |
| chromate transporter | TRNA_RS41650 | BL14DL4_02342 | BLDA23_21670 | CK945_RS22075 | ACH97_213215 |
| PadR family transcriptional regulator | TRNA_RS41655 | BL14DL4_02343 | BLDA23_21675 | CK945_RS22080 | ACH97_213210 |
| ABC transporter ATP-binding protein | TRNA_RS41660 | BL14DL4_02344 | BLDA23_21680 | CK945_RS22085 | ACH97_213205 |
| rhamnogalacturonan acetylesterase | TRNA_RS41665 | BL14DL4_02345 | BLDA23_21685 | CK945_RS22090 | ACH97_213200 |
| hypothetical protein | TRNA_RS41670 | BL14DL4_02346 | BLDA23_21690 | CK945_RS22095 | ACH97_213195 |
| hypothetical protein | TRNA_RS41675 | BL14DL4_02347 | BLDA23_21695 | CK945_RS22100 | ACH97_213190 |
| MFS transporter | TRNA_RS41680 | BL14DL4_02348 | BLDA23_21700 | CK945_RS22105 | ACH97_213185 |
| membrane protein | TRNA_RS41685 | BL14DL4_02349 | BLDA23_21705 | CK945_RS22110 | ACH97_213180 |
| ABC transporter ATP-binding protein | TRNA_RS41690 | BL14DL4_02350 | BLDA23_21710 | CK945_RS22115 | ACH97_213175 |
| hypothetical protein | TRNA_RS41695 | BL14DL4_02351 | BLDA23_21715 | CK945_RS22120 | ACH97_213170 |
| hypothetical protein | TRNA_RS41700 | BL14DL4_02352 | BLDA23_21720 | CK945_RS22125 | ACH97_213165 |
| hypothetical protein | TRNA_RS41705 | BL14DL4_02353 | BLDA23_21725 | CK945_RS22130 | ACH97_213160 |
| RNA polymerase sigma factor SigY | TRNA_RS41710 | BL14DL4_02354 | BLDA23_21730 | CK945_RS22135 | ACH97_213155 |
| MFS transporter | TRNA_RS41715 | BL14DL4_02355 | BLDA23_21735 | CK945_RS22140 | ACH97_213150 |
| catalase | TRNA_RS41720 | BL14DL4_02356 | BLDA23_21740 | CK945_RS22150 | ACH97_213140 |
| catalase | TRNA_RS41725 | BL14DL4_02357 | BLDA23_21745 | CK945_RS22155 | ACH97_213135 |
| transcriptional repressor | TRNA_RS41730 | BL14DL4_02358 | BLDA23_21750 | CK945_RS22160 | ACH97_213130 |
| ferrochelatase | TRNA_RS41735 | BL14DL4_02359 | BLDA23_21755 | CK945_RS22165 | ACH97_213125 |
| transcriptional regulator | TRNA_RS41770 | BL14DL4_02366 | BLDA23_21790 | CK945_RS00970 | ACH97_220280 |
| peptidase domain-containing ABC transporter | TRNA_RS41785 | BL14DL4_02369 | BLDA23_21805 | CK945_RS00995 | ACH97_220255 |
| type 2 lantibiotic | TRNA_RS41795 | BL14DL4_02371 | BLDA23_21815 | CK945_RS01010 | ACH97_220245 |
| plantaricin C family lantibiotic | TRNA_RS41800 | BL14DL4_02372 | BLDA23_21820 | CK945_RS01020 | ACH97_220235 |
| pectate trisaccharide-lyase | TRNA_RS41810 | BL14DL4_02374 | BLDA23_21830 | CK945_RS22170 | ACH97_213120 |
| NAD(P)H-hydrate dehydratase | TRNA_RS41815 | BL14DL4_02375 | BLDA23_21835 | CK945_RS22175 | ACH97_213115 |
| thiol reductant ABC exporter subunit CydC | TRNA_RS41820 | BL14DL4_02376 | BLDA23_21840 | CK945_RS22180 | ACH97_213110 |
| thiol reductant ABC exporter subunit CydD | TRNA_RS41825 | BL14DL4_02377 | BLDA23_21845 | CK945_RS22185 | ACH97_213105 |
| cytochrome d ubiquinol oxidase subunit II | TRNA_RS41830 | BL14DL4_02378 | BLDA23_21850 | CK945_RS22190 | ACH97_213100 |
| cytochrome ubiquinol oxidase subunit I | TRNA_RS41835 | BL14DL4_02379 | BLDA23_21855 | CK945_RS22195 | ACH97_213095 |
| transporter | TRNA_RS41845 | BL14DL4_02380 | BLDA23_21865 | CK945_RS22205 | ACH97_213085 |
| NAD-dependent malic enzyme | TRNA_RS41855 | BL14DL4_02382 | BLDA23_21875 | CK945_RS22215 | ACH97_213075 |
| Na+/H+ antiporter NhaC | TRNA_RS41860 | BL14DL4_02383 | BLDA23_21880 | CK945_RS22220 | ACH97_213070 |
| aspartate ammonia-lyase | TRNA_RS41865 | BL14DL4_02384 | BLDA23_21885 | CK945_RS22225 | ACH97_213065 |
| asparaginase | TRNA_RS41870 | BL14DL4_02385 | BLDA23_21890 | CK945_RS22230 | ACH97_213060 |
| XRE family transcriptional regulator | TRNA_RS41875 | BL14DL4_02386 | BLDA23_21895 | CK945_RS22235 | ACH97_213055 |
| lactonase family protein | TRNA_RS41880 | BL14DL4_02387 | BLDA23_21900 | CK945_RS22240 | ACH97_213050 |
| sensor histidine kinase | TRNA_RS41890 | BL14DL4_02389 | BLDA23_21910 | CK945_RS22250 | ACH97_213040 |
| ABC transporter ATP-binding protein | TRNA_RS41900 | BL14DL4_02390 | BLDA23_21920 | CK945_RS22255 | ACH97_213035 |
| ABC transporter permease | TRNA_RS41905 | BL14DL4_02391 | BLDA23_21925 | CK945_RS22260 | ACH97_213030 |
| YxeA family protein | TRNA_RS41910 | BL14DL4_02392 | BLDA23_21930 | CK945_RS22265 | ACH97_213025 |
| hypothetical protein | TRNA_RS41915 | BL14DL4_02394 | BLDA23_21935 | CK945_RS22275 | ACH97_213015 |
| polysaccharide deacetylase family protein | TRNA_RS41920 | BL14DL4_02395 | BLDA23_21940 | CK945_RS22280 | ACH97_213010 |
| glycosyltransferase | TRNA_RS41930 | BL14DL4_02397 | BLDA23_21955 | CK945_RS22295 | ACH97_212995 |
| L-malate permease | TRNA_RS41935 | BL14DL4_02398 | BLDA23_21965 | CK945_RS22300 | ACH97_212990 |
| glycerophosphoryl diester phosphodiesterase | TRNA_RS41940 | BL14DL4_02399 | BLDA23_21970 | CK945_RS22310 | ACH97_212985 |
| hypothetical protein | TRNA_RS41945 | BL14DL4_02400 | BLDA23_21975 | CK945_RS22315 | ACH97_212980 |
| hypothetical protein | TRNA_RS41950 | BL14DL4_02401 | BLDA23_21980 | CK945_RS22320 | ACH97_212975 |
| LTA synthase family protein | TRNA_RS41955 | BL14DL4_02402 | BLDA23_21985 | CK945_RS22325 | ACH97_212970 |
| hypothetical protein | TRNA_RS41960 | BL14DL4_02403 | BLDA23_21990 | CK945_RS22330 | ACH97_212965 |
| YitT family protein | TRNA_RS41965 | BL14DL4_02404 | BLDA23_21995 | CK945_RS22335 | ACH97_212960 |
| arginine repressor | TRNA_RS41970 | BL14DL4_02405 | BLDA23_22005 | CK945_RS22340 | ACH97_212955 |
| arginine deiminase | TRNA_RS41980 | BL14DL4_02406 | BLDA23_22015 | CK945_RS22350 | ACH97_212950 |
| ornithine carbamoyltransferase | TRNA_RS41985 | BL14DL4_02407 | BLDA23_22020 | CK945_RS22355 | ACH97_212945 |
| arginine-ornithine antiporter | TRNA_RS41990 | BL14DL4_02408 | BLDA23_22025 | CK945_RS22360 | ACH97_212940 |
| carbamate kinase | TRNA_RS41995 | BL14DL4_02409 | BLDA23_22030 | CK945_RS22365 | ACH97_212935 |
| Crp/Fnr family transcriptional regulator | TRNA_RS42000 | BL14DL4_02410 | BLDA23_22035 | CK945_RS22370 | ACH97_212930 |
| glycoside hydrolase family 1 protein | TRNA_RS42005 | BL14DL4_02411 | BLDA23_22040 | CK945_RS22375 | ACH97_212925 |
| PTS sugar transporter subunit IIC | TRNA_RS42010 | BL14DL4_02412 | BLDA23_22045 | CK945_RS22380 | ACH97_212920 |
| DUF4179 domain-containing protein | TRNA_RS42015 | BL14DL4_02413 | BLDA23_22050 | CK945_RS22385 | ACH97_212915 |
| sigma-70 family RNA polymerase sigma factor | TRNA_RS42020 | BL14DL4_02414 | BLDA23_22055 | CK945_RS22390 | ACH97_212910 |
| peptidase T | TRNA_RS42050 | BL14DL4_02416 | BLDA23_22085 | CK945_RS22400 | ACH97_212900 |
| glycoside hydrolase family 32 protein | TRNA_RS42060 | BL14DL4_02417 | BLDA23_22095 | CK945_RS22405 | ACH97_212895 |
| MFS transporter | TRNA_RS42065 | BL14DL4_02418 | BLDA23_22100 | CK945_RS22410 | ACH97_212890 |
| LacI family transcriptional regulator | TRNA_RS42085 | BL14DL4_02419 | BLDA23_22110 | CK945_RS22430 | ACH97_212870 |
| DUF98 domain-containing protein | TRNA_RS42090 | BL14DL4_02420 | BLDA23_22115 | CK945_RS22435 | ACH97_212865 |
| DUF3298/DUF4163 domain-containing protein | TRNA_RS42095 | BL14DL4_02421 | BLDA23_22120 | CK945_RS22440 | ACH97_212860 |
| 5-methyltetrahydropteroyltriglutamate-- homocysteine S-methyltransferase | TRNA_RS42100 | BL14DL4_02423 | BLDA23_22125 | CK945_RS22445 | ACH97_212855 |
| glycosyltransferase family 39 protein | TRNA_RS42105 | BL14DL4_02424 | BLDA23_22130 | CK945_RS22450 | ACH97_212850 |
| glycosyltransferase | TRNA_RS42110 | BL14DL4_02425 | BLDA23_22135 | CK945_RS22455 | ACH97_212845 |
| UDP-glucose 4-epimerase GalE | TRNA_RS42115 | BL14DL4_02426 | BLDA23_22140 | CK945_RS22460 | ACH97_212840 |
| hypothetical protein | TRNA_RS42120 | BL14DL4_02427 | BLDA23_22145 | CK945_RS22465 | ACH97_212835 |
| alpha/beta hydrolase | TRNA_RS42125 | BL14DL4_02428 | BLDA23_22150 | CK945_RS22470 | ACH97_212830 |
| DUF4176 domain-containing protein | TRNA_RS42135 | BL14DL4_02430 | BLDA23_22160 | CK945_RS22480 | ACH97_212820 |
| purine nucleoside transporter | TRNA_RS42140 | BL14DL4_02431 | BLDA23_22165 | CK945_RS22490 | ACH97_212810 |
| DNA gyrase inhibitor | TRNA_RS42150 | BL14DL4_02433 | BLDA23_22175 | CK945_RS22500 | ACH97_212795 |
| glycoside hydrolase family 1 protein | TRNA_RS42155 | BL14DL4_02434 | BLDA23_22180 | CK945_RS22505 | ACH97_212790 |
| PTS beta-glucoside transporter subunit IIBCA | TRNA_RS42160 | BL14DL4_02435 | BLDA23_22185 | CK945_RS22510 | ACH97_212785 |
| PRD domain-containing protein | TRNA_RS42165 | BL14DL4_02436 | BLDA23_22190 | CK945_RS22515 | ACH97_212780 |
| energy-coupling factor transporter transmembrane protein EcfT | TRNA_RS42170 | BL14DL4_02437 | BLDA23_22195 | CK945_RS22520 | ACH97_212775 |
| ABC transporter ATP-binding protein | TRNA_RS42175 | BL14DL4_02438 | BLDA23_22200 | CK945_RS22525 | ACH97_212770 |
| ABC transporter ATP-binding protein | TRNA_RS42180 | BL14DL4_02439 | BLDA23_22205 | CK945_RS22530 | ACH97_212765 |
| thiaminase II | TRNA_RS42185 | BL14DL4_02440 | BLDA23_22210 | CK945_RS22535 | ACH97_212760 |
| isocitrate lyase | TRNA_RS42190 | BL14DL4_02442 | BLDA23_22215 | CK945_RS22540 | ACH97_212755 |
| malate synthase A | TRNA_RS42195 | BL14DL4_02443 | BLDA23_22220 | CK945_RS22545 | ACH97_212750 |
| phospholipid carrier-dependent glycosyltransferase | TRNA_RS42200 | BL14DL4_02445 | BLDA23_22230 | CK945_RS22555 | ACH97_212745 |
| ABC transporter permease | TRNA_RS42205 | BL14DL4_02446 | BLDA23_22235 | CK945_RS22600 | ACH97_212740 |
| ABC transporter ATP-binding protein | TRNA_RS42210 | BL14DL4_02447 | BLDA23_22240 | CK945_RS22605 | ACH97_212735 |
| sensor histidine kinase | TRNA_RS42215 | BL14DL4_02448 | BLDA23_22245 | CK945_RS22610 | ACH97_212730 |
| DNA-binding response regulator | TRNA_RS42220 | BL14DL4_02449 | BLDA23_22250 | CK945_RS22615 | ACH97_212725 |
| glycoside hydrolase family 1 protein | TRNA_RS42225 | BL14DL4_02450 | BLDA23_22255 | CK945_RS22620 | ACH97_212720 |
| PTS beta-glucoside transporter subunit EIIBCA | TRNA_RS42230 | BL14DL4_02451 | BLDA23_22260 | CK945_RS22625 | ACH97_212715 |
| linear amide C-N hydrolase | TRNA_RS42240 | BL14DL4_02453 | BLDA23_22270 | CK945_RS22635 | ACH97_212705 |
| pyrimidine-nucleoside phosphorylase | TRNA_RS42290 | BL14DL4_02463 | BLDA23_22315 | CK945_RS22660 | ACH97_212650 |
| NupC/NupG family nucleoside CNT transporter | TRNA_RS42295 | BL14DL4_02464 | BLDA23_22320 | CK945_RS22665 | ACH97_212645 |
| deoxyribose-phosphate aldolase | TRNA_RS42300 | BL14DL4_02465 | BLDA23_22325 | CK945_RS22670 | ACH97_212640 |
| sugar-binding transcriptional regulator | TRNA_RS42305 | BL14DL4_02466 | BLDA23_22330 | CK945_RS22675 | ACH97_212635 |
| diguanylate cyclase | TRNA_RS42320 | BL14DL4_02469 | BLDA23_22345 | CK945_RS22685 | ACH97_212625 |
| hypothetical protein | TRNA_RS42335 | BL14DL4_02472 | BLDA23_22360 | CK945_RS22700 | ACH97_212610 |
| valine--pyruvate transaminase | TRNA_RS42340 | BL14DL4_02473 | BLDA23_22365 | CK945_RS22705 | ACH97_212605 |
| hypothetical protein | TRNA_RS42350 | BL14DL4_02475 | BLDA23_22375 | CK945_RS22715 | ACH97_212595 |
| ATP-grasp domain-containing protein | TRNA_RS42355 | BL14DL4_02476 | BLDA23_22380 | CK945_RS22720 | ACH97_212590 |
| EamA family transporter | TRNA_RS42360 | BL14DL4_02477 | BLDA23_22385 | CK945_RS22725 | ACH97_212585 |
| class II fructose-1,6-bisphosphate aldolase | TRNA_RS42365 | BL14DL4_02478 | BLDA23_22390 | CK945_RS22730 | ACH97_212580 |
| inosose isomerase | TRNA_RS42370 | BL14DL4_02479 | BLDA23_22395 | CK945_RS22735 | ACH97_212575 |
| sugar phosphate isomerase/epimerase | TRNA_RS42375 | BL14DL4_02480 | BLDA23_22400 | CK945_RS22740 | ACH97_212570 |
| gfo/Idh/MocA family oxidoreductase | TRNA_RS42380 | BL14DL4_02481 | BLDA23_22405 | CK945_RS22745 | ACH97_212565 |
| MFS transporter | TRNA_RS42385 | BL14DL4_02482 | BLDA23_22410 | CK945_RS22750 | ACH97_212560 |
| myo-inosose-2 dehydratase | TRNA_RS42390 | BL14DL4_02483 | BLDA23_22415 | CK945_RS22755 | ACH97_212555 |
| 3D-(3,5/4)-trihydroxycyclohexane-1 | TRNA_RS42395 | BL14DL4_02484 | BLDA23_22420 | CK945_RS22760 | ACH97_212550 |
| 5-dehydro-2-deoxygluconokinase | TRNA_RS42400 | BL14DL4_02485 | BLDA23_22425 | CK945_RS22765 | ACH97_212545 |
| 5-deoxy-glucuronate isomerase | TRNA_RS42405 | BL14DL4_02486 | BLDA23_22430 | CK945_RS22770 | ACH97_212540 |
| methylmalonate-semialdehyde dehydrogenase (CoA acylating) | TRNA_RS42410 | BL14DL4_02487 | BLDA23_22435 | CK945_RS22775 | ACH97_212535 |
| DeoR/GlpR transcriptional regulator | TRNA_RS42415 | BL14DL4_02488 | BLDA23_22440 | CK945_RS22780 | ACH97_212530 |
| aldo/keto reductase | TRNA_RS42420 | BL14DL4_02489 | BLDA23_22445 | CK945_RS22785 | ACH97_212525 |
| glycerophosphodiester phosphodiesterase | TRNA_RS42425 | BL14DL4_02490 | BLDA23_22450 | CK945_RS22790 | ACH97_212520 |
| glycerol-3-phosphate transporter | TRNA_RS42430 | BL14DL4_02491 | BLDA23_22455 | CK945_RS22795 | ACH97_212515 |
| molecular chaperone HtpG | TRNA_RS42435 | BL14DL4_02492 | BLDA23_22460 | CK945_RS22800 | ACH97_212510 |
| ABC transporter ATP-binding protein | TRNA_RS42440 | BL14DL4_02493 | BLDA23_22465 | CK945_RS22805 | ACH97_212505 |
| sugar-phosphatase | TRNA_RS42445 | BL14DL4_02494 | BLDA23_22470 | CK945_RS22810 | ACH97_212500 |
| 2,3-diketo-5-methylthio-1-phosphopentane phosphatase | TRNA_RS42455 | BL14DL4_02496 | BLDA23_22480 | CK945_RS22820 | ACH97_212490 |
| hypothetical protein | TRNA_RS42460 | BL14DL4_02497 | BLDA23_22485 | CK945_RS22825 | ACH97_212485 |
| iron-hydroxamate ABC transporter substrate-binding protein | TRNA_RS42465 | BL14DL4_02498 | BLDA23_22490 | CK945_RS22830 | ACH97_212480 |
| hypothetical protein | TRNA_RS42470 | BL14DL4_02499 | BLDA23_22495 | CK945_RS22835 | ACH97_212475 |
| VOC family protein | TRNA_RS42475 | BL14DL4_02500 | BLDA23_22500 | CK945_RS22840 | ACH97_212470 |
| ferrous iron transport protein B | TRNA_RS42485 | BL14DL4_02501 | BLDA23_22510 | CK945_RS22850 | ACH97_212460 |
| ferrous iron transport protein A | TRNA_RS42490 | BL14DL4_02502 | BLDA23_22515 | CK945_RS22855 | ACH97_212455 |
| acyltransferase | TRNA_RS42495 | BL14DL4_02503 | BLDA23_22520 | CK945_RS22860 | ACH97_212450 |
| ABC transporter permease | TRNA_RS42500 | BL14DL4_02504 | BLDA23_22525 | CK945_RS22865 | ACH97_212445 |
| ABC transporter ATP-binding protein | TRNA_RS42505 | BL14DL4_02505 | BLDA23_22530 | CK945_RS22870 | ACH97_212440 |
| sensor histidine kinase | TRNA_RS42510 | BL14DL4_02506 | BLDA23_22535 | CK945_RS22875 | ACH97_212435 |
| DNA-binding response regulator | TRNA_RS42515 | BL14DL4_02507 | BLDA23_22540 | CK945_RS22880 | ACH97_212430 |
| YxeA family protein | TRNA_RS42520 | BL14DL4_02508 | BLDA23_22545 | CK945_RS22885 | ACH97_212425 |
| methyl-accepting chemotaxis protein | TRNA_RS42525 | BL14DL4_02509 | BLDA23_22550 | CK945_RS22890 | ACH97_212420 |
| sodium:alanine symporter family protein | TRNA_RS42530 | BL14DL4_02510 | BLDA23_22555 | CK945_RS22895 | ACH97_212415 |
| alanine dehydrogenase | TRNA_RS42535 | BL14DL4_02511 | BLDA23_22560 | CK945_RS22900 | ACH97_212410 |
| arabinogalactan endo-beta-1,4-galactanase | TRNA_RS42540 | BL14DL4_02512 | BLDA23_22565 | CK945_RS22905 | ACH97_212405 |
| beta-galactosidase | TRNA_RS42545 | BL14DL4_02513 | BLDA23_22570 | CK945_RS22910 | ACH97_212400 |
| sugar ABC transporter permease | TRNA_RS42550 | BL14DL4_02514 | BLDA23_22575 | CK945_RS22915 | ACH97_212395 |
| sugar ABC transporter permease | TRNA_RS42555 | BL14DL4_02515 | BLDA23_22580 | CK945_RS22920 | ACH97_212390 |
| extracellular solute-binding protein | TRNA_RS42560 | BL14DL4_02516 | BLDA23_22585 | CK945_RS22925 | ACH97_212385 |
| LacI family DNA-binding transcriptional regulator | TRNA_RS42565 | BL14DL4_02517 | BLDA23_22590 | CK945_RS22930 | ACH97_212380 |
| galactokinase | TRNA_RS42570 | BL14DL4_02518 | BLDA23_22595 | CK945_RS22935 | ACH97_212375 |
| UDP-glucose 4-epimerase GalE | TRNA_RS42575 | BL14DL4_02519 | BLDA23_22600 | CK945_RS22940 | ACH97_212370 |
| UDP-glucose--hexose-1-phosphate uridylyltransferase | TRNA_RS42580 | BL14DL4_02520 | BLDA23_22605 | CK945_RS22945 | ACH97_212365 |
| ROK family transcriptional regulator | TRNA_RS42585 | BL14DL4_02521 | BLDA23_22610 | CK945_RS22950 | ACH97_212360 |
| GntR family transcriptional regulator | TRNA_RS42590 | BL14DL4_02522 | BLDA23_22615 | CK945_RS22970 | ACH97_212340 |
| gluconokinase | TRNA_RS42595 | BL14DL4_02523 | BLDA23_22620 | CK945_RS22975 | ACH97_212335 |
| gluconate permease | TRNA_RS42600 | BL14DL4_02524 | BLDA23_22625 | CK945_RS22980 | ACH97_212330 |
| decarboxylating NADP(+)-dependent phosphogluconate dehydrogenase | TRNA_RS42605 | BL14DL4_02525 | BLDA23_22630 | CK945_RS22985 | ACH97_212325 |
| bifunctional acetaldehyde-CoA/alcohol dehydrogenase | TRNA_RS42610 | BL14DL4_02526 | BLDA23_22635 | CK945_RS22995 | ACH97_212320 |
| peroxiredoxin | TRNA_RS42615 | BL14DL4_02527 | BLDA23_22640 | CK945_RS23000 | ACH97_212315 |
| alkyl hydroperoxide reductase subunit F | TRNA_RS42620 | BL14DL4_02528 | BLDA23_22645 | CK945_RS23005 | ACH97_212310 |
| ABC transporter ATP-binding protein | TRNA_RS42645 | BL14DL4_02533 | BLDA23_22670 | CK945_RS23065 | ACH97_212245 |
| DNA-binding response regulator | TRNA_RS42660 | BL14DL4_02536 | BLDA23_22685 | CK945_RS23080 | ACH97_212230 |
| DUF4234 domain-containing protein | TRNA_RS42770 | BL14DL4_02544 | BLDA23_22720 | CK945_RS23105 | ACH97_212210 |
| GNAT family N-acetyltransferase | TRNA_RS42775 | BL14DL4_02545 | BLDA23_22725 | CK945_RS23110 | ACH97_212205 |
| molybdate/tungstate-binding protein | TRNA_RS42785 | BL14DL4_02547 | BLDA23_22735 | CK945_RS23120 | ACH97_212195 |
| serine protease | TRNA_RS42790 | BL14DL4_02548 | BLDA23_22740 | CK945_RS23125 | ACH97_212190 |
| MBL fold metallo-hydrolase | TRNA_RS42795 | BL14DL4_02549 | BLDA23_22745 | CK945_RS23130 | ACH97_212185 |
| yycFG regulatory protein | TRNA_RS42800 | BL14DL4_02550 | BLDA23_22750 | CK945_RS23135 | ACH97_212180 |
| regulatory protein | TRNA_RS42805 | BL14DL4_02551 | BLDA23_22755 | CK945_RS23140 | ACH97_212175 |
| cell wall metabolism sensor histidine kinase WalK | TRNA_RS42810 | BL14DL4_02552 | BLDA23_22760 | CK945_RS23145 | ACH97_212170 |
| DNA-binding response regulator | TRNA_RS42815 | BL14DL4_02553 | BLDA23_22765 | CK945_RS23150 | ACH97_212165 |
| holin | TRNA_RS42850 | BL14DL4_02560 | BLDA23_22800 | CK945_RS23185 | ACH97_212130 |
| replicative DNA helicase | TRNA_RS42855 | BL14DL4_02561 | BLDA23_22805 | CK945_RS23190 | ACH97_212125 |
| DUF2188 domain-containing protein | TRNA_RS42860 | BL14DL4_02562 | BLDA23_22810 | CK945_RS23195 | ACH97_212120 |
| hypothetical protein | TRNA_RS42865 | BL14DL4_02563 | BLDA23_22815 | CK945_RS23200 | ACH97_212115 |
| cyanate MFS transporter | TRNA_RS42870 | BL14DL4_02565 | BLDA23_22825 | CK945_RS23210 | ACH97_212110 |
| N-acetylglucosamine-6-phosphate deacetylase | TRNA_RS42875 | BL14DL4_02566 | BLDA23_22830 | CK945_RS23215 | ACH97_212105 |
| glucosamine-6-phosphate deaminase | TRNA_RS42880 | BL14DL4_02567 | BLDA23_22835 | CK945_RS23220 | ACH97_212100 |
| GntR family transcriptional regulator | TRNA_RS42885 | BL14DL4_02568 | BLDA23_22840 | CK945_RS23225 | ACH97_212095 |
| N-acetylmuramic acid 6-phosphate etherase | TRNA_RS42890 | BL14DL4_02569 | BLDA23_22845 | CK945_RS23230 | ACH97_212090 |
| PTS acetylglucosamine transporter subunit IIB | TRNA_RS42895 | BL14DL4_02570 | BLDA23_22850 | CK945_RS23235 | ACH97_212085 |
| DHH family phosphoesterase | TRNA_RS42905 | BL14DL4_02572 | BLDA23_22860 | CK945_RS23245 | ACH97_212075 |
| DUF2232 domain-containing protein | TRNA_RS42910 | BL14DL4_02573 | BLDA23_22865 | CK945_RS23250 | ACH97_212070 |
| universal stress protein | TRNA_RS42925 | BL14DL4_02575 | BLDA23_22875 | CK945_RS23265 | ACH97_212060 |
| SulP family inorganic anion transporter | TRNA_RS42930 | BL14DL4_02576 | BLDA23_22880 | CK945_RS23270 | ACH97_212055 |
| MFS transporter | TRNA_RS42935 | BL14DL4_02577 | BLDA23_22885 | CK945_RS23275 | ACH97_212050 |
| 30S ribosomal protein S18 | TRNA_RS42940 | BL14DL4_02578 | BLDA23_22890 | CK945_RS23280 | ACH97_212045 |
| single-stranded DNA-binding protein | TRNA_RS42945 | BL14DL4_02579 | BLDA23_22895 | CK945_RS23285 | ACH97_212040 |
| 30S ribosomal protein S6 | TRNA_RS42950 | BL14DL4_02580 | BLDA23_22900 | CK945_RS23290 | ACH97_212035 |
| redox-regulated ATPase YchF | TRNA_RS42955 | BL14DL4_02581 | BLDA23_22905 | CK945_RS23295 | ACH97_212030 |
| DUF951 domain-containing protein | TRNA_RS42965 | BL14DL4_02582 | BLDA23_22915 | CK945_RS23305 | ACH97_212020 |
| hypothetical protein | TRNA_RS42970 | BL14DL4_02583 | BLDA23_22920 | CK945_RS23310 | ACH97_212015 |
| spore protease YyaC | TRNA_RS42975 | BL14DL4_02584 | BLDA23_22925 | CK945_RS23315 | ACH97_212010 |
| ParB/RepB/Spo0J family partition protein | TRNA_RS42980 | BL14DL4_02585 | BLDA23_22930 | CK945_RS23320 | ACH97_212005 |
| ParA family protein | TRNA_RS42985 | BL14DL4_02586 | BLDA23_22935 | CK945_RS23325 | ACH97_212000 |
| carboxymuconolactone decarboxylase family protein | TRNA_RS42990 | BL14DL4_02587 | BLDA23_22940 | CK945_RS23330 | ACH97_211995 |
| RNA polymerase sigma-70 factor | TRNA_RS42995 | BL14DL4_02588 | BLDA23_22945 | CK945_RS23335 | ACH97_211990 |
| nucleoid occlusion protein | TRNA_RS43000 | BL14DL4_02589 | BLDA23_22950 | CK945_RS23340 | ACH97_211985 |
| 16S rRNA (guanine(527)-N(7))-methyltransferase RsmG | TRNA_RS43005 | BL14DL4_02590 | BLDA23_22955 | CK945_RS23345 | ACH97_211980 |
| tRNA uridine-5-carboxymethylaminomethyl(34) synthesis enzyme MnmG | TRNA_RS43010 | BL14DL4_02591 | BLDA23_22960 | CK945_RS23350 | ACH97_211975 |
| tRNA uridine-5-carboxymethylaminomethyl(34) synthesis GTPase MnmE | TRNA_RS43015 | BL14DL4_02592 | BLDA23_22965 | CK945_RS23355 | ACH97_211970 |
| protein jag | TRNA_RS43020 | BL14DL4_02593 | BLDA23_22970 | CK945_RS23360 | ACH97_211965 |
| Membrane integrase YidC | TRNA_RS43025 | BL14DL4_02594 | BLDA23_22975 | CK945_RS23365 | ACH97_211960 |
| ribonuclease P protein component | TRNA_RS43030 | BL14DL4_02595 | BLDA23_22980 | CK945_RS23370 | ACH97_211955 |
| DUF370 domain-containing protein | TRNA_RS21540 | - | BLDA23_00025 | CK945_RS00025 | ACH97_211930 |
| GntP family permease | TRNA_RS21615 | BL14DL4_02614 | - | CK945_RS00100 | ACH97_222430 |
| SMI1/KNR4 family protein | TRNA_RS21625 | BL14DL4_02616 | BLDA23_00110 | CK945_RS00110 | ACH97_222420 |
| sigma factor G inhibitor Gin | TRNA_RS43075 | BL14DL4_02633 | BLDA23_00200 | CK945_RS00200 | - |
| 16S rRNA (cytidine(1402)-2-O)-methyltransferase | TRNA_RS21765 | BL14DL4_02645 | BLDA23_00260 | - | ACH97_205815 |
| transcription-repair coupling factor | TRNA_RS21860 | BL14DL4_02665 | BLDA23_00355 | - | ACH97_205910 |
| hypothetical protein | TRNA_RS21965 | - | BLDA23_00460 | - | - |
| XRE family transcriptional regulator | TRNA_RS43080 | BL14DL4_02692 | BLDA23_00495 | CK945_RS00495 | - |
| hypothetical protein | TRNA_RS22035 | BL14DL4_02711 | BLDA23_00590 | - | ACH97_220645 |
| 50S ribosomal protein L33 | TRNA_RS43085 | - | BLDA23_00665 | CK945_RS00665 | - |
| class I SAM-dependent methyltransferase | TRNA_RS22140 | BL14DL4_02732 | BLDA23_00700 | - | ACH97_220540 |
| 30S ribosomal protein S14 type Z 1 | TRNA_RS43090 | BL14DL4_02754 | BLDA23_00810 | CK945_RS00810 | - |
| PhzF family phenazine biosynthesis protein | TRNA_RS22440 | BL14DL4_02794 | BLDA23_01005 | CK945_RS01120 | ACH97_221745 |
| SRPBCC domain-containing protein | TRNA_RS22590 | BL14DL4_02824 | BLDA23_01155 | - | - |
| amino acid permease | TRNA_RS22610 | BL14DL4_02828 | BLDA23_01175 | CK945_RS01350 | - |
| citrate transporter | TRNA_RS22615 | BL14DL4_02829 | BLDA23_01180 | CK945_RS01355 | - |
| tripartite tricarboxylate transporter substrate binding protein | TRNA_RS22620 | BL14DL4_02830 | BLDA23_01185 | CK945_RS01360 | - |
| response regulator | TRNA_RS22625 | BL14DL4_02831 | BLDA23_01190 | CK945_RS01365 | - |
| sensor histidine kinase | TRNA_RS22630 | BL14DL4_02832 | BLDA23_01195 | CK945_RS01370 | - |
| LysR family transcriptional regulator | TRNA_RS22635 | BL14DL4_02833 | BLDA23_01200 | - | ACH97_221935 |
| ATP-grasp domain-containing protein | TRNA_RS22640 | BL14DL4_02834 | BLDA23_01205 | - | ACH97_221940 |
| MFS transporter | TRNA_RS22645 | BL14DL4_02835 | BLDA23_01210 | - | ACH97_221945 |
| DUF255 domain-containing protein | TRNA_RS22700 | BL14DL4_02844 | BLDA23_01240 | CK945_RS01435 | - |
| hypothetical protein | TRNA_RS22735 | - | - | CK945_RS01525 | ACH97_215630 |
| hypothetical protein | TRNA_RS22765 | BL14DL4_02857 | BLDA23_01310 | - | - |
| methyl-accepting chemotaxis protein | TRNA_RS22785 | BL14DL4_02861 | BLDA23_01330 | - | - |
| sugar ABC transporter substrate-binding protein | TRNA_RS22790 | BL14DL4_02862 | BLDA23_01335 | - | - |
| hypothetical protein | TRNA_RS22850 | - | BLDA23_01395 | - | - |
| class A beta-lactamase BlaP | TRNA_RS22895 | BL14DL4_02882 | BLDA23_01440 | CK945_RS01675 | - |
| hypothetical protein | TRNA_RS23015 | BL14DL4_02907 | BLDA23_01560 | - | - |
| RDD family protein | TRNA_RS23040 | BL14DL4_02912 | BLDA23_01585 | - | - |
| SDR family NAD(P)-dependent oxidoreductase | TRNA_RS23085 | - | BLDA23_01630 | CK945_RS01875 | ACH97_215280 |
| oligoendopeptidase F | TRNA_RS23120 | BL14DL4_02928 | BLDA23_01665 | - | ACH97_215240 |
| chitinase | TRNA_RS23180 | BL14DL4_02940 | - | CK945_RS01975 | ACH97_215180 |
| sulfurtransferase | TRNA_RS23205 | BL14DL4_02945 | BLDA23_01750 | - | ACH97_215155 |
| ABC transporter ATP-binding protein | TRNA_RS23230 | BL14DL4_02950 | BLDA23_01775 | CK945_RS02025 | ACH97_215130 |
| DUF4188 domain-containing protein | TRNA_RS23245 | BL14DL4_02953 | BLDA23_01790 | CK945_RS02040 | - |
| PadR family transcriptional regulator | TRNA_RS23250 | BL14DL4_02954 | BLDA23_01795 | CK945_RS02045 | - |
| CPBP family intramembrane metalloprotease | TRNA_RS23300 | BL14DL4_02964 | BLDA23_01845 | - | ACH97_215070 |
| MerR family transcriptional regulator | TRNA_RS23305 | BL14DL4_02965 | BLDA23_01850 | - | ACH97_215065 |
| NarK/NasA family nitrate transporter | TRNA_RS23310 | BL14DL4_02966 | BLDA23_01855 | - | ACH97_215060 |
| amino acid permease | TRNA_RS23330 | BL14DL4_02970 | BLDA23_01875 | - | ACH97_215040 |
| DNA-entry nuclease | TRNA_RS23430 | BL14DL4_02990 | BLDA23_01975 | - | ACH97_214935 |
| EamA family transporter | TRNA_RS23505 | BL14DL4_03005 | BLDA23_02045 | CK945_RS02290 | ACH97_214865 |
| hypothetical protein | TRNA_RS23535 | BL14DL4_03011 | BLDA23_02075 | - | - |
| ABC transporter ATP-binding protein | TRNA_RS23540 | BL14DL4_03012 | BLDA23_02080 | - | - |
| transcriptional regulator | TRNA_RS23545 | BL14DL4_03013 | BLDA23_02085 | - | - |
| hypothetical protein | TRNA_RS23550 | - | BLDA23_02090 | - | - |
| LysR family transcriptional regulator | TRNA_RS23600 | BL14DL4_03024 | BLDA23_02140 | CK945_RS02390 | - |
| hypothetical protein | TRNA_RS23650 | BL14DL4_03034 | BLDA23_02190 | - | - |
| hypothetical protein | TRNA_RS23655 | BL14DL4_03035 | BLDA23_02195 | - | ACH97_214720 |
| transposase | TRNA_RS23660 | BL14DL4_03036 | BLDA23_02200 | - | - |
| sugar ABC transporter substrate-binding protein | TRNA_RS23675 | BL14DL4_03039 | BLDA23_02215 | - | ACH97_214685 |
| DNA-binding response regulator | TRNA_RS23685 | BL14DL4_03041 | BLDA23_02225 | - | ACH97_214675 |
| germination protein KB | TRNA_RS23750 | BL14DL4_03054 | BLDA23_02290 | - | ACH97_217065 |
| methyl-accepting chemotaxis protein | TRNA_RS23780 | BL14DL4_03060 | - | CK945_RS02575 | ACH97_217035 |
| hypothetical protein | TRNA_RS23920 | BL14DL4_03088 | BLDA23_02460 | - | - |
| hypothetical protein | TRNA_RS23925 | - | BLDA23_02465 | CK945_RS02715 | ACH97_216895 |
| hypothetical protein | TRNA_RS23935 | - | BLDA23_02475 | - | - |
| PRD domain-containing protein | TRNA_RS23940 | BL14DL4_03090 | BLDA23_02480 | - | ACH97_216885 |
| PTS ascorbate transporter subunit IIC | TRNA_RS23950 | BL14DL4_03092 | BLDA23_02490 | - | ACH97_216875 |
| NAD(P)-dependent oxidoreductase | TRNA_RS24025 | BL14DL4_03107 | BLDA23_02565 | - | ACH97_216800 |
| APC family permease | TRNA_RS24075 | BL14DL4_03117 | - | - | - |
| IS3 family transposase,IDENTICAL PARALOGS:TRNA_RS29820 | TRNA_RS24090 | - | BLDA23_02630 | - | - |
| acyl-CoA dehydrogenase | TRNA_RS24180 | BL14DL4_03136 | BLDA23_02715 | - | ACH97_216640 |
| MMPL family transporter | TRNA_RS24305 | BL14DL4_03161 | BLDA23_02840 | - | ACH97_216510 |
| cortex morphogenetic protein CmpA | TRNA_RS43540 | - | BLDA23_02845 | CK945_RS03100 | - |
| hypothetical protein | TRNA_RS24360 | BL14DL4_03170 | BLDA23_02890 | - | ACH97_216460 |
| transcriptional regulator | TRNA_RS24365 | BL14DL4_03171 | BLDA23_02895 | - | ACH97_216455 |
| fatty acid desaturase | TRNA_RS24370 | - | BLDA23_02900 | CK945_RS03180 | ACH97_216450 |
| hypothetical protein | TRNA_RS24385 | BL14DL4_03175 | BLDA23_02915 | - | - |
| YitT family protein | TRNA_RS24390 | BL14DL4_03176 | BLDA23_02920 | CK945_RS03210 | - |
| lysine transporter LysE | TRNA_RS24425 | BL14DL4_03183 | - | - | - |
| LysR family transcriptional regulator | TRNA_RS24430 | BL14DL4_03184 | BLDA23_02960 | - | - |
| MFS transporter | TRNA_RS24440 | BL14DL4_03186 | BLDA23_02970 | CK945_RS03270 | ACH97_216350 |
| YIP1 family protein | TRNA_RS24460 | BL14DL4_03190 | BLDA23_02990 | - | - |
| response regulator | TRNA_RS24485 | BL14DL4_03195 | BLDA23_03015 | - | ACH97_216285 |
| hypothetical protein | TRNA_RS24615 | BL14DL4_03221 | BLDA23_03415 | - | - |
| hypothetical protein | TRNA_RS24620 | BL14DL4_03222 | BLDA23_03420 | - | - |
| hypothetical protein | TRNA_RS24625 | - | - | - | - |
| hypothetical protein | TRNA_RS24630 | BL14DL4_03223 | BLDA23_03425 | - | - |
| YolD-like family protein | TRNA_RS24635 | BL14DL4_03224 | BLDA23_03430 | CK945_RS03845 | - |
| hypothetical protein | TRNA_RS24665 | - | BLDA23_03460 | CK945_RS03900 | ACH97_204870 |
| TetR/AcrR family transcriptional regulator | TRNA_RS24670 | BL14DL4_03230 | - | CK945_RS03905 | ACH97_204875 |
| phosphoenolpyruvate synthase | TRNA_RS24675 | BL14DL4_03231 | BLDA23_03470 | - | ACH97_204880 |
| membrane protein | TRNA_RS24735 | BL14DL4_03243 | BLDA23_03530 | - | ACH97_204940 |
| hypothetical protein | TRNA_RS24755 | - | BLDA23_03550 | - | - |
| YafY family transcriptional regulator | TRNA_RS24765 | BL14DL4_03248 | BLDA23_03560 | CK945_RS03995 | ACH97_204965 |
| DUF3298/DUF4163 domain-containing protein | TRNA_RS24770 | BL14DL4_03249 | BLDA23_03565 | CK945_RS04000 | - |
| DUF4163 domain-containing protein | TRNA_RS24775 | BL14DL4_03250 | BLDA23_03570 | - | ACH97_204975 |
| hypothetical protein | TRNA_RS24935 | - | BLDA23_03730 | - | - |
| DDE transposase | TRNA_RS25040 | BL14DL4_03303 | BLDA23_03840 | - | - |
| HNH endonuclease | TRNA_RS25225 | - | BLDA23_04055 | CK945_RS04445 | - |
| PTS beta-glucoside transporter subunit EIIBCA | TRNA_RS25230 | BL14DL4_03340 | BLDA23_04065 | CK945_RS04450 | - |
| glycoside hydrolase family 1 protein | TRNA_RS25235 | BL14DL4_03341 | BLDA23_04070 | CK945_RS04455 | - |
| PRD domain-containing protein | TRNA_RS25240 | BL14DL4_03342 | BLDA23_04075 | CK945_RS04460 | ACH97_205435 |
| DASS family sodium-coupled anion symporter | TRNA_RS25285 | BL14DL4_03351 | BLDA23_04125 | - | ACH97_205530 |
| hypothetical protein | TRNA_RS25295 | - | - | - | - |
| NAD-binding site protein | TRNA_RS25305 | BL14DL4_03354 | BLDA23_04140 | CK945_RS04575 | ACH97_205555 |
| 6-carboxyhexanoate--CoA ligase | TRNA_RS25325 | BL14DL4_03357 | BLDA23_04155 | - | ACH97_205570 |
| VanZ family protein | TRNA_RS25400 | - | BLDA23_04230 | CK945_RS04680 | ACH97_205665 |
| DUF2639 domain-containing protein | TRNA_RS43120 | BL14DL4_03381 | BLDA23_04275 | CK945_RS04730 | - |
| hypothetical protein | TRNA_RS43550 | BL14DL4_03382 | - | CK945_RS04735 | - |
| sodium:alanine symporter family protein | TRNA_RS25460 | BL14DL4_03386 | BLDA23_04295 | - | ACH97_205730 |
| collagen-like protein | TRNA_RS25485 | - | - | CK945_RS04785 | - |
| collagen-like protein | TRNA_RS43040 | BL14DL4_03393 | - | - | - |
| NAD-dependent epimerase/dehydratase family protein | TRNA_RS25510 | BL14DL4_03396 | BLDA23_04350 | CK945_RS04805 | ACH97_211270 |
| delta-lactam-biosynthetic de-N-acetylase | TRNA_RS25625 | BL14DL4_03420 | BLDA23_04465 | - | ACH97_211385 |
| hypothetical protein | TRNA_RS25635 | BL14DL4_03422 | - | CK945_RS04930 | ACH97_211395 |
| chorismate mutase | TRNA_RS43125 | - | BLDA23_04500 | - | - |
| hypothetical protein | TRNA_RS25660 | BL14DL4_03427 | - | - | ACH97_211425 |
| sigma-54-dependent Fis family transcriptional regulator | TRNA_RS25745 | BL14DL4_03444 | BLDA23_04590 | - | ACH97_211520 |
| extracellular solute-binding protein | TRNA_RS25810 | BL14DL4_03457 | BLDA23_04655 | CK945_RS05115 | ACH97_211585 |
| flavodoxin family protein | TRNA_RS25815 | BL14DL4_03458 | - | CK945_RS05120 | - |
| hypothetical protein | TRNA_RS43130 | - | - | - | - |
| YfhE family protein | TRNA_RS43135 | BL14DL4_03466 | BLDA23_04695 | CK945_RS05155 | - |
| YpzG family protein | TRNA_RS43140 | BL14DL4_03471 | BLDA23_04720 | CK945_RS05180 | - |
| hypothetical protein | TRNA_RS25880 | - | BLDA23_04735 | - | - |
| aspartate phosphatase | TRNA_RS26090 | BL14DL4_03515 | - | - | - |
| integrase | TRNA_RS43145 | - | BLDA23_05385 | - | - |
| hypothetical protein | TRNA_RS26095 | - | BLDA23_05390 | - | - |
[truncated: 149,204 more chars]
