# Supplementary material for: Two genes involved in clindamycin resistance of Bacillus licheniformis and Bacillus paralicheniformis identified by comparative genomic analysis
Source: PLoS One. 2020 Apr 9;15(4):e0231274. doi: 10.1371/journal.pone.0231274 (PMC7144989; doi:10.1371/journal.pone.0231274)
Supplement: S2 Table — (DOCX) [file pone.0231274.s002.docx]

**S2 Table. List of CDSs commonly identified only in the genomes of C^R^E^R^ strains 14DA11 and KJ-16^T^.**

|  | DSM 13^T^ | 14ADL4 | 0DA23-1 | 14DA11 | KJ-16^T^ |
| --- | --- | --- | --- | --- | --- |
| EamA family transporter | - | - | - | CK945_RS04490 | ACH97_205465 |
| PLP-dependent aminotransferase family protein | - | - | - | CK945_RS04495 | ACH97_205470 |
| EamA/RhaT family transporter | - | - | - | CK945_RS04500 | ACH97_205475 |
| hypothetical protein | - | - | - | CK945_RS00930 | ACH97_220315 |
| hypothetical protein | - | - | - | CK945_RS00950 | ACH97_220300 |
| hypothetical protein | - | - | - | CK945_RS00955 | ACH97_220295 |
| hypothetical protein | - | - | - | CK945_RS00960 | ACH97_220290 |
| ABC transporter ATP-binding protein | - | - | - | CK945_RS00965 | ACH97_220285 |
| hypothetical protein | - | - | - | CK945_RS00975 | ACH97_220275 |
| hypothetical protein | - | - | - | CK945_RS00980 | ACH97_220270 |
| bacitracin ABC transporter ATP-binding protein | - | - | - | CK945_RS00985 | ACH97_220265 |
| hypothetical protein | - | - | - | CK945_RS00990 | ACH97_220260 |
| hypothetical protein | - | - | - | CK945_RS01000 | ACH97_220250 |
| hypothetical protein | - | - | - | CK945_RS01015 | ACH97_220240 |
| hypothetical protein | - | - | - | CK945_RS01025 | ACH97_220230 |
| hypothetical protein | - | - | - | CK945_RS01030 | ACH97_220225 |
| two-component system response regulator | - | - | - | CK945_RS01040 | ACH97_220220 |
| hypothetical protein | - | - | - | CK945_RS01330 | ACH97_221910 |
| hypothetical protein | - | - | - | CK945_RS01410 | ACH97_222505 |
| serine hydrolase | - | - | - | CK945_RS01420 | ACH97_222510 |
| hypothetical protein | - | - | - | CK945_RS01455 | ACH97_208545 |
| hypothetical protein | - | - | - | CK945_RS01465 | ACH97_215685 |
| 2-diaminobutyrate decarboxylase | - | - | - | CK945_RS01480 | ACH97_215675 |
| GntR family transcriptional regulator | - | - | - | CK945_RS01490 | ACH97_215665 |
| hypothetical protein | - | - | - | CK945_RS01495 | ACH97_215660 |
| hypothetical protein | - | - | - | CK945_RS01505 | ACH97_215650 |
| SAM-dependent methyltransferase | - | - | - | CK945_RS01565 | ACH97_215590 |
| saccharopine dehydrogenase | - | - | - | CK945_RS01570 | ACH97_215585 |
| lipoprotein YvcA | - | - | - | CK945_RS01725 | ACH97_215425 |
| hypothetical protein | - | - | - | CK945_RS01730 | ACH97_215420 |
| hypothetical protein | - | - | - | CK945_RS01735 | ACH97_215415 |
| L-asparaginase | - | - | - | CK945_RS01900 | ACH97_215255 |
| hypothetical protein | - | - | - | CK945_RS02140 | ACH97_215020 |
| hypothetical protein | - | - | - | CK945_RS02440 | ACH97_214710 |
| hypothetical protein | - | - | - | CK945_RS02515 | ACH97_214635 |
| hypothetical protein | - | - | - | CK945_RS02860 | ACH97_216750 |
| aminobenzoate synthetase | - | - | - | CK945_RS02865 | ACH97_216745 |
| 1,4-beta-xylanase | - | - | - | CK945_RS03175 | ACH97_216465 |
| dehydrogenase | - | - | - | CK945_RS03245 | ACH97_216375 |
| transcriptional regulator | - | - | - | CK945_RS03250 | ACH97_216370 |
| hydrolase | - | - | - | CK945_RS03255 | ACH97_216365 |
| transcriptional regulator | - | - | - | CK945_RS03260 | ACH97_216360 |
| zinc-binding protein | - | - | - | CK945_RS03290 | ACH97_216330 |
| hypothetical protein | - | - | - | CK945_RS03295 | ACH97_216325 |
| hypothetical protein | - | - | - | CK945_RS03300 | ACH97_216320 |
| DNA mismatch repair protein MutT | - | - | - | CK945_RS03320 | ACH97_216300 |
| butanol dehydrogenase | - | - | - | CK945_RS03330 | ACH97_216290 |
| hypothetical protein | - | - | - | CK945_RS03355 | ACH97_216265 |
| hypothetical protein | - | - | - | CK945_RS03815 | ACH97_218735 |
| hypothetical protein | - | - | - | CK945_RS03840 | ACH97_209835 |
| hypothetical protein | - | - | - | CK945_RS03885 | ACH97_204855 |
| hypothetical protein | - | - | - | CK945_RS04165 | ACH97_205135 |
| DNA helicase | - | - | - | CK945_RS04480 | ACH97_205455 |
| hypothetical protein | - | - | - | CK945_RS04485 | ACH97_205460 |
| protoheme IX farnesyltransferase | - | - | - | CK945_RS04530 | ACH97_205520 |
| TetR family transcriptional regulator | - | - | - | CK945_RS04535 | ACH97_205525 |
| hypothetical protein | - | - | - | CK945_RS04550 | ACH97_205540 |
| peptidase | - | - | - | CK945_RS04555 | ACH97_205545 |
| hypothetical protein | - | - | - | CK945_RS04665 | ACH97_205650 |
| hypothetical protein | - | - | - | CK945_RS04670 | ACH97_205655 |
| hypothetical protein | - | - | - | CK945_RS04675 | ACH97_205660 |
| glycosylase | - | - | - | CK945_RS04705 | ACH97_205690 |
| hypothetical protein | - | - | - | CK945_RS04990 | ACH97_211455 |
| hypothetical protein | - | - | - | CK945_RS05430 | ACH97_200420 |
| hypothetical protein | - | - | - | CK945_RS05490 | ACH97_218935 |
| hypothetical protein | - | - | - | CK945_RS05495 | ACH97_218940 |
| phage tail protein | - | - | - | CK945_RS05625 | ACH97_200580 |
| transposase | - | - | - | CK945_RS05660 | ACH97_210755 |
| hypothetical protein | - | - | - | CK945_RS05695 | ACH97_204620 |
| hypothetical protein | - | - | - | CK945_RS05700 | ACH97_204615 |
| hypothetical protein | - | - | - | CK945_RS05705 | ACH97_204610 |
| hypothetical protein | - | - | - | CK945_RS05850 | ACH97_204460 |
| hypothetical protein | - | - | - | CK945_RS05965 | ACH97_204340 |
| general stress protein | - | - | - | CK945_RS06145 | ACH97_204160 |
| hypothetical protein | - | - | - | CK945_RS06625 | ACH97_205615 |
| hypothetical protein | - | - | - | CK945_RS06810 | ACH97_203515 |
| hypothetical protein | - | - | - | CK945_RS06920 | ACH97_203405 |
| spore gernimation protein | - | - | - | CK945_RS06980 | ACH97_203345 |
| hypothetical protein | - | - | - | CK945_RS07305 | ACH97_203025 |
| Pathogenicity locus | - | - | - | CK945_RS07545 | ACH97_202805 |
| lipase | - | - | - | CK945_RS07610 | ACH97_202745 |
| hypothetical protein | - | - | - | CK945_RS07615 | ACH97_202740 |
| DNA-binding protein | - | - | - | CK945_RS07620 | ACH97_202735 |
| hypothetical protein | - | - | - | CK945_RS07635 | ACH97_202720 |
| N-acetylmuramoyl-L-alanine amidase | - | - | - | CK945_RS07640 | ACH97_202715 |
| hypothetical protein | - | - | - | CK945_RS07645 | ACH97_202710 |
| hypothetical protein | - | - | - | CK945_RS07650 | ACH97_202705 |
| hypothetical protein | - | - | - | CK945_RS07655 | ACH97_202700 |
| hypothetical protein | - | - | - | CK945_RS08220 | ACH97_221595 |
| methylthioribulose-1-phosphate dehydratase | - | - | - | CK945_RS08435 | ACH97_222265 |
| hypothetical protein | - | - | - | CK945_RS08545 | ACH97_204705 |
| hypothetical protein | - | - | - | CK945_RS08550 | ACH97_204700 |
| hypothetical protein | - | - | - | CK945_RS08555 | ACH97_204695 |
| hypothetical protein | - | - | - | CK945_RS08565 | ACH97_204680 |
| hypothetical protein | - | - | - | CK945_RS08580 | ACH97_204670 |
| lipase | - | - | - | CK945_RS08685 | ACH97_218530 |
| hypothetical protein | - | - | - | CK945_RS08695 | ACH97_218520 |
| hypothetical protein | - | - | - | CK945_RS09110 | ACH97_218120 |
| hypothetical protein | - | - | - | CK945_RS09170 | ACH97_218070 |
| hypothetical protein | - | - | - | CK945_RS10385 | ACH97_206950 |
| hypothetical protein | - | - | - | CK945_RS10390 | ACH97_206955 |
| hypothetical protein | - | - | - | CK945_RS10410 | ACH97_206970 |
| hypothetical protein | - | - | - | CK945_RS10425 | ACH97_206985 |
| tetracycline resistance protein | - | - | - | CK945_RS10450 | ACH97_207005 |
| hypothetical protein | - | - | - | CK945_RS10455 | ACH97_207010 |
| hypothetical protein | - | - | - | CK945_RS10680 | ACH97_211145 |
| peptidase S66 | - | - | - | CK945_RS10740 | ACH97_211100 |
| hypothetical protein | - | - | - | CK945_RS10765 | ACH97_211075 |
| peptidase | - | - | - | CK945_RS10770 | ACH97_211070 |
| non-ribosomal peptide synthetase | - | - | - | CK945_RS11090 | ACH97_222480 |
| non-ribosomal peptide synthetase | - | - | - | CK945_RS11095 | ACH97_208115 |
| non-ribosomal peptide synthetase | - | - | - | CK945_RS11100 | ACH97_208110 |
| TetR family transcriptional regulator | - | - | - | CK945_RS11165 | ACH97_208045 |
| hypothetical protein | - | - | - | CK945_RS11170 | ACH97_208040 |
| hypothetical protein | - | - | - | CK945_RS11175 | ACH97_208035 |
| hypothetical protein | - | - | - | CK945_RS11180 | ACH97_208030 |
| hypothetical protein | - | - | - | CK945_RS11190 | ACH97_208020 |
| hypothetical protein | - | - | - | CK945_RS11310 | ACH97_207910 |
| potassium transporter | - | - | - | CK945_RS11850 | ACH97_207380 |
| potassium:proton antiporter | - | - | - | CK945_RS11855 | ACH97_207375 |
| catalase | - | - | - | CK945_RS11860 | ACH97_207370 |
| hypothetical protein | - | - | - | CK945_RS12210 | ACH97_207300 |
| hypothetical protein | - | - | - | CK945_RS12215 | ACH97_207295 |
| hypothetical protein | - | - | - | CK945_RS12220 | ACH97_207290 |
| urease accessory protein UreH | - | - | - | CK945_RS12225 | ACH97_207285 |
| urease accessory protein UreD | - | - | - | CK945_RS12230 | ACH97_207280 |
| urease accessory protein UreG | - | - | - | CK945_RS12235 | ACH97_207275 |
| urease accessory protein UreF | - | - | - | CK945_RS12240 | ACH97_207270 |
| urease accessory protein UreE | - | - | - | CK945_RS12245 | ACH97_207265 |
| urease subunit alpha | - | - | - | CK945_RS12250 | ACH97_207260 |
| urease subunit beta | - | - | - | CK945_RS12255 | ACH97_207255 |
| urea transporter | - | - | - | CK945_RS12265 | ACH97_207245 |
| D-alanyl-D-alanine carboxypeptidase | - | - | - | CK945_RS12325 | ACH97_211155 |
| hypothetical protein | - | - | - | CK945_RS12370 | ACH97_211190 |
| hypothetical protein | - | - | - | CK945_RS12375 | ACH97_211195 |
| hypothetical protein | - | - | - | CK945_RS12435 | ACH97_211250 |
| hypothetical protein | - | - | - | CK945_RS12480 | ACH97_208160 |
| serine hydrolase | - | - | - | CK945_RS12485 | ACH97_208165 |
| hypothetical protein | - | - | - | CK945_RS13705 | ACH97_209490 |
| GNAT family acetyltransferase | - | - | - | CK945_RS13820 | ACH97_209605 |
| bacitracin ABC transporter permease | - | - | - | CK945_RS13990 | ACH97_209770 |
| bacitracin ABC transporter permease | - | - | - | CK945_RS13995 | ACH97_209775 |
| bacitracin ABC transporter ATP-binding protein | - | - | - | CK945_RS14000 | ACH97_209780 |
| ATPase | - | - | - | CK945_RS14005 | ACH97_209785 |
| PhoP family transcriptional regulator | - | - | - | CK945_RS14010 | ACH97_209790 |
| non-ribosomal peptide synthetase | - | - | - | CK945_RS14030 | ACH97_209800 |
| gramicidin dehydrogenase | - | - | - | CK945_RS14040 | ACH97_209810 |
| hypothetical protein | - | - | - | CK945_RS14490 | ACH97_210275 |
| hypothetical protein | - | - | - | CK945_RS15440 | ACH97_219150 |
| HxlR family transcriptional regulator | - | - | - | CK945_RS15445 | ACH97_219155 |
| general stress protein | - | - | - | CK945_RS15450 | ACH97_219160 |
| 6-phospho-beta-glucosidase | - | - | - | CK945_RS15625 | ACH97_219300 |
| RpiR family transcriptional regulator | - | - | - | CK945_RS15630 | ACH97_219305 |
| 3-methylitaconate isomerase | - | - | - | CK945_RS15665 | ACH97_219335 |
| hypothetical protein | - | - | - | CK945_RS15670 | ACH97_219340 |
| nitrate reductase | - | - | - | CK945_RS15675 | ACH97_219345 |
| nitrate reductase | - | - | - | CK945_RS15680 | ACH97_219350 |
| nitrate reductase | - | - | - | CK945_RS15685 | ACH97_219355 |
| nitrate reductase | - | - | - | CK945_RS15690 | ACH97_219360 |
| nitrate/nitrite transporter | - | - | - | CK945_RS15695 | ACH97_219365 |
| dihydropteridine reductase | - | - | - | CK945_RS15700 | ACH97_219370 |
| 23S rRNA (adenine 2058-N6)-dimethyltransferase (ermC) | - | - | - | CK945_RS15790 | ACH97_219465 |
| tautomerase | - | - | - | CK945_RS15795 | ACH97_219470 |
| beta-xylosidase | - | - | - | CK945_RS15895 | ACH97_219575 |
| MFS transporter | - | - | - | CK945_RS15900 | ACH97_219580 |
| histidine kinase | - | - | - | CK945_RS15970 | ACH97_219650 |
| potassium-transporting ATPase subunit C | - | - | - | CK945_RS15975 | ACH97_219655 |
| potassium-transporting ATPase subunit B | - | - | - | CK945_RS15980 | ACH97_219660 |
| hypothetical protein | - | - | - | CK945_RS16760 | ACH97_217300 |
| hypothetical protein | - | - | - | CK945_RS16820 | ACH97_217360 |
| peptidase M84 | - | - | - | CK945_RS16825 | ACH97_217365 |
| TetR family transcriptional regulator | - | - | - | CK945_RS16855 | ACH97_217395 |
| hypothetical protein | - | - | - | CK945_RS16860 | ACH97_217400 |
| hypothetical protein | - | - | - | CK945_RS16865 | ACH97_217405 |
| hypothetical protein | - | - | - | CK945_RS17085 | ACH97_217625 |
| beta-glucanase | - | - | - | CK945_RS17090 | ACH97_217630 |
| hypothetical protein | - | - | - | CK945_RS17310 | ACH97_221330 |
| hypothetical protein | - | - | - | CK945_RS17495 | ACH97_221150 |
| ABC transporter ATP-binding protein | - | - | - | CK945_RS17500 | ACH97_221145 |
| hypothetical protein | - | - | - | CK945_RS18125 | ACH97_202150 |
| hypothetical protein | - | - | - | CK945_RS18145 | ACH97_202095 |
| hypothetical protein | - | - | - | CK945_RS18550 | ACH97_201675 |
| hypothetical protein | - | - | - | CK945_RS18675 | ACH97_218635 |
| hypothetical protein | - | - | - | CK945_RS18685 | ACH97_204655 |
| hypothetical protein | - | - | - | CK945_RS18690 | ACH97_204660 |
| hypothetical protein | - | - | - | CK945_RS18695 | ACH97_204665 |
| hypothetical protein | - | - | - | CK945_RS18705 | ACH97_204675 |
| hypothetical protein | - | - | - | CK945_RS18760 | ACH97_204715 |
| hypothetical protein | - | - | - | CK945_RS18780 | ACH97_204735 |
| hypothetical protein | - | - | - | CK945_RS18820 | ACH97_201530 |
| hypothetical protein | - | - | - | CK945_RS19025 | ACH97_201335 |
| glycosyl hydrolase | - | - | - | CK945_RS19305 | ACH97_201075 |
| sodium:solute symporter | - | - | - | CK945_RS19310 | ACH97_201070 |
| carboxylesterase | - | - | - | CK945_RS19460 | ACH97_200920 |
| hypothetical protein | - | - | - | CK945_RS19475 | ACH97_200905 |
| hypothetical protein | - | - | - | CK945_RS19700 | ACH97_200690 |
| glucuronoxylanase | - | - | - | CK945_RS19705 | ACH97_200685 |
| hypothetical protein | - | - | - | CK945_RS19830 | ACH97_200295 |
| hypothetical protein | - | - | - | CK945_RS19890 | ACH97_200240 |
| LysR family transcriptional regulator | - | - | - | CK945_RS20790 | ACH97_214465 |
| methyltransferase | - | - | - | CK945_RS20795 | ACH97_214460 |
| hypothetical protein | - | - | - | CK945_RS20860 | ACH97_214400 |
| AAA family ATPase | - | - | - | CK945_RS20865 | ACH97_214395 |
| hypothetical protein | - | - | - | CK945_RS20870 | ACH97_214390 |
| hypothetical protein | - | - | - | CK945_RS20875 | ACH97_214385 |
| transcriptional regulator | - | - | - | CK945_RS21005 | ACH97_214260 |
| epimerase | - | - | - | CK945_RS21010 | ACH97_214255 |
| hypothetical protein | - | - | - | CK945_RS21325 | ACH97_213940 |
| hypothetical protein | - | - | - | CK945_RS21330 | ACH97_213935 |
| hypothetical protein | - | - | - | CK945_RS21975 | ACH97_213310 |
| hypothetical protein | - | - | - | CK945_RS22145 | ACH97_213145 |
| pyridoxamine 5-phosphate oxidase | - | - | - | CK945_RS22290 | ACH97_213000 |
| hypothetical protein | - | - | - | CK945_RS22485 | ACH97_212815 |
| hypothetical protein | - | - | - | CK945_RS22655 | ACH97_212655 |
| transcriptional regulator | - | - | - | CK945_RS22955 | ACH97_212355 |
| ArsR family transcriptional regulator | - | - | - | CK945_RS22960 | ACH97_212350 |
| hypothetical protein | - | - | - | CK945_RS22965 | ACH97_212345 |
| fructose 1,6-bisphosphatase | - | - | - | CK945_RS23015 | ACH97_212285 |
| ABC transporter substrate-binding protein | - | - | - | CK945_RS23040 | ACH97_212270 |
| iron ABC transporter | - | - | - | CK945_RS23045 | ACH97_212265 |
| iron ABC transporter permease | - | - | - | CK945_RS23050 | ACH97_212260 |
| D-alanine/D-serine/glycine permease | - | - | - | CK945_RS23010 | ACH97_212305 |
| transcriptional regulator | - | - | - | CK945_RS03825 | ACH97_218730 |
| MFS transporter | - | - | - | CK945_RS12280 | ACH97_207215 |
