# Supplementary material for: Two genes involved in clindamycin resistance of Bacillus licheniformis and Bacillus paralicheniformis identified by comparative genomic analysis
Source: PLoS One. 2020 Apr 9;15(4):e0231274. doi: 10.1371/journal.pone.0231274 (PMC7144989; doi:10.1371/journal.pone.0231274)
Supplement: S3 Table — (DOCX) [file pone.0231274.s003.docx]

**S3 Table. List of CDSs commonly identified only in the genomes of C^R^E^S^ strains DSM 13^T^ and 14ADL4.**

|  | DSM 13^T^ | 14ADL4 | 0DA23-1 | 14DA11 | KJ-16^T^ |
| --- | --- | --- | --- | --- | --- |
| APC family permease | TRNA_RS24075 | BL14DL4_03117 | - | - | - |
| lysine transporter LysE | TRNA_RS24425 | BL14DL4_03183 | - | - | - |
| collagen-like protein | TRNA_RS43040 | BL14DL4_03393 | - | - | - |
| aspartate phosphatase | TRNA_RS26090 | BL14DL4_03515 | - | - | - |
| alpha/beta hydrolase | TRNA_RS43260 | BL14DL4_04428 | - | - | - |
| hypothetical protein | TRNA_RS32515 | BL14DL4_00443 | - | - | - |
| hypothetical protein | TRNA_RS32525 | BL14DL4_00444 | - | - | - |
| DNA (cytosine-5-)-methyltransferase | TRNA_RS36215 | BL14DL4_01182 | - | - | - |
| hypothetical protein | TRNA_RS36220 | BL14DL4_01183 | - | - | - |
| HAMP domain-containing protein | TRNA_RS37780 | BL14DL4_01507 | - | - | - |
| hypothetical protein | TRNA_RS43650 | BL14DL4_01733 | - | - | - |
| CDP-glycerol--glycerophosphate glycerophosphotransferase | TRNA_RS40270 | BL14DL4_02059 | - | - | - |
