# Supplementary material for: Two genes involved in clindamycin resistance of Bacillus licheniformis and Bacillus paralicheniformis identified by comparative genomic analysis
Source: PLoS One. 2020 Apr 9;15(4):e0231274. doi: 10.1371/journal.pone.0231274 (PMC7144989; doi:10.1371/journal.pone.0231274)
Supplement: S4 Table — (DOCX) [file pone.0231274.s004.docx]

**S4 Table. Predicted interaction sites of clindamycin and erythromycin in the 23S rRNA of *B. licheniformis* and *B. paralicheniformis*.**

| Phenotype | Strain | Position in the 23S rRNA sequence of *B. licheniformis* and *B. paralicheniformis* | | | | | | | | | | | |
| --- | --- | --- | --- | --- | --- | --- | --- | --- | --- | --- | --- | --- | --- |
|  |  | Clindamycin binding site | | | | | | Erythromycin binding site | | | | | |
|  |  | 2036  (G2057) | 2037  (A2058) | 2038  (A2059) | 2482  (A2503) | 2484  (G2505) | 2590  (C2611) | 2036  (G2057) | 2037  (A2058) | 2038  (A2059) | 2041  (A2062) | 2484  (G2505) | 2588  (U2609) |
| C^R^E^R^ | *B. paralicheniformis* 14DA11 | G | A | A | A | G | C | G | A | A | A | G | T |
|  | *B. paralicheniformis* KJ-16^T^ | G | A | A | A | G | C | G | A | A | A | G | T |
| C^R^E^S^ | *B. licheniformis* 14ADL4 | G | A | A | A | G | C | G | A | A | A | G | T |
|  | *B. licheniformis* DSM 13^T^ | G | A | A | A | G | C | G | A | A | A | G | T |
| C^S^E^S^ | *B. licheniformis* 0DA23-1 | G | A | A | A | G | C | G | A | A | A | G | T |

Corresponding position in the 23S rRNA sequence of *E. coli* is given in parenthesis.
