# Supplementary material for: Two genes involved in clindamycin resistance of Bacillus licheniformis and Bacillus paralicheniformis identified by comparative genomic analysis
Source: PLoS One. 2020 Apr 9;15(4):e0231274. doi: 10.1371/journal.pone.0231274 (PMC7144989; doi:10.1371/journal.pone.0231274)
Supplement: S1 Fig — (DOCX) [file pone.0231274.s005.docx]

**S1 Fig. Structures of the genes surrounding erythromycin resistance gene *ermC* in five *Bacillus* genomes.**


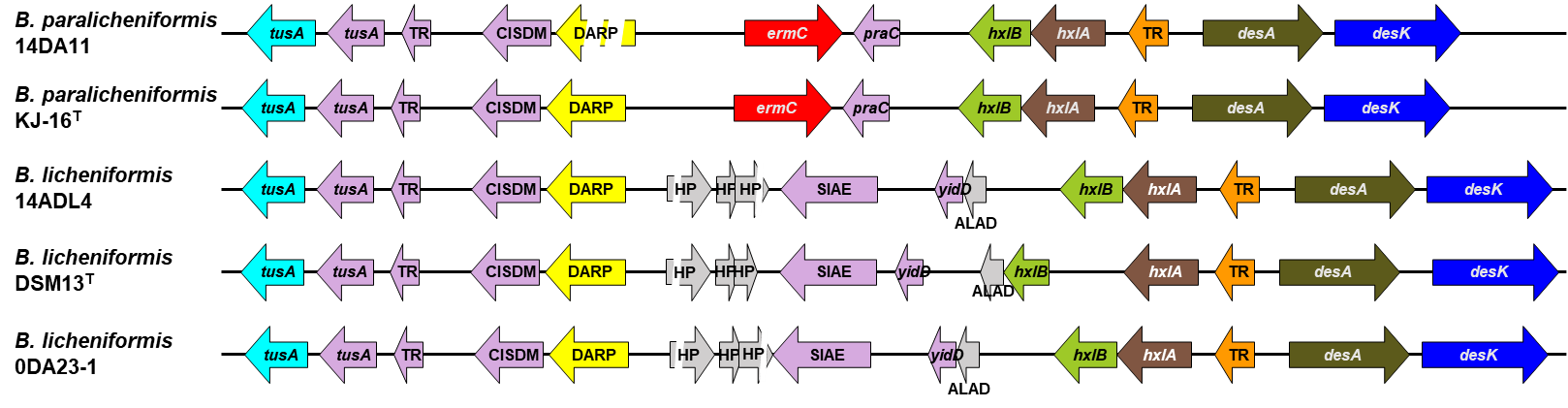


Abbreviations: ALAD, δ-aminolevulinic acid dehydratase gene; CISDM, class I SAM-dependent methyltransferase gene; DARP, DNA alkylation repair protein geme; *desA*, fatty acid desaturase gene; *desK*, sensor histidine kinase gene; *ermC*, 23S ribosomal RNA methyltransferase gene; HP, hypothetical protein gene; *hxlA*, 3-hexulose-6-phosphate synthase gene; *hxlB*, 6-phospho-3-hexuloisomerase gene; *praC*, tautomerase family protein gene; SIAE, sialate-O-acetylesterase gene; TR, transcriptional regulator gene; *yidD*, membrane protein insertion efficiency factor YidD gene; and *tusA*, sulfur carrier protein gene.
